# On the Moisture Absorption Capability of Ionic Liquids

**DOI:** 10.1021/acs.jpcb.4c02289

**Published:** 2024-06-14

**Authors:** Toshiyuki Itoh, Kentaro Kamada, Toshiki Nokami, Taiji Ikawa, Kenichi Yagi, Shuji Ikegami, Ryo Inoue, Andrew D. DeYoung, Hyung J. Kim

**Affiliations:** † Toyota Physical and Chemical Research Institute, 41-1 Yokomichi, Nagakute, Aichi 480-1192, Japan; ‡ Department of Chemistry and Biotechnology, Graduate School of Engineering, Tottori University, 4-101 Koyama-Minami, Tottori 680-8552, Japan; § Toyota Central R&D Laboratories, Inc., 41-1 Yokomichi, Nagakute, Aichi 480-1192, Japan; ∥ Technology and Innovation Center, Daikin Industries, Ltd., 1-1 Nishi-Hitotsuya, Settsu, Osaka 566-8585, Japan; ⊥ Department of Chemistry, Carnegie Mellon University, 4400 Fifth Avenue, Pittsburgh, Pennsylvania 15213, United States

## Abstract

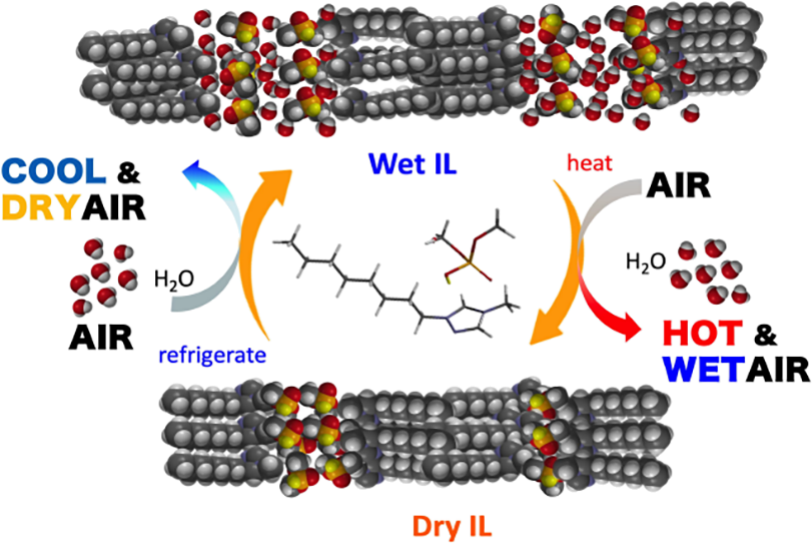

Due to their many attractive physicochemical properties, ionic liquids (ILs) have received extensive attention with numerous applications proposed in various fields of science and technology. Despite this, the molecular origins of many of their properties, such as the moisture absorption capability, are still not well understood. For insight into this, we systematically synthesized 24 types of ILs by the combination of the dimethyl phosphate anion with various types of alkyl group-substituted cyclic cations—imidazolium, pyrazolium, 1,2,3-triazolium, and 1,2,4-triazolium cations—and performed a detailed analysis of the dehumidification properties of these ILs and their aqueous solutions. It was found that these IL systems have a high dehumidification capability (DC). Among the monocationic ILs, the best performance was obtained with 1-cyclohexylmethyl-4-methyl-1,2,4-triazolium dimethyl phosphate, whose DC (per mol) value is 14 times higher than that of popular solid desiccants like CaCl_2_ and silica gel. Dicationic ILs, such as 1,1′-(propane-1,3-diyl)bis(4-methyl-1,2,4-triazolium) bis(dimethyl phosphate), showed an even better moisture absorption, with a DC (per mol) value about 20 times higher than that of CaCl_2_. Small- and wide-angle X-ray scattering measurements of eight types of 1,2,4-triazolium dimethyl phosphate ILs were performed and revealed that the majority of these ILs form nanostructures. Such nanostructures, which vary with the identity of the IL and the water content, fall into three main categories: bicontinuous microemulsions, hexagonal cylinders, and micelle-like structures. Water in the solutions exists primarily in polar regions in the nanostructures; these spaces function as water pockets at relatively low water concentrations. Since the structure and stability of the aggregated forms of the ILs are mainly governed by the interactions of nonpolar groups, the alkyl side chains of the cations play an important role in the DC and temperature-dependent equilibrium water vapor pressure of the IL solutions. Our experimental findings and molecular dynamics simulation results shed light on the moisture absorption mechanism of the IL aqueous solutions from a molecular perspective.

## Introduction

Ionic liquids (ILs) have attracted significant attention due to their interesting physical and chemical properties.^1^ Diverse applications have been reported for ILs during the past two decades as solvents for chemical and biocatalytic reactions^2−6^ and for electrochemical reactions,^7^ as media for the separation of functional molecules,^8^ as capture materials of carbon dioxide,^9^ and as bioactive compounds.^10^ One fascinating property of ILs is their moisture absorption capability,^11,12^ which enables ILs to be used as novel desiccants in liquid desiccant air conditioners (LDACs). This type of air conditioner is expected to contribute to the reinforcement of basic infrastructure because the electric energy consumption of LDACs is more than 20% lower than that of conventional compressor-type air conditioners.^11^ The most widely used desiccant for LDACs is presently an aqueous solution of lithium chloride (LiCl).^11−18^ However, LiCl aqueous solution is caustic to normal metals, such as iron, aluminum, and copper. Consequently, LDACs using LiCl-based desiccants require special corrosion-resistant pipes, thereby increasing production costs and preventing widespread adoption of LDACs.^18^ Moreover, lithium is limited in supply, largely due to its use in Li-ion batteries that power a wide range of modern electronic devices.^19^ Thus, it is desirable to find alternative desiccant materials that can replace Li salts.^11,12^

It is known that the hydrophilic and hygroscopic properties of ILs can be tuned by properly combining cations and anions.^20^ Moisture absorption from air by aqueous solutions of ILs was first studied by Lee and co-workers in 2004.^21^ In the following year, Kato and Gmehling reported that an aqueous solution of 1,3-dimethylimidazolium dimethyl phosphate ([C_1_mim][DMPO_4_]) exhibits a strong negative deviation from Raoult’s law and thus a very low water vapor pressure.^22^ Welton and co-workers observed that imidazolium ILs exposed to air can absorb moisture from it.^20^ They found that the incorporation rate of moisture into imidazolium ILs depends on the anionic species; specifically, the incorporation rate increases with the anion basicity in the order [PF_6_]^−^ < [SbF_6_]^−^ < [BF_4_]^−^ < [Tf_2_N]^−^ < [ClO_4_]^−^ < [OTf]^−^ < [NO_3_]^−^ < [TFA]^−^.^20^ Later, Mu and co-workers reported that the moisture absorption capability of the 1-butyl-3-methylimidazolium salts increases in the order [PF_6_]^−^ < [Tf_2_N]^−^ < [BF_4_]^−^ < [OTf]^−^ < [NO_3_]^−^ < [TFA]^−^ < Br^–^ < Cl^–^ < [OAc]^−^.^23^ These results suggest that these and related ILs can be used as desiccant and sorption material alternatives to LiCl, CaCl_2_, and similar salts.^11,12^

To the best of our knowledge, the first reports on the use of ILs as a desiccant source for LDACs were published by Luo et al., who demonstrated the feasibility of the ionic liquid 1-ethyl-3-methylimidazolium tetrafluoroborate ([C_2_mim][BF_4_]) as a desiccant.^24,25^ Subsequently, numerous reports^26−52^ and reviews^11,12^ on this topic were published. The most extensively investigated were the imidazolium ILs.^11,12^ Choline ILs were also studied; it was demonstrated that choline (2-hydroxy-*N,N,N-*trimethylethan-1-aminium) alkylcarboxylates ([Ch][RCO_2_]) can act as efficient desiccants.^26^ Nevertheless, the primary focus of many of these studies was not on the development of IL desiccants but on the design of efficient liquid desiccant equipment; some of them did not even report the structure of the ILs employed.^48−52^ Our own efforts, on the other hand, have been directed toward the design and optimization of ILs as liquid desiccants for LDACs. Our initial investigations of ammonium and phosphonium ILs as well as imidazolium and choline ILs revealed that tributyl(methyl)phosphonium dimethyl phosphate ([P_4441_][DMPO_4_])^33^ and choline dimethyl phosphate ([Ch][DMPO_4_])^35^ are characterized by excellent dehumidification capabilities (DCs). In our follow-up study, we examined the dehumidification properties, equilibrium water vapor pressures, viscosities, and corrosive effects of aqueous solutions of a range of dicationic quaternary ammonium bis(dimethyl) and bis(diethyl) phosphates by varying both the alkyl substituents and the spacer group in the dications.^38^

Understanding the mechanisms of moisture absorption of ILs at the molecular level is critical to guide the development and optimization of efficient desiccant ILs for LDACs. One of the key governing factors is undoubtedly the hydrogen-bonded interaction between the water and IL components.^23^ It was found that the moisture absorption behavior of the imidazolium ILs is mainly determined by the anions, though the cations also have some influence on the dehumidification capability.^53^ It was also found that the structure of the cationic species of the ILs can affect the temperature (*T*) dependence of the vapor pressure of their aqueous solutions.^11,12^ Related to this from the structural perspective is the formation of unique nanostructures in ILs through aggregations of the polar and nonpolar groups of ions.^54^ While the detailed understanding of how nanostructures modulate the various physical properties of ILs is still limited, nanostructures are expected to play an important role in the water absorption by ILs. For insight into this, we studied the influence of both the cations and water on the IL nanostructures by employing small- and wide-angle X-ray scattering (SWAXS) in combination with molecular dynamics (MD) simulations. In this paper, we report our findings for the 1,2,4-triazolium ILs and their aqueous solutions. Results for the DC and water vapor pressure of these solution systems, as well as aqueous solutions of other ILs, including the imidazolium, pyrazolium, and 1,2,3-triazolium ILs, are also presented.

## Experimental Section

### General Experimental Details

^1^H and ^13^C NMR spectra were recorded by a JNM-ECA500 (500 MHz for ^1^H and 125 MHz for ^13^C) and a Magritek Spinsolve 80 (80 MHz for ^1^H and 20 MHz for ^13^C). The chemical shifts are expressed in ppm downfield from tetramethylsilane (TMS) in CDCl_3_ as the internal reference. High-resolution mass spectra (HRMS) were recorded by a Thermo Fisher Scientific EXACTIVE mass spectrometer. For the small- and wide-angle X-ray scattering (SWAXS) analysis: samples were placed in fused silica capillaries of diameter 1.5 mm and were immediately sealed with an epoxy adhesive. The SWAXS measurements were conducted on the BL8S3 beamline at the Aichi Synchrotron Radiation Center, Japan. A monochromated X-ray beam with a wavelength of 0.92 Å was used to irradiate the samples at room temperature (rt). Scattering photons were recorded using the detectors PILATRUS 3S2M. The sample-to-detector distance was set at 0.45 m. The scattering intensity was recorded in the range of the scattering vector *q* from 0.02 to 3.01 Å^–1^, with *q* = |*q⃗*| = 4π sin θ λ^–1^ and the scattering angle of 2θ. The two-dimensional isotropic scattering patterns thus obtained were radially averaged to obtain one-dimensional scattering curves.

#### Synthesis of 1-Ethyl-2-methylpyrazolium Dimethyl Phosphate ([Pyra-1,2][DMPO_4_])

(1) Sodium hydride (310 mmol, 12.4 g of the ca. 60% mineral oil mixture) was placed in a 500 mL three-neck flask, connected to a Dimroth condenser and a 100 mL cylindrical funnel. After replacing the air inside the apparatus with argon gas, the mineral oil was removed by twice washing with dry hexane. Then, 100 mL of dry tetrahydrofuran (THF) was added at rt to form a slurry. A dry THF (100 mL) solution of 1,2-pyrazole (20.42 g, 300 mmol) at 0 °C was dropwise added (40 min) to this mixture through the cylindrical funnel while carefully removing the hydrogen gas. The resulting mixture was stirred for 1 h at rt, and then a THF (100 mL) solution of 1-iodoethane (51.47 g, 330 mmol) was dropwise added (30 min) at rt. After completing the addition of the 1-iodoethane solution, the mixture was stirred at 70 °C for 19 h to form the slurry. After allowing the mixture to cool to rt, the reaction was quenched by the addition of 5.58 g (310 mmol) of crushed ice at 0 °C. This content was then added to a 500 mL flask, and almost all of the solvent was removed by evaporation to afford a gray residue. The Claisen distillation of the resulting residue afforded 1-ethyl-1,2-pyrazole (21.17 g, 220 mmol) as a colorless liquid in 73% yield. ^1^H NMR (500 MHz, CDCl_3_, δ): 1.47 (3H, t, *J* = 7.2 Hz), 4.17 (2H, q, *J* = 7.2 Hz), 6.22 (1H, t, *J* = 2.4 Hz), 7.37 (1H, d, *J* = 4.0 Hz), 7.48 (1H, d, *J* = 2.4 Hz); ^13^C NMR (20 MHz, CDCl_3_, δ): 15.6, 45.8, 105.2, 128.1, 139.0; bp 72–75 °C/22 hPa.
(2) 1-Ethyl-1,2-pyrazole (20.60 g, 214 mmol) was mixed with trimethyl phosphate (33.10 g, 236 mmol) at rt, and the mixture was stirred at 120 °C under argon gas for 72 h. After allowing the mixture to cool to rt, the mixture was twice washed with hexane and ether and then dissolved in methanol (100 mL). Active charcoal (1.0 g) was added to this solution and stirred for 1 h at 50 °C. The charcoal was removed by filtration through a glass filter with a Celite pad and then dried under a reduced pressure of 7.1 hPa at 50 °C for 3 h to afford [Pyra-1,2][DMPO_4_] as a half-melted solid (49.29 g, 209 mmol) in 97% yield. ^1^H NMR (500 MHz, CDCl_3_, δ): 1.60 (3H, t, *J* = 7.2 Hz), 3.56 (3H, s), 3.71 (3H, s), 4.42 (2H, q, *J* = 7.2 Hz), 6.80 (1H, t, *J* = 2.4 Hz), 8.16 (2H, t, *J* = 3.2 Hz); ^13^C NMR (20 MHz, CDCl_3_, δ): 13.1, 36.5, 45.3, 53.0, 53.2, 107.3, 137.1, 139.6; HRMS (ESI) *m*/*z*: (M^+^) calcd for C_6_H_11_N_2_^+^, 111.09227; found, 111.092; *m*/*z*: (X^–^) calcd for C_2_H_6_O_4_P^–^, 125.000382; found, 124.9995.

Using the same method, we synthesized 1-butyl-2-methyl-1,2-pyrazolium dimethyl phosphate ([Pyra-1,4][DMPO_4_]) and 1-octyl-2-methyl-1,2-pyrazolium dimethyl phosphate ([Pyra-1,8][DMPO_4_]).

##### Pyra-1,4

^1^H NMR (500 MHz, CDCl_3_, δ): 0.87 (3H, t, *J* = 6.4 Hz), 1.23–1.60 (2H, m), 1.72–2.09 (2H, m), 3.42 (3H, s), 3.44 (3H, s), 4.28 (3H, s), 4.57 (2H, t, *J* = 6.4 Hz), 6.57 (1H, t, *J* = 2.4 Hz), 8.47 (1H, s), 8.84 (1H, d, *J* = 3.2 Hz); ^13^C NMR (20 MHz, CDCl_3_, δ): 13.5, 19.4, 31.3, 37.1, 50.0, 52.2, 52.5, 107.7, 137.8, 139.7; HRMS (ESI) *m*/*z*: (M^+^) calcd for C_8_H_15_N_2_^+^, 139.12359; found, 139.1221; *m*/*z*: (X^–^) calcd for C_2_H_6_O_4_P^–^, 125.000382; found, 124.9986.

##### Pyra-1,8

^1^H NMR (500 MHz, CDCl_3_, δ): 0.79 (3H, t, *J* = 5.6 Hz), 1.30–1.70 (10H, m), 1.76–2.50 (2H, m), 3.54 (3H, s), 3.41 (3H, s), 3.43 (3H, s), 4.19 (3H, s), 4.50 (2H, t, *J* = 6.4 Hz), 6.65 (1H, t, *J* = 3.2 Hz), 8.26 (1H, d, *J* = 3.2 Hz), 8.56 (1H, d, *J* = 3.2 Hz); ^13^C NMR (20 MHz, CDCl_3_, δ): 14.1, 22.6, 26.2, 29.1, 29.4, 31.7, 37.2, 50.3, 52.3, 52.6, 107.5, 137.7, 139.7; HRMS (ESI) *m*/*z*: (M^+^) calcd for C_12_H_23_N_2_^+^, 195.18623; found, 195.1847; *m*/*z*: (X^–^) calcd for C_2_H_6_O_4_P^–^, 125.000382; found, 124.9992.

#### Synthesis of 1-Ethyl-4-methyl-1,2,4-triazolium Dimethyl Phosphate ([124-Tz-1,2][DMPO_4_])

(1) Sodium methoxide (NaOMe: 10.86 g, 320 mmol) was placed in a 500 mL three-neck flask connected to a Dimroth condenser and a 100 mL cylindrical funnel. After replacing the air inside the apparatus with argon gas, 70 mL of dry methanol (MeOH) was added to form a clear solution. To this solution was dropwise (30 min) added a dry MeOH (50 mL) solution of 1*H*-1,2,4-triazole (13.81 g, 200 mmol) at 0 °C. The resulting mixture was stirred for 1 h at rt, and then, a dry MeOH (50 mL) solution of 1-bromoethane (23.97 g, 220 mmol) was dropwise added (30 min) at rt. After completing the addition of the 1-bromoethane solution, the mixture was stirred at 80 °C for 24 h to form a slurry. After allowing the mixture to cool to rt, the content was placed in a 500 mL flask, and almost all of the solvent was removed by evaporation to afford a white precipitate with an oily residue. The Claisen distillation of the resulting residue afforded 1-ethyl-1*H*-1,2,4-triazole (13.19 g, 136 mmol) as a colorless liquid in 68% yield: bp 53–55 °C/2.9 hPa; ^1^H NMR (500 MHz, CDCl_3_, δ): 1.46 (3H, t, *J* = 7.2 Hz), 4.18 (2H, q, *J* = 7.2 Hz), 7.87 (1H, s), 8.04 (1H, s); ^13^C NMR (20 MHz, CDCl_3_, δ): 15.0, 44.6, 142.2, 151.8.
(2) 1-Ethyl-1*H*-1,2,4-triazole (13.14 g, 135 mmol) was mixed with trimethyl phosphate (20.85 g, 149 mmol) at rt, and the mixture was then stirred at 120 °C for 24 h. After allowing the mixture to cool to rt, the mixture was washed with hexane and ether (twice) and evaporated to dryness to afford a dark brownish oil. The residue was then dissolved in 50 mL of MeOH. To this solution was added activated charcoal (1.00 g), and the resulting mixture was stirred at 50 °C for 1 h; then the activated charcoal was removed by filtration. Evaporation and drying under reduced pressure at 5.7 hPa and 50 °C for 5 h afforded [123-Tz-1,4][DMPO_4_] as a red-brown oil (32.11 g, 135 mmol) in quantitative yield. ^1^H NMR (80 MHz, CDCl_3_, δ): 1.63 (3H, t, *J* = 7.2 Hz), 3.53 (3H, s), 3.66 (3H, s), 4.20 (3H, s), 4.53 (2H, q, *J* = 7.2 Hz), 9.36 (1H, s), 11.53 (1H, s); ^13^C NMR (20 MHz, CDCl_3_, δ): 14.3, 34.5, 47.8, 52.5, 52.7, 144.4, 145.7; HRMS (ESI) *m*/*z*: (M^+^) calcd for C_5_H_10_N_3_^+^, 112.08751; found, 112.0874; *m*/*z*: (X^–^) calcd for C_2_H_6_O_4_P^–^, 125.000382; found, 124.9986.

Using the same method, we synthesized 1-butyl-4-methyl-1,2,4-triazolium dimethyl phosphate ([124-Tz-1,4][DMPO_4_]), 1-hexyl-4-methyl-1,2,4-triazolium dimethyl phosphate ([124-Tz-1,6][DMPO_4_]), 1-octyl-4-methyl-1,2,4-triazolium dimethyl phosphate ([124-Tz-1,8][DMPO_4_]), 1-decyl-4-methyl-1,2,4-triazolium dimethyl phosphate ([124-Tz-1,10][DMPO_4_]), 1-tetradecyl-4-methyl-1,2,4-triazolium dimethyl phosphate ([124-Tz-1,14][DMPO_4_]), 1-cyclohexylmethyl-4-methyl-1,2,4-triazolium dimethyl phosphate ([124-Tz-1,c6][DMPO_4_]), and 1-(2-ethylhexyl)-4-methyl-1,2,4-triazolium dimethyl phosphate ([124-Tz-1,(2-Et)6][DMPO_4_]).

##### 124-Tz-1,4

^1^H NMR (500 MHz, CDCl_3_, δ): 0.99 (3H, t, *J* = 6.4 Hz), 1.19–1.54 (2H, m), 1.64–2.15 (2H, m), 3.53 (3H, s), 3.62 (3H, s), 4.19 (3H, s), 4.46 (2H, t, *J* = 6.4 Hz), 9.36 (1H, s), 11.63 (1H, s); ^13^C NMR (20 MHz, CDCl_3_, δ): 13.4, 19.4, 30.9, 34.5, 52.2, 52.4, 52.7, 144.8, 145.7; HRMS (ESI) *m*/*z*: (M^+^) calcd for C_7_H_14_N_3_^+^, 140.11883; found, 140.1183; *m*/*z*: (X^–^) calcd for C_2_H_6_O_4_P^–^, 125.000382; found, 124.9986.

##### 124-Tz-1,6

^1^H NMR (500 MHz, CDCl_3_, δ): 0.94 (3H, t, *J* = 5.6 Hz), 1.25–1.75 (6H, m), 1.70–2.25 (2H, m), 3.44 (3H, s), 3.57 (3H, s), 4.23 (3H, s), 4.49 (2H, *J* = 7.2 Hz), 9.4 (1H, s), 11.5 (1H, s); ^13^C NMR (20 MHz, CDCl_3_, δ): 14.0, 22.4, 25.9, 29.0, 31.3, 52.5, 52.8, 52.8, 144.7, 145.8; HRMS (ESI) *m*/*z*: (M^+^) calcd for C_9_H_18_N_3_^+^, 166.15015; found, 166.1494; *m*/*z*: (X^–^) calcd for C_2_H_6_O_4_P^–^, 125.000382; found, 124.9992.

##### 124-Tz-1,8

^1^H NMR (80 MHz, CDCl_3_, δ): 0.92 (3H, t, *J* = 6.4 Hz), 1.2–1.6 (10H, brs), 1.9–2.2 (4H, m), 3.56 (3H,s), 3.69 (3H, s), 4.22 (3H, s), 4.47 (2H, t, *J* = 6.4 Hz), 9.43 (1H,s), 11.55 (1H, s); ^13^C NMR (20 MHz, CDCl_3_, δ): 14.1, 22.6, 26.2, 29.1, 31.7, 34.5, 52.5, 52.8, 144.7, 145.8; HRMS (ESI) *m*/*z*: (M^+^) calcd for C_11_H_22_N_3_^+^, 196.18147; found, 196.1813; *m*/*z*: (X^–^) calcd for C_2_H_6_O_4_P^–^, 125.000382; found, 124.9987.

##### 124-Tz-1,10

^1^H NMR (80 MHz, CDCl_3_, δ): 0.95 (3H, t, *J* = 5.6 Hz), 1.25–1.75 (17H, m), 1.75–2.25 (2H, m), 3.57 (3H, s), 3.70 (3H, s), 4.25 (3H, s), 4.47 (2H, t, *J* = 5.6 Hz), 9.50 (1H, s), 11.5 (1H, s); ^13^C NMR (20 MHz, CDCl_3_, δ): 14.2, 22.7, 26.3, 29.1, 29.3, 29.5, 31.9, 34.5, 52.5, 52.8, 144.6, 146.0; HRMS (ESI) *m*/*z*: (M^+^) calcd for C_13_H_26_N_3_^+^, 224.21279; found, 224.212; *m*/*z*: (X^–^) calcd for C_2_H_6_O_4_P^–^, 125.000382; found, 124.9994.

##### 124-Tz-1,14

^1^H NMR (80 MHz, CDCl_3_, δ): 0.98 (3H, t, *J* = 6.4 Hz), 1.20–1.75 (22H, brs), 2.0 (2H, brs), 3.62 (3H, s), 3.75 (3H, s), 4.23 (3H. S), 4.52 (2H, t, *J* = 6.4 Hz), 9.02 (1H, s), 11.2 (1H, s); ^13^C NMR (20 MHz, CDCl_3_, δ): 14.2, 22.8, 26.3, 29.1, 29.4, 29.5, 29.7, 32.0, 34.6, 52.6, 52.9, 144.6, 145.4; HRMS (ESI) *m*/*z*: (M^+^) calcd for C_17_H_34_N_3_^+^, 280.27543; found, 280.2744; *m*/*z*: (X^–^) calcd for C_2_H_6_O_4_P^–^, 125.000382; found, 124.9993.

##### 124-Tz-1,c6

^1^H NMR (80 MHz, CDCl_3_, δ): 0.75–1.20 (5H, m), 1.20–20.0 (6H, m), 3.40 (3H, s), 3.52 (3H, s), 3.87 (3H, s), 4.16 (2H, d, *J* = 6.4 Hz), 8.70 (1H, s), 9.64 (1H, s); ^13^C NMR (20 MHz, CDCl_3_, δ): 24.9, 25.2, 25.5, 29.4, 34.0, 37.0, 52.8, 52.9, 58.0, 142.5, 145.4; HRMS (ESI) *m*/*z*: (M^+^) calcd for C_10_H_18_N_3_^+^, 180.15015; found, 180.1493; *m*/*z*: (X^–^) calcd for C_2_H_6_O_4_P^–^, 125.000382; found, 124.9992.

##### 124-Tz-1,(2-Et)6

^1^H NMR (80 MHz, CDCl_3_, δ): 10.94 (3H, t, *J* = 5.6), 1.25–1.75 (6H, brs), 1.70–2.25 (2H, m), 3.44 (3H, s), 3.57 (3H, s), 4.23 (3H, s), 4.49 (2H, t, *J* = 7.2), 9.40 (1H, s), 11.46 (1H, s); ^13^C NMR (20 MHz, CDCl_3_, δ): 9.6, 13.3, 22.2, 22.8, 27.7, 29.3, 34.0, 38.6, 52.8, 52.9, 55.5, 144.7, 145.8; HRMS (ESI) *m*/*z*: (M^+^) calcd for C_11_H_22_N_3_^+^, 196.18147; found, 196.1804; *m*/*z*: (X^–^) calcd for C_2_H_6_O_4_P^–^, 125.000382; found, 124.9992.

#### Synthesis of 1-Butyl-3-methyl-1,2,3-triazolium Dimethyl Phosphate ([123-Tz-1,4][DMPO_4_])

1-Butyl-1*H*-1,2,3-triazole was prepared by the reaction of 1*H*-1,2,3-triazole with iodobutane as a colorless liquid. ^1^H NMR (80 MHz, CDCl_3_, δ): 0.98 (3H, t, *J* = 7.2 Hz), 1.20–1.62 (2H, m), 1.76–2.07 (2H, m), 4.44 (2H, t, *J* = 7.2 Hz), 7.66 (1H, s), 7.71 (1H, s); ^13^C NMR (20 MHz, CDCl_3_, δ): 13.5, 19.7, 32.3, 49.9, 123.4, 133.7.

1-Butyl-1*H*-1,2,3-triazole (9.45 g, 75.5 mmol) was mixed with trimethyl phosphate (11.63 g, 83 mmol) at rt, and the mixture was stirred at 120 °C for 24 h. After allowing the mixture to cool to rt, the mixture was washed with hexane and ether (twice) and then evaporated to dryness to afford a dark brownish oil. The residue was next dissolved in 50 mL of MeOH. To this solution was added activated charcoal (1.00 g), and the resulting mixture was stirred at 50 °C for 1 h. The activated charcoal was then removed by filtration. Evaporation of the filtrate and drying under reduced pressure at 4.8 hPa and 50 °C for 5 h afforded [123-Tz-1,4][DMPO_4_] as a red-brown oil (16.47 g, 62.1 mmol) in 83% yield. ^1^H NMR (80 MHz, CDCl_3_, δ): 1.07 (3H, t, *J* = 6.4 Hz), 1.25–1.73 (2H, m), 1.92–2.28 (2H, m), 3.61 (3H, s), 3.74 (3H, s), 4.58 (3H, s), 4.84 (2H, q, *J* = 6.4 Hz), 9.90 (2H, s); ^13^C NMR (20 MHz, CDCl_3_, δ): 13.4, 19.5, 31.7, 40.1, 52.4, 52.7, 53.6, 132.5, 133.3; HRMS (ESI) *m*/*z*: (M^+^) calcd for C_7_H_14_N_3_^+^, 140.11883; found, 140.1183; *m*/*z*: (X^–^) calcd for C_2_H_6_O_4_P^–^, 125.000382; found, 124.9987.

Using the same method, we synthesized [123-Tz-1,2][DMPO_4_]. ^1^H NMR (80 MHz, CDCl_3_, δ): 1.69 (3H, t, *J* = 7.2 Hz), 3.54 (3H, s), 3.68 (3H, s), 4.49 (3H, s), 4.82 (2H, q, *J* = 7.2 Hz), 9.56 (1H, s), 9.61 (1H, s); ^13^C NMR (20 MHz, CDCl_3_, δ): 14.8, 40.1, 49.3, 52.6, 52.9, 131.9, 133.0; HRMS (ESI) *m*/*z*: (M^+^) calcd for C_5_H_10_N_3_^+^, 112.08751; found, 112.0873; *m*/*z*: (X^–^) calcd for C_2_H_6_O_4_P^–^, 125.000382; found, 124.9985.

#### Synthesis of 4-Butyl-1-ethyl-3-methyl-1,2,3-triazolium Dimethyl Phosphate ([123-Tz-1,2,4][DMPO_4_])

(1) To a 500 mL three-neck flask that was connected to a Dimroth condenser and a 100 mL cylindrical funnel was added sodium azide (26.0 g, 400 mmol); then, the inside air was replaced by argon gas. To this flask was added 80 mL of dry *N*,*N*-dimethylformamide to afford a colorless solution. To this solution was dropwise (30 min) added a DMF (20 mL) solution of bromoethane (21.8 g, 200 mmol) through the 100 mL cylindrical funnel at 0 °C, and then, the mixture was stirred at 80 °C for 24 h to afford a white slurry. After allowing the mixture to cool to rt, CuI powder (3.81 g, 20 mmol) was added in one portion through the bypass; this caused a color change of the mixture to a red-brownish slurry. The mixture was diluted with 50 mL of dry DMF; then, 50 mL of a DMF solution of 1-hexyne (18.07 g, 220 mmol) was added to this mixture at rt to afford a gray slurry. The mixture was stirred at 80 °C for 24 h. After allowing the mixture to cool to rt, the gray precipitate was removed by filtration through a glass filter on a Celite pad to give a dark green solution. The solution was diluted with ethyl acetate to form a white precipitate CuI, which was removed by filtration. The filtrate was washed three times with water and then evaporated and dried under reduced pressure at 7.8 hPa and 40 °C for 2 h to afford 4-butyl-1-ethyl-1*H*-1,2,3-triazole (26.2 g, 171 mmol, bp 97 °C, 4.6 hPa) as a coloress liquid in 85% yield. ^1^H NMR (80 MHz, CDCl_3_, δ): 0.98 (3H, t, *J* = 6.4 Hz), 1.33–1.89 (4H, m), 1.58 (3H, t, *J* = 7.2 Hz), 2.77 (2H, t, *J* = 6.4 Hz), 4.42 (2H, q, *J* = 7.2 Hz), 7.39 (1H, s); ^13^C NMR (20 MHz, CDCl_3_, δ): 13.9, 15.6, 22.4, 25.5, 31.7, 45.1, 120.1, 148.4.
(2) 4-Butyl-1-ethyl-1*H*-1,2,3-triazole (26.2 g, 171 mmol) was mixed with trimethyl phosphate (28.70 g, 205 mmol) at rt, and then, the mixture was stirred for 24 h at 120 °C. After allowing the mixture to cool to rt, the mixture was twice washed with hexane and ether and dissolved in methanol (50 mL). To this solution was added 1.0 g of activated charcoal and stirred for 1 h at 50 °C. The activated charcoal was then removed by filtration and dried by evaporation under reduced pressure at 5.4 hPa and 50 °C for 4 h to afford [123-Tz-1,2,4][DMPO_4_] (33.1 g, 113 mmol) as a light brownish oil in 91% yield. ^1^H NMR (500 MHz, CDCl_3_, δ): 0.98 (3H, t, *J* = 7.3 Hz), 1.47–1.49 (2H, m), 1.67 (3H, t, *J* = 6.7 Hz), 1.75–1.85 (2H, m), 2.89 (2H, t, *J* = 7.8 Hz), 3.58 (6H, s), 4.22 (3H, s), 4.83 (2H, q, *J* = 7.1 Hz), 9.70 (1H, s); ^13^C NMR (125 MHz, CDCl_3_, δ): 13.6, 14.7, 22.2, 23.1, 29.1, 37.6, 49.3, 52.5, 130.3, 144.3; HRMS (ESI) (M^+^) calcd for C_9_H_18_N_3_^+^, 168.15015; found, 168.1486; *m*/*z*: (M^–^) calcd for C_2_H_6_O_4_P^–^, 125.0004; found, 124.9990; mp −77.4 °C (DSC); the 10% decomposition temperature was 204 °C (TG-DTA).

Using the same method, we prepared 1,4-dibutyl-3-methyl-1,2,3-triazolium dimethyl phosphate ([123-Tz-1,4,4][DMPO_4_]) and 1-(2-methoxy)ethyl-3-methyl-4-butyl-1,2,3-triazolium dimethyl phosphate ([123-Tz-1,4,ME][DMPO_4_]).

##### [123-Tz-1,4,4][DMPO_4_]

Mp −65.7 °C (DSC); the 10% decomposition temperature was 205 °C (TG-DTA); ^1^H NMR (80 MHz, CDCl_3_, δ): 1.05 (3H, t, *J* = 6.4 Hz), 1.28–2.19 (8H, m), 3.02 (2H, t, *J* = 6.4 Hz), 3.56 (3H, s), 3.69 (3H, s), 4.37 (3H, s), 4.82 (2H, t, *J* = 7.2 Hz), 0, 9.56 (1H, s); ^13^C NMR (20 MHz, CDCl_3_, δ): 13.5, 13.7, 19.6, 22.3, 23.2, 29.3, 31.5, 37.7, 52.3, 52.6, 53.7, 130.4, 144.5; HRMS (ESI) *m*/*z*: (M^+^) calcd for C_11_H_22_N_3_^+^, 196.18147; found, 196.1797; *m*/*z* (X^–^) calcd for C_2_H_6_O_4_P^–^, 125.0004; found, 124.9989.

##### [123-Tz-1,4,ME][DMPO_4_]

Mp −61.7 °C (DSC); ^1^H NMR (80 MHz, CDCl_3_, δ): 1.01 (3H, t, *J* = 6.4 Hz), 1.29–2.00 (4H, m), 2.93 (2H, t, *J* = 7.2 Hz), 3.97 (2H, t, *J* = 6.4 Hz), 3.39 (3H, s), 3.52 (3H, s), 3.65 (3H, s), 4.30 (3H, s), 9.75 (1H, s); ^13^C NMR (20 MHz, CDCl_3_, δ): 13.6, 22.3, 23.2, 29.1, 37.5, 52.2, 52.5, 53.7, 58.8, 69.6, 130.7, 144.35; HRMS (ESI) *m*/*z*: (M^+^) calcd for C_10_H_20_N_3_O^+^, 196.16072; found, 198.1601; *m*/*z*: (X^–^) calcd for C_2_H_6_O_4_P^–^, 125.0004; found, 124.9986.

#### Synthesis of 1,1′-(Hexane-1,6-diyl)bis(3-methylimidazol-3-ium) Bis(dimethyl phosphate) ([Bis(MeIm)C6][DMPO_4_]_2_)

(1) Sodium hydride (8.14 g of the ca. 60% mineral oil mixture, 204 mmol) was placed in a 500 mL three-neck flask connected to a Dimroth condenser and a 100 mL cylindrical funnel. After replacing the air inside the apparatus with argon gas, the mineral oil was removed by twice washing with dry hexane twice. The addition of 100 mL of dry THF formed a slurry. A dry THF (100 mL) solution of 1*H*-imidazole at 0 °C was dropwise added (40 min) to the solution through the cylindrical funnel with careful removal of the hydrogen gas. The resulting mixture was stirred for 1 h at rt; then a THF (50 mL) solution of 1,6-dibromohexane (26.10 g, 107 mmol) was added dropwise (30 min) at rt. After completing the addition of 1,6-dibromohexane, the mixture was stirred at 70 °C for 24 h to form a white slurry. After allowing the mixture to cool to rt, the reaction was quenched by the addition of 1.10 g of crushed ice at 0 °C; then the content was placed in a 500 mL flask, and anhydrous sodium sulfate was added and stirred at rt for 30 min. After removal of sodium sulfate, the filtrate was evaporated to afford a gray residue. Since the boiling point of 1,6-di(1*H*-imidazol-1-yl)hexane was high, direct Claisen distillation was unsuccessful. Hence, we performed Kugelrohr distillation repeated three times to afford 1,6-di(1*H*-imidazol-1-yl)hexane (18.10 g, 83 mmol) as a colorless liquid in 78% yield. ^1^H NMR (80 MHz, CDCl_3_, δ): δ 1.30–1.48 (4H, m), 1.50–1.94 (4H, m), 3.88 (4H, t, *J* = 6.4 Hz), 6.98 (2H, d, *J* = 4.8 Hz), 7.17 (2H, d, *J* = 4.8 Hz), 7.54 (2H, s); ^13^C NMR (20 MHz, CDCl_3_, δ): 26.1, 31.0, 46.9, 119.0, 122.0, 129.4, 137.1; bp 196 °C/4.8 hPa (Kugelrohr).
(2) A 100 mL flask was connected to a Dimroth condenser and a 100 mL cylindrical flask in which the air was replaced by argon gas. To this flask were added 1,6-di(1*H*-imidazol-1-yl)hexane (4.05 g, 18.6 mmol) and trimethyl phosphate (5.71 g, 40.8 mmol) at rt; then the mixture was stirred at 120 °C for 24 h. After allowing the mixture to cool to rt, the content was twice washed with hexane and ether and then dissolved in water (50 mL). To this solution was added 1.0 g of activated charcoal, which was stirred for 1 h at 50 °C, then lyophilized and dried under reduced pressure at 7.4 hPa for 5 h to afford [Bis(MeIm)C6][DMPO_4_] (9.23 g, 18.5 mmol) as a light yellow syrup in 97% yield. ^1^H NMR (80 MHz, D_2_O, δ): 1.21–1.40 (4H, m), 1.68–1.93 (4H, m), 3.49 (6H, s), 3.62 (6H, s), 3.87 (6H, s), 4.16 (4H, t, *J* = 7.2 Hz), 7.38–7.47 (4H, m), 8.68 (2H, brs); ^13^C NMR (20 MHz, D_2_O, δ): 24.9, 29.1, 35.7, 49.4, 52.7, 53.0, 122.2, 123.6, 135.9; HRMS (ESI) *m*/*z*: (M^2+^) calcd for C_14_H_24_N_42_^2+^, 248.2002/124.1001; found, 124.0966 (*Z* = +2); *m*/*z*: (X^–^) calcd for C_2_H_6_O_4_P^–^, 125.000382; found, 124.9995; mp (DSC) −45.4 °C (*T*_g_).

Using the same method, we synthesized [Bis(MeIm)C3][DMPO_4_]_2_. ^1^H NMR (80 MHz, D_2_O, δ): 2.36–2.72 (4H, m), 3.50 (6H, s), 3.63 (6H, s), 3.93 (6H, s), 4.34 (4H, t, *J* = 6.4 Hz), 7.45–7.58 (4H, m), 8.82 (2H, brs); ^13^C NMR (20 MHz, D_2_O, δ): 29.8, 35.9, 46.4, 52.7, 53.1, 122.2, 124.1, 136.3; HRMS (ESI) *m*/*z*: (M^2+^) calcd for C_11_H_18_N_4_^2+^, 206.15322/103.07661; found, 103.0752 (*Z* = +2); *m*/*z*: (X^–^) calcd for C_2_H_6_O_4_P^–^, 125.000382; found, 124.9995; mp (DSC) −43.0 °C (*T*_g_).

#### Synthesis of 4,4′-(Hexane-1,6-diyl)bis(1-methyl-1,2,4-triazol-1-ium) Bis(dimethyl phosphate) ([Bis(124-Tz-1)C6][DMPO_4_]_2_)

This salt was synthesized using 1,6-dibromohexane and trimethyl phosphate in 81% yield as a dark brown solid. ^1^H NMR (80 MHz, D_2_O, δ): 1.10–1.60 (4H, m), 1.79–2.05 (4H, m), 3.51 (6H, s), 3.64 (6H, s), 3.98 (6H, s), 4.42 (4H, t, *J* = 7.2 Hz), 8.81 (4H, s), 9.75(4H, s);^13^C NMR (20 MHz, D_2_O, δ): 24.8, 27.9, 34.0, 52.3, 52.8, 53.1, 142.5, 145.5; HRMS (ESI) *m*/*z*: (M^2+^) calcd for C_12_H_22_N_6_^2+^, 250.19068/125.09534; found, 125.1900 (*Z* = +2); *m*/*z*: (X^–^) calcd for C_2_H_6_O_4_P^–^, 125.000382; found, 124.9995.

Using the same method, we synthesized [bis(124-Tz-1)C3][DMPO_4_]_2_). ^1^H NMR (80 MHz, D_2_O, δ): 3.14–3.67 (2H, m), 3.47 (6H, s), 3.60 (6H, s), 3.86 (6H, s), 4.55 (4H, t, *J* = 7.2 Hz), 8.83–8.90 (4H, brs); ^13^C NMR (20 MHz, D_2_O, δ): 22.7, 34.1, 48.7, 52.7, 52.9, 145.7; HRMS (ESI) *m*/*z*: (M^2+^) calcd for C_9_H_16_N_6_^2+^, 208.1437/104.007185; found, 104.0106 (*Z* = +2); *m*/*z*: (X^–^) calcd for C_2_H_6_O_4_P^–^, 125.000382; found, 124.9995.

#### Synthesis of 1,1′-(Hexane-1,6-diyl)bis(2-methyl-pyrazol-1-ium) Bis(dimethyl phosphate) ([Bis(Pyra-1)C6][DMPO4]_2_)

Using the same method, we synthesized [Bis(Pyra-1)C6][DMPO_4_]_2_ in 89% yield (2 steps) as a white powder. ^1^H NMR (80 MHz, D_2_O, δ): 1.17–1.50 (4H, m), 1.72–2.20 (4H, m), 3.51 (6H, s), 3.64 (6H, s), 4.10 (6H, s), 6.78 (4H, d, *J* = 1.6 Hz), 8.13–8.22 (4H, m); ^13^C NMR (20 MHz, D_2_O, δ): 24.9, 27.9, 49.8, 52.7, 53.2, 107.3, 136.7, 138.0; HRMS (ESI) *m*/*z*: (M^2+^) calcd for C_14_H_24_N_4_^2+^, 248.2002/124.1001; found, 124.0995 (*Z* = +2); *m*/*z*: (X^–^) calcd for C_2_H_6_O_4_P^–^, 125.000382; found, 124.9995; mp (DSC) 179.8, 198.9 °C (*T*_g_).

Bis(Pyra-1)C3 was also synthesized by the same method in similar yield. ^1^H NMR (80 MHz, D_2_O, δ): (2H, q, *J* = 7.2 Hz), 3.50 (6H, s), 3.64 (6H, s), 3.87 (6H, s), 4.58 (4H, t, *J* = 7.2 Hz), 8.86 (2H, s), 9.84 (2H, s); ^13^C NMR (20 MHz, D_2_O, δ): 27.7, 34.2, 48.8, 52.9, 53.1, 143.2, 145.8; HRMS (ESI) *m*/*z*: (M^2+^) calcd for C_11_H_18_N_4_^2+^, 206.15322/103.07661; found, 103.0762 (*Z* = +2); *m*/*z*: (X^–^) calcd for C_2_H_6_O_4_P^–^, 125.000382; found, 124.9994; mp (DSC) −55.1 °C (*T*_g_).

For measurements of the DC and the equilibrium water vapor pressure, we used the same method that we previously reported.^38^

### Computer Simulation Details

The [124-Tz-1,8] cation was modeled using the bonded and Lennard-Jones parameters from the force field for dialkylimidazolium cations developed by Lopes et al.^55,56^ Partial charges for the cation’s polar head group (i.e., the triazolium ring) were obtained by the CHELPG method^57^ at the MP2/cc-pVTZ(−f)//HF/6-31G(d) level using the Gaussian 16 program,^58^ while the Lopes force field charges^55,56^ were used for the nonpolar tail. The [DMPO_4_] anion was modeled with bonded and Lennard-Jones parameters from the OPLS-AA force field.^59^ Partial charges for [DMPO_4_] were determined by the CHELPG method^57^ at the MP2/cc-pVTZ(−f)//HF/6-31G(d) level. The TIP4P/2005 model^60^ was used to model the water molecule, present in the TZ8_50 and TZ8_80 systems. The TZ8_50 system consisted of 488 TZ8 ([124-Tz-1,8][DMPO_4_]) ion pairs and 8705 water molecules, a 50% (w/w) aqueous solution of TZ8. The TZ8_80 system consisted of 488 TZ8 ion pairs and 2176 water molecules, i.e., an 80% (w/w) aqueous solution of TZ8. The TZ8_100 system (pure IL) consisted of 1000 TZ8 ion pairs only. The MD simulations were performed using the GROMACS package^61^ with a time step of 1 fs; long-range Coulomb interactions were included using the particle mesh Ewald method.^62^ For each system, annealing and equilibration were performed for 15 ns in the isothermal–isobaric (*NPT*) ensemble using the Parrinello–Rahman barostat^63^ (at a pressure of 1 bar) and the Nosé–Hoover thermostat;^64,65^ the temperature was gradually reduced from *T* = 1000 K to the target temperature of *T* = 350 K in order to obtain the thermodynamically correct simulation box size. Subsequently, an additional 15 ns of equilibration, followed by production runs of 10 ns, was performed in the canonical (*NVT*) ensemble using the Nosé–Hoover thermostat at *T* = 350 K.

## Results and Discussion

### Dehumidification Capability of ILs

The dehumidification capability of ILs is a crucial factor in their application as desiccant and sorption materials. A prior study indicated that water molecules are incorporated more easily into weakly associative ILs than into strongly associative ILs.^23^ To understand this, we previously conducted a thermophysical investigation of many different types of monocationic and dicationic quaternary ammonium salts (mono-QAs and di-QAs).^35,38^ The latter includes *N*^1^,*N*^1^,*N*^1^,*N*^2^,*N*^2^,*N*^2^-hexamethylethane-1,2-diaminium bis(dimethyl phosphate) (HMC2),^35,38^*N*^1^,*N*^1^,*N*^1^,*N*^3^,*N*^3^,*N*^3^*-*hexamethylpropane-1,3-diaminium bis(dimethyl phosphate) (HMC3),^38^*N*^1^,*N*^1^,*N*^1^,*N*^6^,*N*^6^,*N*^6^-hexamethylhexane-1,6-diaminium bis(dimethyl phosphate) (HMC6), and their variants.^38^ We found that the di-QAs exhibit a high DC that varies with the length of the spacer group between their terminal cationic moieties but not with the length of the alkyl group of the anions.^38^ In addition, while the equilibrium water vapor pressure (Pv) of aqueous solutions of the mono- and di-QAs was found to vary considerably with the IL at a high *T* (50 °C), no significant IL dependence of Pv was observed at a low *T* (25 °C).^38^ Another interesting result was that the activation energy of vaporization, *E*_a_, is nearly the same (∼51 kJ/mol) for 80% (w/w) aqueous solutions of the di-QAs, HMC2, HMC3, and HMC6 (see Figure S5 in the Supporting Information). This implies that the Pv values differ due to the pre-exponential factor *A*_0_, a frequency factor (more generally, an entropic effect) often closely related to the molecular shape.^66^ Similar results were obtained for the 80% (w/w) aqueous solutions of two imidazolium ILs, 1-ethyl-3-methylimidazolium (C_2_mim) and 1-butyl-3-methylimidazolium (C_4_mim) dimethyl phosphate (Figure S6 in the Supporting Information). The experimental results for *E*_a_ in HMC2, HMC3, and HMC6 solutions are in good agreement with the MD results for the partial molar internal energy of water.^38^ These findings indicate that the structure of the cation plays an important role in Pv.

**Figure 1:**
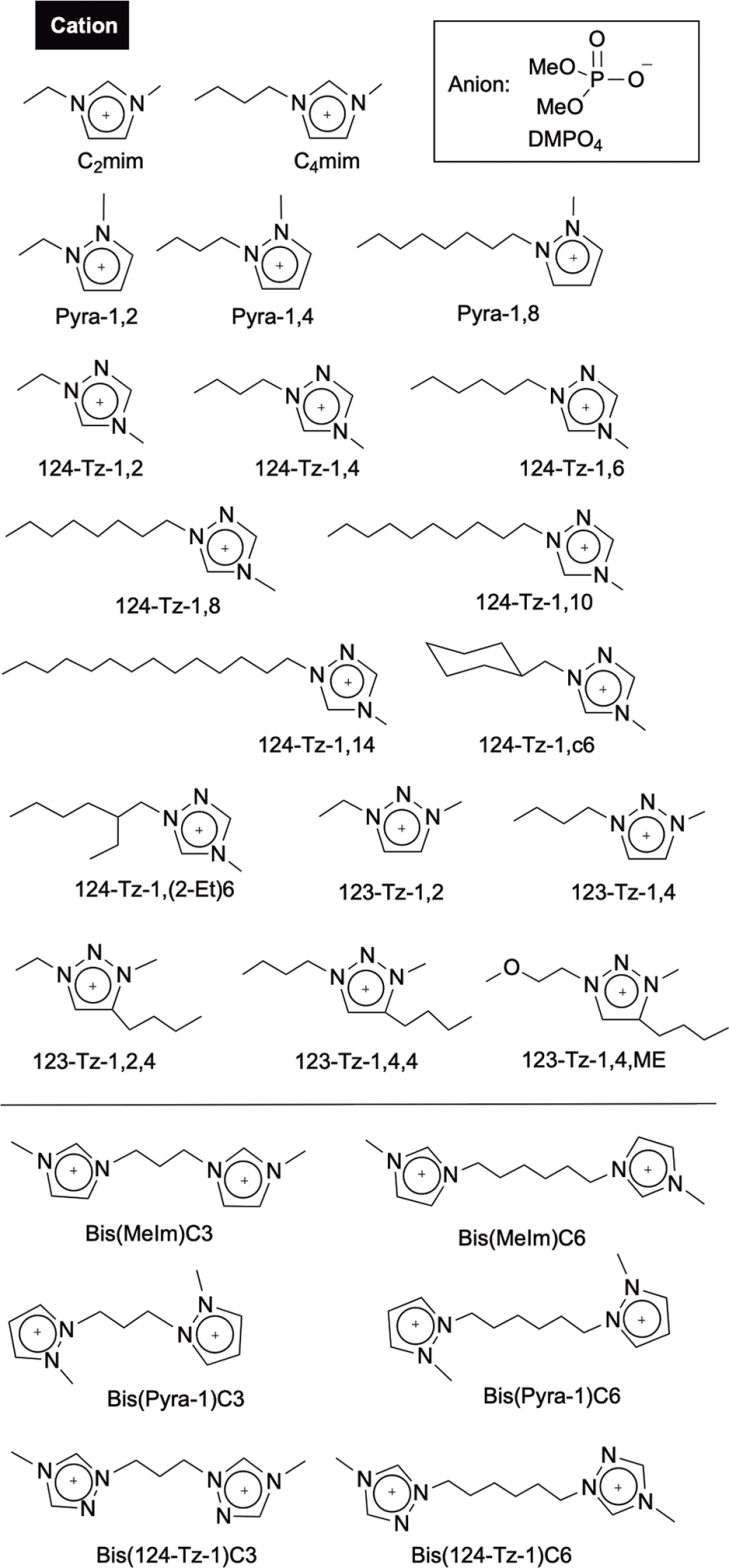
List of ILs investigated for their dehumidification capabilities in the present study.

In the present study, we analyzed the DC and *T*-dependent Pv of IL solutions. The ILs employed consisted of the dimethyl phosphate anion paired with five types of 5-membered cyclic cations: imidazolium, pyrazolium, 1,2,4-triazolium, and two types of 1,2,3-triazolium cations (Figure 1). We excluded alkyl quaternary ammonium salts with high conformational flexibility from the current study to make the analysis more manageable.

**Figure 2:**
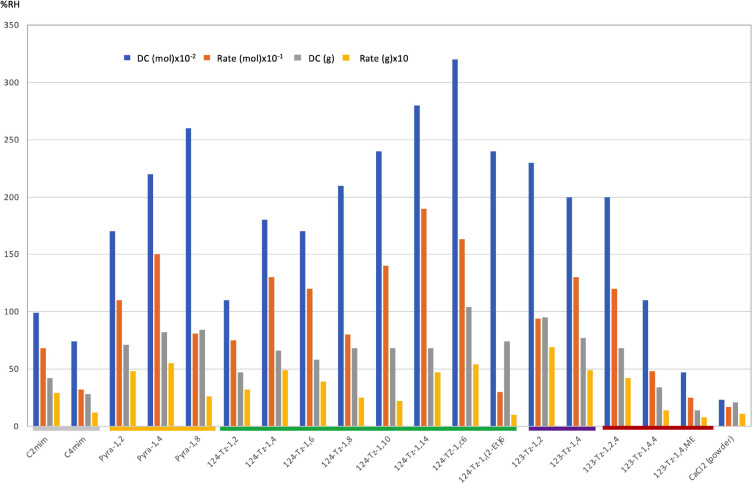
Dehumidification capability (DC) of various ILs. DC (mol) = %RH, in mol^–1^; rate (mol) = %RH, in min^–1^ mol^–1^; DC (g) = %RH, in g^–1^; rate (g) = %RH, in min^–1^ g^–1^.

**Figure 3:**
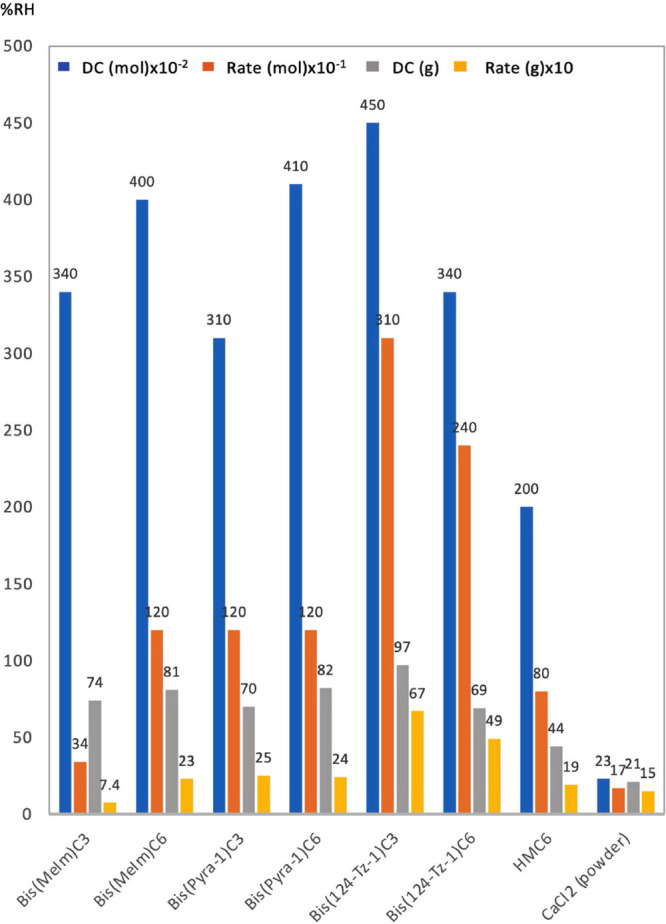
Dehumidification capability (DC) of dicationic ILs. DC (mol) = %RH, in mol^–1^; rate (mol) = %RH, in min^–1^ mol^–1^; DC (g) = %RH, in g^–1^; rate (g) = %RH, in min^–1^ g^–1^.

We systematically synthesized 18 types of dimethyl phosphate ILs, consisting of imidazolium, pyrazolium, or triazolium salts: 1-ethyl-3-methylimidazolium (C_2_mim), 1-butyl-3-methylimidazolium (C_4_mim), 1-ethyl-2-methylpyrazolium (Pyra-1,2), 1-butyl-2-methylpyrazolium (Pyra-1,4), 1-methyl-2-octylpyrazolium (Pyra-1,8), 1-ethyl-4-methyl-1,2,4-triazolium (124-Tz-1,2),^67^ 1-butyl-4-methyl-1,2,4-triazolium (124-Tz-1,4),^67,68^ 1-octyl-4-methyl-1,2,4-triazolium (124-Tz-1,8), 1-decyl-4-methyl-1,2,4-triazolium (124-Tz-1,10), 1-tetradecyl-4-methyl-1,2,4-triazolium (124-Tz-1,14), 1-cyclohexylmethyl-4-methyl-1,2,4-triazolium (124-Tz-1,c6), 1-(2-ethylhexyl)-4-methyl-1,2,4-triazolium (124-Tz-1,(2-Et)6), 1-ethyl-3-methyl-1,2,3-triazolium (123-Tz-1,2), 1-butyl-3-methyl-1,2,3-triazolium (123-Tz-1,4), 1-ethyl-3-methyl-4-butyl-1,2,3-triazolium (123-Tz-1,2,4), 1-butyl-3-methyl-4-butyl-1,2,3-triazolium (123-Tz-1,4,4), 1-(2-methoxyethyl)-3-methyl-4-butyl-1,2,3-triazolium (123-Tz-1,4,ME), and 1-octyl-3-methyl-4-butyl-1,2,3-triazolium (123-TZ-1,4,8). We also prepared dicationic-type imidazolium, pyrazolium, and 1,2,4-triazolium bis(dimethyl phosphate) salts: 1,1′-(propane-1,3-diyl)bis(3-methylimidazolium) (bis(MeIm)C3), 1,1′-(hexane-1,6-diyl)bis(3-methylimidazolium) (bis(MeIm)C6), 1,1′-(propane-1,3-diyl)bis(2-methylpyrazol-2-ium) (bis(pyra-1)C3), 1,1′-(hexane-1,6-diyl)bis(2-methylpyrazol-2-ium) (bis(pyra-1)C6), 1,1′-(propane-1,3-diyl)bis(4-methyl-1,2,4-triazol-1,4-ium) (bis(124-Tz-1)C3), and 1,1′-(hexane-1,3-diyl)bis(4-methyl-1,2,4-triazol-1,4-ium) (bis(124-Tz-1)C6). The results for the dehumidification capabilities (%RH, in mol^–l^, or %RH, in g^–1^) and rates (%RH, in min^–1^ mol^–1^, or %RH, in min^–1^ g^–1^) of these salts are shown in Figures 2 and 3. The results are scaled by 1/100 for the DC (per mol), by 1/10 for the rate (per mol), and by 10 for rate (per gram) for a clear exposition. The results for the popular desiccant CaCl_2_ powder, as well as for [HMC6][DMPO_4_],^38^ which we previously investigated, are presented as the control. For detailed results of the DC and RH, see Table S1 in the Supporting Information.

Ficke and Brennecke previously found that the DC of ILs consisting of small nonprotic imidazolium cations decreases as the cation alkyl chain length increases.^53^ We also observed the same trend for the quaternary ammonium salts.^38^ Hereafter, this DC trend will be referred to as the Brennecke rule. In Figure 2, we notice that the imidazolium and 1,2,3-triazolium ILs follow this rule. For instance, the DC decreases in the order ethyl (123-Tz-1,2,4) > butyl (123-Tz-1,4,4) > 2-methoxyethyl (123-Tz-1,4,ME). In contrast, the pyrazolium salts exhibit the opposite behavior; their DC increases with an increasing alkyl chain length, and Pyra-1,8 displays the highest DC among the three pyrazolium ILs that we studied. This opposite trend in the DC, referred to as the anti-Brennecke rule, is also conspicuous for the 1,2,4-triazolium ILs, with the exception of 124-Tz-1,6. The highest DC and the second highest DC were attained with the cyclohexylmethyl-substituted (124-Tz-1,c6) and the tetradecyl-substituted (124-Tz-1,14) 1,2,4-triazolium ILs; their respective DC (mol) values are 14- and 11-fold higher than that of CaCl_2_. Such high DC values were obtained despite the fact that these ILs have a highly lipophilic substituent. For perspective, we note that it was repeatedly observed that longer side chains generally decrease the water absorption capacity.^20,53^ As for the dehumidification rate, the 124-Tz-1,14 exhibits the fastest absorption, with the rate (mol) value 11 times higher than that of CaCl_2_ (Figure 2).

The results for the dicationic ILs, bis(MeIm)C3, bis(MeIm)C6, bis(Pyra-1)C3, bis(Pyra-1)C6, bis(124-TZ-1)C3, and bis(124-TZ-1)C6, are presented in Figure 3. As expected from our prior study that demonstrated a high DC for the di-QAs,^38^ all these six salts are very hygroscopic and are characterized by extremely high DCs. For example, the DC of bis(124-Tz-1)C3 reaches 450 (4.5 × 10^4^ %RH, mol^–1^), which is 20-fold higher than that of CaCl_2_. Its dehumidification rate is also very high; it is 18 times higher than that of CaCl_2_ and 3.8 times higher than that of HMC6, which exhibits the highest DC among the di-QAs that we previously studied.^38^ To the best of our knowledge, the DC of these dicationic salts is a record among hygroscopic materials. Another interesting result is the effect of the spacer carbon chain length on the DC; while the DC values are in the order bis(MeIm)C6 > bis(MeIm)C3 and bis(Pyra-1)C6 > bis(Pyra-1)C3, they are reversed for the bis-1,2,4-triazolium salts, viz., bis(124-Tz-1)C6 < bis(124-Tz-1)C3. This shows that the dependence of the DC on the alkyl chain length is complex in that the DC depends not only on the alkyl chain spacer group but also on the cationic moiety of the dicationic salt.

### Equilibrium Water Vapor Pressure of Aqueous IL Solutions

The temperature dependence of the equilibrium water vapor pressure, Pv, is another key factor in evaluating desiccant materials for use in LDACs. To help the reader to see this point better, we give a brief description of the operation cycle of LDACs: Starting with the cooling process of the cycle, indoor air comes into contact with a cold and dry liquid desiccant, for example a cold dry IL, where moisture accumulates. The resulting wet IL is warmed to ∼50 °C and brought into contact with the outside air. The outside air, which is normally at a temperature below 50 °C, removes the moisture from the IL through vaporization. The hot dry IL thus produced is then cooled to regenerate the cold dry IL, and the entire cycle is repeated.^11,12,33,35^ Thus, desiccant materials with both a low Pv at low *T* and a high Pv at high *T* are desirable for desiccant materials for LDACs to ensure a smooth moisture transfer.

**Figure 4:**
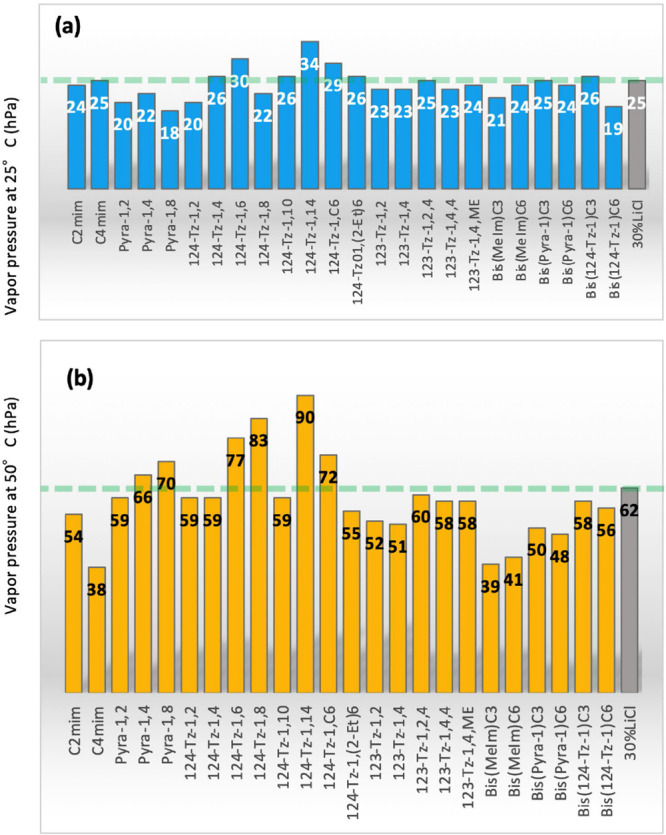
Equilibrium water vapor pressure (Pv) of 80% (w/w) aqueous solutions of ILs at (a) 25 and (b) 50 °C. The Pv of 30% (w/w) LiCl aqueous solution is shown as a green dashed line.

We investigated the temperature-driven changes of the water vapor pressures, Pv, of 80% (w/w) aqueous solutions of the ILs. The results for the Pv at 25 and 50 °C are presented in Figure 4a,b. The results for the 30% (w/w) LiCl aqueous solution, employed as the control, are shown as a green dashed line there. With the exception of 124-Tz-1,6, 124-Tz-1,14, and 124-Tz-1,c6, the Pv values of the ILs are similar to or lower than that of the 30% (w/w) LiCl solution at 25 °C (Figure 4a). This implies that many IL aqueous solutions are more effective in absorbing moisture from air than LiCl aqueous solution.

**Figure 5:**
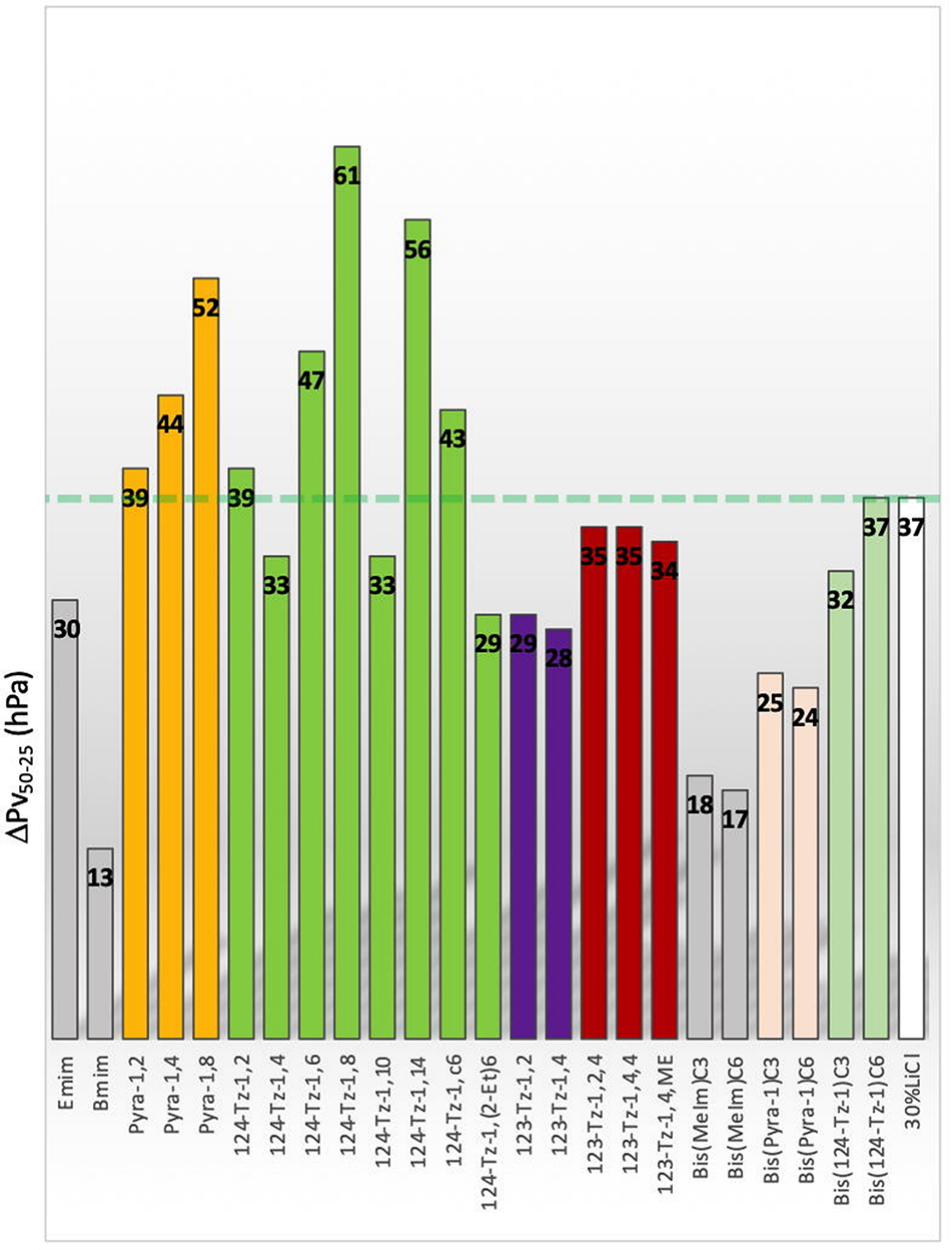
Difference in equilibrium water vapor pressure (ΔPv_50–25_) of 80% (w/w) aqueous solutions of ILs at 50 and 25 °C. ΔPv_50–25_ of 30% (w/w) LiCl aqueous solution is shown as a green dashed line.

The Pv at 50 °C, on the other hand, varies significantly with the IL employed (Figure 4b). The ΔPv_50–25_ results for the tested IL aqueous solutions are displayed in Figure 5, where ΔPv_50–25_ is the difference in the Pv at 50 and 25 °C, i.e., Pv(50 °C) – Pv(25 °C). The ΔPv_50–25_ value is a quantitative measure for the efficiency of the moisture transfer by the IL from the inside to the outside air. Among the ILs that we studied, the ΔPv_50–25_ of three pyrazolium salts and five 1,2,4-triazolium salts exceeds that of the LiCl aqueous solution, revealing a high moisture transfer efficiency of these eight ILs. As such, they provide promising candidates as desiccant sources for LDACs. On the other hand, it is disappointing that aqueous solutions of all the dicationic salts that we considered show a lower ΔPv_50–25_ than the LiCl solution; in other words, the solutions of these salts would not easily release moisture when brought in contact with the outside air. Therefore, while the dicationic salts (bis(MeIm)C3, bis(MeIm)C6, bis(Pyra-1)C3, bis(Pyra-1)C6, and bis(124-TZ-1)C3) can function as strong moisture absorption agents, they may not be suitable as desiccants in LDACs.

### Investigation of the Origin of Moisture Absorption by Small- and Wide-Angle X-ray Scattering (SWAXS) Analysis and MD Simulations

Nanoscale segregations in ILs are well-established, thanks to numerous X-ray diffraction^54,69−80^ and MD simulation^54,70−73^ studies. Specifically, many ILs and their aqueous solutions form complex structures, such as micelles,^75^ lamellar morphology forms,^78^ and bicontinuous microemulsions (alkyl group channel forms).^70−74^ Since small- and wide-angle X-ray scattering (SWAXS) is a powerful tool to probe the nanosized aggregate structures of surfactant molecules and ionic liquids,^73−85^ we have conducted SWAXS analysis of aqueous solutions of 1,2,4-triazolium ILs. The objective was to obtain detailed information on the structures of these ILs, especially the influence of water, in order to gain insight into the roles played by cations in Pv and its *T* dependence. The reason for choosing 1,2,4-triazolium ILs is twofold: First, they show high dehumidification capabilities. Interestingly, they generally follow the anti-Brennecke rule (Figure 2). Second, many of them are characterized by high ΔPv_50–25_, as already mentioned (Figure 5).

**Figure 6:**
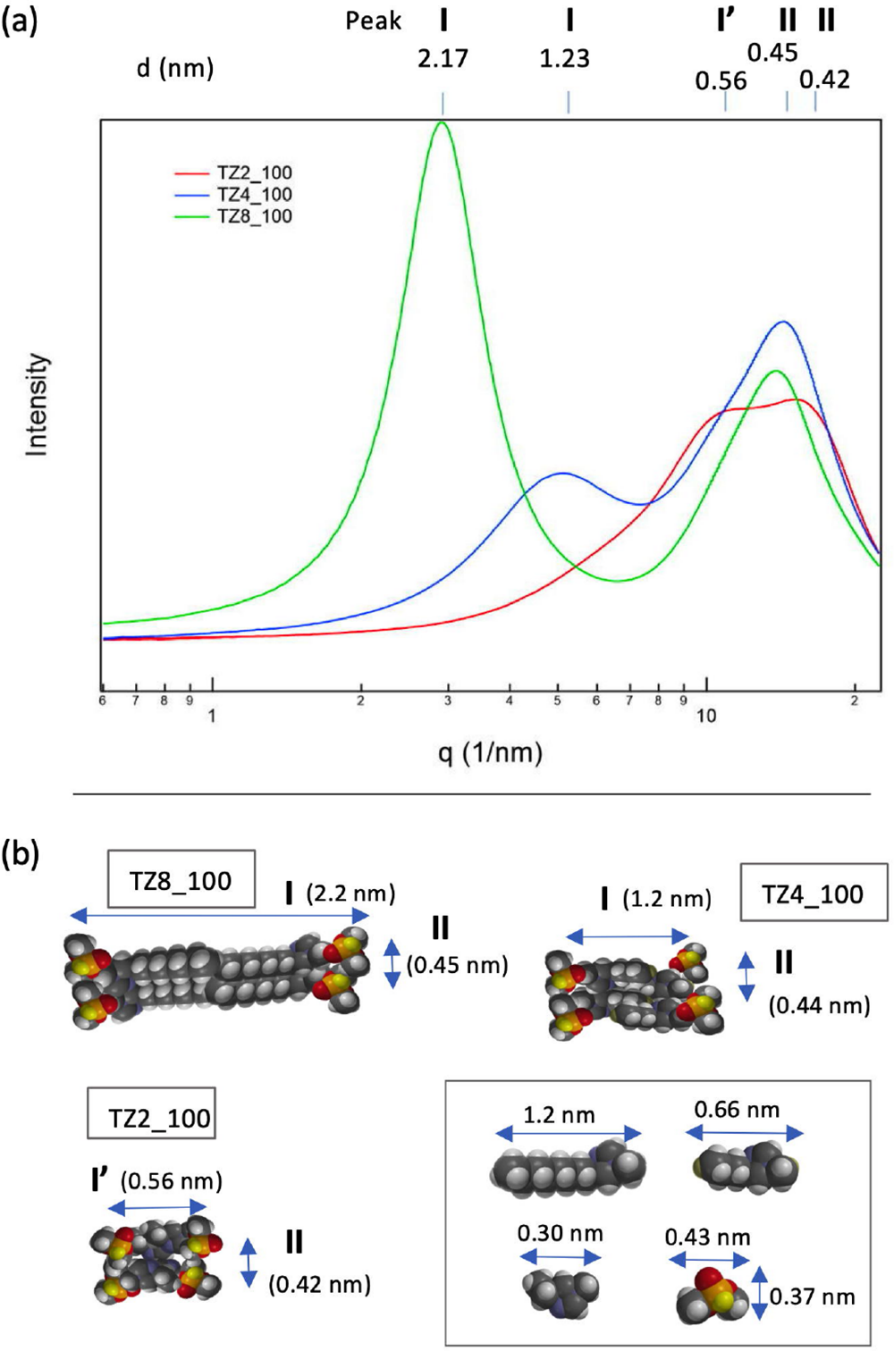
(a) SWAXS analysis of the three types of pure 1,2,4-triazolium dimethyl phosphate ILs: TZ8_100, TZ4_100, and TZ2_100. Here, *q* = || = 4π sin θ/λ, and *d* = λ/2 sin θ = 2π/*q*. (b) MM estimates of the size of IL ions and their ion pair dimeric structures.

We first considered the structural influence of the alkyl chain length of cations in the pure ILs. The SWAXS results for three pure 1,2,4-triazolium dimethyl phosphate ILs, namely, 124-Tz-1,2, 124-Tz-1,4, and 124-Tz-1,8, are compared in Figure 6a. For simplicity, these ILs will be hereafter denoted, respectively, as TZ2, TZ4, and TZ8 (and similarly for the other 1,2,4-triazolium ILs). Furthermore, the percentage (w/w) concentration of the IL aqueous solutions, when needed, will be added at the end of each IL symbol; for example, the 100% (w/w) aqueous solution of 124-Tz-1,8 will be represented as TZ8_100.

The SWAXS spectra in Figure 6a show two distinct peaks (I and II) for both TZ4_100 and TZ8_100. The two peaks of TZ8_100 at *q* = 2.88 and 14.0 nm^–1^ correspond to 2.17 and 0.45 nm in length, respectively, while the corresponding lengths for TZ4_100 are 1.2 (*q* = 5.06 nm^–1^) and 0.45 nm (*q* = 14.3 nm^–1^). By contrast, the TZ2_100 spectrum does not show peak I; rather, it is characterized by a broad band of a double-peak nature with peaks I’ (*q* = 11.2 nm^–1^) and II (*q* = 15 nm^–1^), corresponding to 0.56 and 0.42 nm in length. Peak I, often referred to as the prepeak, is the key signature of the formation of heterogeneous nanostructures.^54,71^ Its absence in TZ2_100 means that nanoscale aggregates are not formed in this IL (see below).

It is well-known that SWAXS signals indicate the most electron-rich atoms in their aggregation structure.^79^ With this in mind, we considered schematic models of ion pair dimeric structures (IPDSs) based on MM calculations,^86^ as illustrated in Figure 6b to gain insight into the SWAXS results at least at the semiquantitative level. The interactions of nonpolar alkyl chains of the cations are responsible for the formation of the IPDSs. Comparison of the SWAXS and MM results suggests the following assignments: peak I (and I’) to the size or length of IPDSs and peak II to the separation of two IPDSs. Specifically, the size and separation of the IPDSs are, respectively, 2.18 and 0.45 nm for TZ8_100, 1.24 and 0.44 nm for TZ4_100, and 0.56 and 0.42 nm for TZ2_100. Therefore, the size of the IPDSs in TZ2_100 is on the subnanometer scale, implying that nanosized structures are not formed. For comparison, MM calculations estimate the lengths of single TZ8, TZ4, and TZ2 cations as 1.2, 0.66, and 0.30 nm, respectively, while the size of the dimethyl phosphate anion is estimated to be 0.43 × 0.37 nm (Figure 6b). The assignments of the peaks based on this simple MM description are generally corroborated by the MD results (see details below).

**Figure 7:**
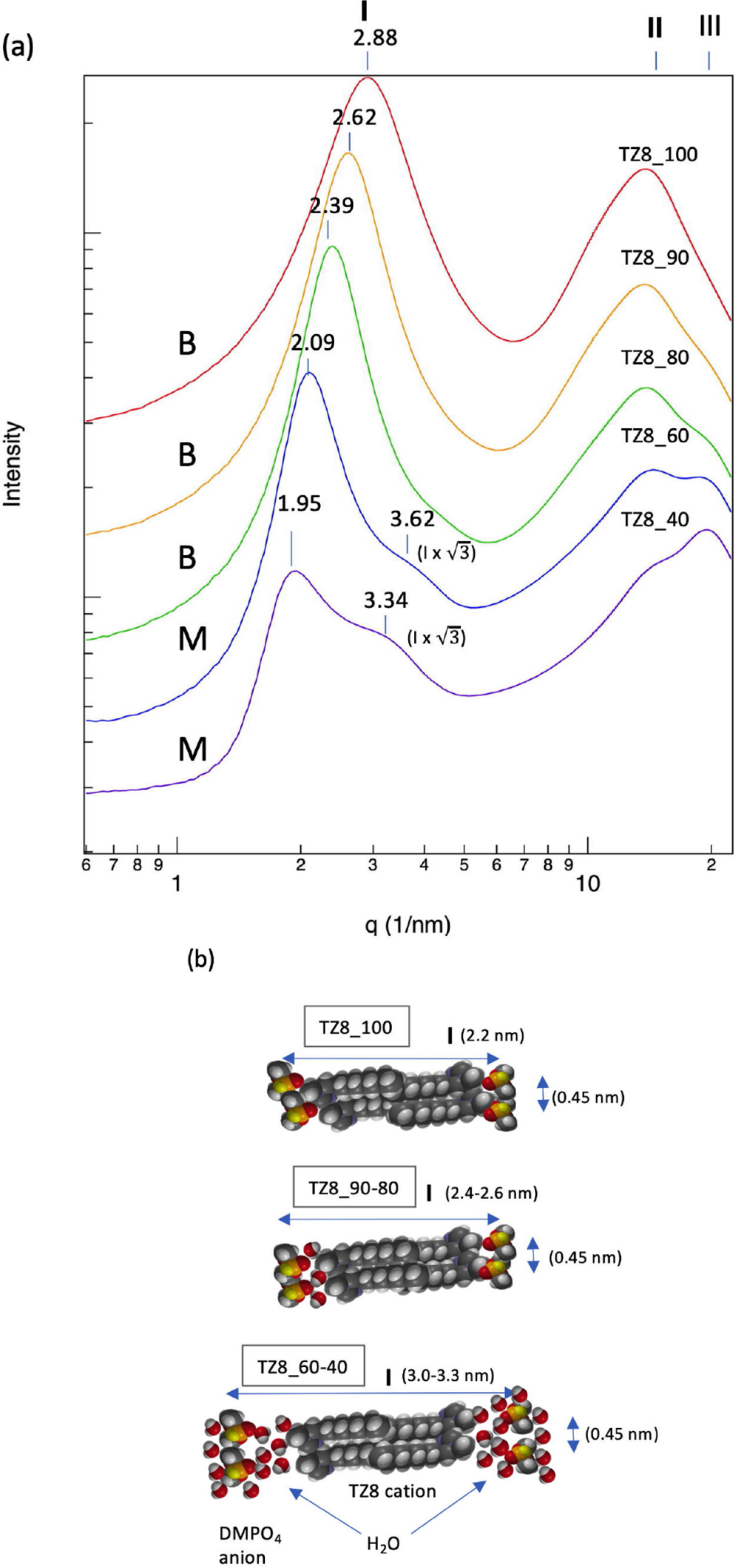
(a) Results of the SWAXS analysis of TZ8 (124-Tz-1,8). TZ8_100 denotes the pure IL (χIL = 1.0), TZ8_90 denotes a 90% (w/w) aqueous solution (χIL = 0.34), TZ8_80 denotes an 80% (w/w) aqueous solution (χIL = 0.18), TZ8_60 denotes a 60% (w/w) aqueous solution (χIL = 0.078), and TZ8_40 denotes a 40% (w/w) aqueous solution (χIL = 0.036). **B** and **M** represent the bicontinuous microemulsion and micelle-like structures, respectively. As in Figure 6, *q* = || = 4π sin θ/λ, and *d* = λ/2 sin θ= 2π/*q*. (b) Illustration of the shift of the peak I position with water concentration in the SWAXS spectra of TZ8 solutions, based on MM estimates of the size of the ion pair dimeric structures.

SWAXS results for the aqueous solutions of TZ8 are exhibited in Figure 7a (see Table S2 in the Supporting Information for further details). One of the most pronounced features is that as the water content increases, peak I shifts to low *q* values from *q* = 2.88 nm^–1^ (*d* = 2.18 nm) for the pure TZ8 (χIL = 1.0) to *q* = 1.95 nm^–1^ (*d* = 3.22 nm) for TZ8_40 (χIL = 0.036). This suggests that the IPDSs in TZ8 expand by ∼0.23 nm per 10% (w/w) decrease in the IL concentration, i.e., an increase in the water content (cf. Figure 7b). In terms of the mole concentration, the water mole fraction increases by 24% per 10% (w/w) decrease. In contrast to peak I, no significant *q* value change was observed for peak II.

Another salient aspect of the SWAXS results is that TZ8_60 and TZ8_40 show a distinct peak (peak III) around *q* = 19.5 nm^–1^ (*d* = 0.32 nm), which becomes weaker as the water concentration decreases. Though it completely disappears in TZ8_100, it persists even in the 90% (w/w) solution as a very minor shoulder structure, revealing that it originates from the water. It is notable that the SWAXS spectrum of pure water shows a pronounced peak at essentially the same location (see Figure S3 in the Supporting Information). In addition to peaks I–III, we notice a small but discernible structure at *q* = 4.14, 3.62, and 3.34 nm^–1^ in the SWAXS spectra of the 80, 60, and 40% (w/w) solutions, respectively. This spectral feature is completely absent in both the pure IL and the 90% (w/w) aqueous solution. Interestingly, the location (i.e., *q* value) of this spectral structure is  times that of peak I. While this relation of the SWAXS signal locations (i.e.,  times the peak I position) suggests the formation of hexagonal cylinder structures,^75,76^ micelle-like structures,^75,78^ or permeabilized lipid membrane-type structures,^81−85^ this is attributed to the formation of micelle-like structures in the TZ8 aqueous solutions (see below). Overall, the SWAXS results in Figure 7a clearly indicate that the three-dimensional structures of the TZ8 vary with the water concentration.

**Figure 8:**
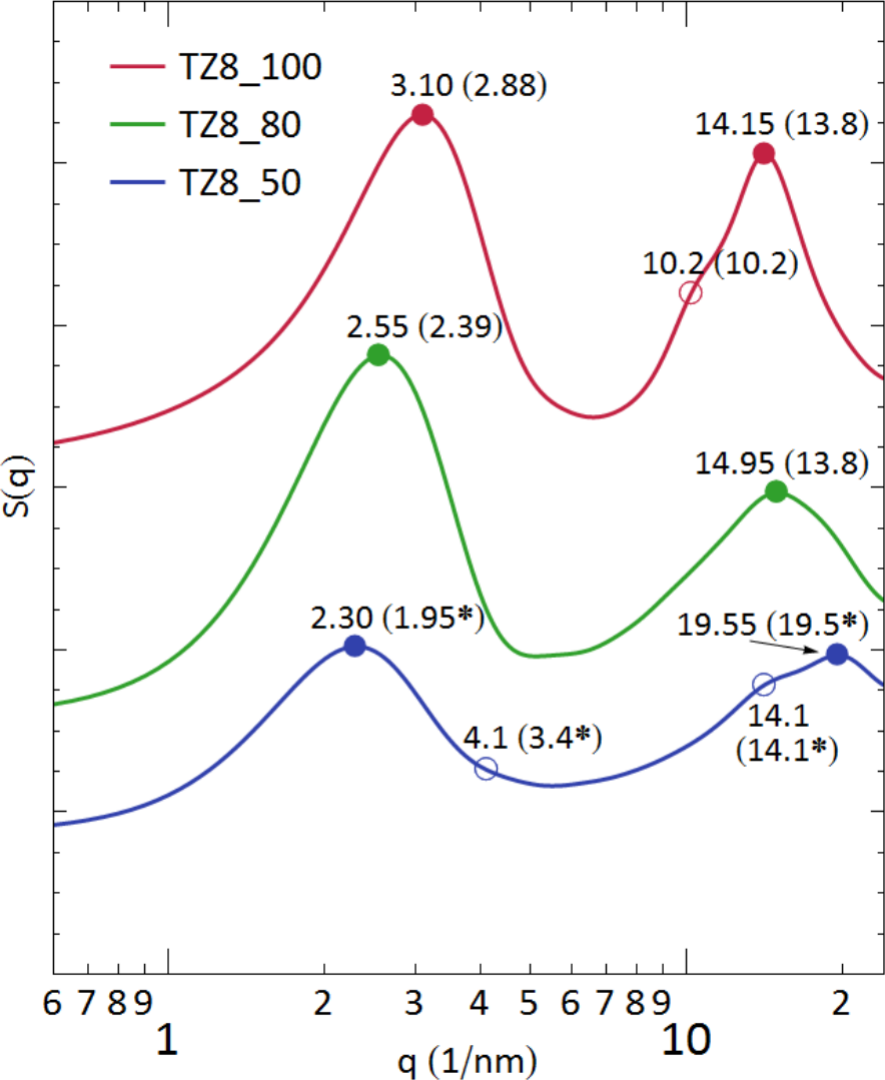
MD simulation results for the X-ray structure factors of TZ8. For comparison, the SWAXS results for the peak positions (Figure 7a) are given in parentheses. Values with an asterisk (*) are the corresponding SWAXS results for TZ8_40.

To obtain a quantitative understanding at the molecular level, we have performed MD simulations of the TZ8_100, TZ8_80, and TZ8_50 systems at *T* = 350 K. Results for the X-ray structure factor *S*(*q*), calculated by^87−89^
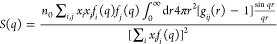
1using the Lorch window function for smoothing,^89,90^ are displayed in Figure 8. In eq 1, *i* and *j* label the atomic species, *g*_*ij*_(*r*) is the pair correlation function (radial distribution function) of *i* and *j*, *x*_*i*_ and *f*_*i*_(*q*) are the mole fraction and atomic form factor of *i*, and *n*_0_ is the total atom number density. Following ref (87), we used the *f*_*i*_(*q*) results compiled in ref (91) in the calculations of *S*(*q*). Though not presented here, we also analyzed the partial structure factors at various levels, viz., contributions to *S*(*q*) from different components—atoms, polar/nonpolar groups, and ions/molecules—of the IL solutions (see Figures S12–S14 for the partial structure factors arising from polar/nonpolar groups). The MD spectra in Figure 8 are in excellent agreement with the SWAXS results in Figure 7a; essentially, all key spectral features are well-captured by the simulations. For example, peak I shifts to lower *q* with an increasing water concentration, whereas the position of peak II remains nearly unchanged. Moreover, a new structure (peak III) appears at *q* ≈ 20 nm^–1^ as water is added to the pure TZ8. While peak III is quite prominent for TZ8_50, it is realized as a very minor shoulder structure in the MD spectrum of TZ8_80. Finally, an extremely weak but noticeable structure appears around *q* = 4.1 nm^–1^ in TZ8_50, consonant with the measurements at a high water concentration.

According to the analysis of the partial structure factors, the primary contribution to peak I arises from the anion–anion pair distribution (mainly P–O and O–O distributions) for TZ8_100, anion–water and water–water distributions for TZ8_80, and water–water and cation tail–tail distributions for TZ8_50 (Figures S12–S14). Results in Figures S12 and S13 indicate that contributions of the cation head and tail groups to the intensity of peak I are canceled to a large degree in TZ8_100 and TZ8_80. As a result, cations contribute little to peak I in these two IL solutions. Here and hereafter, the head and tail of cations refer, respectively, to their polar (i.e., ring atoms and CH_2_ and CH_3_ groups directly bonded to the ring atoms) and nonpolar (i.e., alkyl group atoms, except for the CH_2_ and CH_3_ groups directly bonded to the ring atoms) groups. The shift of the peak I location with an increasing water content is evidence of the expansion of the polar domains due to accumulation of water there; qualitatively, the average distance between the polar regions at the opposite ends of the IPDSs increases because of the increasing water content (Figure 7b).

For peak II, the main contributors are primarily the pair distributions of the same polarity groups of ions, specifically the head–head, tail–tail, and anion−head distributions for TZ8_100 and TZ8_80 and head–head, tail–tail, and anion–anion distributions for TZ8_50 (Figures S12–S14). For instance, the head–head and tail–tail distributions account for more than 50% of the intensity of peak II for TZ8_100. These MD results generally confirm our peak assignments above based on the MM calculations; namely, the length scale of peak II (*d* ≈ 0.45 nm) represents the average distance of two neighboring IPDSs. Peak III near *q* = 19.5 nm^–1^ (*d* = 0.32 nm), on the other hand, is due to pair distributions involving water, as already mentioned (MM estimation of the size of a water molecule is 0.15–0.20 nm). In TZ8_50, the water–water distribution accounts for nearly 50% of its peak III intensity. As for its very minor spectral structure around *q* = 4.1 nm^–1^, the water–water, anion–anion, and cation tail–tail distributions are mainly responsible.

**Figure 9:**
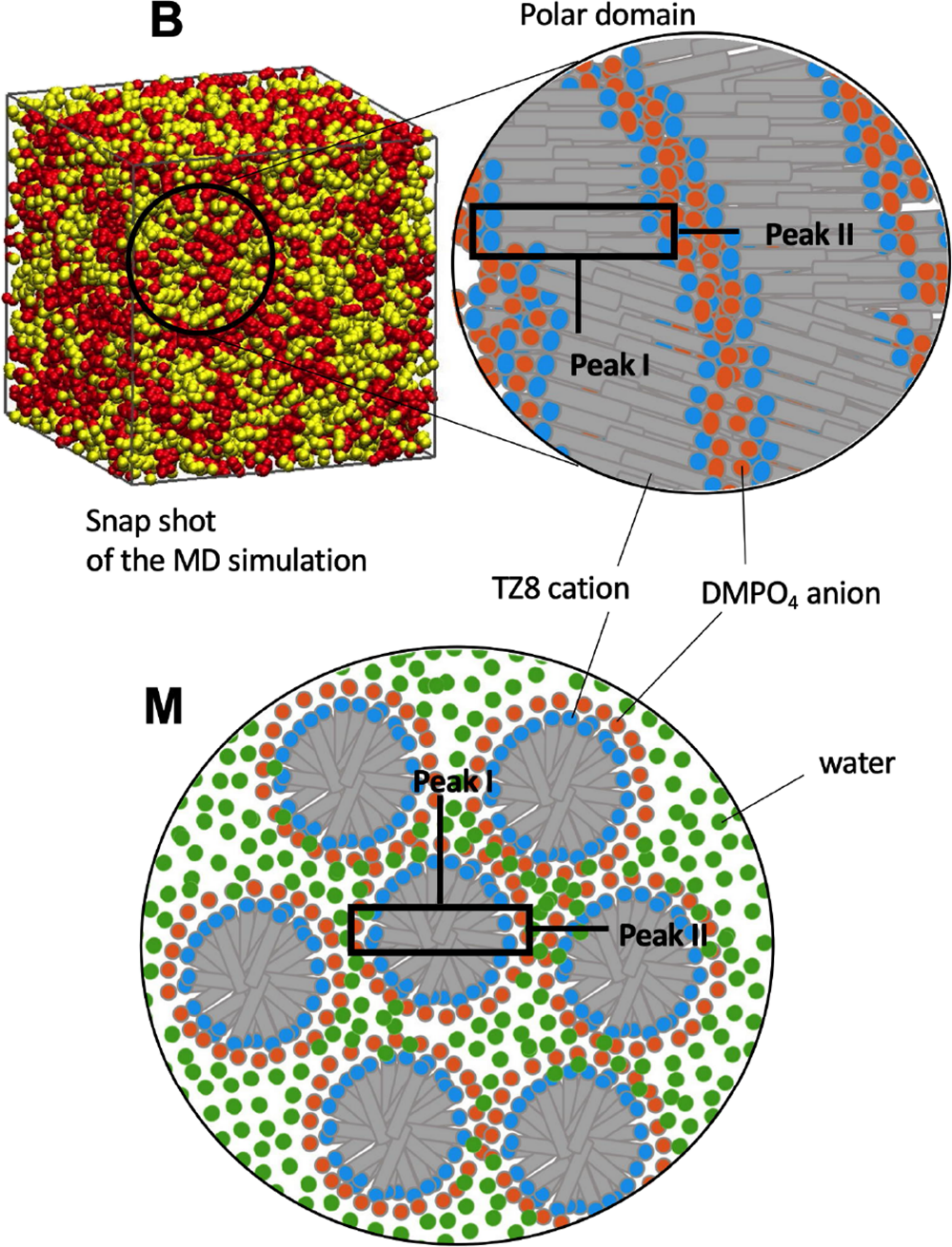
Aggregation structure of TZ8 (124-Tz-1,8). **B** and **M** represent, respectively, the bicontinuous microemulsion and micelle-like structures. We postulate the existence of the **B** form based on our MD simulations. The polar and nonpolar groups are shown in red and yellow, respectively.

**Figure 10:**
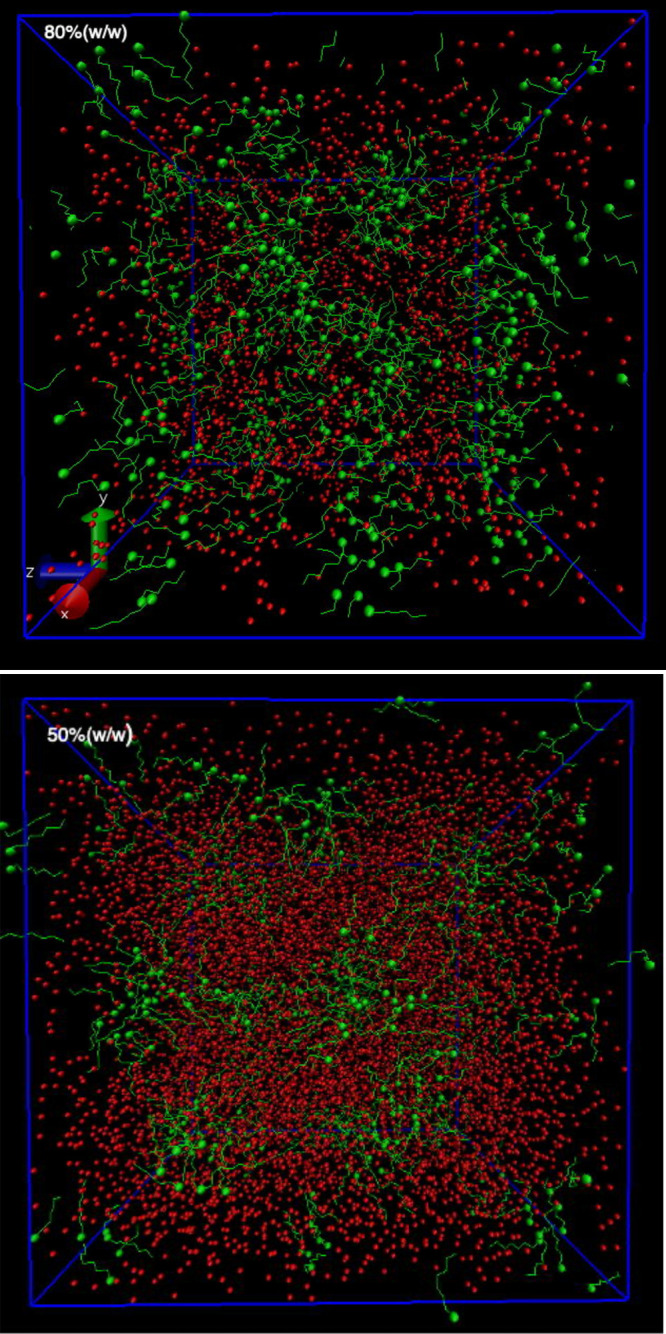
MD simulation snapshots of 80 and 50% (w/w) aqueous solutions of TZ8 at *T* = 350 K. The nonpolar tail of the TZ8 cations is shown in green, with the terminal carbon atom of each cation tail represented as a green sphere. The oxygen atoms of water and of the dimethyl phosphate anions are shown in red.

To illustrate the three-dimensional structures of TZ8, a representative molecular configuration of TZ8_100 obtained from MD is displayed in Figure 9 as **B**, while those of TZ8_80 and TZ8_50 are shown in Figure 10 (see also Figure S2 in the Supporting Information). Analogous to many imidazolium ILs studied already,^70−74^ the results clearly demonstrate the aggregation of the IPDSs to form nanostructures. Furthermore, the characteristics of these structures vary with the water content, viz., a bicontinuous microemulsion in TZ8_100 and TZ8_80 and micelle-like structures in TZ8_50. The tails (i.e., alkyl substituents) of the cations aggregate into nonpolar nanostructures, and the anions (anions and water in the case of IL aqueous solutions) fill in the space between these nonpolar structures and form polar domains together with the polar head groups of cations. In TZ8_80 (Figure 10), water is mainly located in the space spanned by anions (see also Figure S2); this is attributed to strong hydrogen-bonded interactions between the two.^92^ Although these nanostructures are flexible and fluctuate with time, the aggregation of the nonpolar tails of the cations is likely to prevent the total decomposition of the cages of the polar domains. Therefore, the stable nature of the aggregated form **B**, combined with the strong water–anion hydrogen-bonded interaction, is likely the driving force of the moisture absorption by TZ8 and other ILs. In this context, the polar domains shown in red in Figure 10 act as the so-called “water pocket” proposed by Abe et al.^93−96^

Based on the above SWAXS and MD results, we conclude that the TZ8 aqueous solutions can afford two different nanostructures: a bicontinuous microemulsion (**B**) at relatively low water concentrations and micelle-like structures (**M**) at high water concentrations, as indicated in Figure 7a. The appearance of a spectral structure at the *q* value  times the peak I position signifies the transition from a bicontinuous microemulsion to micelle-like structures as the water content increases.

**Figure 11:**
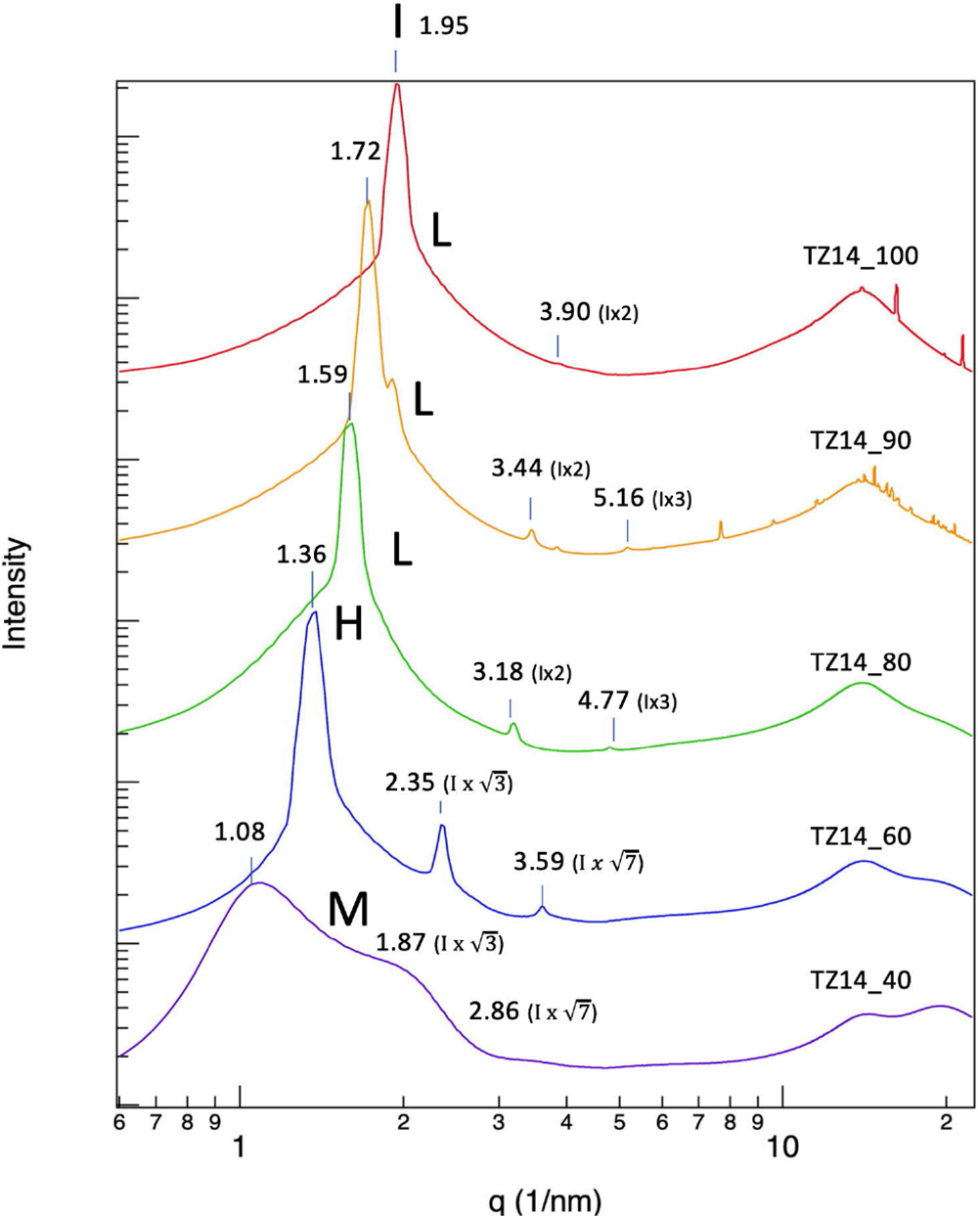
Results of the SWAXS analysis of TZ14 (124-Tz-1,14). TZ14_100 denotes the pure IL (χIL = 1.0), TZ14_90 denotes the 90% (w/w) aqueous solution (χIL = 0.29), TZ14_80 denotes the 80% (w/w) aqueous solution (χIL = 0.15), TZ14_60 denotes the 60% (w/w) aqueous solution (χIL = 0.06), and TZ14_40 denotes the 40% (w/w) aqueous solution (χIL = 0.03). **L**, **H**, and **M** stand for the lamellar form, hexagonal cylinder form, and micelle-like structures, respectively. *q* = |*q⃗*| = 4π sin θ/λ, and *d* = λ/2 sin θ = 2π/*q*.

The SWAXS results for the aqueous solutions of 124-Tz-1,14 (TZ14), which displays an extremely high DC (Figure 2) despite its very hydrophobic tetradecyl substituent on the triazolium cation, are shown in Figure 11. In contrast to the bicontinuous microemulsion (**B**) in TZ8 in Figure 7, a lamellar structure (**L**) is formed in pure TZ14 (TZ14_100, χIL = 1.0) as well as in the 90% (w/w) (TZ14_90, χIL = 0.29) and 80% (w/w) (TZ14_80, χIL = 0.15) solutions, as evidenced by the appearance of sharp peaks at *q* values that are multiples of the peak I position. Specifically, TZ14_100 shows a second peak, albeit low in intensity, at *q* = 3.90 nm^–1^ (*d* = 2.03 nm), which is twice the *q* value of peak I, 1.95 nm^–1^. The 90 and 80% solutions exhibit the second and third peaks located at *q* = 3.44 and 5.16 nm^–1^ (TZ14_90) and *q* = 3.18 and 4.77 nm^–1^ (TZ14_80), which are 2 and 3 times their respective peak I positions. It is well known that this pattern of peak positions in the SWAXS spectra is characteristic of a lamellar form of surfactant compounds.^97^ In TZ14_60, the positions of the second and third peaks shift to *q* = 2.35 and 3.59 nm^–1^, corresponding to  and  times its peak I location of 1.36 nm^–1^. The relative peak positions of 1:: strongly suggest the formation of hexagonal cylinder structures (**H**), even though no clearly noticeable peak was found at the relative position of 2. (However, it does appear that a very minor shoulder structure is present around the relative position of 2, i.e., *q* = 2.7 nm^–1^.) In the 40% (w/w) aqueous solution, the peaks at the relative positions  and  become much weaker than those of the 60% (w/w) solution; in fact, the peak at position  essentially disappears, while that at  becomes a shoulder structure. As a result, the overall SWAXS spectrum of TZ14_40 becomes very similar to that of TZ8_40 in Figure 7a. As in the latter solution, we interpret this as the transformation to micelle-like structures in TZ14_40. In summary, aqueous solutions of TZ14 can form three different structures: a lamellar structure (**L**) at low water concentrations as well as in pure IL, hexagonal cylinder forms (**H**) at moderate water concentrations, and micelle-like structures (**M**) at high water concentrations. It is worth mentioning that Yada et al. observed similar water-induced structural changes for the surfactant hexaoxyethylene dodecyl methyl ether (C_12_EO_8_OMe), by a combined analysis of SAXS and cryo-TEM;^97^ the hexagonal cylinder structures of the surfactant change to micelle-like structures as the water content of the solution increases. Structural transformations of TZ14 induced by water are in contrast to those of TZ8, characterized by two forms, **B** and **M**. This is ascribed to the difference in the stability of their IPDSs and thus of their nanostructures. Since the longer alkyl chain of TZ14 makes their IPDSs more stable, they tend to form more stable, and likely more ordered, nanostructures than TZ8 at a given water concentration. We believe that this is also responsible for the difference in their water absorption capability. The degree of structural change with water absorption and the accompanying energy cost, i.e., the increase in enthalpy, will be lower in TZ14 than in TZ8. Assuming that the entropic effect is small compared to enthalpy, this would make TZ14 more water absorbent than TZ8.

**Figure 12:**
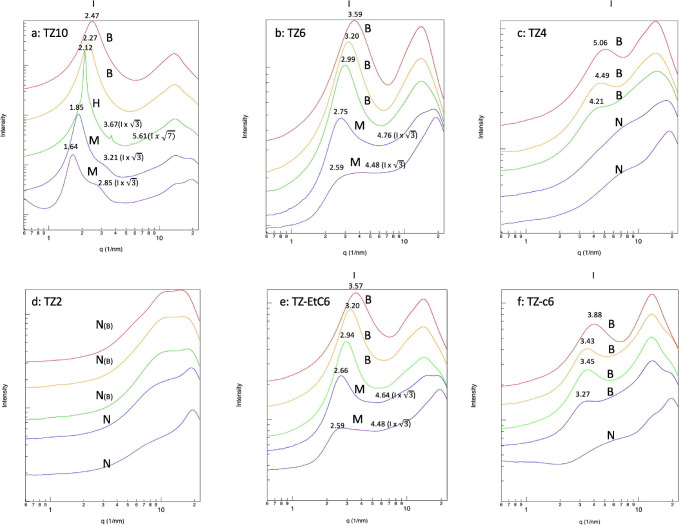
Results of the SWAXS analysis of six types of 1,2,4-triazolium ILs: (a) TZ10 (124-Tz-1,10), (b) TZ6 (124-Tz-1,6), (c) TZ4 (124-Tz-1,4), (d) TZ2 (124-Tz-1,2), (e) TZ-EtC6 (124-Tz-1,(2-Et)6), and (f) TZ-c6 (124-Tz-1,c6). The red line denotes pure ILs, the yellow line denotes 90% (w/w) aqueous solutions, the green line denotes 80% (w/w) aqueous solutions, the blue line denotes 60% (w/w) aqueous solutions, and the purple line denotes 40% (w/w) aqueous solutions (*q* = || = 4π sin θ/λ, and *d* = λ/2 sin θ = 2π/*q*). **B**, **H**, and **M** represent, respectively, the bicontinuous microemulsion, hexagonal cylinder form, and micelle-like structures, while **N(B)** and **N** denote that no aggregation occurs in the solution and that the structure is approximately uniform without any nanostructure.

The SWAXS results of the remaining six types of 1,2,4-triazolium ILs, along with assignments of their aggregation structures, are presented in Figure 12a–f. Briefly, TZ-EtC6 (124-Tz-1,(2-Et)6) and TZ6 (124-Tz,-1,6) can form two nanostructures (**B** and **M**) like TZ8, whereas TZ10 (124-Tz-1,10) affords three different forms: **B**, **H**, and **M**. TZ4 (124-Tz-1,4) and TZ-c6 (124-TZ-1,c6), on the other hand, are characterized by either a bicontinuous microemulsion (**B**) or no aggregation (**N**), depending on the water concentration (Figure 12c,f). By contrast, due to the short alkyl chain length of its cations, TZ2 does not aggregate into heterogeneous nanostructures under all conditions (Figure 12d) as indicated above. We assign **N**(**B**) to TZ2_100, TZ2_90, and TZ2_80 because the size of the subnanoscale particles, e.g., IPDSs, can be estimated from their spectra (see Figure 6 above).

**Figure 13:**
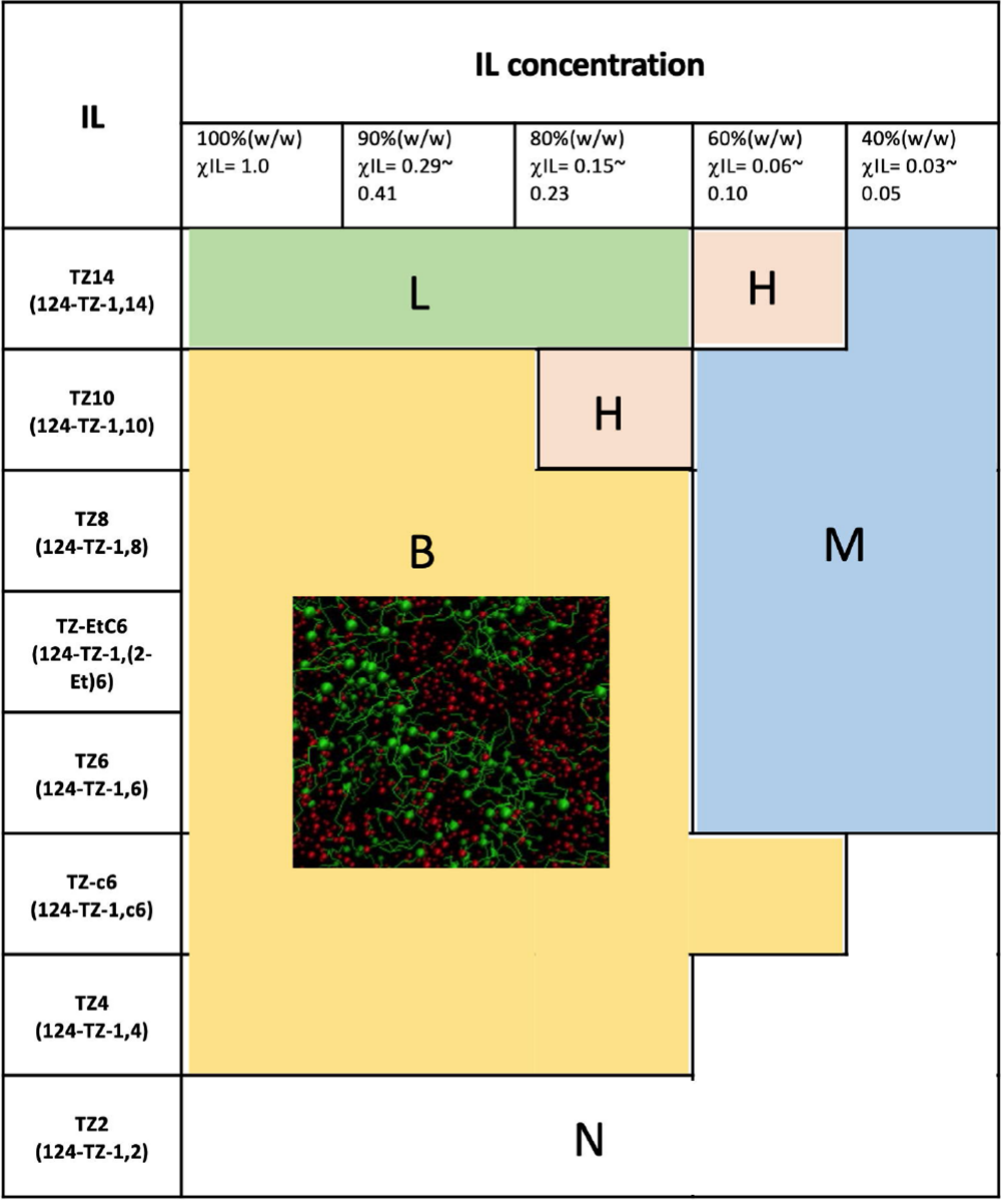
Aggregation form map of eight types of 1,2,4-triazolium IL aqueous solutions. **L** denotes the lamellar form, **H** denotes the hexagonal cylinder form, **B** denotes the bicontinuous microemulsion form, and **M** denotes the micelle-like structure form. **N** denotes that no aggregation form occurs in the solution and that the structure is approximately uniform without any nanostructure.

For convenience, our findings are summarized in Figure 13 as a diagram that maps the 1,2,4-triazolium ILs and their water-dependent aggregation forms. It is interesting that TZ-c6 maintains the **B** structure even at 60% (w/w) and does not show the **B**-to-**M** phase change (Figure 12f). We attribute this to the bulky cyclohexyl substituent of the cation, whose flexible and disordered nature allows TZ-c6 to better adapt to increasing water concentrations (without entailing major changes in its aggregation structure) than other 1,2,4-triazolium ILs consisting of cations with linear alkyl chain substituents. Furthermore, the bulky and disordered alkyl chain would likely yield a more porous aggregate form, i.e., larger voids for the formation of water pockets, in TZ-c6, resulting in a significantly better water uptake than the other 1,2,4-triazolium ILs. This explains the superb DC of TZ-c6 (Figure 2).

As for cations with a linear alkyl chain substituent, a comparison with the results in Figure 2 indicates that there is generally a correlation (referred to as “DC−nanostructure correlation”) between the DC and water-induced structural transformations of their IL solutions, though the DC measurements are for the nearly pure ILs. Specifically, DC is higher in ILs whose aqueous solutions tend to form more ordered aggregates (e.g., the DC is higher in **L** than in **B**). In addition, the DC tends to be lower in ILs whose aggregated forms are more fragile, i.e., their nanostructures transform to **N** (or **M**) more easily with an increasing water concentration. As in the case of TZ14 versus TZ8 discussed above, this is attributed to the enhanced stability of the nonpolar domains and thus of the polar domains of the aggregates with an increasing alkyl chain length. This lowers the (free) energy cost for the formation of water pockets and therefore increases the water uptake. In the case of ΔPv_50–25_, a comparison with Figure 5 suggests that the 80% solutions in the **L** or **B** form generally afford higher ΔPv_50–25_ than those in the **H** form. However, a proper understanding of the highly nonmonotonic trend of ΔPv_50–25_ with a cation alkyl chain length would require a further analysis, e.g., an investigation of structural changes in the aggregates with temperature. Despite this, our results and observations in the present study clearly indicate that the introduction of the appropriate alkyl side chain in the cationic moiety is crucial for the design of efficient desiccant materials for LDACs.

## Conclusions

In this paper, we studied the dehumidification capability of 24 types of ILs that were synthesized by combining the dimethyl phosphate anion with various types of alkyl group-substituted cyclic cations: imidazolium, pyrazolium, 1,2,3-triazolium, and 1,2,4-triazolium cations. These ILs exhibit high dehumidification capabilities; the best DC was attained for 1-cyclohexylmethyl-4-methyl-1,2,4-triazolium dimethyl phosphate, which displayed a DC (mol) 14 times higher than that of popular solid desiccants like CaCl_2_ and silica gel. Furthermore, we discovered that the DC (mol) value of the dicationic ILs, such as 1,1′-(propane-1,3-diyl)bis(4-methyl-1,2,4-triazolium) bis(dimethyl phosphate), is 20 times higher than that of CaCl_2_; this DC value is, to our knowledge, the highest among desiccant materials known. The small- and wide-angle X-ray scattering (SWAXS) analysis of eight types of 1,2,4-triazolium dimethyl phosphates indicated that three types of water concentration-dependent nanostructures—the bicontinuous microemulsion, hexagonal cylinder, and micelle-like structures—can be produced in these ILs and their aqueous solutions through aggregation of the ion pairs. A lamellar structure can also be formed for cations with long alkyl side chains. The aggregate forms and their water-dependent structural changes exhibited strong correlations with the DCs of the ILs. This DC−nanostructure correlation is likely due to the fact that water molecules are incorporated into the polar domains in the nanostructure.

MD simulations of the aqueous solutions of 1-methyl-4-octyl-1,2,4-triazolium dimethyl phosphate (124-Tx-1,8) clearly showed that as expected, water molecules accumulate in polar regions of the aggregated nanostructures of the IL. In this context, these polar domains work as water pockets at low water concentrations, as previously proposed.^82−85^ This result, along with the DC–nanostructure correlation, implies that the moisture absorption capability of 1,2,4-triazolium dimethyl phosphate ILs is closely related to the stability of the polar domains of the nanostructure. Additionally, the *T* dependence of the polar domain stability is likely among the most important factors that govern ΔPv_50–25_ of the IL solutions, although this point was not pursued in the present study. It would thus be worthwhile in the future to analyze this via MD simulations. In conclusion, the alkyl side chain of the cation is the key determining factor of the aggregation forms and their stability, which play important roles in moisture absorption. Therefore, the design of suitable cations is crucial for the development of efficient desiccant ILs for LDACs, which have immense potential to reduce energy consumption and thus to contribute to the sustainability of society.

## Supporting Information Available

Experimental details for measurement of the dehumidification capability, equilibrium water vapor pressure, and ^1^H and ^13^C NMR spectra for all products (PDF)
