# Supplementary material for: On the Moisture Absorption Capability of Ionic Liquids
Source: J Phys Chem B. 2024 Jun 14;128(25):6134–50. doi: 10.1021/acs.jpcb.4c02289 (PMC11215776; doi:10.1021/acs.jpcb.4c02289)
Supplement: Supplementary file 1 — jp4c02289_si_001.pdf [file jp4c02289_si_001.pdf]

Supporting Information

On the Moisture Absorption Capability of Ionic Liquids

Toshiyuki Itoh,[a,*] Kentaro Kamada,[b] Toshiki Nokami,[b] Taiji Ikawa,[c] Kenichi Yagi,[c] Shuji Ikegami,[d] Ryo Inoue,[d] Andrew D. DeYoung,[e] and Hyung J. Kim[e,*]

[a] Toyota Physical and Chemical Research Institute, 41-1 Yokomichi, Nagakute, Aichi 480-1192, Japan, [b]Department of Chemistry and Biotechnology, Graduate School of Engineering, Tottori University, 4-101 Koyama-minami, Tottori 680-8552, Japan. [c]Toyota Central R&D Labs., Inc., 41-1 Yokomichi, Nagakute, Aichi 480-1192, Japan, [d]Daikin Industries, Ltd., Technology and Innovation Center, 1-1 Nishi-Hitotsuya, Settsu, Osaka 566-8585, Japan. [e]Department of Chemistry, Carnegie Mellon University, 4400 Fifth Avenue, Pittsburgh, PA 15213, USA

E-mail: titoh@toyotariken.jp, hjkim@cmu.edu

Number of pages: 72
Number of figures: 105
Number of tables: 2

Contents		
**Table S1. Dehumidification capability of imidazolium, pyrazolium, 1,2,3-triazolium, and 1,2,4-triazolium dimethyl phosphate ILs.**	---------------------------	S5
**Figure S1. Snapshots of TZ8 ([124-Tz-1,8][$DMPO_4$]) from MD simulations.**	---------------------------	S6
**Figure S2. MD snapshots of 80% (w/w) aqueous solution of TZ8 ([124-Tz-1,8][$DMPO_4$]) at *T* = 350 K.**	---------------------------	S6
**Figure S3. Results of SWAXS analysis of water.**	---------------------------	S7
**Determination of the saturated water vapor pressure**	---------------------------	S7
**Figure S4. The experimental apparatus for measuring the saturated water vapor pressure.**	---------------------------	S7
**Figure S5. Arrhenius plot of *T*-depended saturated water vapor pressure of three types of dicationic quaternary ammonium-bis(dimethyl phosphate) 80% aq. solutions.**	---------------------------	S8
**Figure S6. Arrhenius plot of *T*-depended saturated water vapor pressure change of C_2mim or C_4mim dimethyl phosphate 80% aq. solution.**	---------------------------	S8
**Figure S7. Arrhenius plot of *T*-depended saturated water vapor pressure change.**	---------------------------	S8
**Determination of the dehumidification capability**	---------------------------	S9
**Figure S8. Typical example of determination of dehumidification capability (DC): [HMC6][$DMPO_4$]$_2$.[38]**	---------------------------	S9
**Figure S9-1. The dehumidification capability of [C_2mim][$DMPO_4$].**	---------------------------	S9
**Figure S9-2. The dehumidification capability of [C_4mim][$DMPO_4$].**	---------------------------	S9
**Figure S9-3. The dehumidification capability of [Pyra-1,2][$DMPO_4$].**	---------------------------	S9
**Figure S9-4. The dehumidification capability of [Pyra-1,4][$DMPO_4$].**	---------------------------	S9
**Figure S9-5. The dehumidification capability of [Pyra-1,8][$DMPO_4$].**	---------------------------	S10
**Figure S9-6. The dehumidification capability of [124-Tz-1,2][DMPO4].**	---------------------------	S10
**Figure S9-7. The dehumidification capability of [124-Tz-1,4][$DMPO_4$].**	---------------------------	S10
**Figure S9-8. The dehumidification capability of [124-Tz-1,6][$DMPO_4$].**	---------------------------	S10
**Figure S9-9. The dehumidification capability of [124-Tz-1,8][$DMPO_4$].**	---------------------------	S10
**Figure S9-10. The dehumidification capability of [124-Tz-1,10][$DMPO_4$].**	---------------------------	S10
**Figure S9-11. The dehumidification capability of [124-Tz-1,14][$DMPO_4$].**	---------------------------	S11
**Figure S9-12. The dehumidification capability of [124-Tz-1,c6][$DMPO_4$].**	---------------------------	S11
**Figure S9-13. The dehumidification capability of [124-Tz-1,(2-Et)6][$DMPO_4$].**	---------------------------	S11
**Figure S9-14. The dehumidification capability of [123-Tz-1,2][$DMPO_4$].**	---------------------------	S11
**Figure S9-15. The dehumidification capability of [123-Tz-1,4][$DMPO_4$].**	---------------------------	S11
**Figure S9-16. The dehumidification capability of [123-Tz-1,2,4][$DEPO_4$].**	---------------------------	S11
**Figure S9-17. The dehumidification capability of [123-Tz-1,4,4][$DMPO_4$].**	---------------------------	S12
**Figure S9-18. The dehumidification capability of [123-Tz-1,4,ME][$DEPO_4$].**	---------------------------	S12
**Figure S9-19. The dehumidification capability of [Bis(MeIm)C3][$DMPO_4$]$_2$.**	---------------------------	S12
**Figure S9-20. The dehumidification capability of [Bis(MeIm)C6][$DMPO_4$]$_2$.**	---------------------------	S12
**Figure S9-21. The dehumidification capability of [Bis(Pyra-1)C3][$DMPO_4$]$_2$.**	---------------------------	S12
**Figure S9-22. The dehumidification capability of [Bis(Pyra-12)C6][$DMPO_4$]$_2$.**	---------------------------	S12
**Figure S9-23. The dehumidification capability of [Bis(124-Tz-1)C3][$DMPO_4$]$_2$.**	---------------------------	S13
**Figure S9-24. The dehumidification capability of [Bis(124-Tz-1)C6][$DMPO_4$]$_2$.**	---------------------------	S13
**Figure S9-25. The dehumidification capability of $CaCl_2$ (Powder, Nacalai Tesque, Lot. MOA0090).**	---------------------------	S13
**Figure S10-1. The equilibrium water vapor pressure of 30%(w/w) LiCl aq. solution.**	---------------------------	S14
**Figure S10-2. The equilibrium water vapor pressure of [C_2mim][$DMPO_4$] 80% aq. solution.**	---------------------------	S14
**Figure S10-3. The equilibrium water vapor pressure of [C_4mim][$DMPO_4$] 80% aq. solution.**	---------------------------	S14
**Figure S10-4. The equilibrium water vapor pressure of [Pyra-1,2][$DMPO_4$] 80% aq. solution.**	---------------------------	S14
**Figure S10-5. The equilibrium water vapor pressure of [Pyra-1,4][$DMPO_4$] 80% aq. solution.**	---------------------------	S14
**Figure S10-6. The equilibrium water vapor pressure of [Pyra-1,8][$DMPO_4$] 80% aq. solution.**	---------------------------	S14
**Figure S10-7. The equilibrium water vapor pressure of [124-Tz-1,2][$DMPO_4$] 80% aq. solution.**	---------------------------	S14
**Figure S10-8. The equilibrium water vapor pressure of [124-Tz-1,4][$DMPO_4$] 80% aq. solution.**	---------------------------	S14
**Figure S10-9. The equilibrium water vapor pressure of [124-Tz-1,6][$DMPO_4$] 80% aq. solution.**	---------------------------	S15
**Figure S10-10. The equilibrium water vapor pressure of [124-Tz-1,8][$DMPO_4$] 80% aq. solution.**	---------------------------	S15

**Figure S10-11. The equilibrium water vapor pressure of [124-Tz-1,10][DMPO$_4$] 80% aq. solution.**	---------------------------	S15
**Figure S10-12. The equilibrium water vapor pressure of [124-Tz-1,14][DMPO$_4$] 80% aq. solution.**	---------------------------	S15
**Figure S10-13. The equilibrium water vapor pressure of [124-Tz-1,c6][DMPO$_4$] 80% aq. solution.**	---------------------------	S15
**Figure S10-14. The equilibrium water vapor pressure of [124-Tz-1,(2-Et)6][DMPO$_4$] 80% aq. solution.**	---------------------------	S15
**Figure S10-15. The equilibrium water vapor pressure of [123-Tz-1,2][DMPO$_4$] 80% aq. solution.**	---------------------------	S15
**Figure S10-16. The equilibrium water vapor pressure of [123-Tz-1,4-][DMPO$_4$] 80% aq. solution.**	---------------------------	S15
**Figure S10-17. The equilibrium water vapor pressure of [123-Tz-1,2,4][DMPO$_4$] 80% aq. solution.**	---------------------------	S16
**Figure S10-18. The equilibrium water vapor pressure of [123-Tz-1,4,4][DMPO$_4$] 80% aq. solution.**	---------------------------	S16
**Figure S10-19. The equilibrium water vapor pressure of [123-Tz-1,4,ME][DMPO$_4$] 80% aq. solution.**	---------------------------	S16
**Figure S10-20. The equilibrium water vapor pressure of [Bis(MeIm)C3][DMPO$_4$]$_2$ 80% aq. solution.**	---------------------------	S16
**Figure S10-21. The equilibrium water vapor pressure of [Bis(MeIm)C6][DMPO$_4$]$_2$ 80% aq. solution.**	---------------------------	S16
**Figure S10-22. The equilibrium water vapor pressure of [Bis(Pyra-1)C3][DMPO$_4$]$_2$ 80% aq. solution.**	---------------------------	S16
**Figure S10-23. The equilibrium water vapor pressure of [Bis(Pyra-1)C6][DMPO$_4$]$_2$ 80% aq. solution.**	---------------------------	S16
**Figure S10-24. The equilibrium water vapor pressure of [Bis(124-Tz-1)C3][DMPO$_4$]$_2$ 80% aq. solution.**	---------------------------	S17
**Figure S10-25. The equilibrium water vapor pressure of [Bis(124-Tz-1)C6][DMPO$_4$]$_2$ 80% aq. solution.**	---------------------------	S17
**Figure S11-1. ^{1}H NMR of [Pyra-1,2][DMPO$_4$].**	---------------------------	S18
**Figure S11-2. ^{13}C NMR of [Pyra-1,2][DMPO$_4$].**	---------------------------	S18
**Figure S11-3. ^{1}H NMR of [Pyra-1,4][DMPO$_4$].**	---------------------------	S19
**Figure S11-4. ^{13}C NMR of [Pyra-1,4][DMPO$_4$].**	---------------------------	S19
**Figure S11-5. ^{1}H NMR of [Pyra-1,8][DMPO$_4$].**	---------------------------	S20
**Figure S11-6. ^{13}C NMR of [Pyra-1,8][DMPO$_4$].**	---------------------------	S20
**Figure S11-7. ^{1}H NMR of [124-Tz-1,2][DMPO$_4$].**	---------------------------	S21
**Figure S11-8. ^{13}C NMR of [124-Tz-1,2][DMPO$_4$].**	---------------------------	S21
**Figure S11-9. ^{1}H NMR of [124-Tz-1,4][DMPO$_4$].**	---------------------------	S22
**Figure S11-10. ^{13}C NMR of [124-Tz-1,4][DMPO$_4$].**	---------------------------	S22
**Figure S11-11. ^{1}H NMR of [124-Tz-1,6][DMPO$_4$].**	---------------------------	S23
**Figure S11-12. ^{13}C NMR of [124-Tz-1,6][DMPO$_4$].**	---------------------------	S23
**Figure S11-13. ^{1}H NMR of [124-Tz-1,8][DMPO$_4$].**	---------------------------	S24
**Figure S11-14. ^{13}C NMR of [124-Tz-1,8][DMPO$_4$].**	---------------------------	S24
**Figure S11-15. ^{1}H NMR of [124-Tz-1,10][DMPO$_4$].**	---------------------------	S25
**Figure S11-16. ^{13}C NMR of [124-Tz-1,10][DMPO$_4$].**	---------------------------	S25
**Figure S11-17. ^{1}H NMR of [124-Tz-1,14][DMPO$_4$].**	---------------------------	S26
**Figure S11-18. ^{13}C NMR of [124-Tz-1,14][DMPO$_4$].**	---------------------------	S26
**Figure S11-19. ^{1}H NMR of [124-Tz-1,c6][DMPO$_4$].**	---------------------------	S27
**Figure S11-20. ^{13}C NMR of [124-Tz-1,c6][DMPO$_4$].**	---------------------------	S27
**Figure S11-21. ^{1}H NMR of [124-Tz-1,(2-Et)6][DMPO$_4$].**	---------------------------	S28
**Figure S11-22. ^{13}C NMR of [124-Tz-1,(2-Et)6][DMPO$_4$].**	---------------------------	S28
**Figure S11-23. ^{1}H NMR of [123-Tz-1,2][DMPO$_4$].**	---------------------------	S29
**Figure S11-24. ^{13}C NMR of [123-Tz-1,2][DMPO$_4$].**	---------------------------	S29
**Figure S11-25. ^{1}H NMR of [123-Tz-1,4][DMPO$_4$].**	---------------------------	S30
**Figure S11-26. ^{13}C NMR of [123-Tz-1,4][DMPO$_4$].**	---------------------------	S30
**Figure S11-27. ^{1}H NMR of [123-Tz-1,2,4][DMPO$_4$].**	---------------------------	S31
**Figure S11-28. ^{13}C NMR of [123-Tz-1,2,4][DMPO$_4$].**	---------------------------	S31
**Figure S11-29. ^{1}H NMR of [123-Tz-1,4,4][DMPO$_4$].**	---------------------------	S32
**Figure S11-30. ^{13}C NMR of [123-Tz-1,4,4][DMPO$_4$].**	---------------------------	S32
**Figure S11-31. ^{1}H NMR of [123-Tz-1,4,ME][DMPO$_4$].**	---------------------------	S33
**Figure S11-32. ^{13}C NMR of [123-Tz-1,4,ME][DMPO$_4$].**	---------------------------	S33
**Figure S11-33. ^{1}H NMR of [Bis(MeIm)C3][DMPO$_4$]$_2$.**	---------------------------	S34
**Figure S11-34. ^{13}C NMR of [Bis(MeIm)C3][DMPO$_4$]$_2$.**	---------------------------	S34
**Figure S11-35. ^{1}H NMR of [Bis(MeIm)C6][DMPO$_4$]$_2$.**	---------------------------	S35

**Figure S11-36. ^{13}C NMR of [Bis(MeIm)C6][$DMPO_4$]$_2$**	---------------------------	S35
**Figure S11-37. ^{1}H NMR of [Bis(Pyra-1)C3][$DMPO_4$]$_2$.**	---------------------------	S36
**Figure S11-38. ^{13}C NMR of [Bis(Pyra-1)C3][$DMPO_4$]$_2$.**	---------------------------	S36
**Figure S11-39. ^{1}H NMR of [Bis(Pyra-1)C6][$DMPO_4$]$_2$.**	---------------------------	S37
**Figure S11-40. ^{13}C NMR of [Bis(Pyra-1)C6][$DMPO_4$]$_2$.**	---------------------------	S37
**Figure S11-41. ^{1}H NMR of [Bis(124-Tz-1)C3][$DMPO_4$]$_2$.**	---------------------------	S38
**Figure S11-42. ^{13}C NMR of [Bis(124-Tz-1)C3][$DMPO_4$]$_2$.**	---------------------------	S38
**Figure S11-43. ^{1}H NMR of [Bis(124-Tz-1)C6][$DMPO_4$]$_2$.**	---------------------------	S39
**Figure S11-44. ^{13}C NMR of [Bis(124-Tz-1)C6][$DMPO_4$]$_2$.**	---------------------------	S39
**Figure S12. MD results for partial structure factors of pure TZ8 ([124-Tz-1,8][$DMPO_4$]).**	---------------------------	S40
**Figure S13. MD results for partial structure factors of 80% (w/w) aqueous solution of TZ8 ([124-Tz-1,8][$DMPO_4$])**	---------------------------	S40
**Figure S14. MD results for partial structure factors of 50% (w/w) aqueous solution of TZ8 ([124-Tz-1,8][$DMPO_4$])**	---------------------------	S41
**Table S2. Results of SWAXS analysis of Tz8 ([124-Tz-1,8][$DMPO_4$])**	---------------------------	S42

Table S1. Dehumidification capability of imidazolium, pyrazolium, 1,2,3-triazolium, and 1,2,4-triazolium dimethylnphosphate ILs.

Entry	Compound	Dehumidification capability/mol [a]	Dehumidification Rate/mol [a]	Dehumidification capability/g [a]	Dehumidification Rate/g [a]
		%RH, in mol^{-1}	%RH, in min^{-1}, mol^{-1}	%RH, in g^{-1}	%RH, in min^{-1}, g^{-1}
**1**	C_2mim	$9.9x10^3$	$6.8x10^2$	42	2.9
**2**	C_4mim	$7.4x10^3$	$3.2x10^2$	28	1.2
**3**	Pyra-1,2	$1.7x10^4$	$1.1x10^3$	71	4.8
**4**	Pyra-1,4	$2.2x10^4$	$1.5x10^3$	82	5.5
**5**	Pyra-1,8	$2.6x10^4$	$8.1x10^2$	84	2.6
**6**	124-Tz-1,2	$1.1x10^4$	$7.5x10^2$	47	3.2
**7**	124-Tz-1,4	$1.8x10^4$	$1.3x10^3$	66	4.9
**8**	124-Tz-1,6	$1.7x10^4$	$1.2x10^3$	58	3.9
**9**	124-Tz-1,8	$2.1x10^4$	$8.0x10^2$	68	2.5
**10**	124-Tz-1,10	$2.4x10^4$	$1.4x10^3$	68	2.25
**11**	124-Tz-1,14	$2.8x10^4$	$1.9x10^3$	68	4.7
**12**	124-Tz-1,c6	$3.2x10^4$	$1.6x10^3$	100	5.4
**13**	124-Tz-1,(2-Et)6	$2.4x10^4$	$3.1x10^2$	74	0.95
**14**	123-Tz-1,2	$7.1x10^3$	$4.2x10^2$	30	1.8
**15**	123-Tz-1,4	$6.9x10^3$	$4.4x10^2$	26	1.7
**16**	123-Tz-1,2	$2.3x10^4$	$9.4x10^2$	95	4.0
**17**	123-Tz-1,4	$2.0x10^4$	$1.3x10^3$	77	4.917
**18**	123-Tz-1,2,4	$2.0x10^4$	$1.2x10^3$	68	4.2
**19**	123-Tz-1,4,4	$1.1x10^4$	$4.8x10^2$	34	1.4
**20**	123-Tz-1,4,ME	$4.7x10^3$	$2.5x10^2$	14	0.77
**21**	Bis(MeIm)C3	$3.4x10^4$	$3.4x10^2$	74	0.74
**22**	Bis(MeIm)C6	$4.0x10^4$	$1.2x10^3$	81	2.3
**23**	Bis(MePyra)C3	$3.1x10^4$	$1.2x10^3$	70	2.5
**24**	Bis(MePyra)C6	$4.1x10^4$	$1.2x10^2$	82	2.4
**25**	Bis(124-Tz-1)C3	$4.5x10^4$	$3.1x10^3$	97	6.7
**26**	Bis(124-Tz-1)C6	$3.4x10^4$	$2.4x10^3$	69	4.9
**27**	HMC6[38]	$2.0x10^4$	$8.0x10^2$	37	1.5
**28**	$CaCl_2$ (powder)[b,c]	$2.3x10^3$	$1.4x10^2$	21	1.5

[a] The experiments were conducted following our original method (Ref. 38 in the main text). The details are reported in the SI. [b] We used this salt immediately after opening the package. [c] Nacalai Tesque, Lot MOA0090.

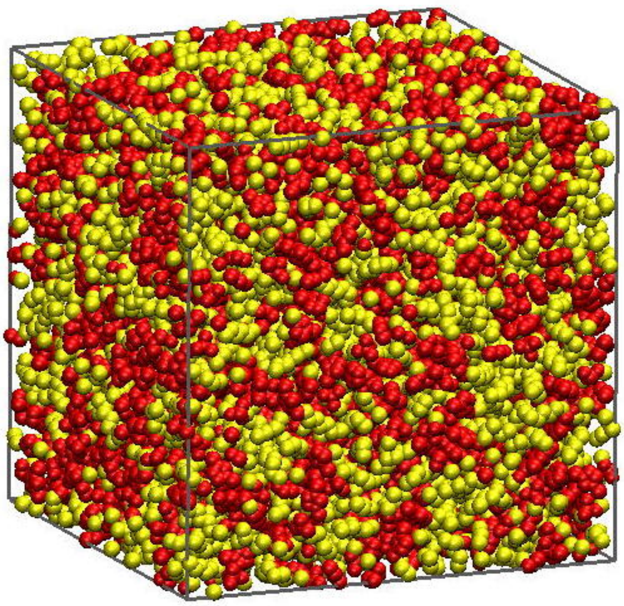

Figure S1. Snapshots of TZ8 ([124-Tz-1,8][$DMPO_4$]) from MD simulations. The simulation temperature was 350 K. The polar domain is shown in red and the nonpolar domain is shown in yellow.

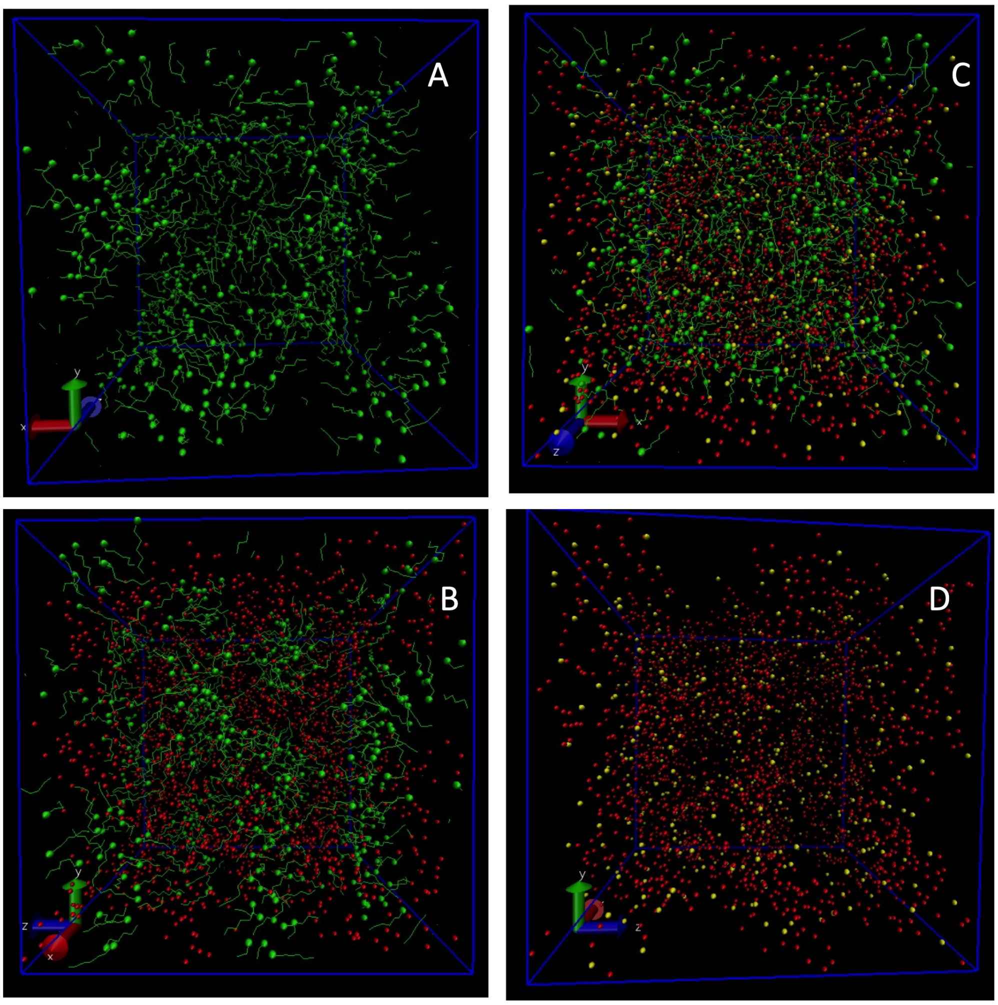

Figure S2. MD snapshots of 80% (w/w) aqueous solution of TZ8 ([124-Tz-1,8][$DMPO_4$]) at T = 350 K. For a clear exposition of the bicontinuous microemulsion structure of the solution, images A, B, C, and D display different groups/atoms of IL ions and water molecules of the same configuration from different angles: (A) nonpolar tail of cations; (B) nonpolar tail of cations + O atoms of anions and water; (C) nonpolar tail of cations + P and O atoms of anions + O atom of water; (D) P and O atoms of anions + O atom of water. The nonpolar tails of TZ8 cations are shown in green, with the terminal carbon atom of each nonpolar tail represented as a green sphere. The oxygen atoms of water and the anions are shown in red, while the phosphorus atoms of the anions are shown in yellow.

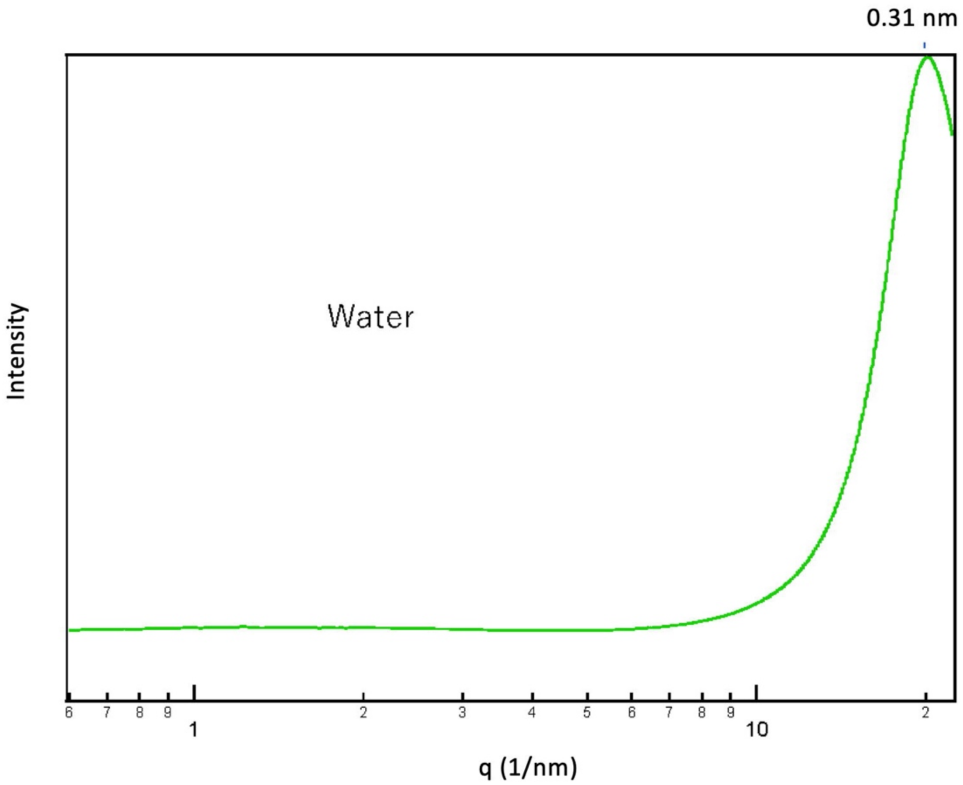

Figure S3. Results of SWAXS analysis of water. Here $q = |\vec{q}| = 4\pi \sin\theta / \lambda$, $d = \lambda / 2\sin\theta = 2\pi/q$.

Determination of the saturated water vapor pressure

(1) The saturated water vapor pressure was measured using the equipment shown in Figure S4 of this study. We used a Pirani pressure gauge (OKANO iPasCAl) for measuring the water vapor pressure. The experiment was carried out as follows: 10.0 g of a sample aqueous solution was added to a 200 mL two-necked flask connected to a Pirani vacuum gauge through a three-way stopcock. Then the flask was placed in the water bath. The bath temperature was initially cooled to ca. 5 °C until the solution temperature was below 8 °C, and then it was connected to the vacuum line. After the pressure reached a constant level (ca. 8-10 hPa), the vacuum line was closed and the bath temperature was gradually elevated to 70 °C for 15 min. Then the saturated water vapor pressure data were recorded at each temperature point until the temperature of the sample solution reached 60 °C.

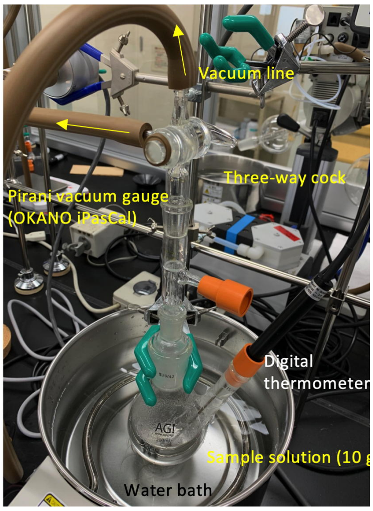

Figure S4 The experimental apparatus for measuring the saturated water vapor pressure.

(2) The equilibrium water vapor pressure was measured using the equipment as reported in our previous study, using a Pirani pressure gauge (OKANO iPasCAl):[38] 10.0 g of a sample aqueous solution was added to a 200 mL two-necked flask, and 10 mL of ultrapure water (milli-Q H_2O) was added to a 100 mL flask. The two flasks were connected to a Pirani vacuum gauge through a three-way stopcock. Then the two flasks were placed in the water bath. The bath temperature was initially cooled to ca. 5 °C until the solution temperature was below 8 °C, and then it was connected to the vacuum line. After the pressure reached a constant level (ca. 8-10 hPa), the vacuum line was closed and the bath temperature was gradually elevated to 65 °C for 15 min. The equilibrium water vapor pressure data were then recorded at each temperature point until the temperature of the sample solution reached 60 °C.

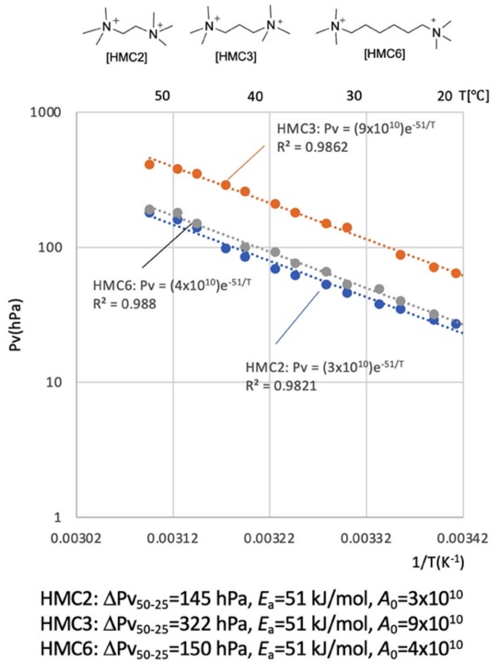

Figure S5. Arrhenius plot of *T*-depended saturated water vapor pressure of three types of dicationic quaternary ammonium bis(dimethyl phosphate) 80% aq. solutions.

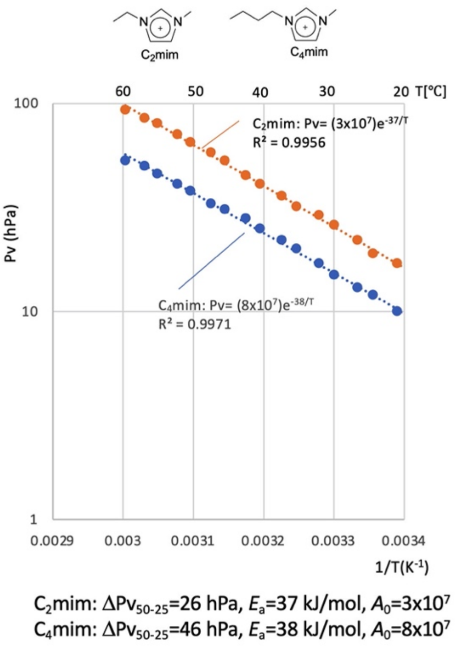

Figure S6. Arrhenius plot of *T*-depended saturated water vapor pressure change of C_2mim or C_4mim dimethyl phosphate 80% aq. solution.

As mentioned in the main text, we conducted a thermophysical investigation of three types of 80% (w/w) aqueous solutions of the dicationic quaternary ammonium salts, i.e., HMC2,[35,38] HMC3,[38] and HMC6 [38]; the E_a values of these solutions were the same (51 kJ/mol), and the vapor pressure (Pv) depended on their pre-exponential factor (A_0) (Figure S5). The same trend was observed for the *T*-dependent water vapor pressures of [C_2mim][$DMPO_4$] and of [C_4mim][$DMPO_4$] (Figure S6). Since we obtained these results using a very simple experimental apparatus (Figure S4), we conducted a control experiment using pure water. The results were shown in Figure S7. As can be seen there, the results were in complete agreement with the reference data of the Society of Japan Mechanical Engineers (Figure S7). The results provide confidence in the reliability of the experimental results shown in Figures S5 and S6.

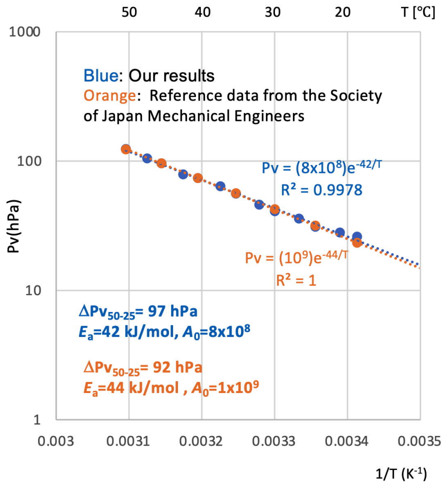

Figure S7. Arrhenius plot of *T*-depended saturated water vapor pressure.

Determination of the dehumidification capability

We evaluated the dehumidification capability of the ILs by the same method reported in our previous article.[33,35,38] The experiment was carried out as follows: a sealed Ziplock® freezer bag (L) that includes the desiccant sample (ca. 1.0 g) in a lab dish (ϕ 32 x15 mm) and the humidity sensor (TR-74Ui) was placed on a stainless steel dish (170 mm x 210 mm x 30 mm) in an oven (at 30°C). The volume in the bag was ca. 1,150 cm^3. The dehumidification capability (DC) was calculated by the record of humidity (%RH) change inside the freezer bag. The dehumidification rate of the sample was determined by the time it reached a 50% humidity between the maximum humidity and that of the lowest one after reaching the equilibrium state. The dehumidification capability per mol (DC (mol)), the dehumidification rate per mol (rate (mol)), the dehumidification capability per gram (DC (g)), and the dehumidification rate per gram (Rate (g)) were calculated by the equations shown in Figure S8. Although this method was not applicable to measure the accurate dehumidification capability, we obtained reproducible results with significant figures of two digits. Therefore, it was useful for the evaluation of the dehumidification capability of the various desiccant materials. The Ziplock® freezer bags were purchased from Asahi Kasei Home Products, Ltd.

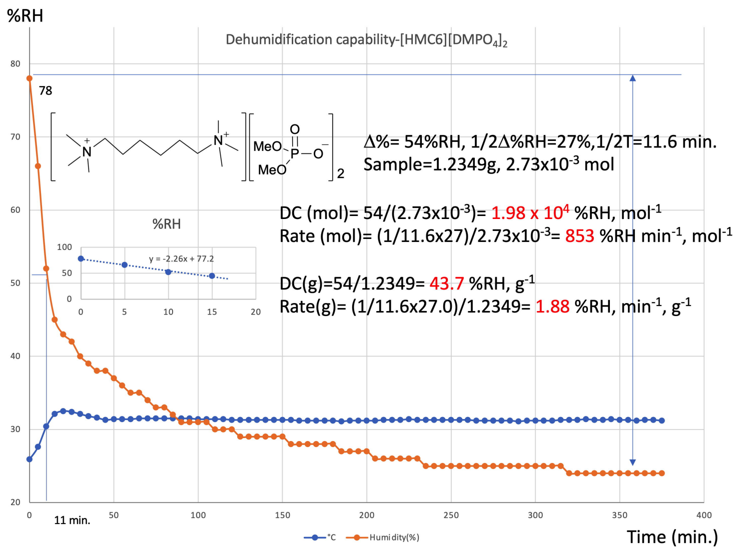

Figure S8. Representative example of determination of dehumidification capability (DC): [HMC6][$DMPO_4$]$_2$.[38]

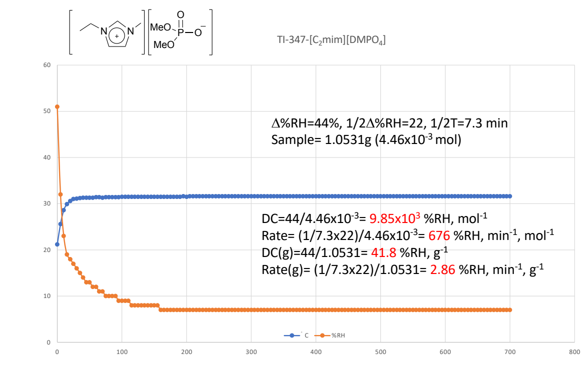

Figure S9-1. The dehumidification capability of [C_2mim][$DMPO_4$].

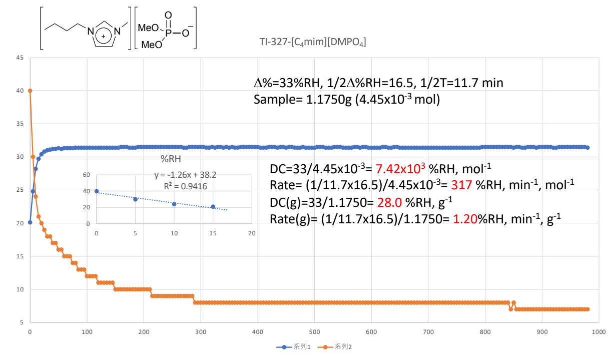

Figure S9-2. The dehumidification capability of [C_4mim][$DMPO_4$].

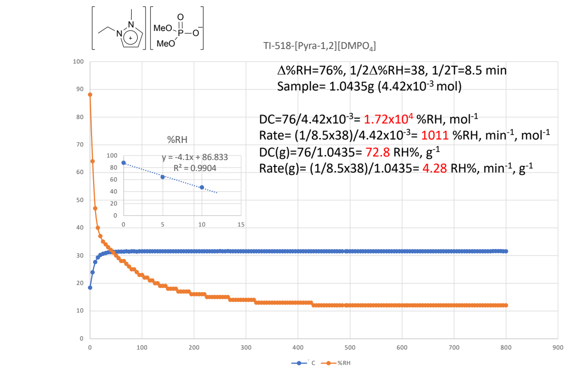

Figure S9-3. The dehumidification capability of [Pyra-1,2][$DMPO_4$].

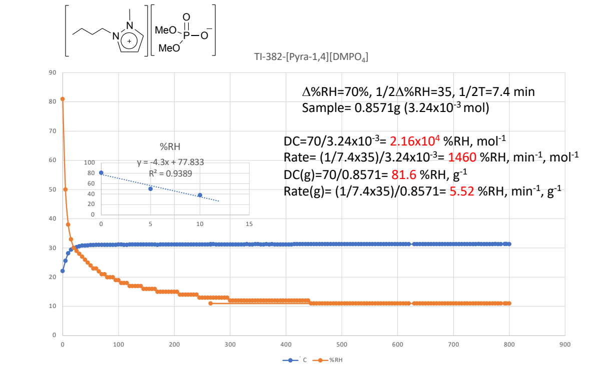

Figure S9-4. The dehumidification capability of [Pyra-1,4][$DMPO_4$].

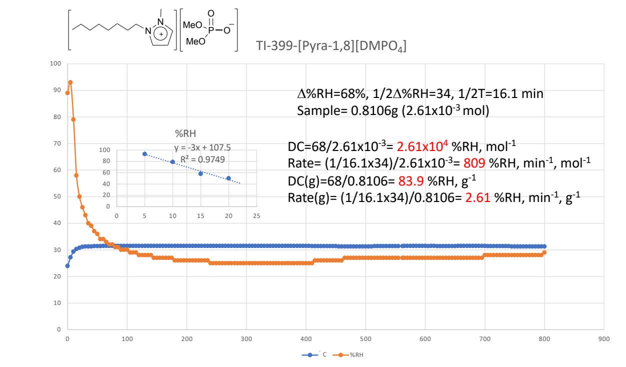

Figure S9-5. The dehumidification capability of [Pyra-1,8][DMPO4].

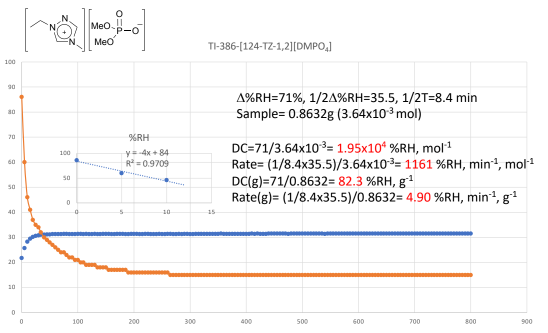

Figure S9-6. The dehumidification capability of [124-Tz-1,2][DMPO4].

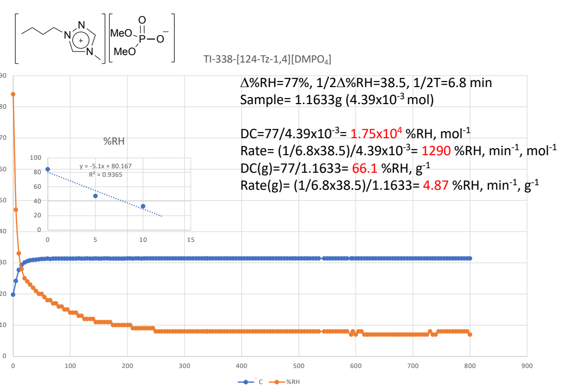

Figure S9-7. The dehumidification capability of [124-Tz-1,4][DMPO4].

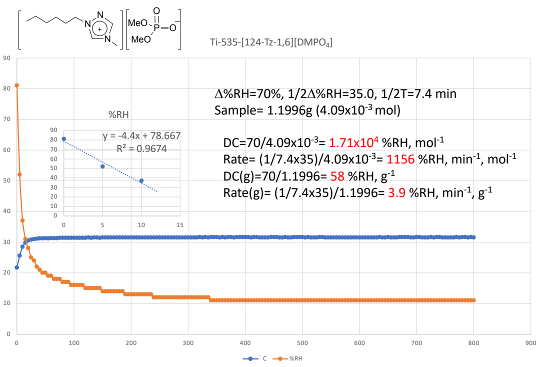

Figure S9-8. The dehumidification capability of [124-Tz-1,6][DMPO4].

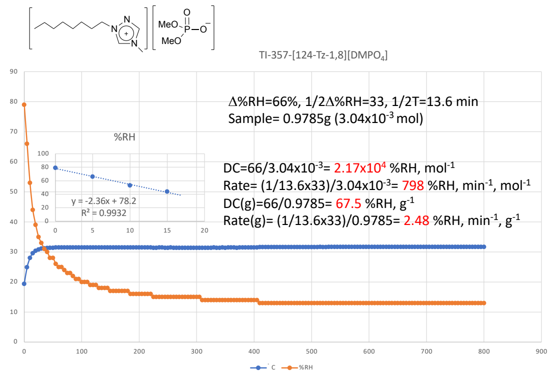

Figure S9-9. The dehumidification capability of [124-Tz-1,8][DMPO4].

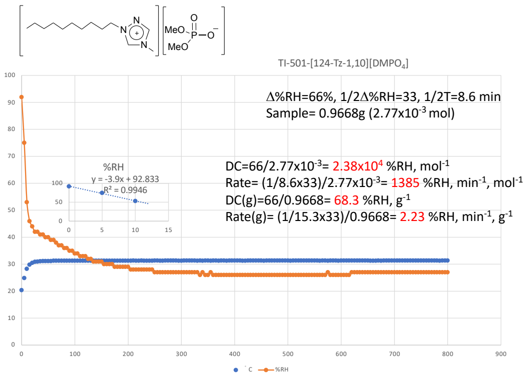

Figure S9-10. The dehumidification capability of [124-Tz-1,10][DMPO4].

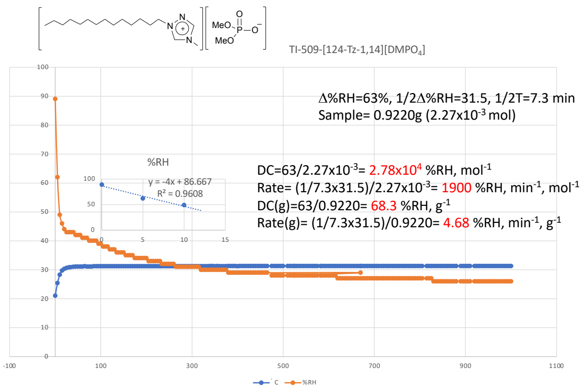

Figure S9-11. The dehumidification capability of [124-Tz-1,14][DMPO$_4$].

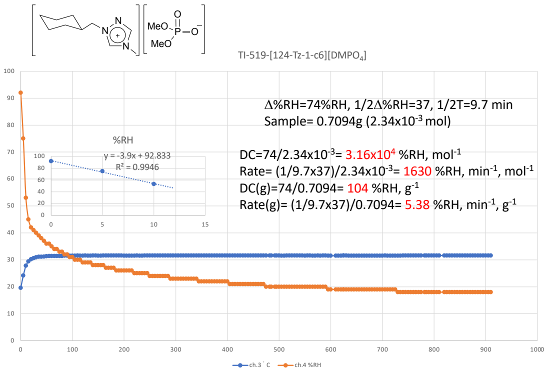

Figure S9-12. The dehumidification capability of [124-Tz-1,c6][DMPO$_4$].

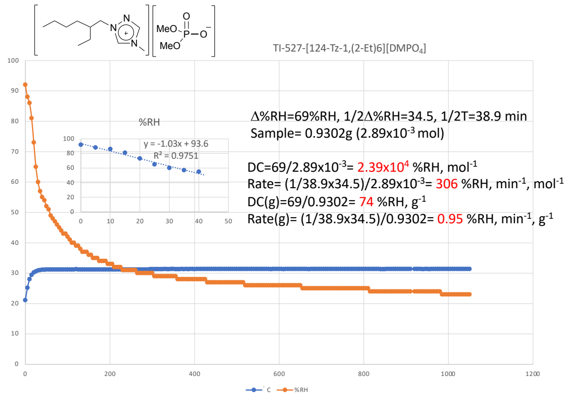

Figure S9-13. The dehumidification capability of [124-Tz-1,(2-Et)6][DMPO$_4$].

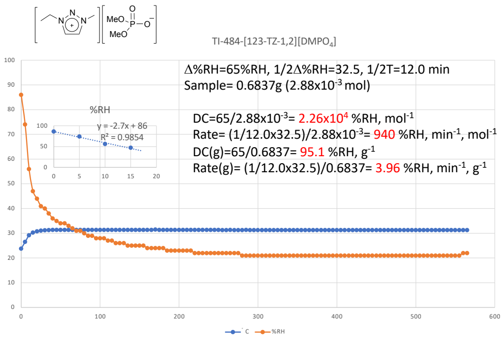

Figure S9-14. The dehumidification capability of [123-Tz-1,2][DMPO$_4$].

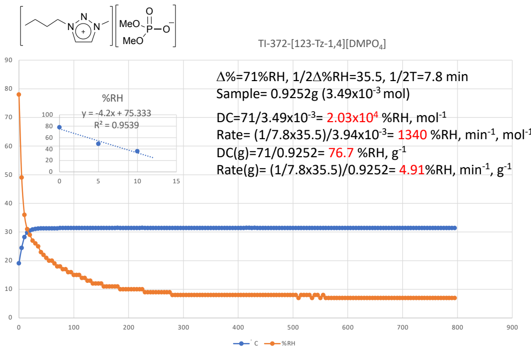

Figure S9-15. The dehumidification capability of [123-Tz-1,4][DMPO$_4$].

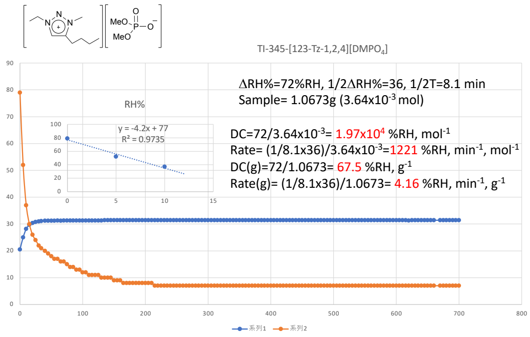

Figure S9-16. The dehumidification capability of [123-Tz-1,2,4][DMPO$_4$].

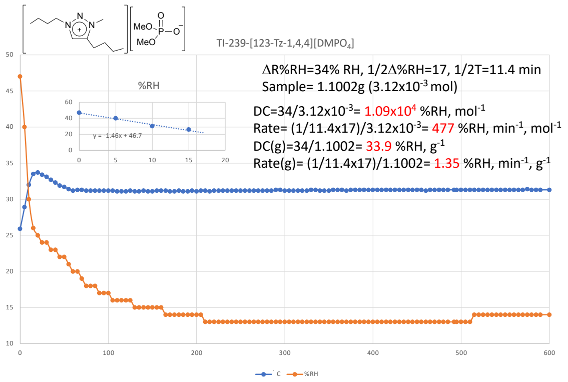

Figure S9-17. The dehumidification capability of [123-Tz-1,4,4][DMPO₄].

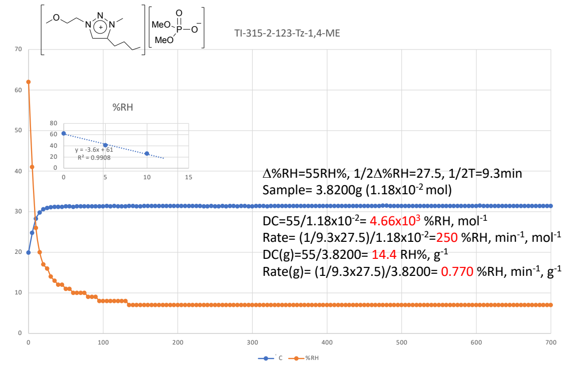

Figure S9-18. The dehumidification capability of [123-Tz-1,4,ME][DMPO₄].

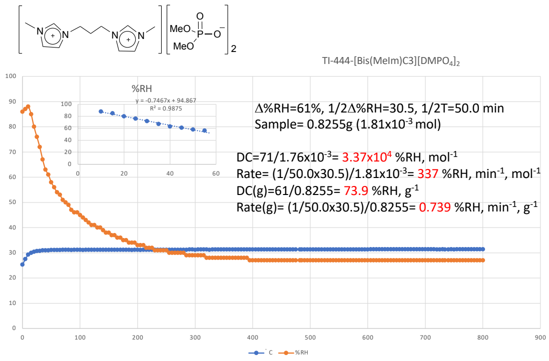

Figure S9-19. The dehumidification capability of [Bis(MeIm)C3][DMPO₄]₂.

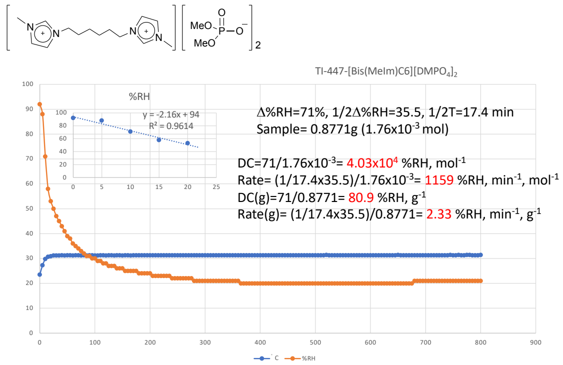

Figure S9-20. The dehumidification capability of [Bis(MeIm)C6][DMPO₄]₂.

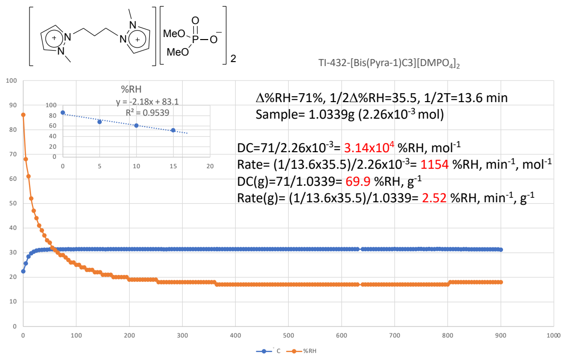

Figure S9-21. The dehumidification capability of [Bis(Pyra-1)C3][DMPO₄]₂.

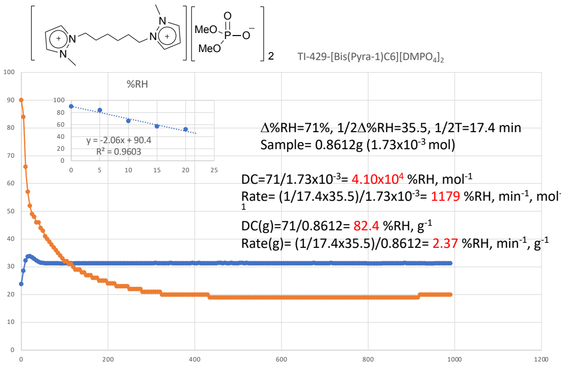

Figure S9-22. The dehumidification capability of [Bis(Pyra-12)C6][DMPO₄]₂.

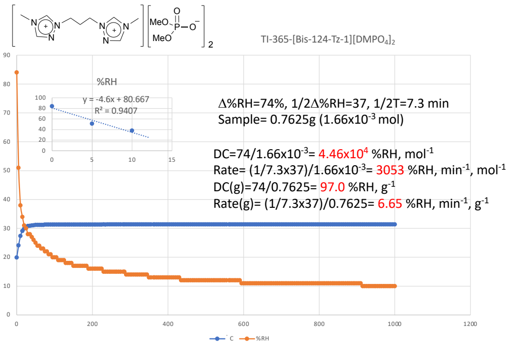

Figure S9-23. The dehumidification capability of [Bis(124-Tz-1)C3][DMPO$_4$]$_2$.

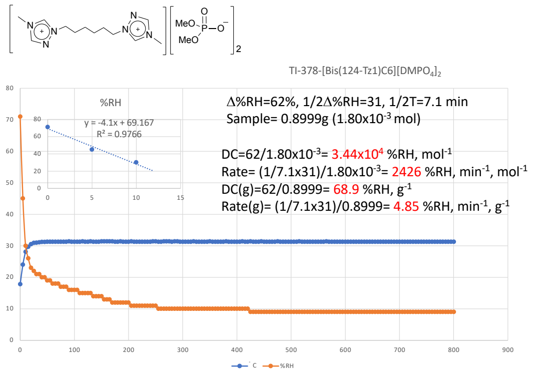

Figure S9-24. The dehumidification capability of [Bis(124-Tz-1)C6][DMPO$_4$]$_2$.

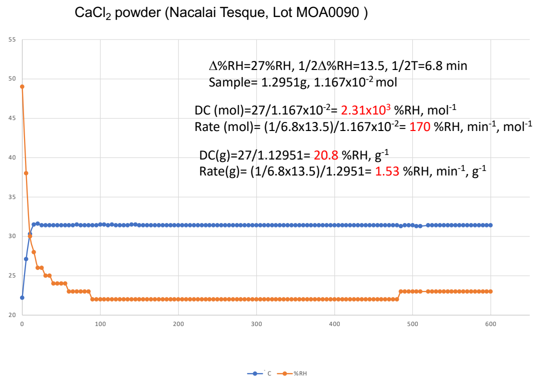

Figure S9-25. The dehumidification capability of $CaCl_2$ Powder, (Nacalai Tesque, Lot. MOA0090).

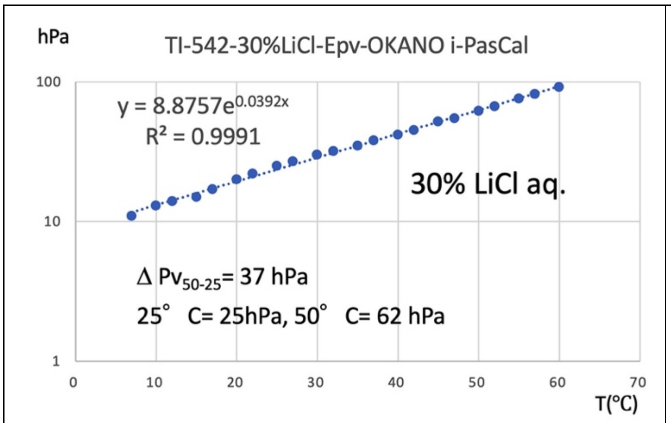

Figure S10-1. The equilibrium water vapor pressure of 30%(w/w) LiCl aq. solution.

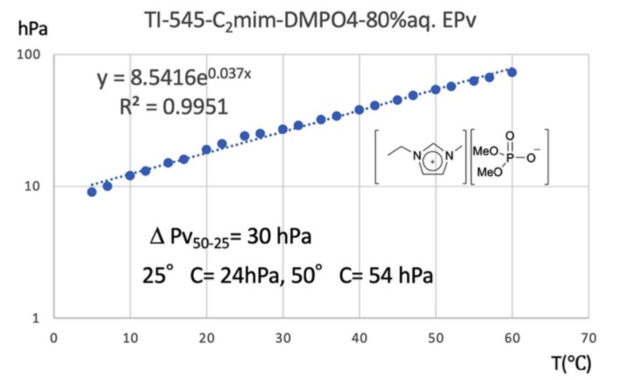

Figure S10-2. The equilibrium water vapor pressure of [C_2mim][$DMPO_4$] 80% aq. solution.

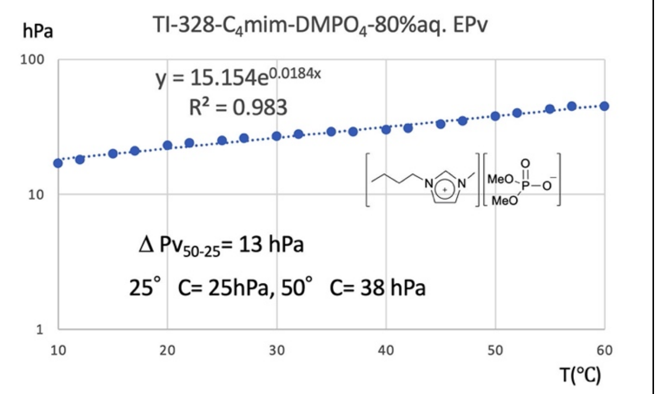

Figure S10-3. The equilibrium water vapor pressure of [C_4mim][$DMPO_4$] 80% aq. solution.

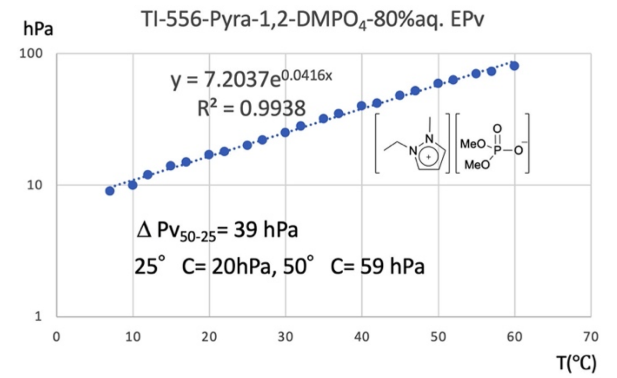

Figure S10-4. The equilibrium water vapor pressure of [Pyra-1,2][$DMPO_4$] 80% aq. solution.

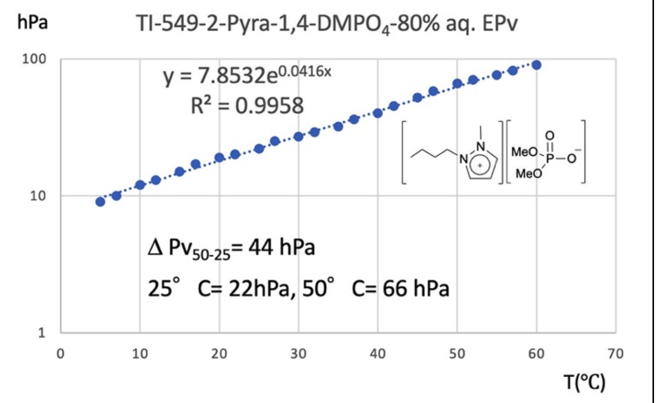

Figure S10-5. The equilibrium water vapor pressure of [Pyra-1,4][$DMPO_4$] 80% aq. solution.

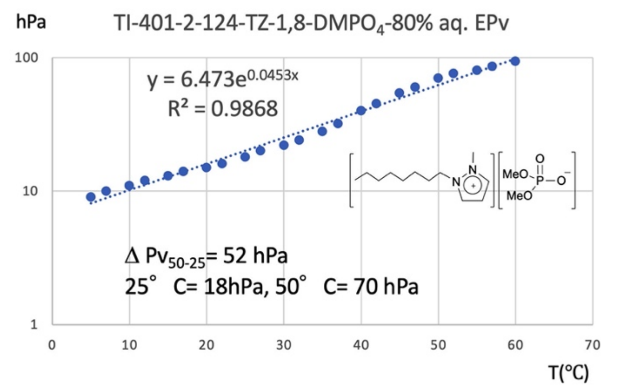

Figure S10-6. The equilibrium water vapor pressure of [Pyra-1,8][$DMPO_4$] 80% aq. solution.

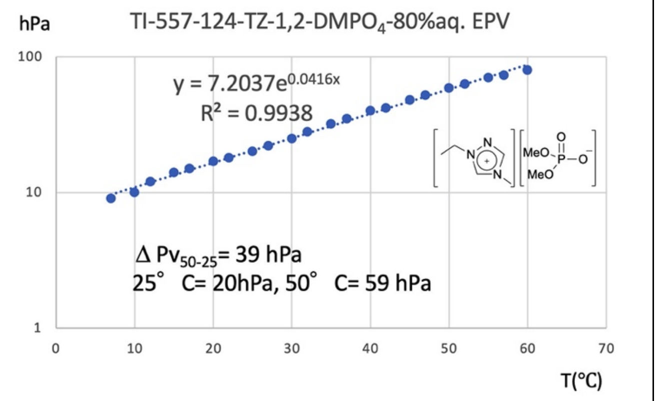

Figure S10-7. The equilibrium water vapor pressure of [124-Tz-1,2][$DMPO_4$] 80% aq. solution.

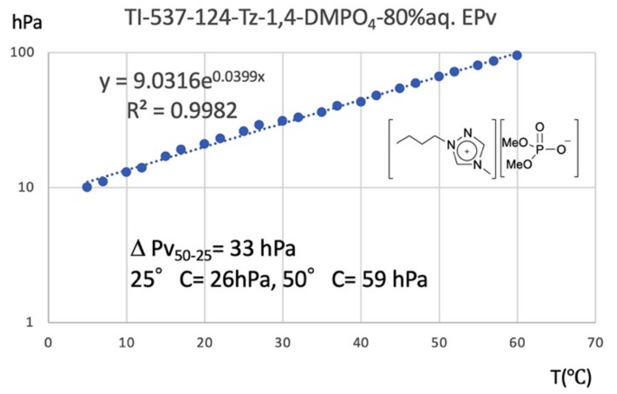

Figure S10-8. The equilibrium water vapor pressure of [124-Tz-1,4][$DMPO_4$] 80% aq. solution.

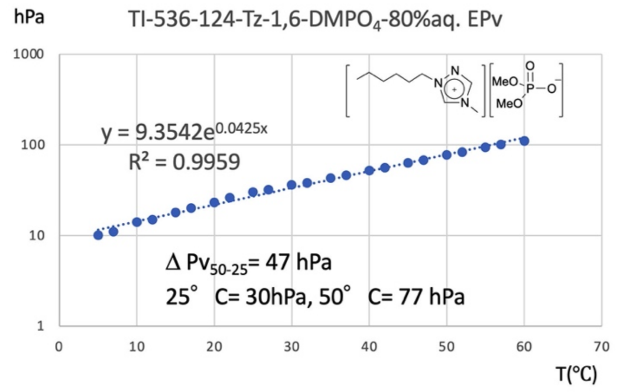

Figure S10-9. The equilibrium water vapor pressure of [124-Tz-1,6][$DMPO_4$] 80% aq. solution.

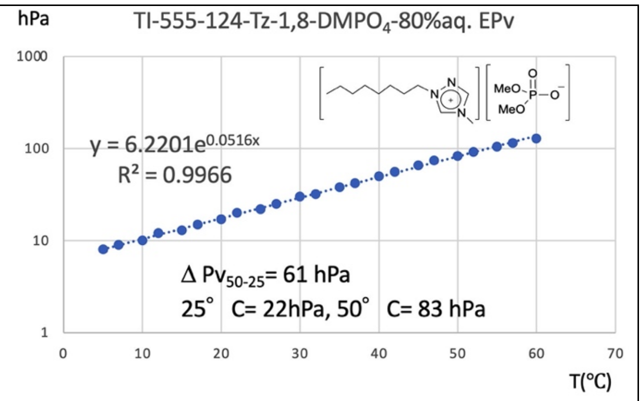

Figure S10-10. The equilibrium water vapor pressure of [124-Tz-1,8][$DMPO_4$] 80% aq. solution.

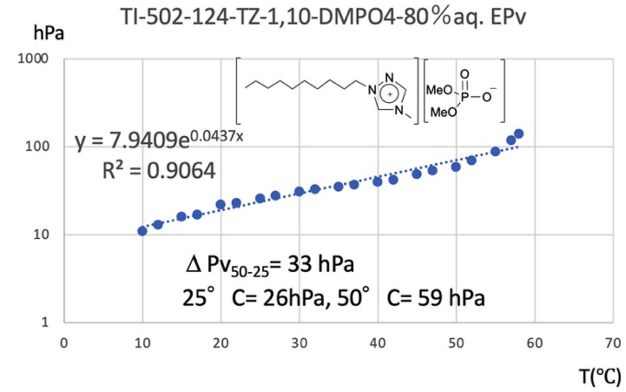

Figure S10-11. The equilibrium water vapor pressure of [124-Tz-1,10][$DMPO_4$] 80% aq. solution.

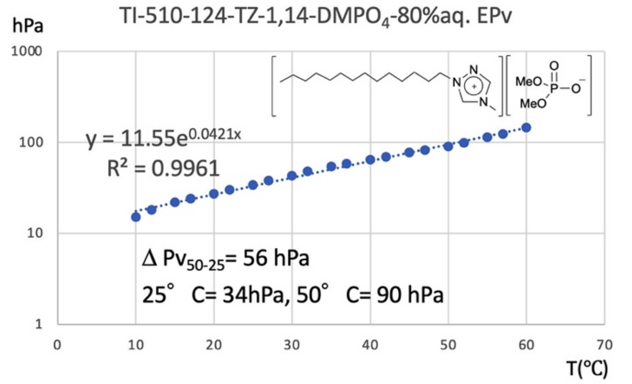

Figure S10-12. The equilibrium water vapor pressure of [124-Tz-1,14][$DMPO_4$] 80% aq. solution.

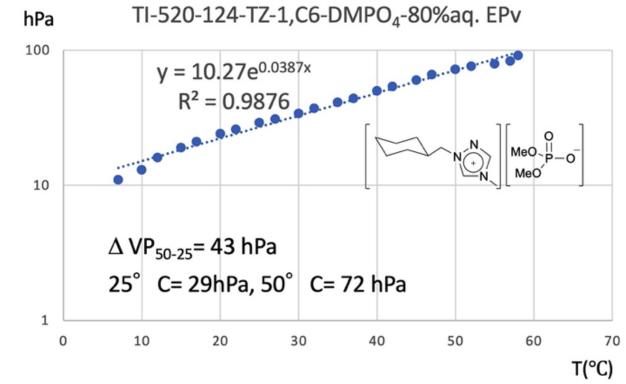

Figure S10-13. The equilibrium water vapor pressure of [124-Tz-1,c6][$DMPO_4$] 80% aq. solution.

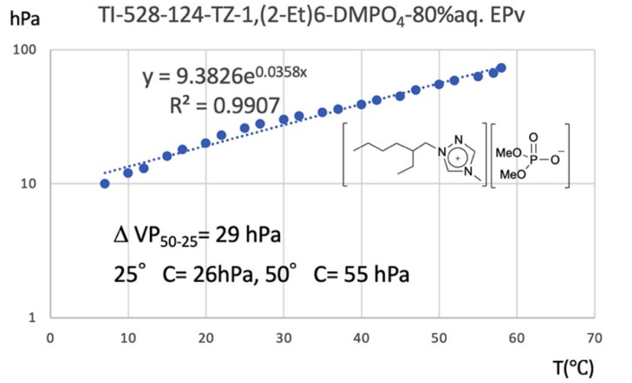

Figure S10-14. The equilibrium water vapor pressure of [124-Tz-1,(2-Et)6][$DMPO_4$] 80% aq. solution.

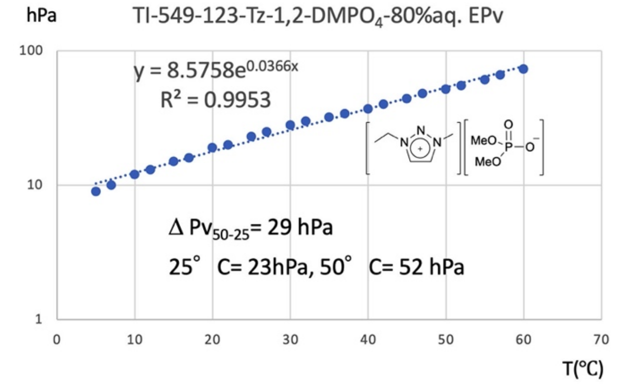

Figure S10-15. The equilibrium water vapor pressure of [123-Tz-1,2][$DMPO_4$] 80% aq. solution.

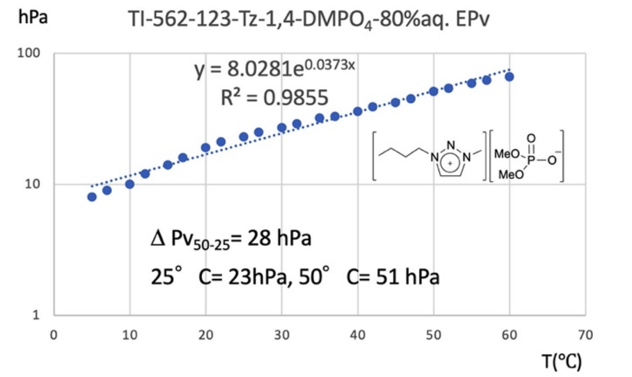

Figure S10-16. The equilibrium water vapor pressure of [123-Tz-1,4][$DMPO_4$] 80% aq. solution.

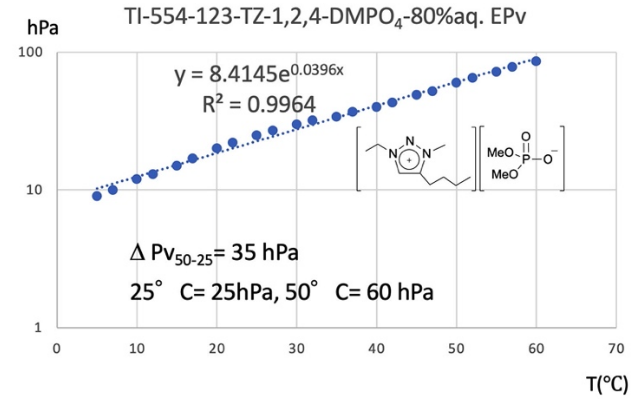

Figure S10-17. The equilibrium water vapor pressure of [123-Tz-1,2,4][DMPO4] 80% aq. solution.

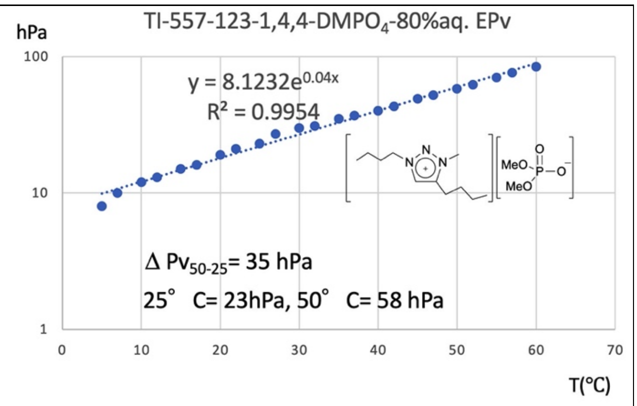

Figure S10-18. The equilibrium water vapor pressure of [123-Tz-1,4,4][DMPO4] 80% aq. solution.

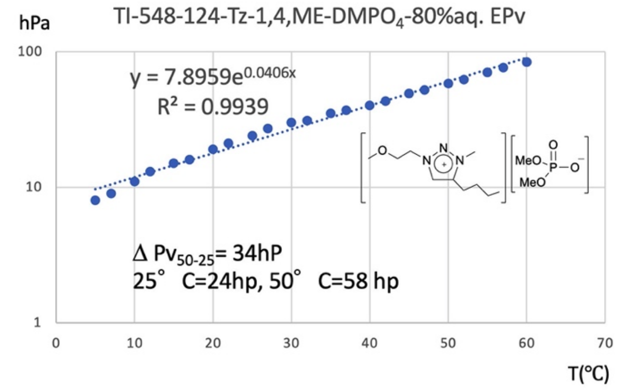

Figure S10-19. The equilibrium water vapor pressure of [123-Tz-1,4,ME][DMPO4] 80% aq. solution.

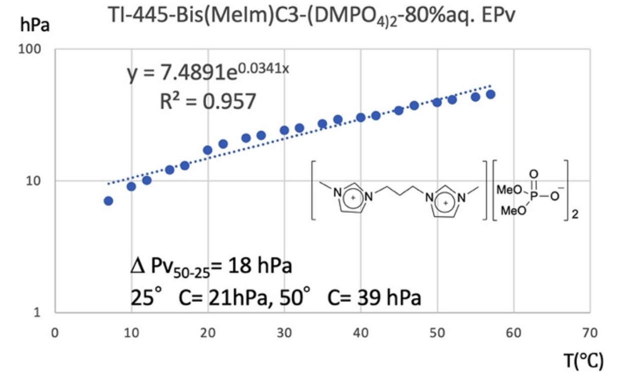

Figure S10-20. The equilibrium water vapor pressure of [Bis(MeIm)C3][DMPO4]2 80% aq. solution.

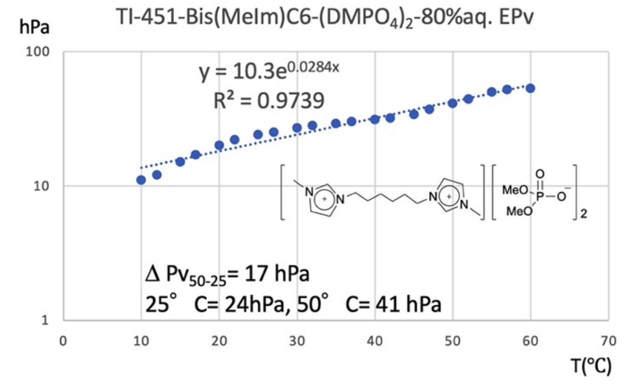

Figure S10-21. The equilibrium water vapor pressure of [Bis(MeIm)C6][DMPO4)]2 80% aq. solution.

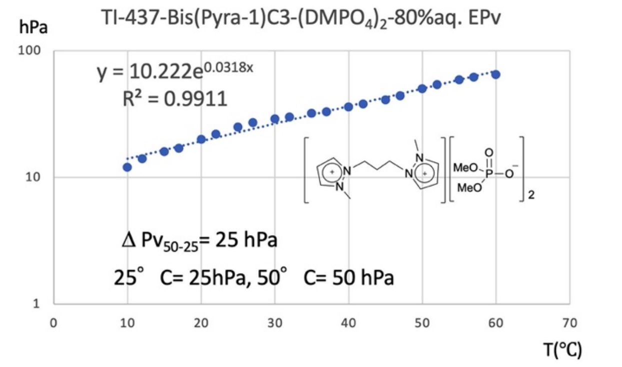

Figure S10-22. The equilibrium water vapor pressure of [Bis(Pyra-1)C3][DMPO4]2 80% aq. solution.

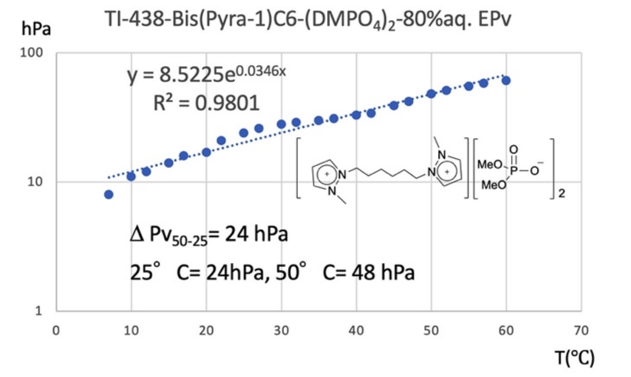

Figure S10-23. The equilibrium water vapor pressure of [Bis(Pyra-1)C6][DMPO4]2 80% aq. solution.

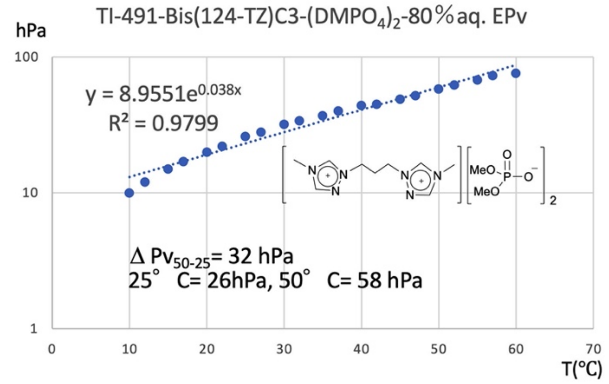<br>**Figure S10-24.** The equilibrium water vapor pressure of [Bis(124-Tz-1)C3][DMPO4]2 80% aq. solution.	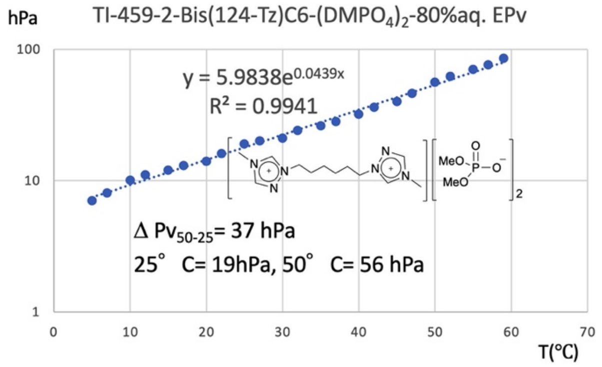<br>**Figure S10-25.** The equilibrium water vapor pressure of [Bis(124-Tz-1)C6][DMPO4]2 80% aq. solution.

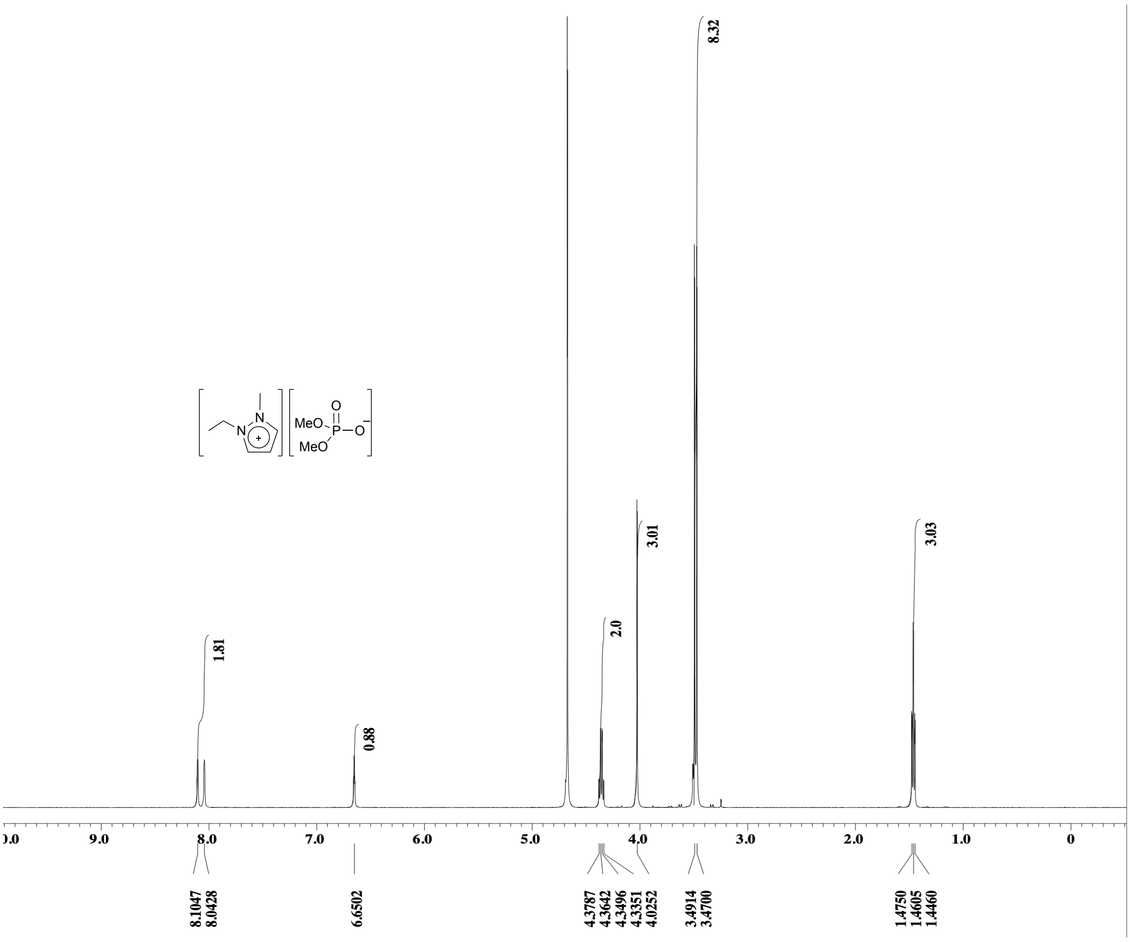

Figure S11-1. ^{1}H NMR of [Pyra-1,2][DMPO$_4$]. (500 MHz, $CDCl_3$)

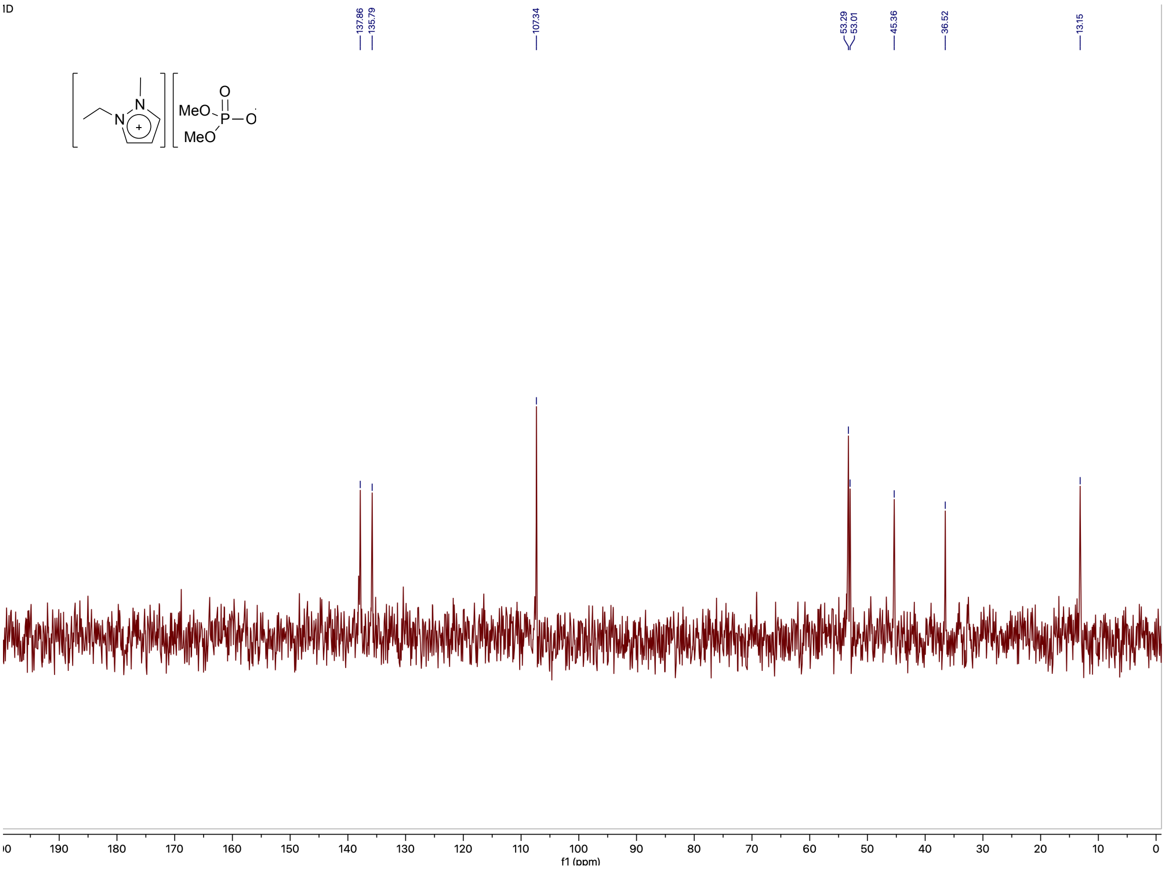

Figure S11-2. ^{13}C NMR of [Pyra-1,2][DMPO$_4$] (20 MHz-D_2O).

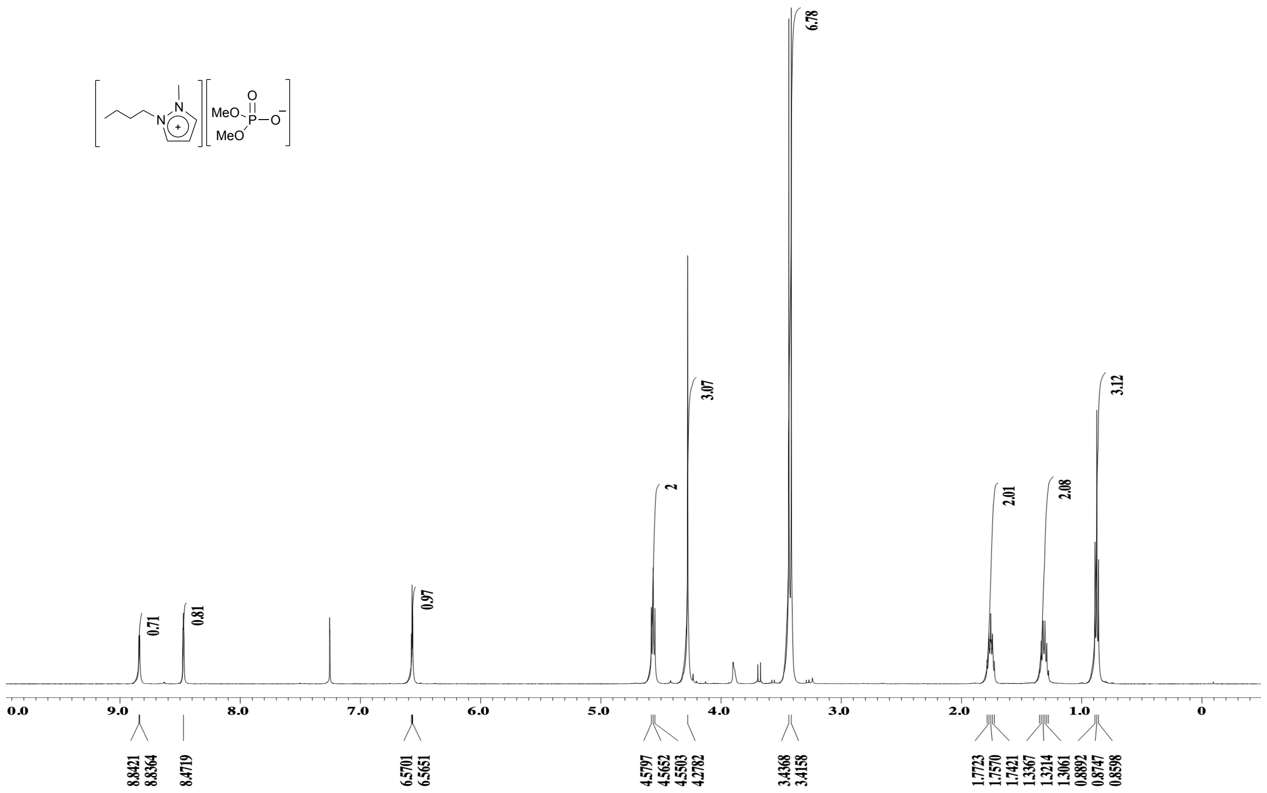

Figure S11-3. ^{1}H NMR of [Pyra-1,4][DMPO$_4$] (500 MHz, CDCl$_3$).

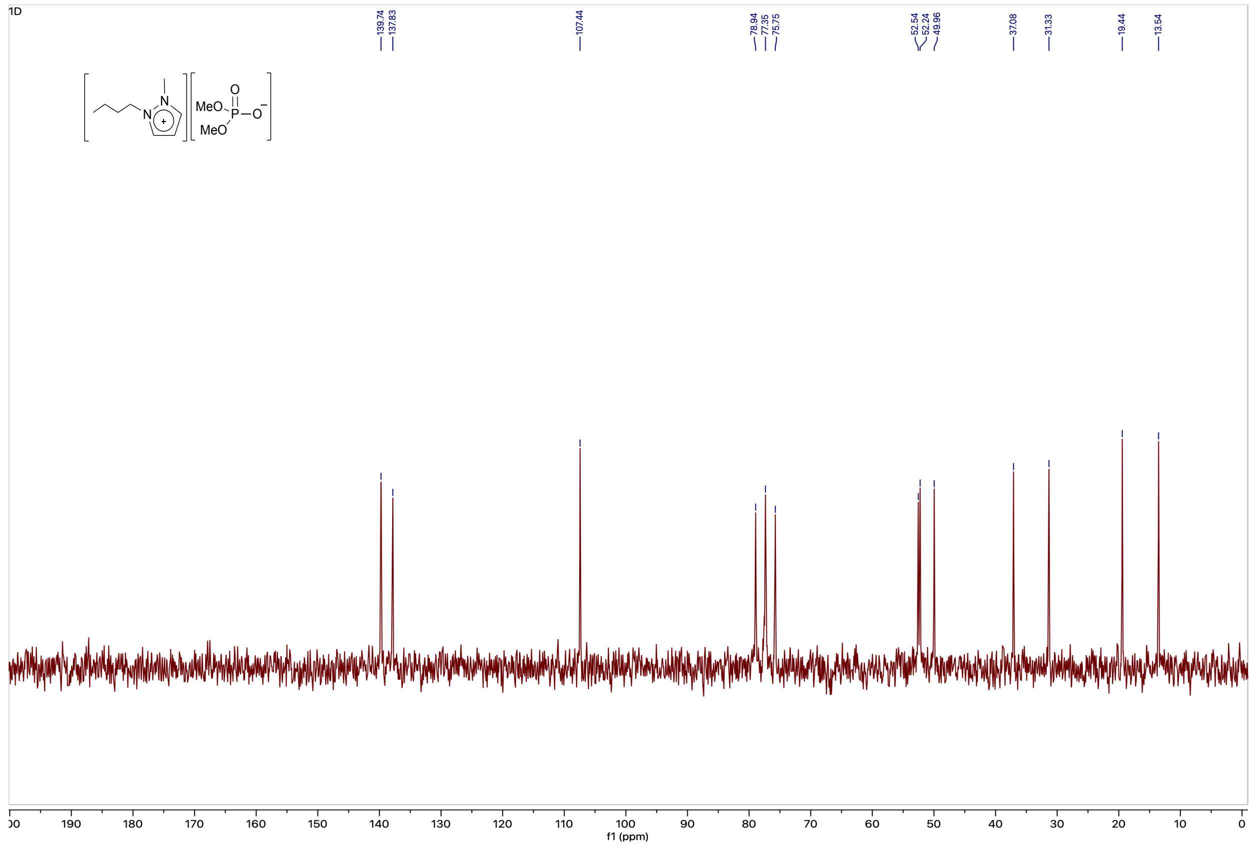

Figure S11-4. ^{13}C NMR of [Pyra-1,4][DMPO$_4$] (20 MHz, CDCl$_3$)

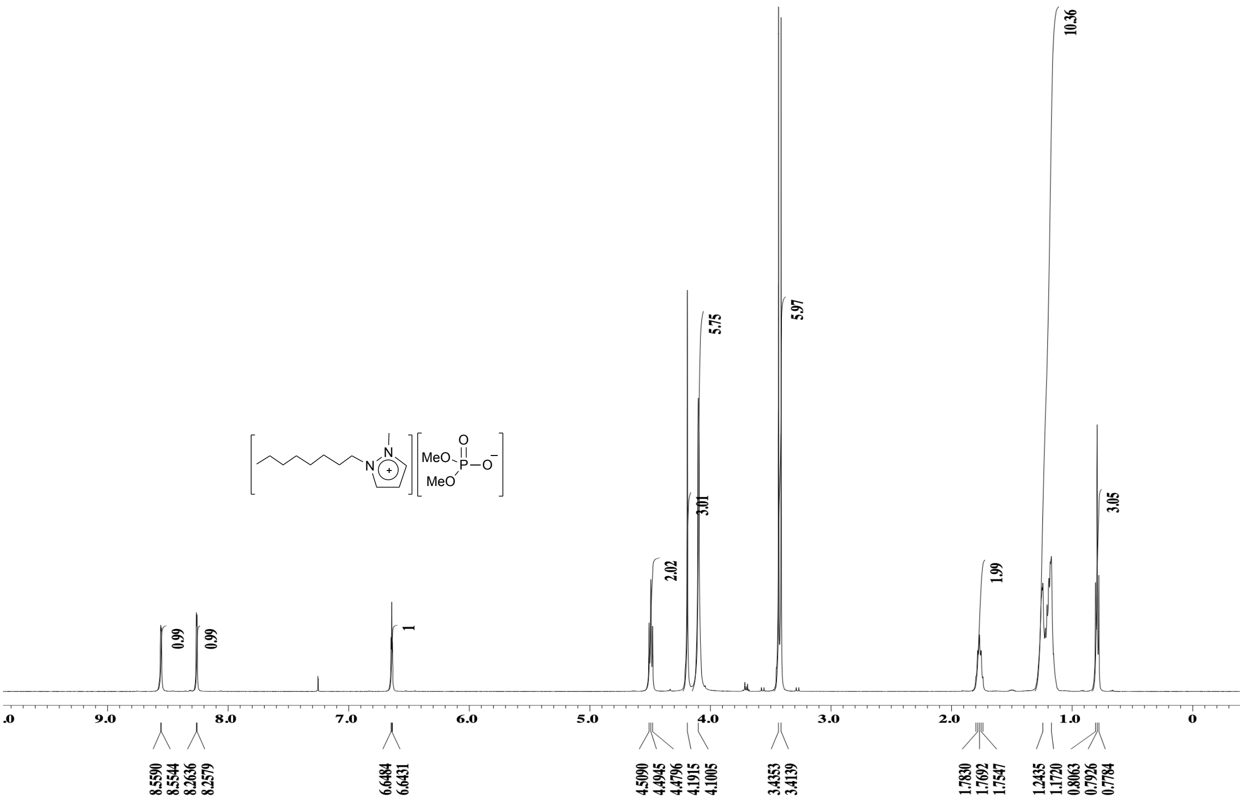

Figure S11-5. ^{1}H NMR of [Pyra-1,8][DMPO$_4$] (500 MHz, $CDCl_3$).

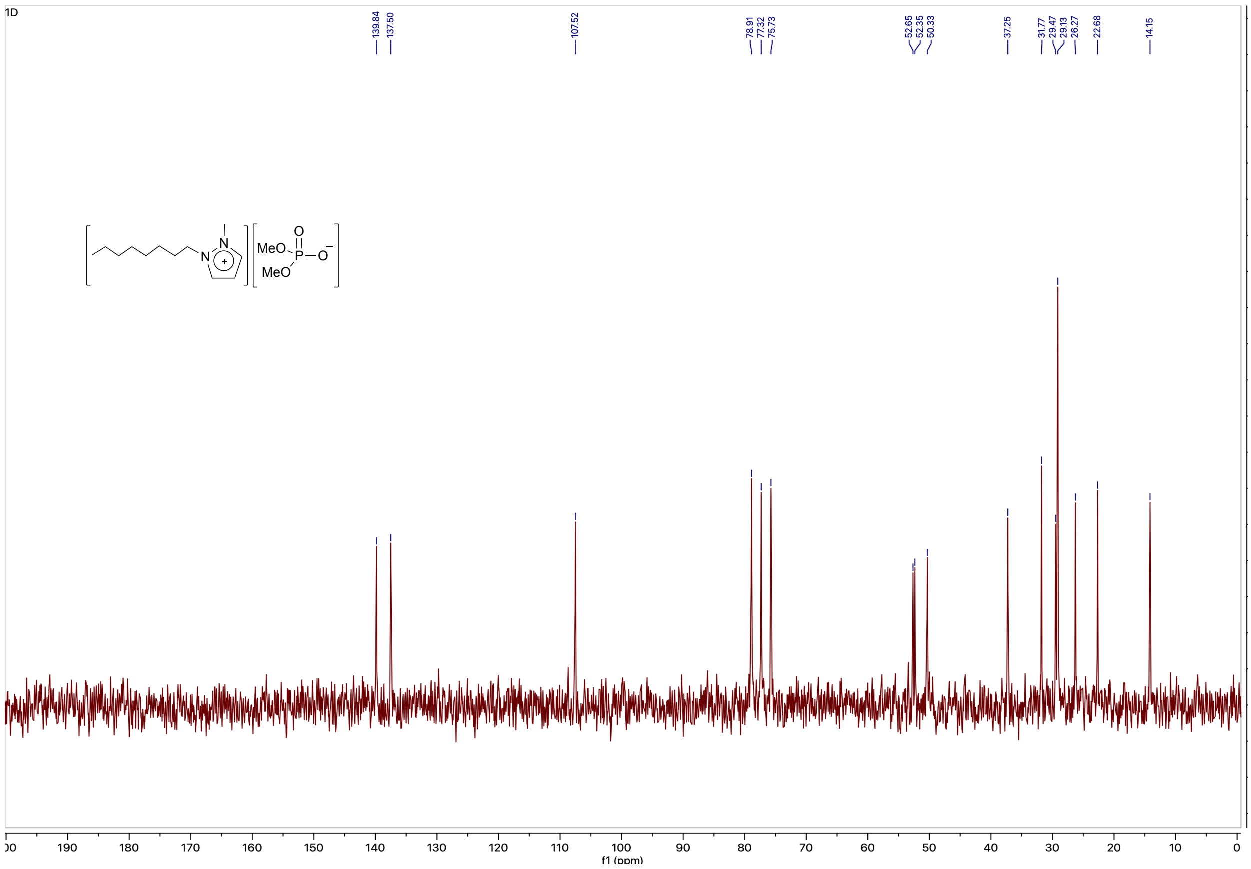

Figure S11-6. ^{13}C NMR of [Pyra-1,8][DMPO$_4$] (20 MHz, $CDCl_3$).

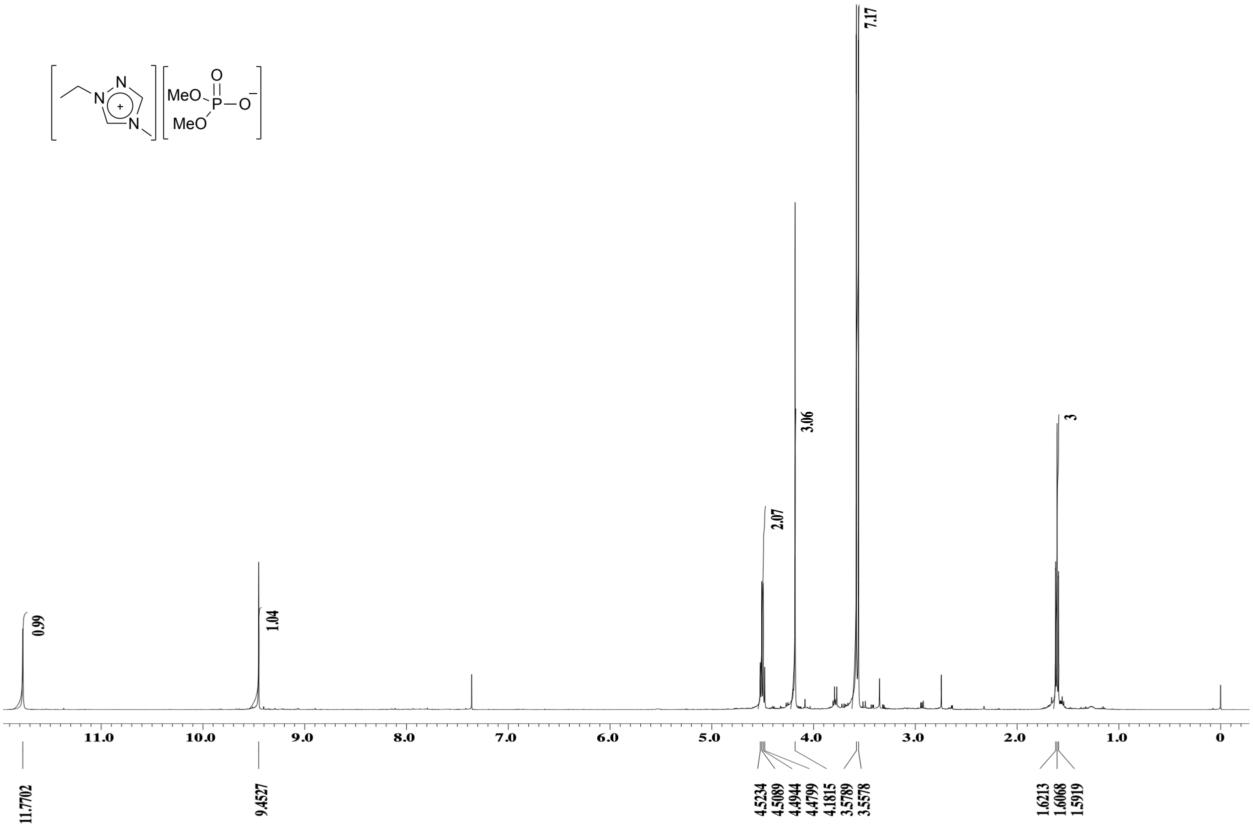

Figure S11-7. ^{1}H NMR of [124-Tz-1,2][DMPO$_4$] (500 MHz, $CDCl_3$)

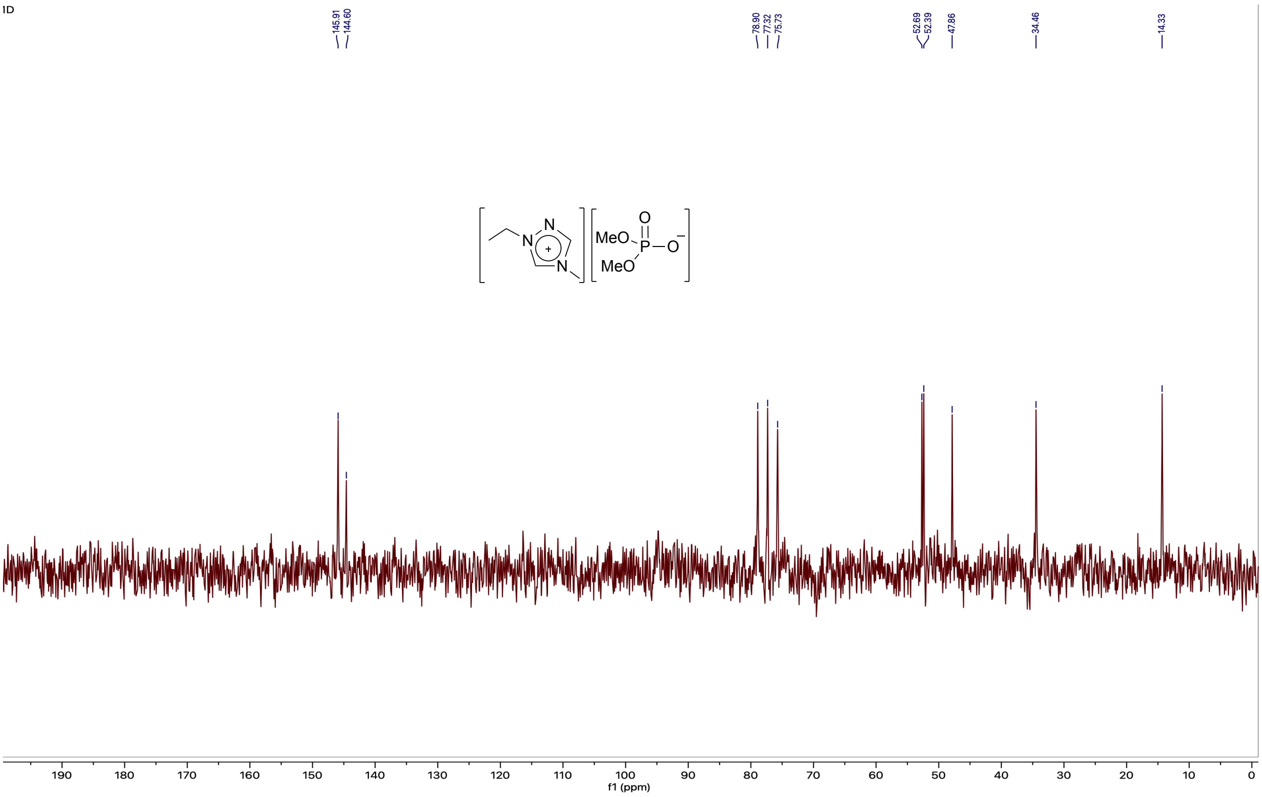

Figure S11-8. ^{13}C NMR of [124-Tz-1,2][DMPO$_4$] (20 MHz, CDCl3).

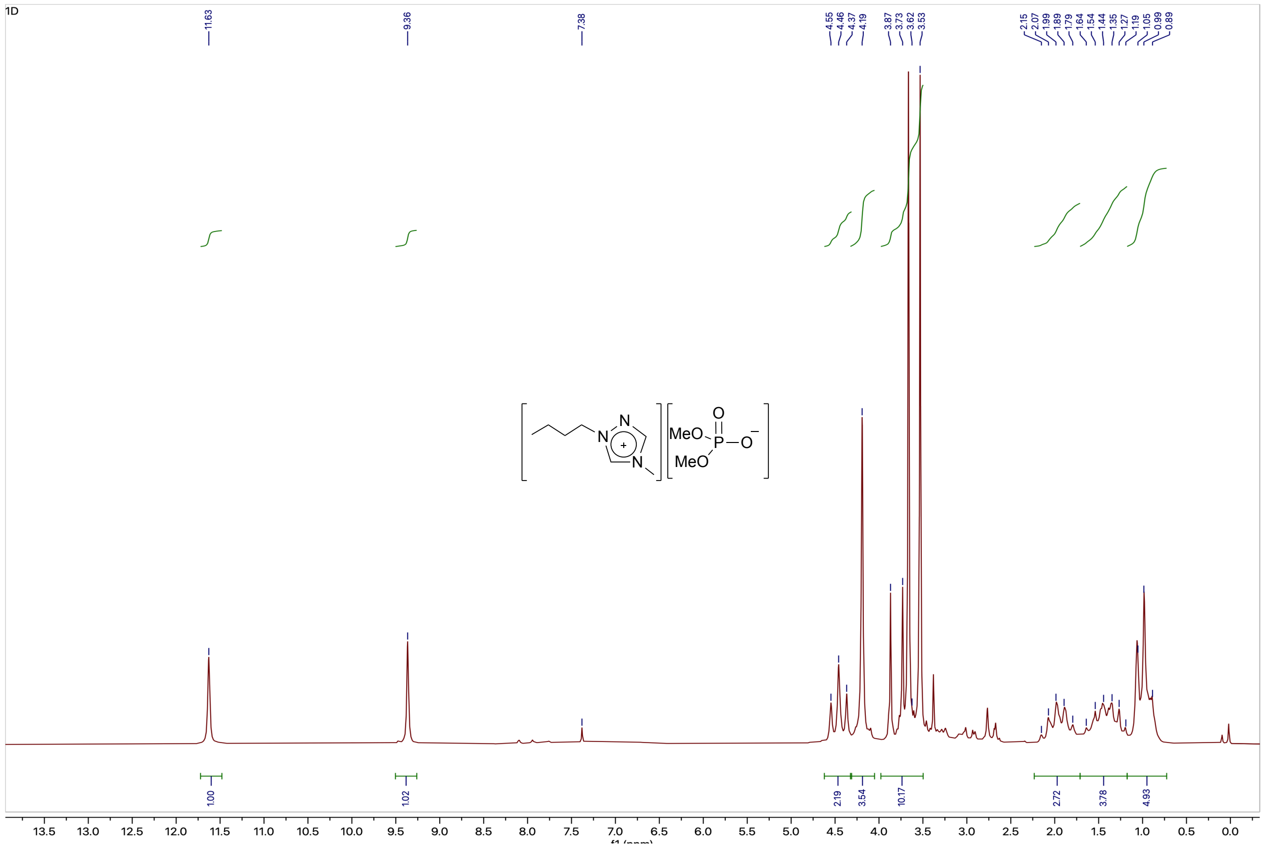

Figure S11-9. ^{1}H NMR of [124-Tz-1,4][DMPO$_4$]. (80 MHz, $CDCl_3$).

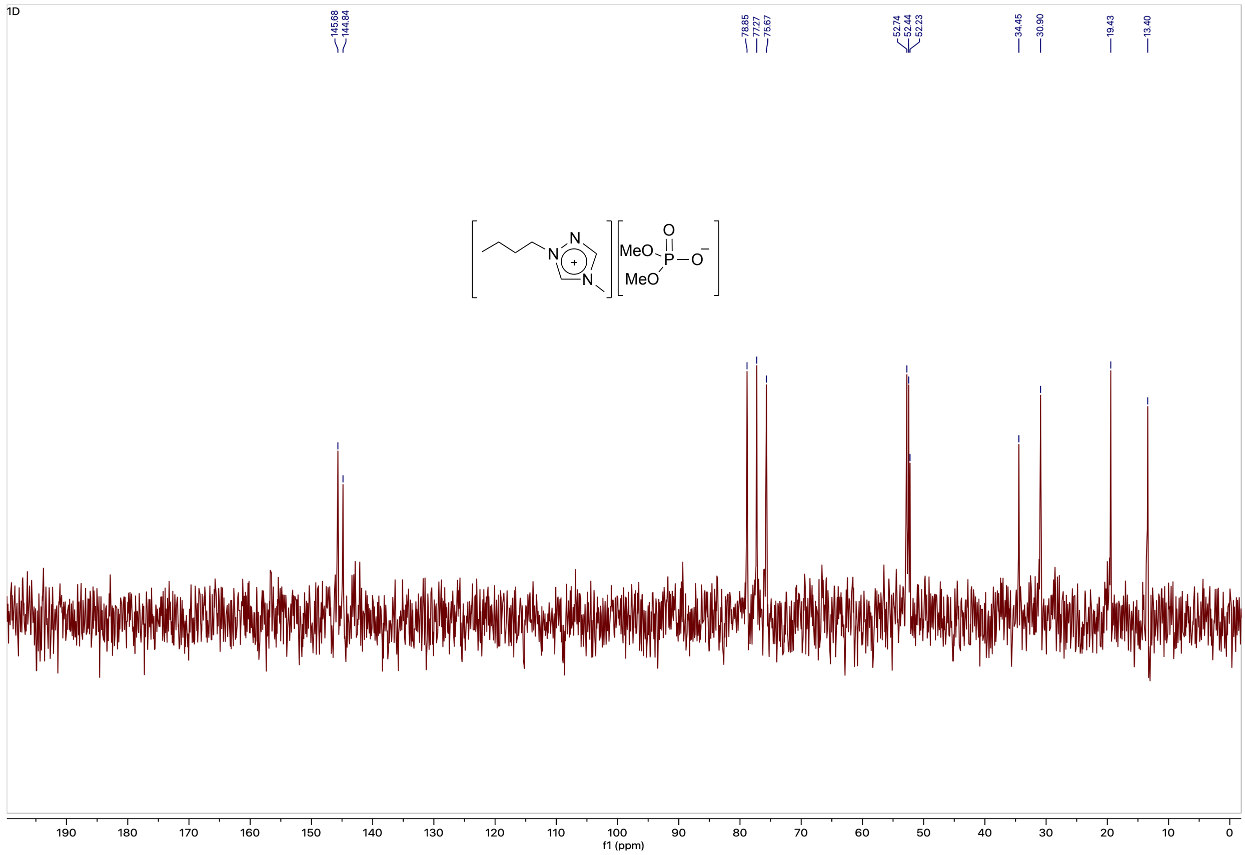

Figure S11-10. ^{13}C NMR of [124-Tz-1,4][DMPO$_4$]. (20 MHz, $CDCl_3$).

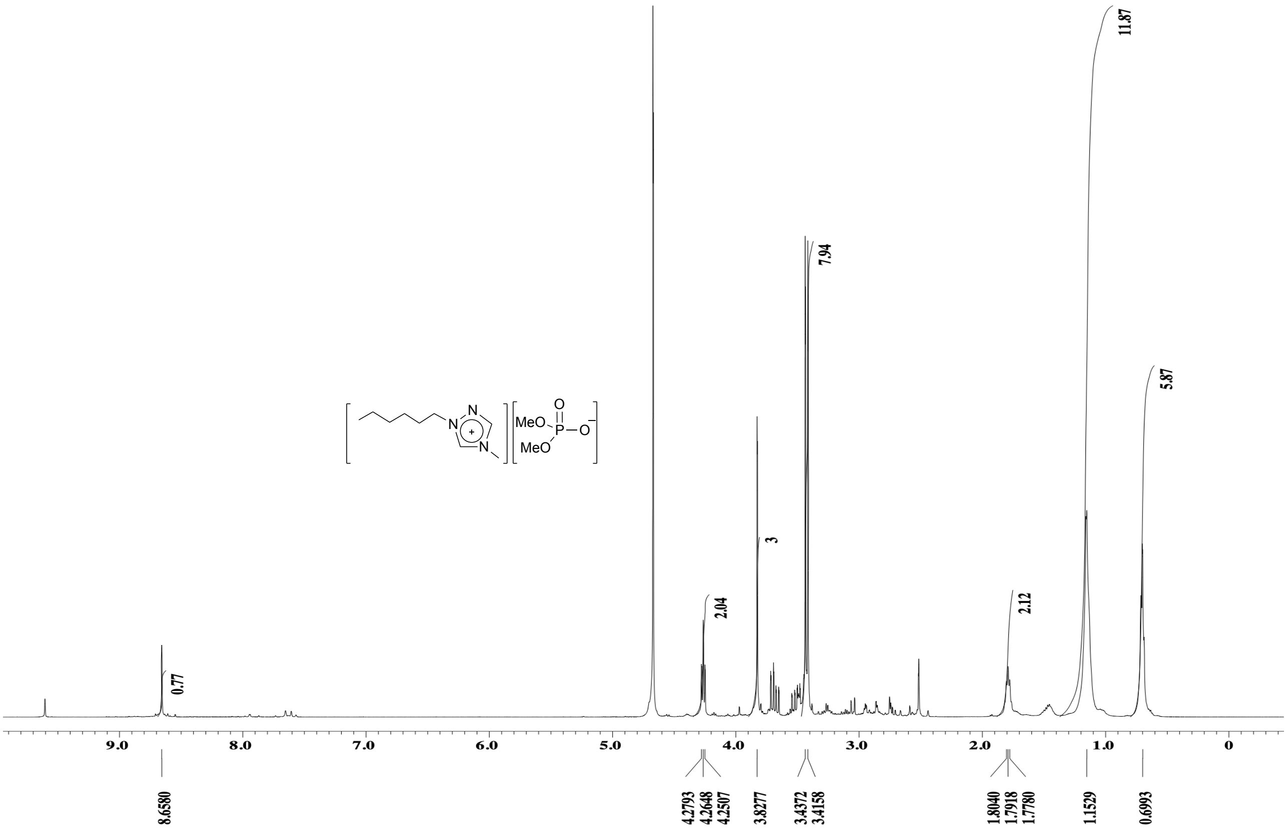

Figure S11-11. ^{1}H NMR of [124-Tz-1,6][DMPO4]. (500 MHz, D_2O).

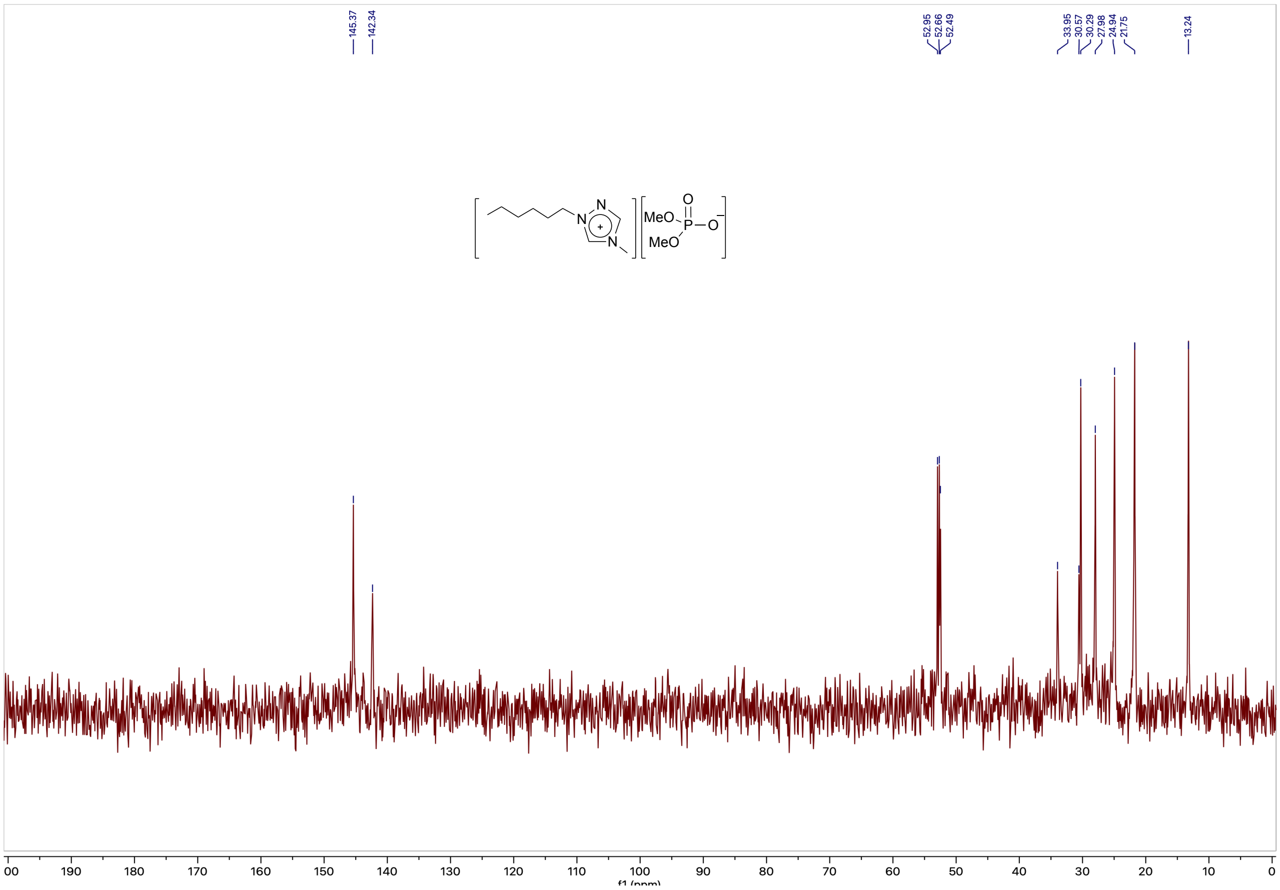

Figure S11-12. ^{13}C NMR of [124-Tz-1,6][DMPO4]. (20 MHz, D_2O).

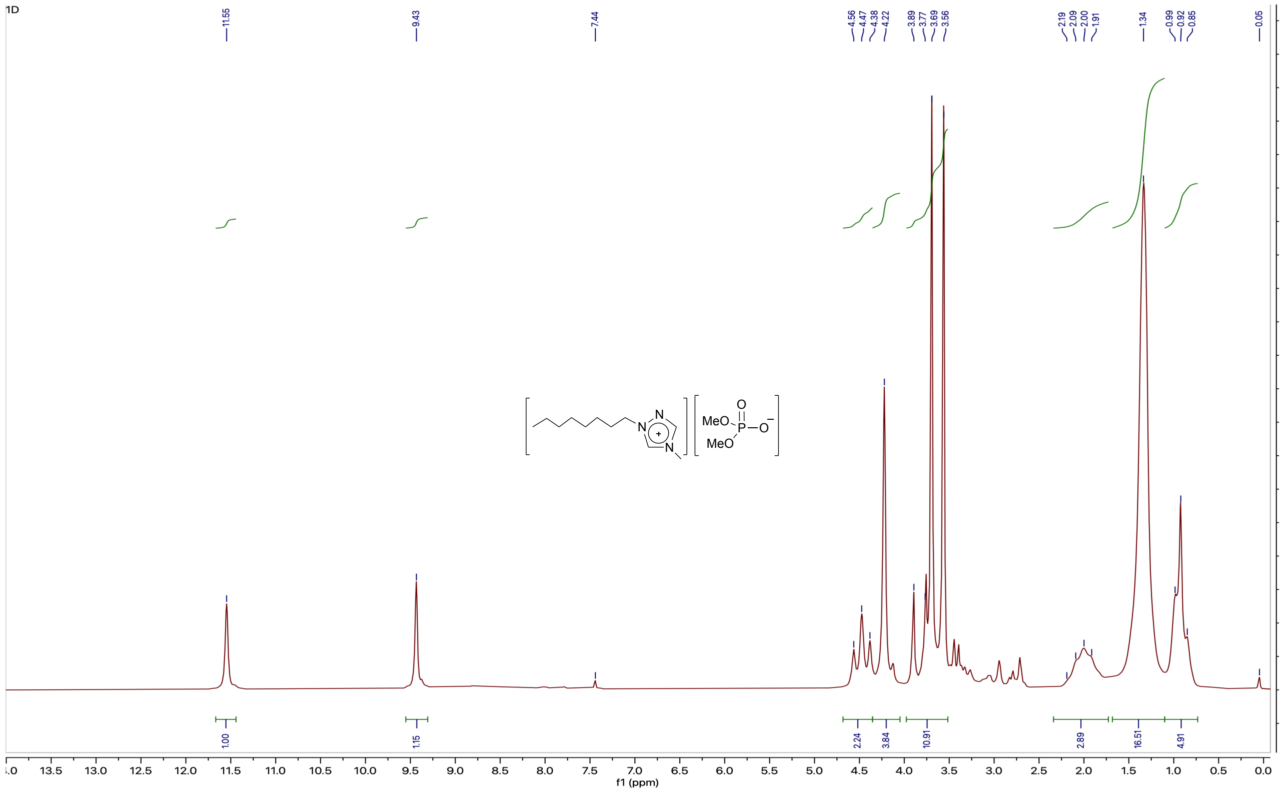

Figure S11-13. ^{1}H NMR of [124-Tz-1,8][DMPO4]. (80 MHz, $CDCl_3$).

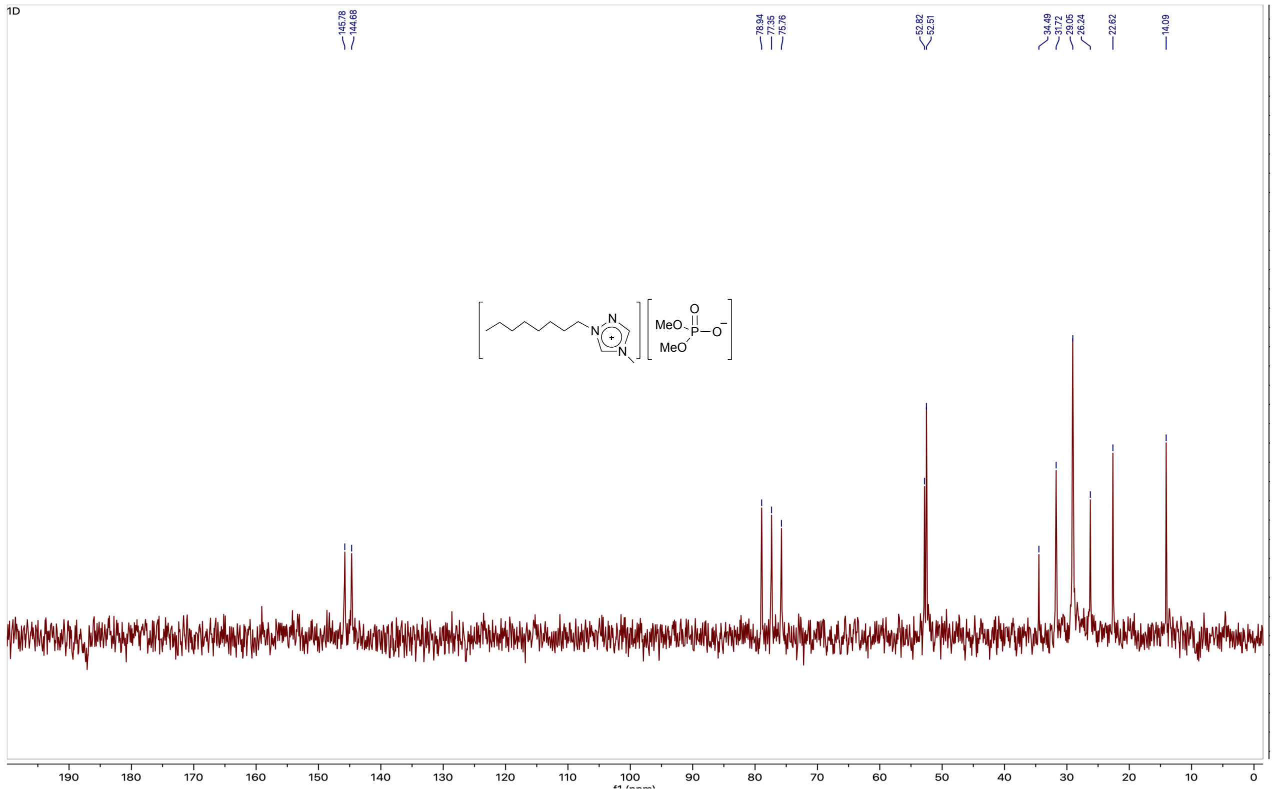

Figure S11-14. ^{13}C NMR of [124-Tz-1,8][DMPO4]. (20 MHz, $CDCl_3$).

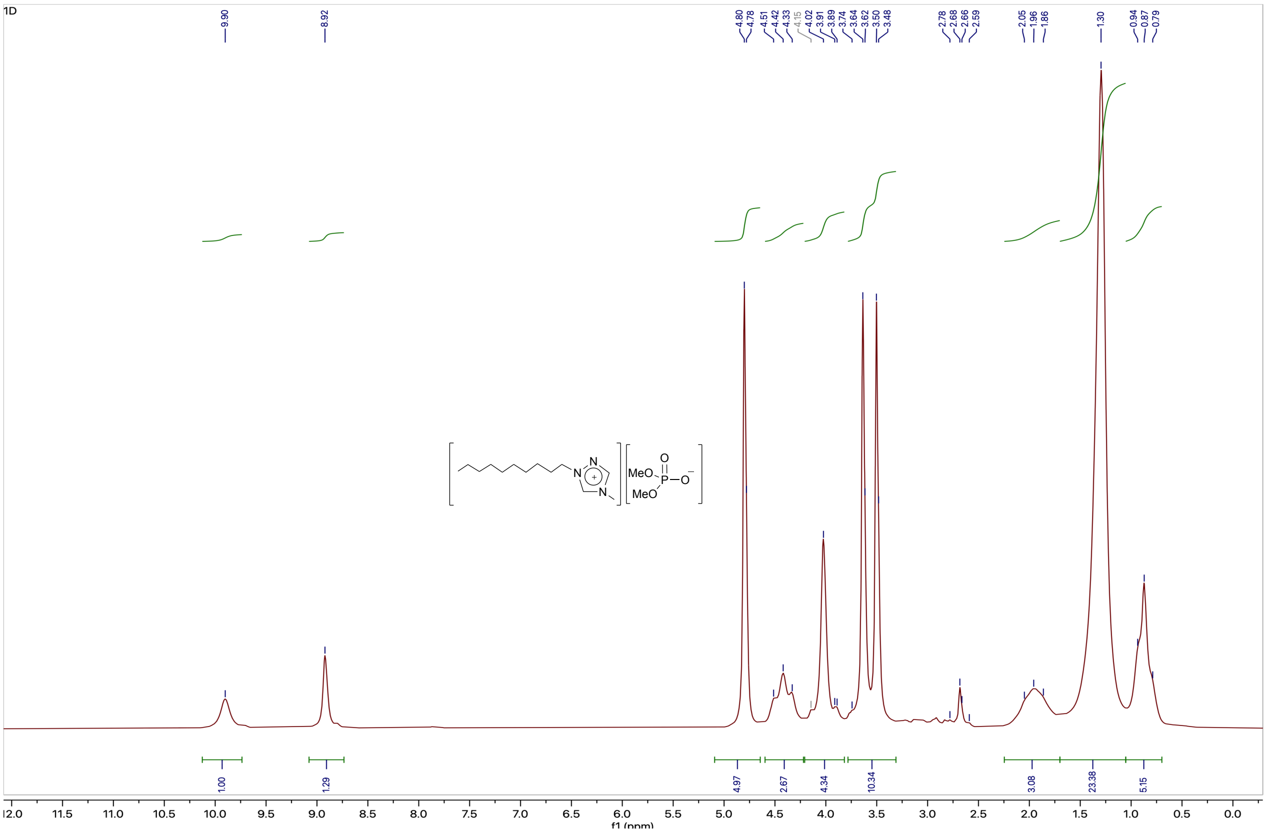

Figure S11-15. ^{1}H NMR of [124-Tz-1,10][DMPO$_4$]. (80 MHz, D_2O).

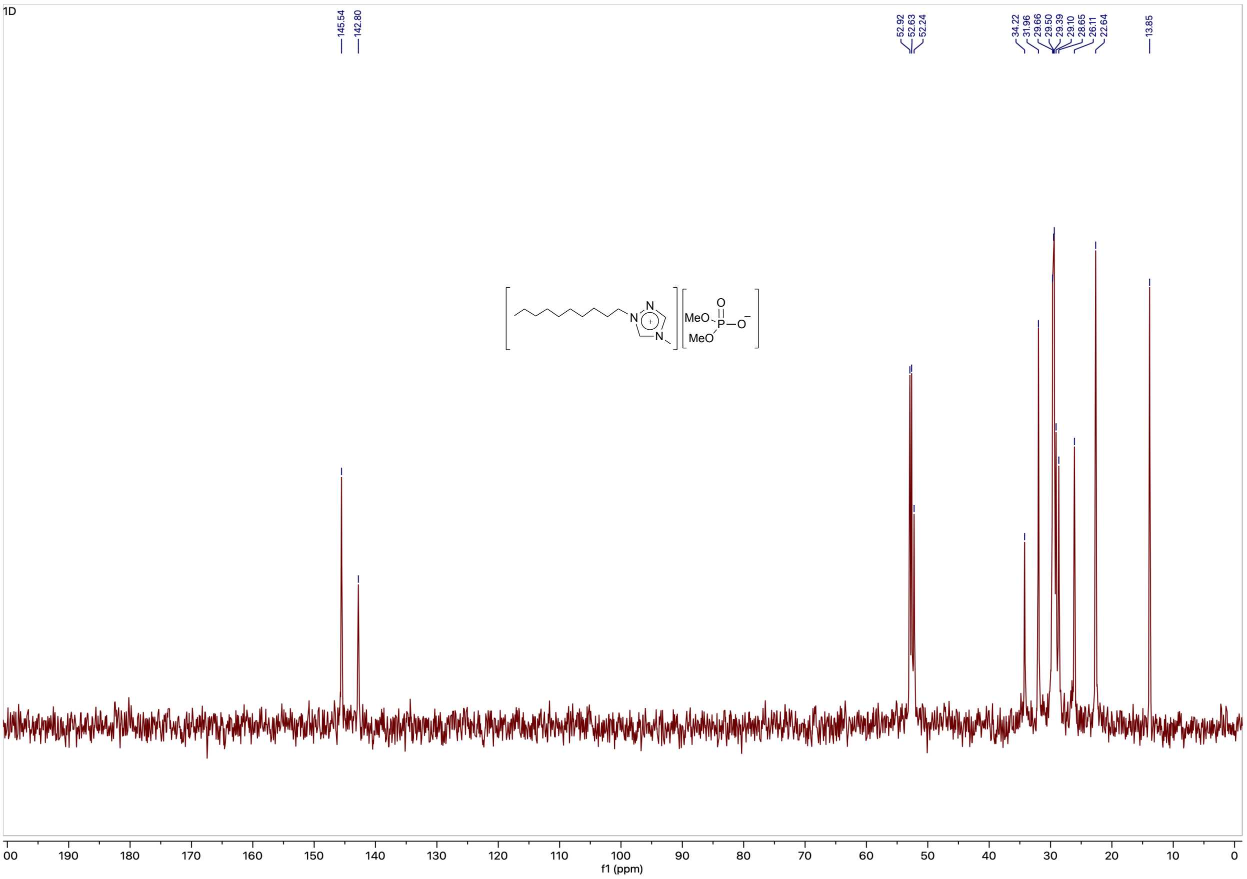

Figure S11-16. ^{13}C NMR of [124-Tz-1,10][DMPO$_4$]. (20 MHz, D_2O).

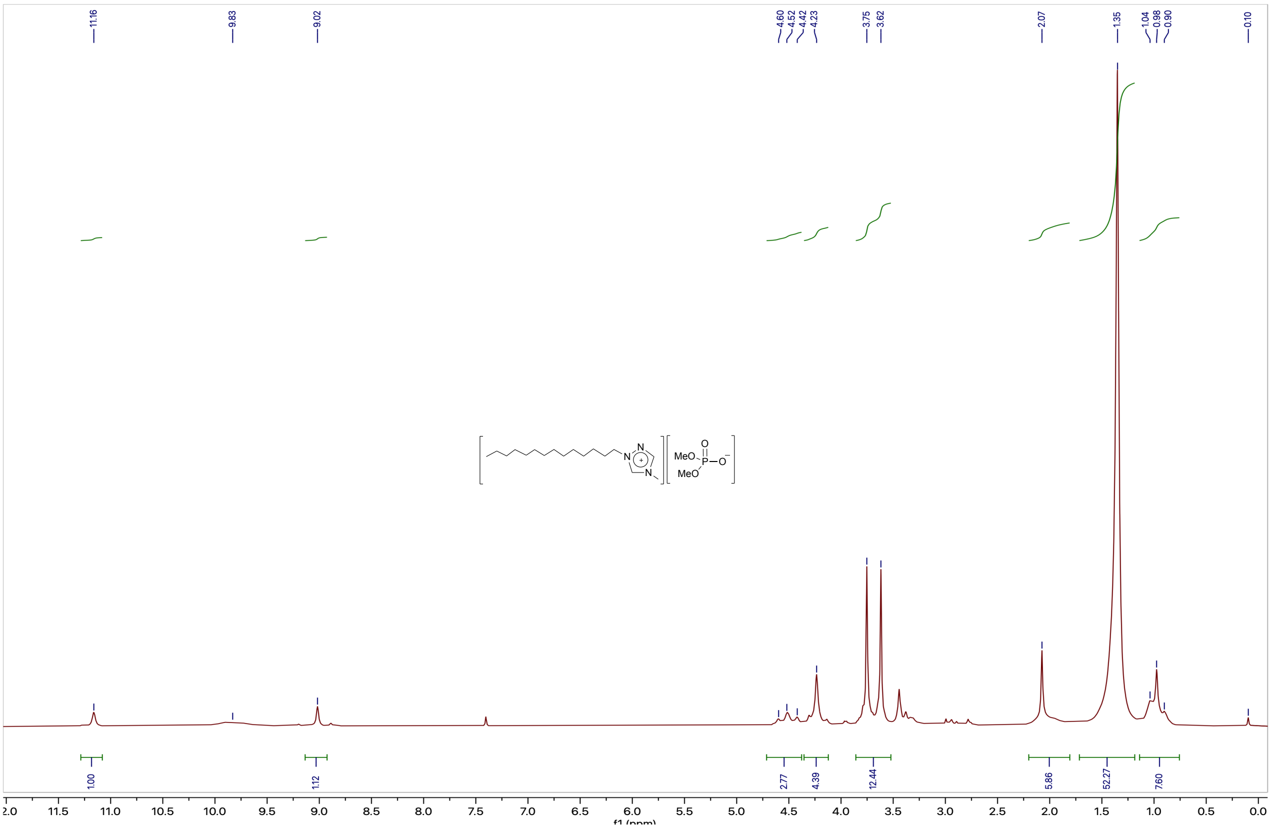

Figure S11-17. ^{1}H NMR of [124-Tz-1,14][DMPO$_4$]. (80 MHz, $CDCl_3$).

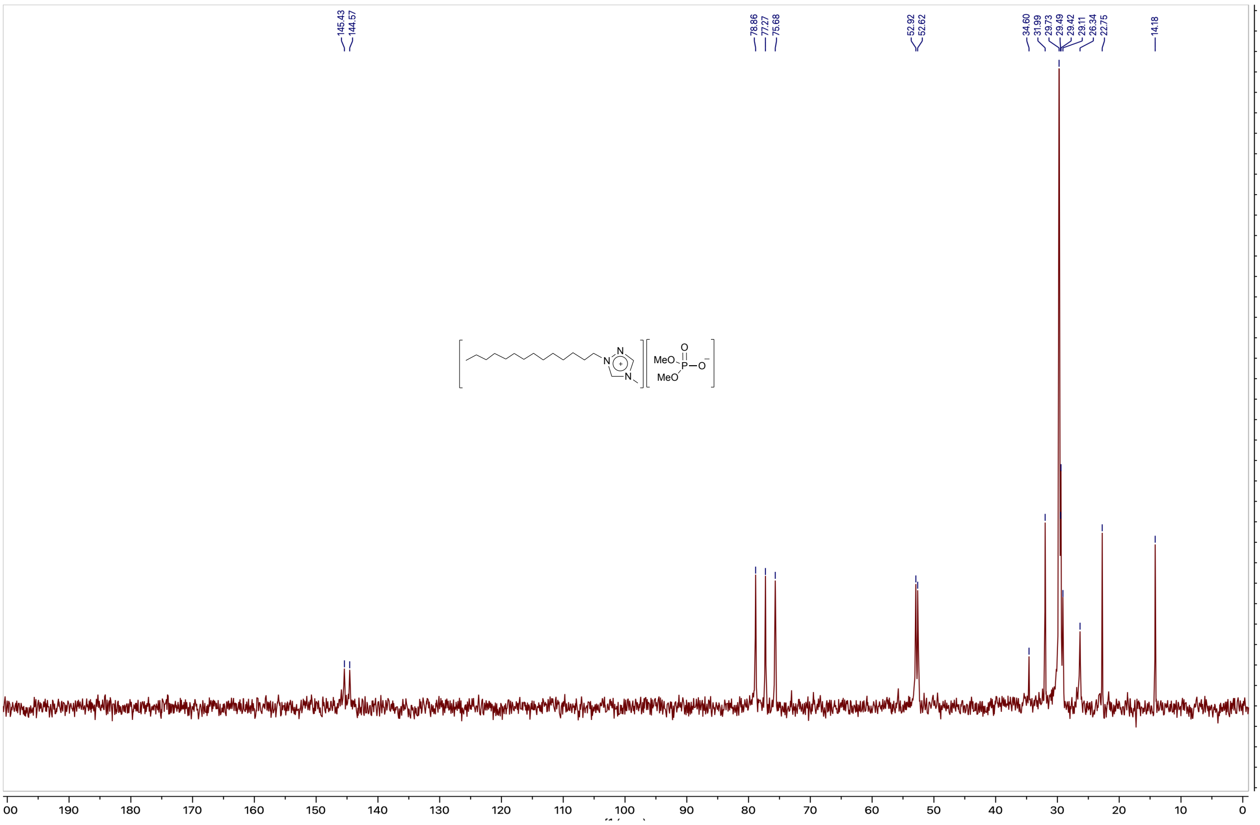

Figure S11-18. ^{13}C NMR of [124-Tz-1,14][DMPO$_4$]. (20 MHz, $CDCl_3$).

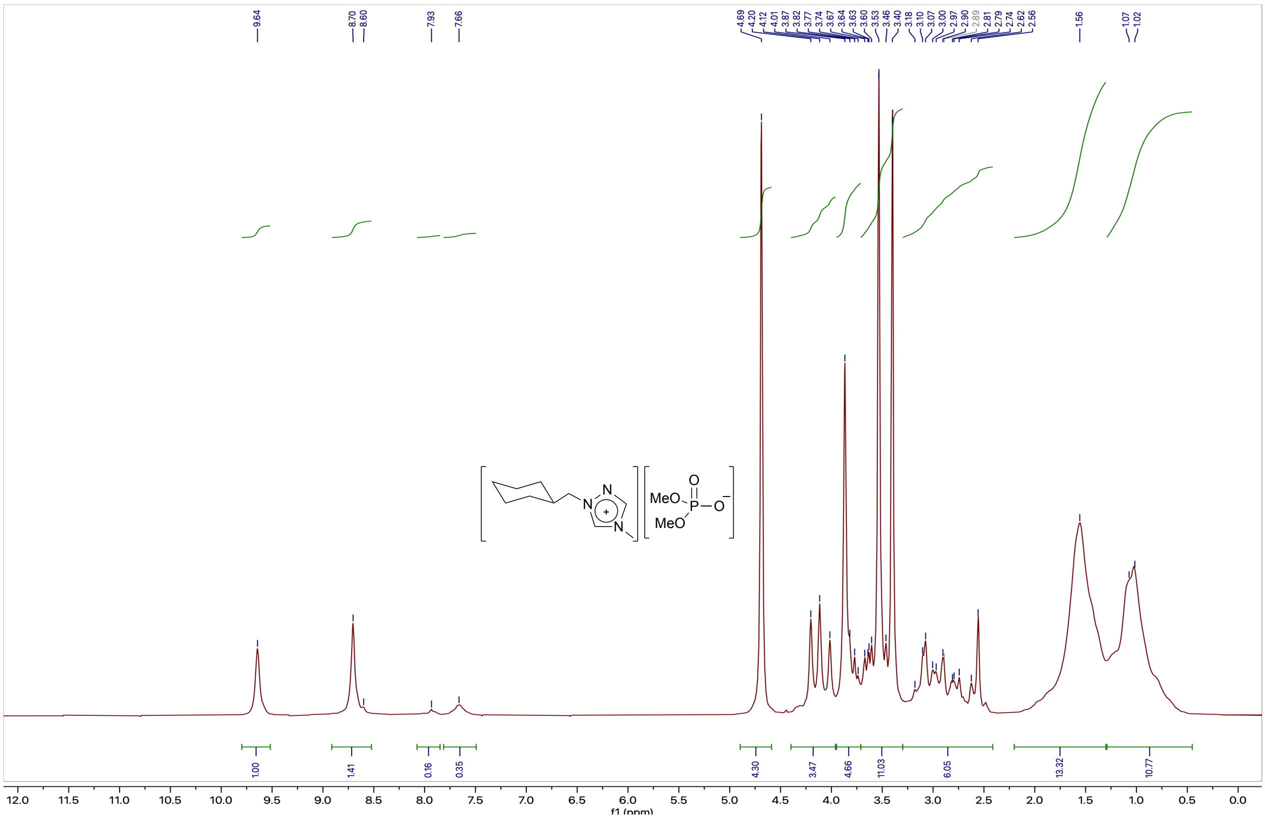

Figure S11-19. ^{1}H NMR of [124-Tz-1,c6][DMPO$_4$]. (80 MHz, D$_2$O).

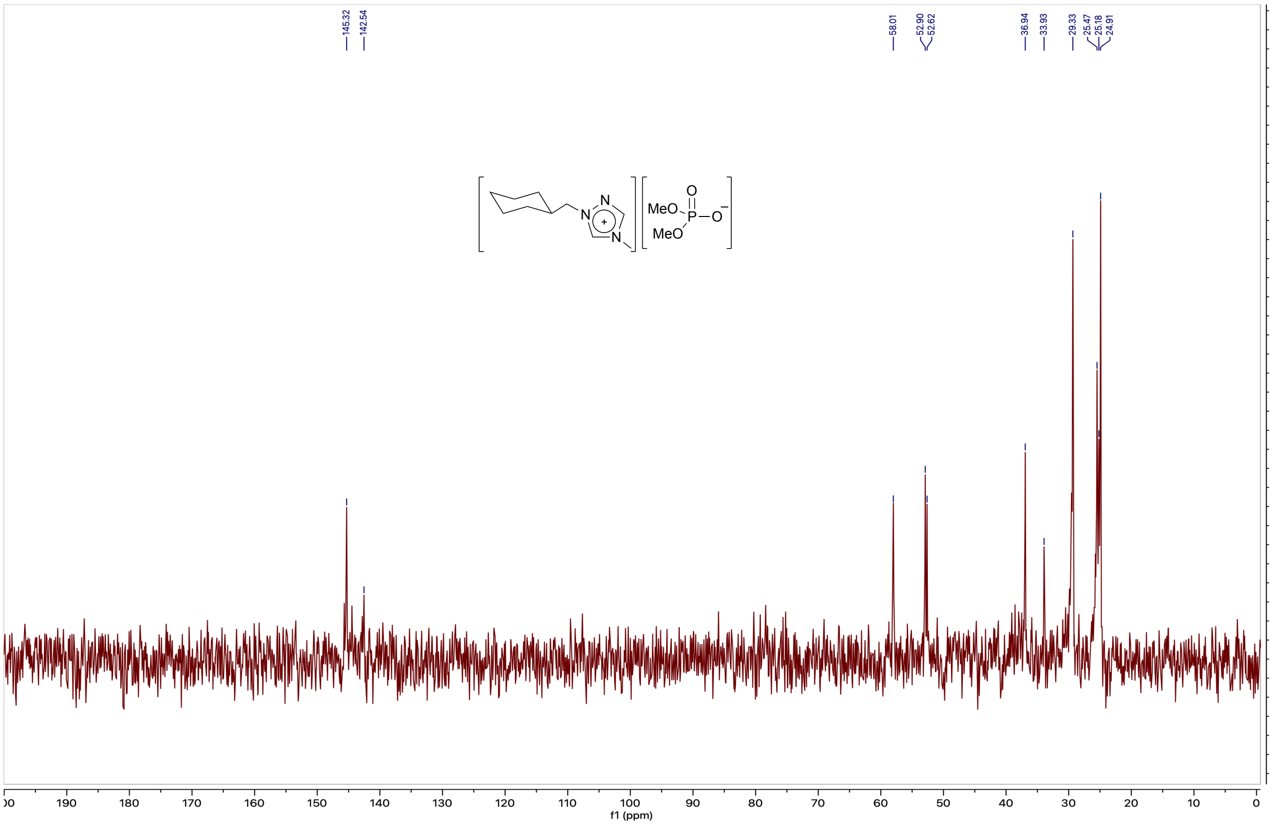

Figure S11-20. ^{13}C NMR of [124-Tz-1,c6][DMPO$_4$]. (20 MHz, D$_2$O).

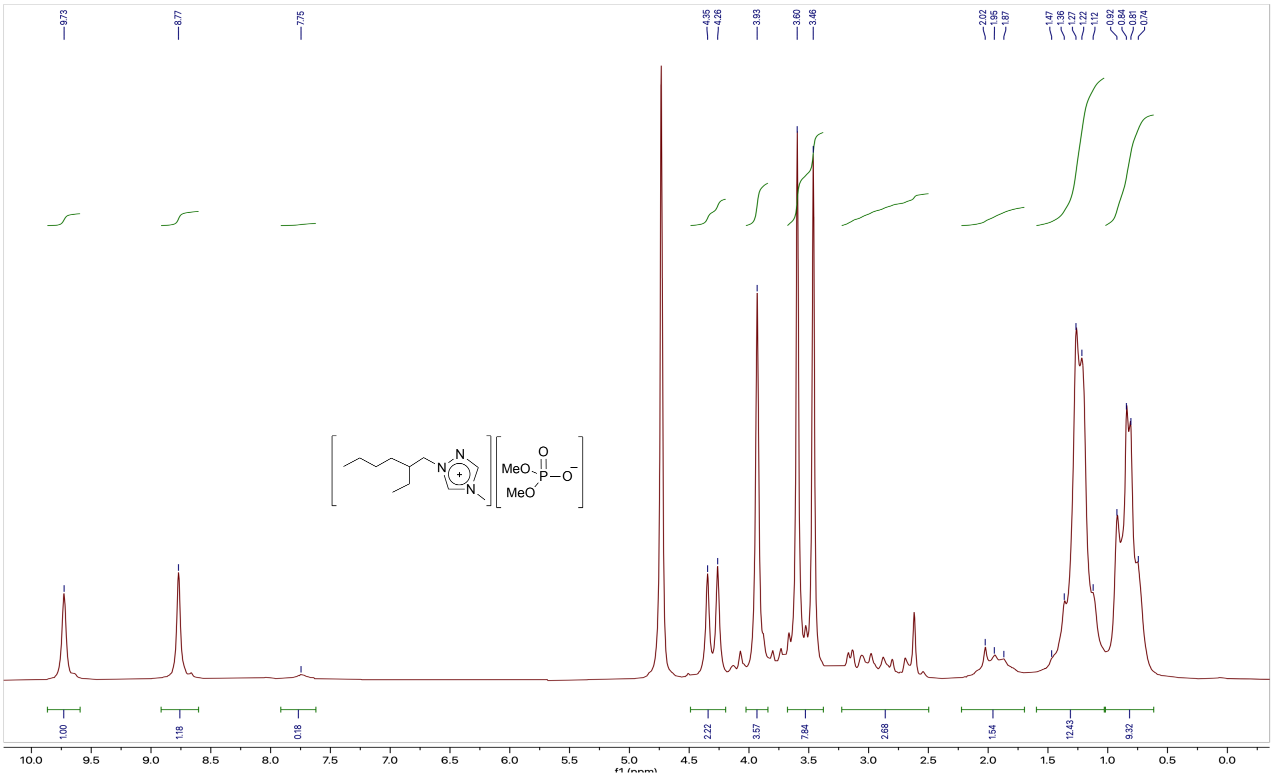

Figure S11-21. ^{1}H NMR of [124-Tz-1,(2-Et)6][DMPO$_4$]. (80 MHz, D_2O).

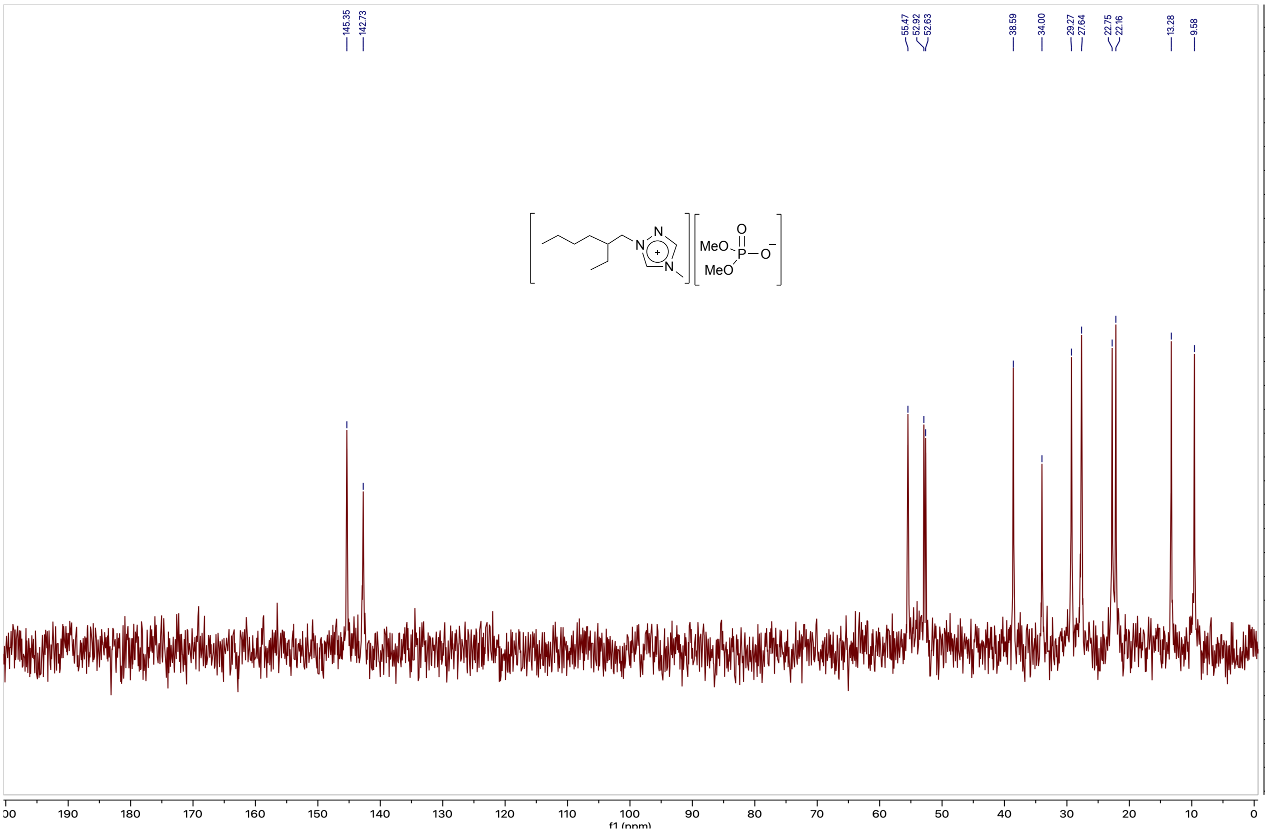

Figure S11-22. ^{13}C NMR of [124-Tz-1,(2-Et)6][DMPO$_4$]. (20 MHz, D_2O).

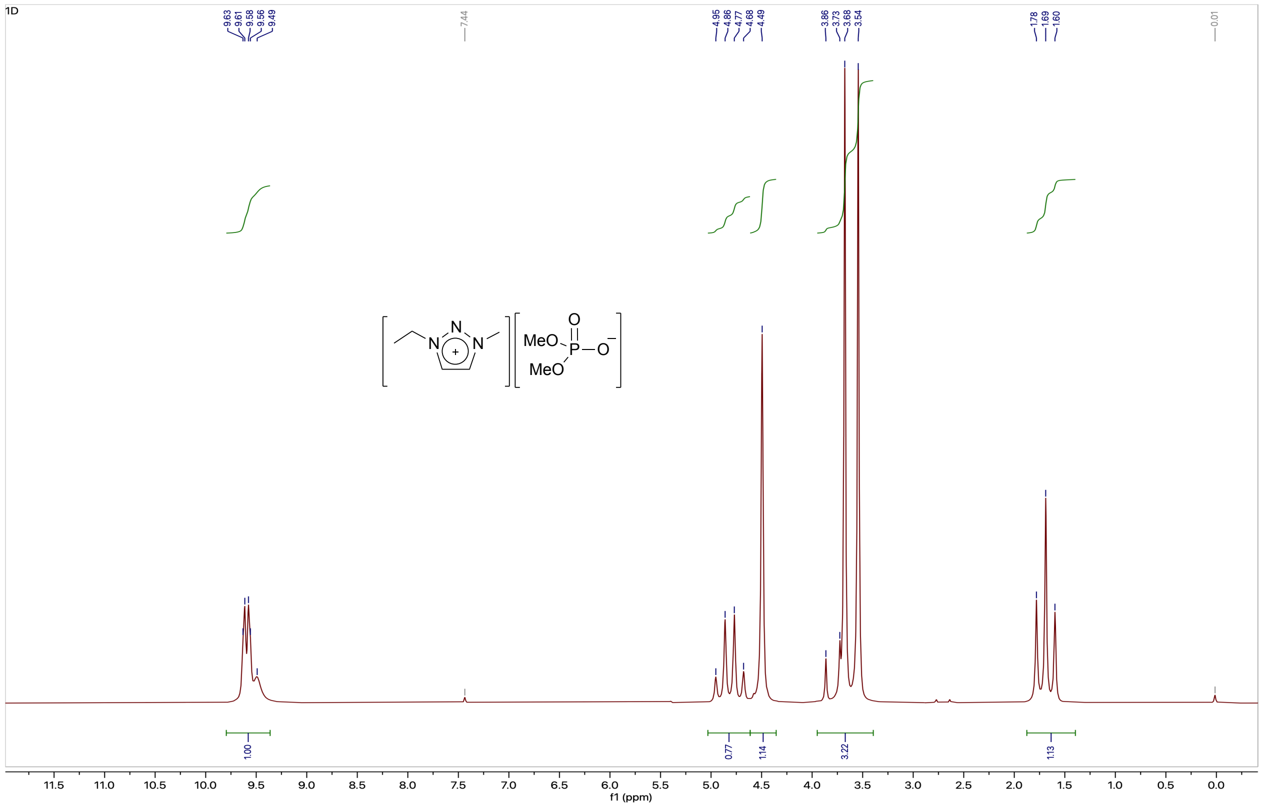

Figure S11-23. ^{1}H NMR of [123-Tz-1,2][DMPO$_4$]. (80 MHz, $CDCl_3$).

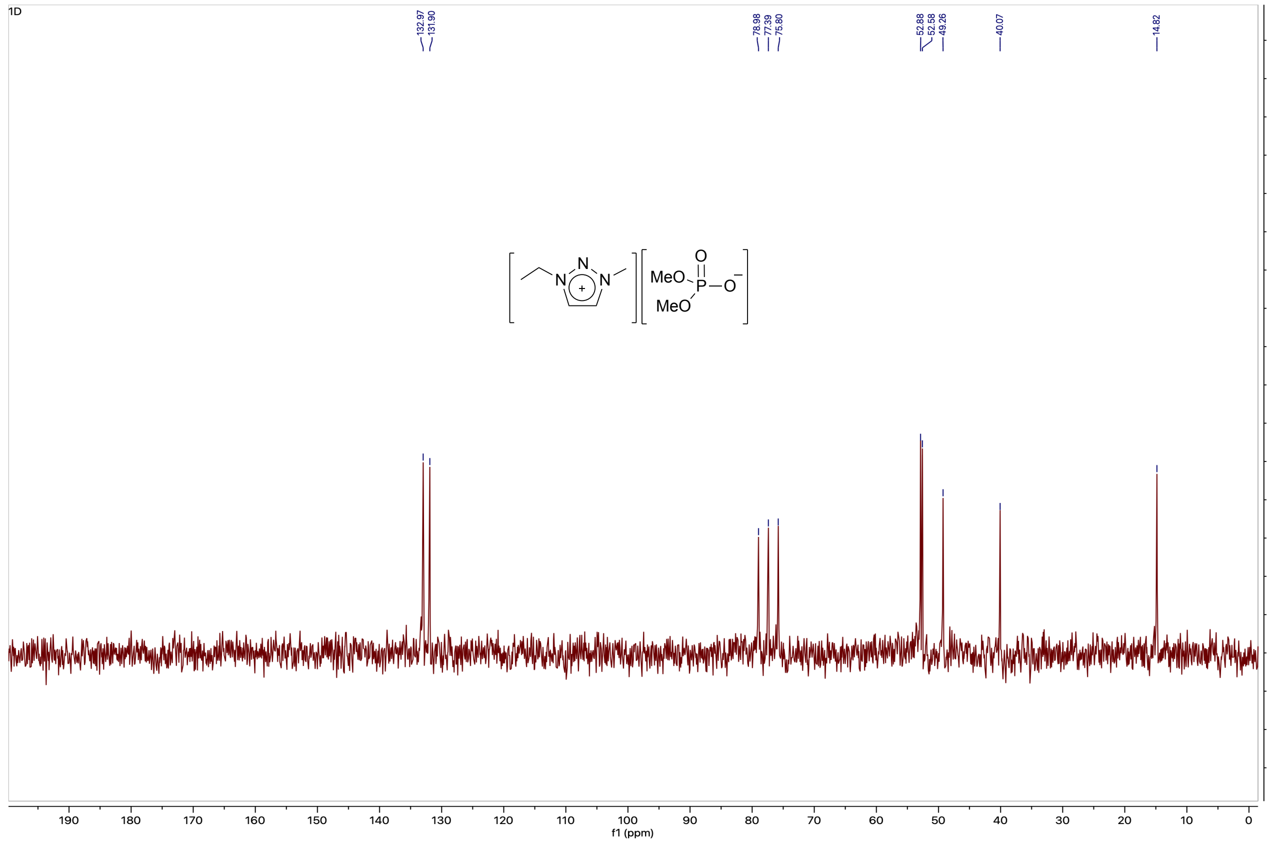

Figure S11-24. ^{13}C NMR of [123-Tz-1,2][DMPO$_4$] . (20 MHz, $CDCl_3$).

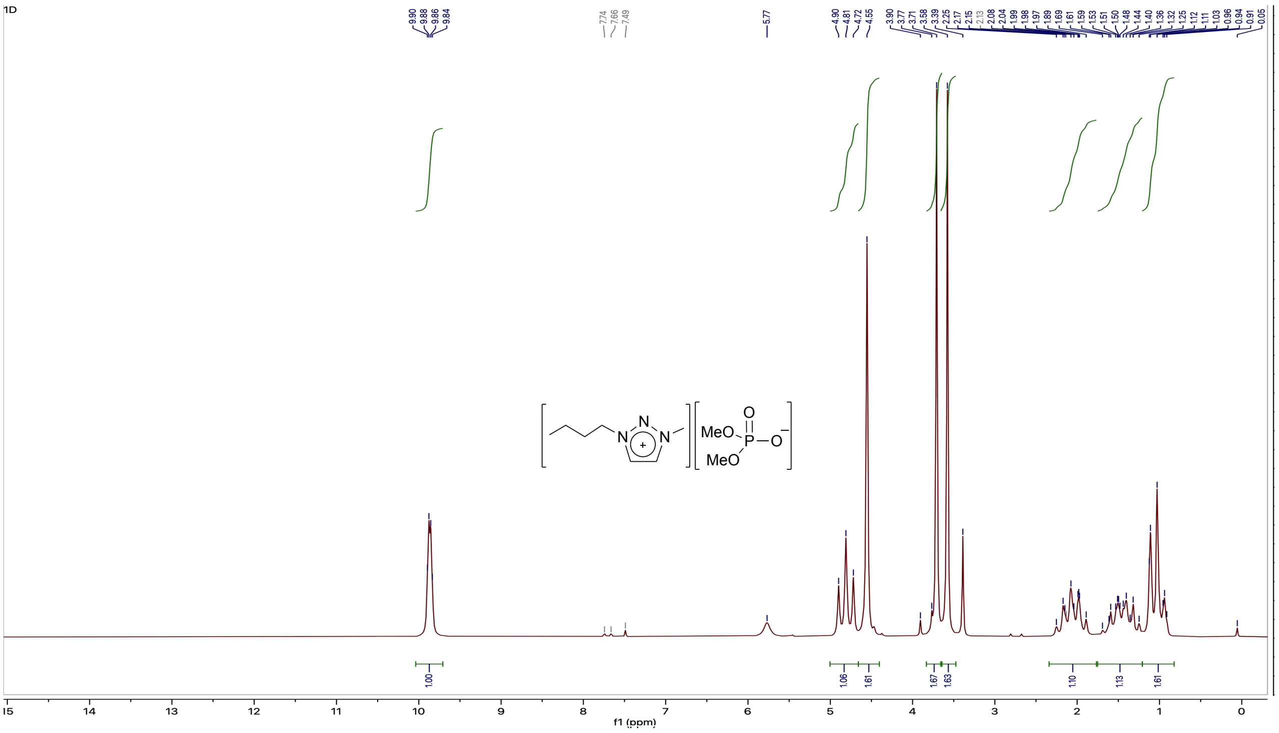

Figure S11-25. ^{1}H NMR of [123-Tz-1,4][DMPO$_4$]. (80 MHz, CDCl$_3$).

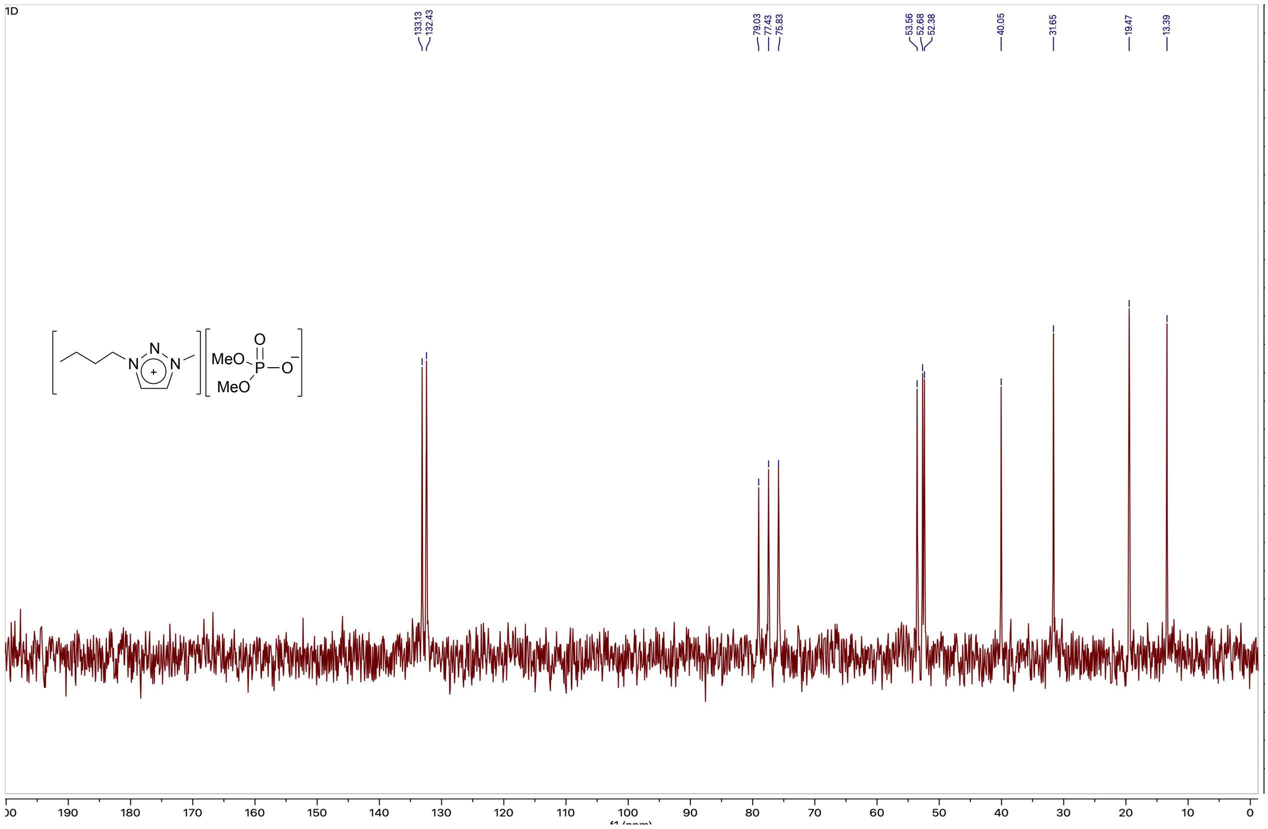

Figure S11-26. ^{13}C NMR of [123-Tz-1,4][DMPO$_4$]. (20 MHz, CDCl$_3$).

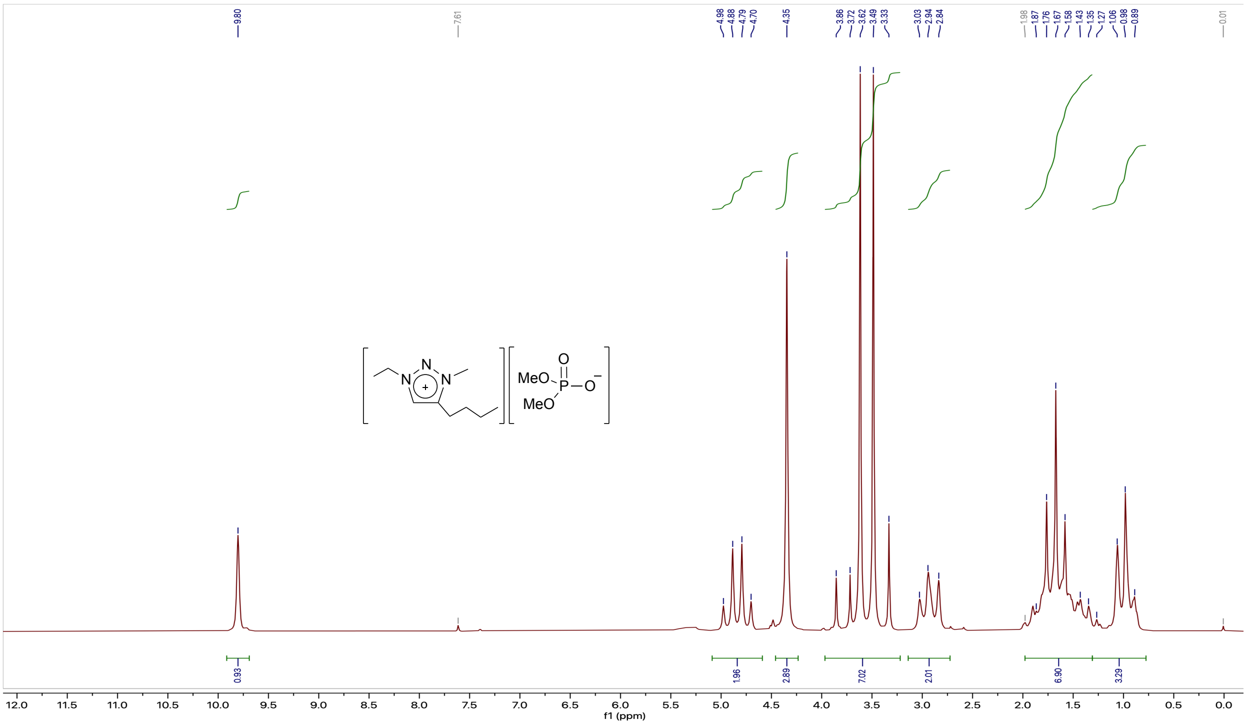

Figure S11-27. ^{1}H NMR of [123-Tz-1,2,4][DMPO$_4$]. (80 MHz, $CDCl_3$).

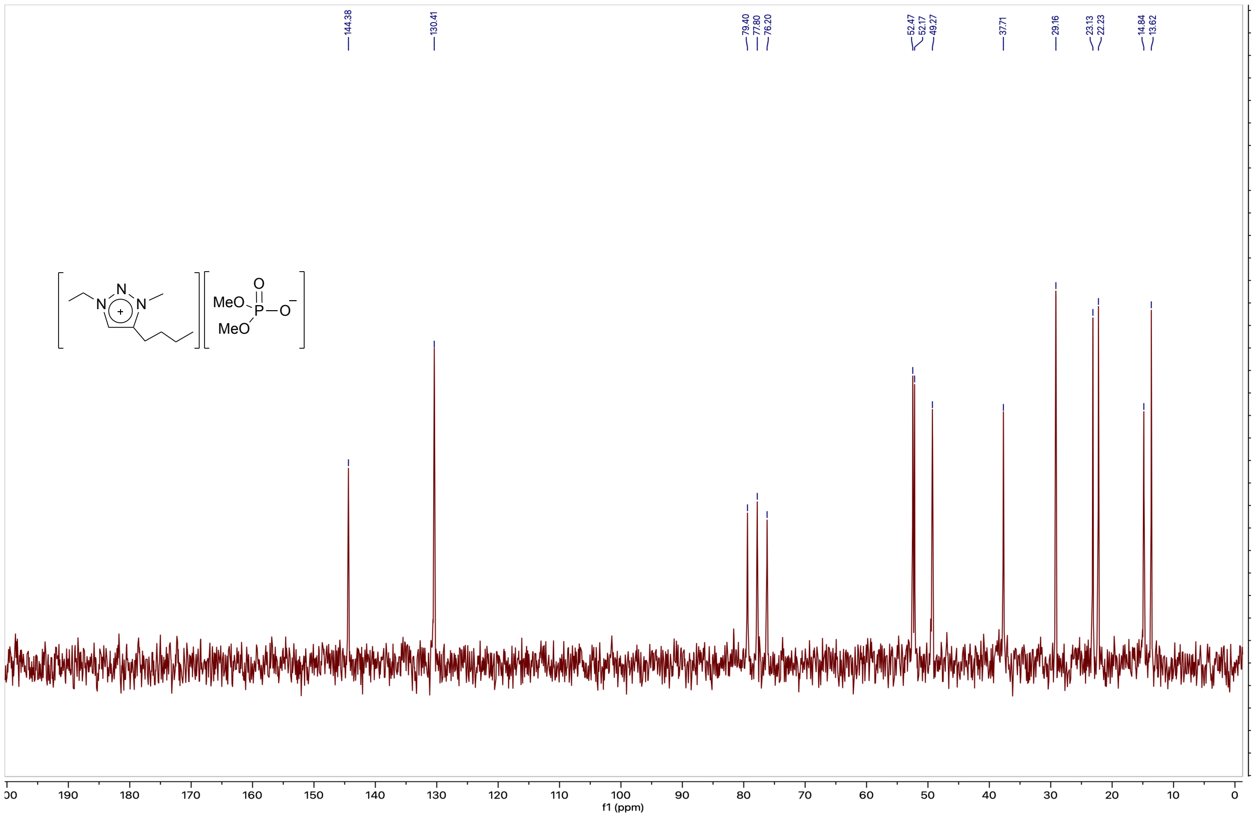

Figure S11-28. ^{13}C NMR of [123-Tz-1,2,4][DMPO$_4$]. (20 MHz, $CDCl_3$).

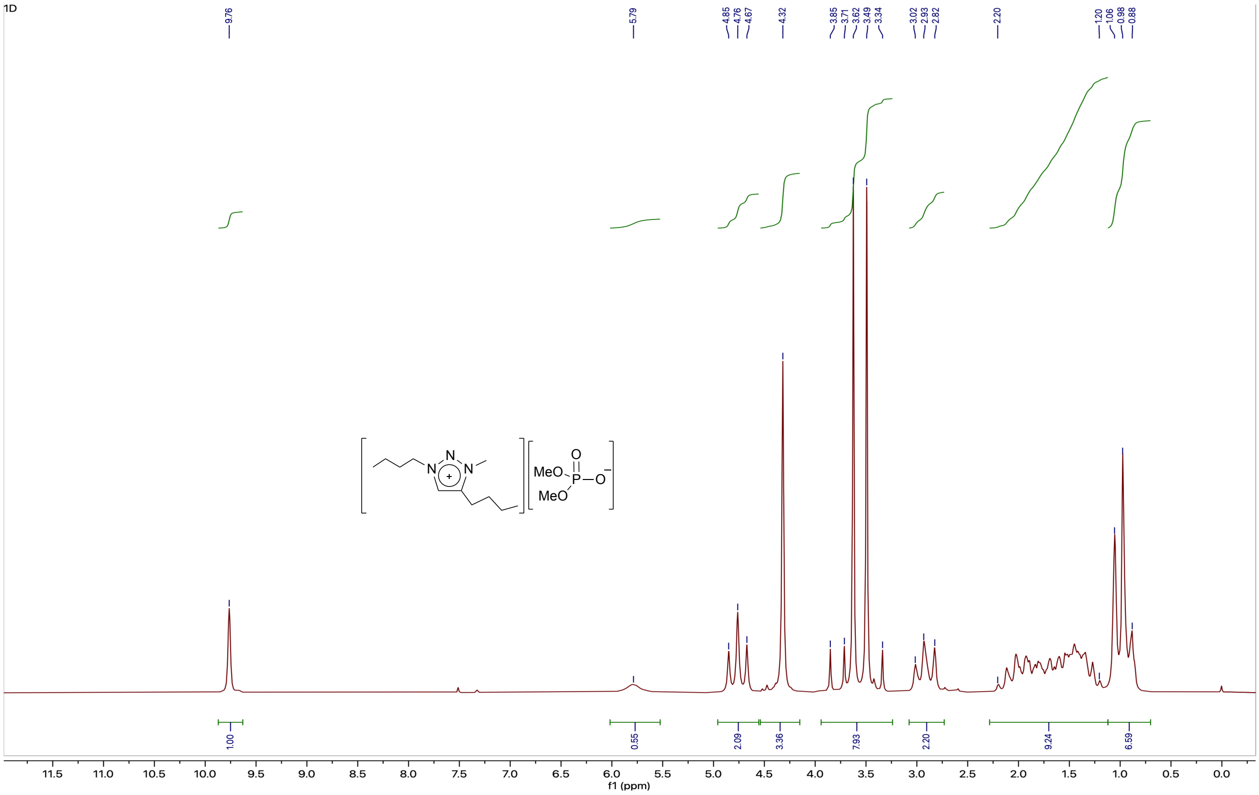

Figure S11-29. ^{1}H NMR of [123-Tz-1,4,4][DMPO$_4$]. (80 MHz, $CDCl_3$).

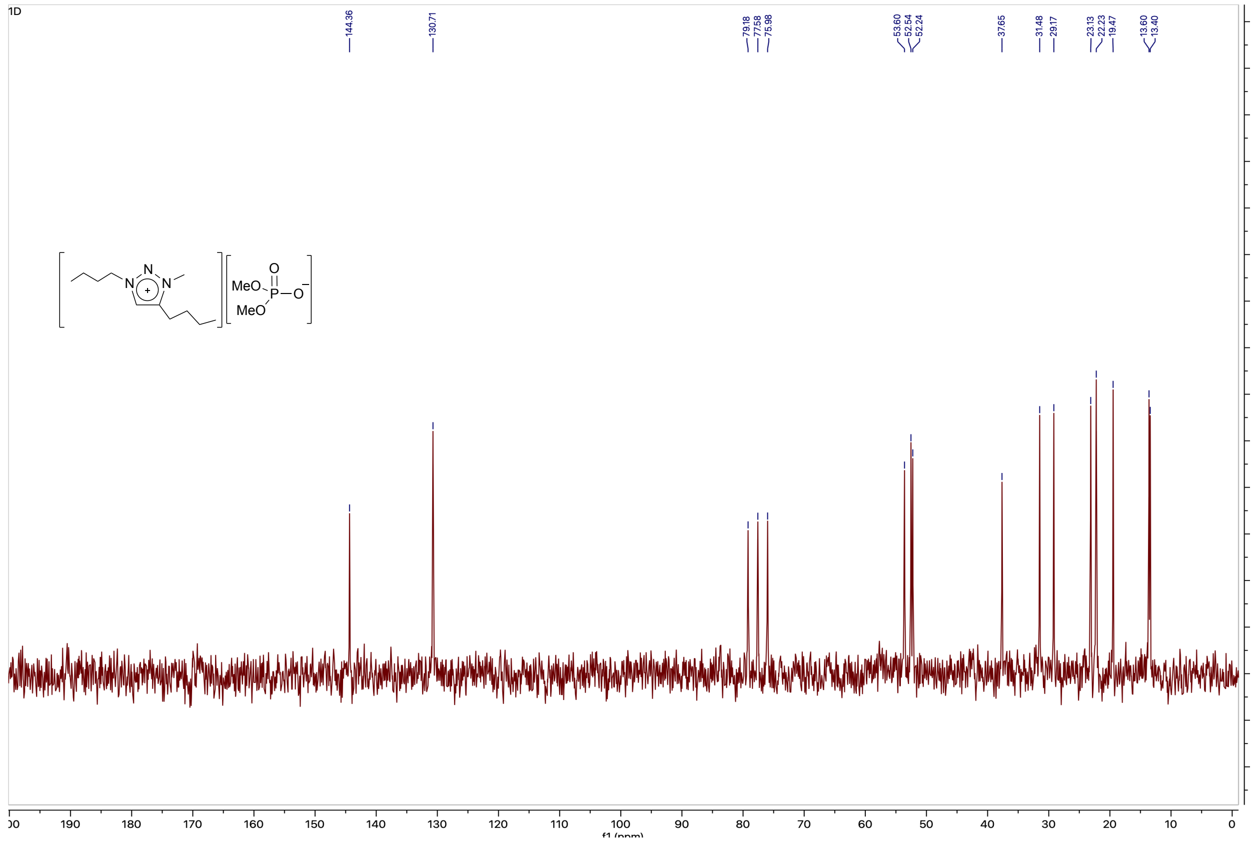

Figure S11-30. ^{13}C NMR of [123-Tz-1,4,4][DMPO$_4$]. (20 MHz, $CDCl_3$).

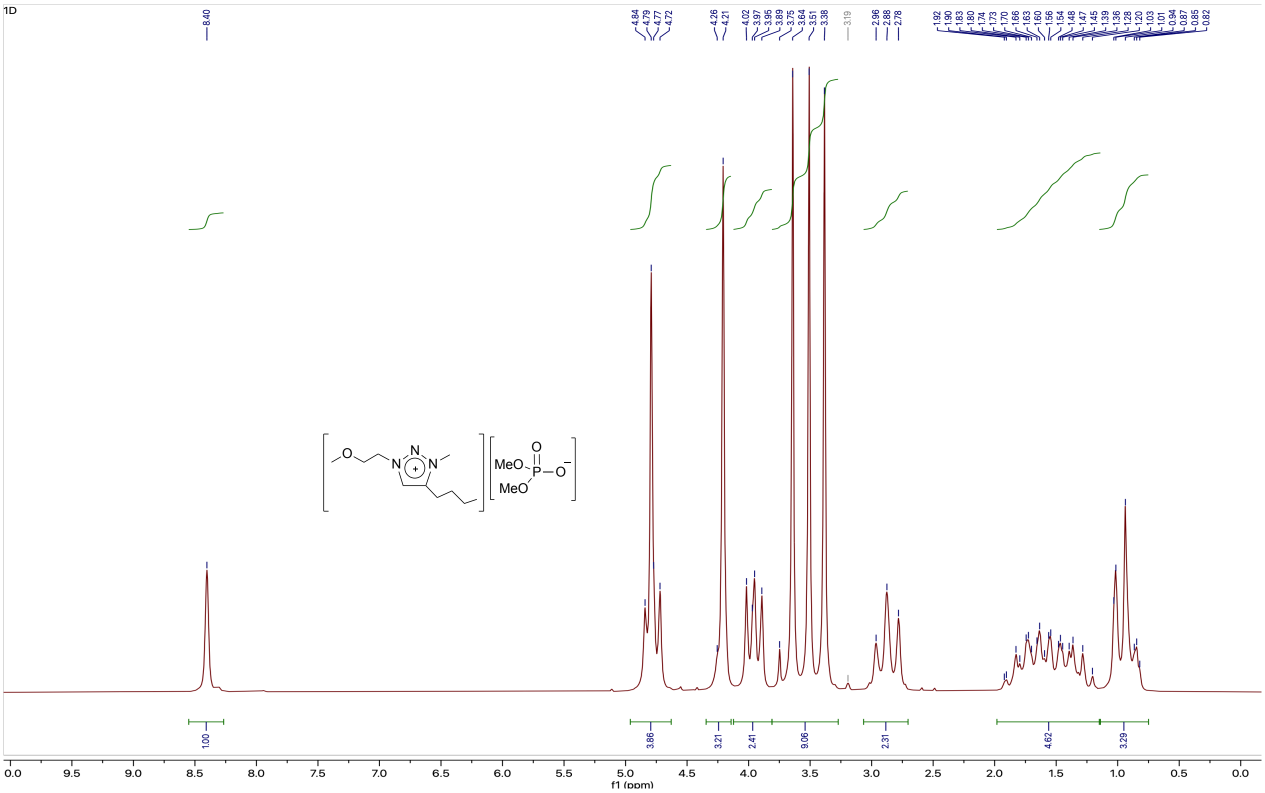

Figure S11-31. ^{1}H NMR of [123-Tz-1,4,ME][DMPO$_4$]. (80 MHz, D$_2$O).

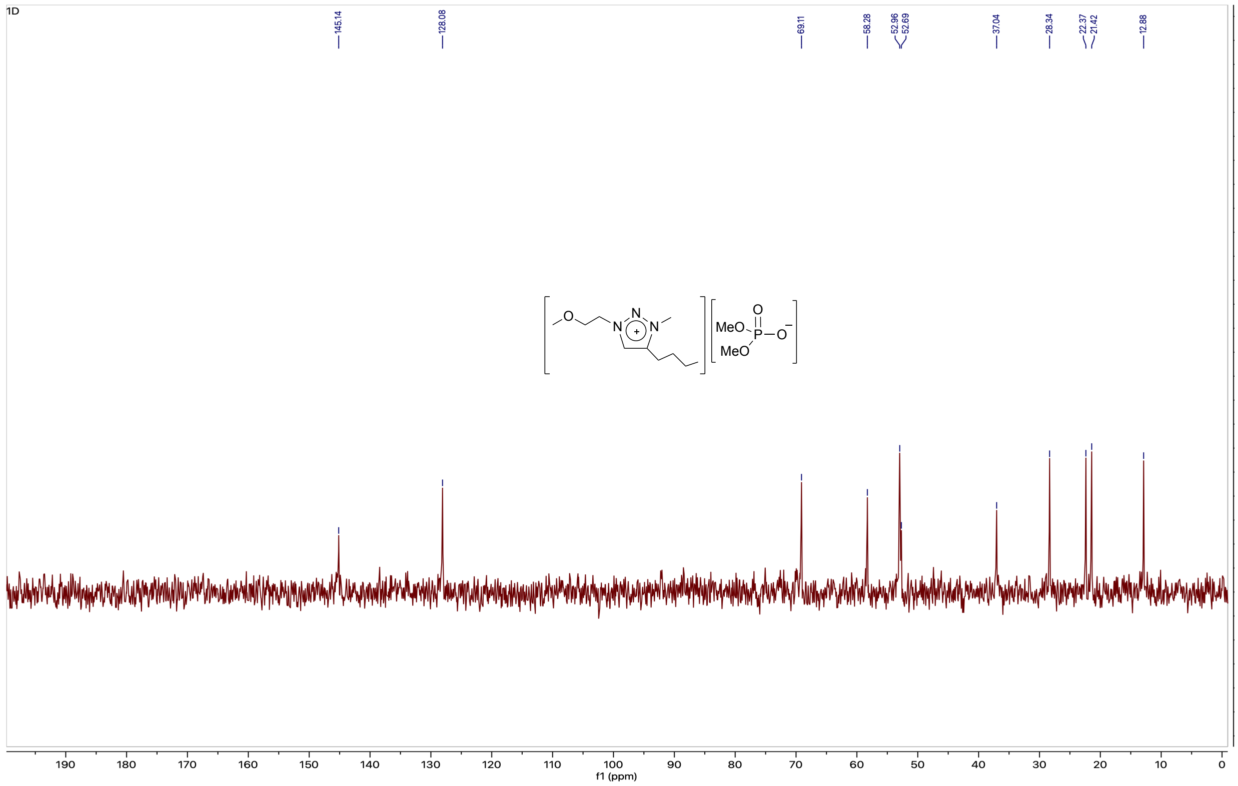

Figure S11-32. ^{13}C NMR of [123-Tz-1,4,ME][DMPO$_4$]. (20 MHz, D$_2$O).

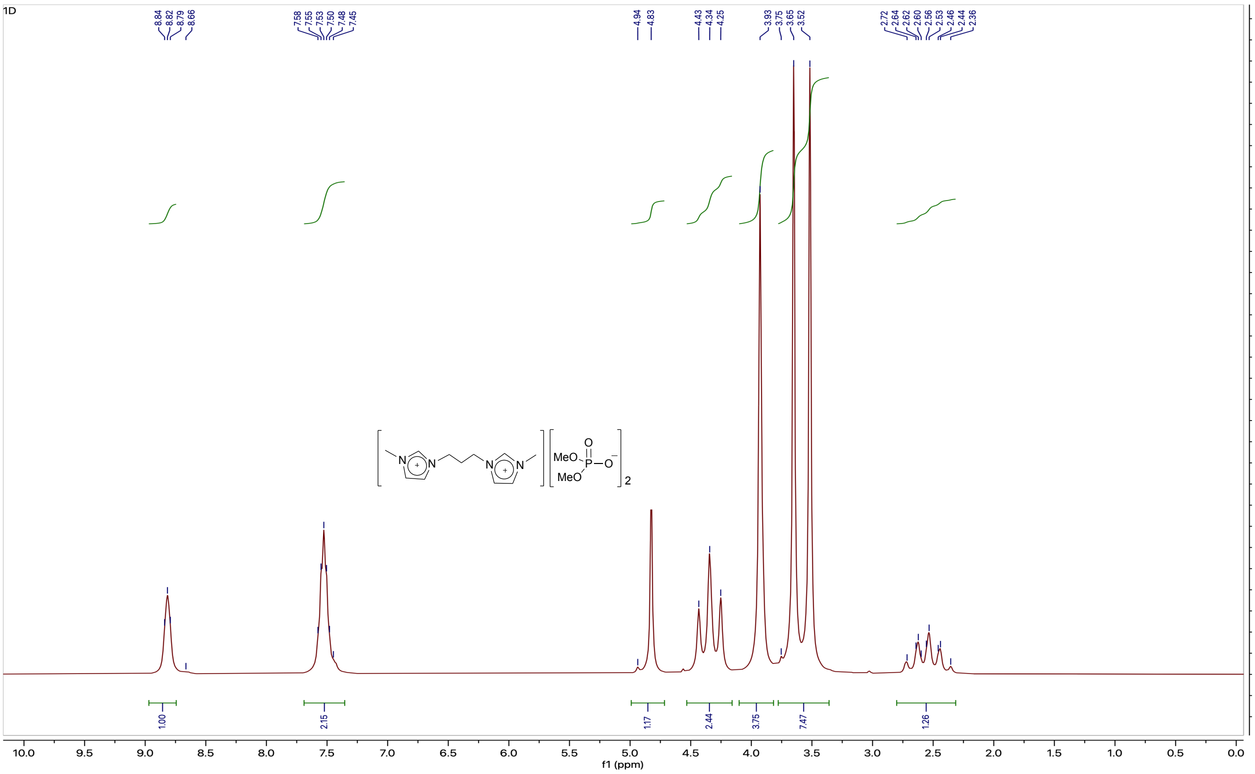

Figure S11-33. ^{1}H NMR of [Bis(MeIm)C3][DMPO$_4$]$_2$. (80 MHz, D$_2$O).

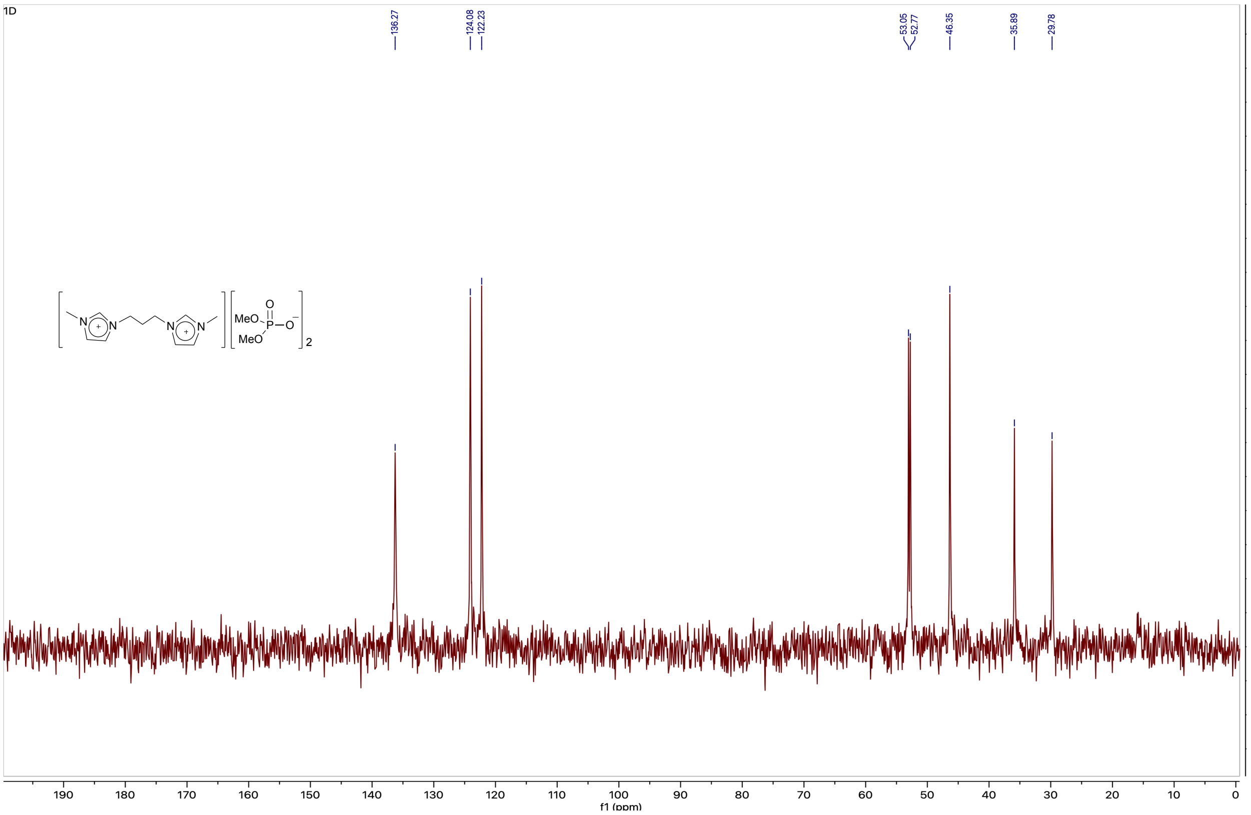

Figure S11-34. ^{13}C NMR of [Bis(MeIm)C3][DMPO$_4$]$_2$. (20 MHz, D$_2$O).

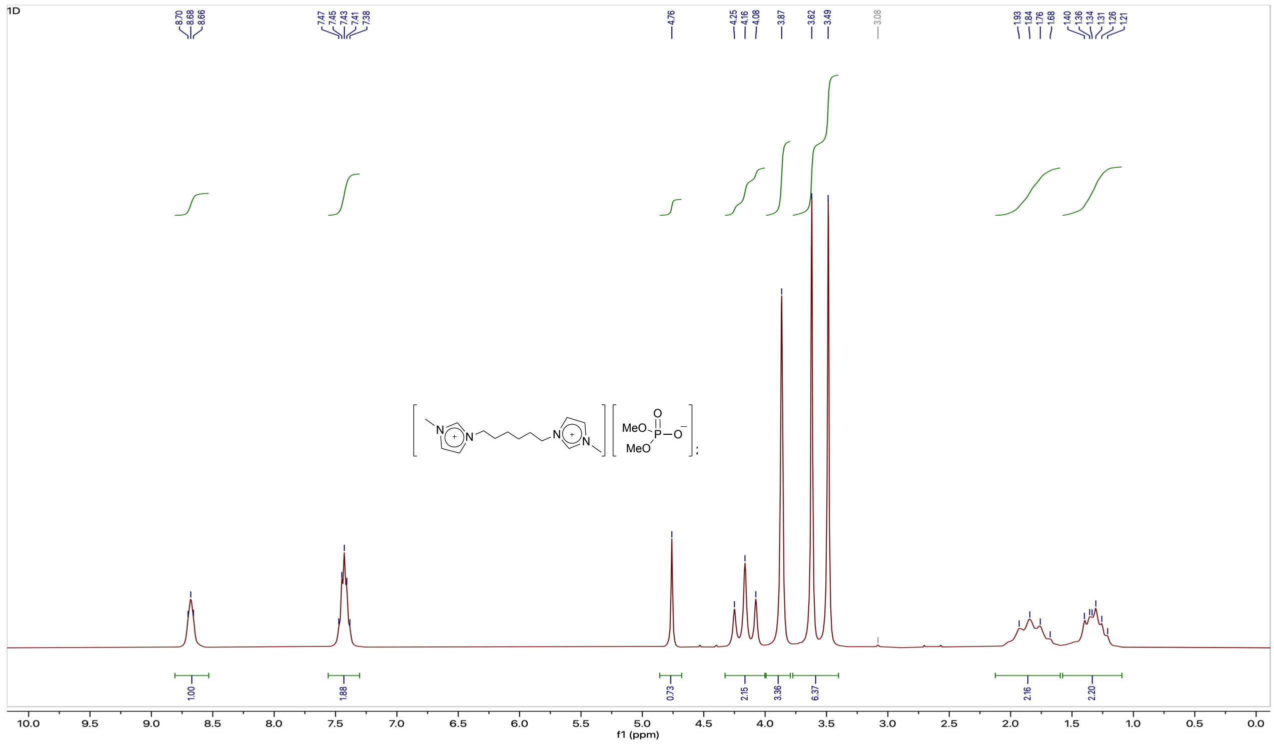

Figure S11-35. ^{1}H NMR of [Bis(MeIm)C6][DMPO$_4$]$_2$. (80 MHz, D_2O).

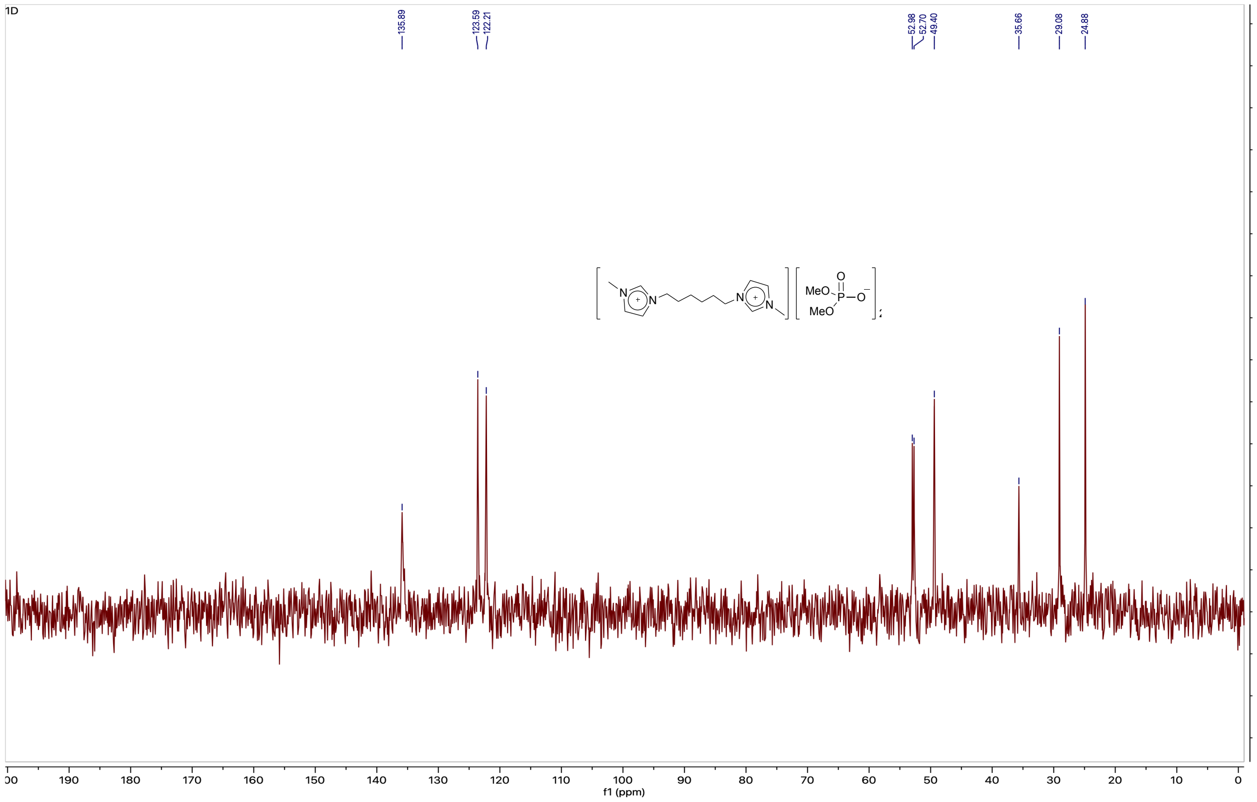

Figure S11-36. ^{13}C NMR of [Bis(MeIm)C6][DMPO$_4$]$_2$. (20 MHz, D_2O).

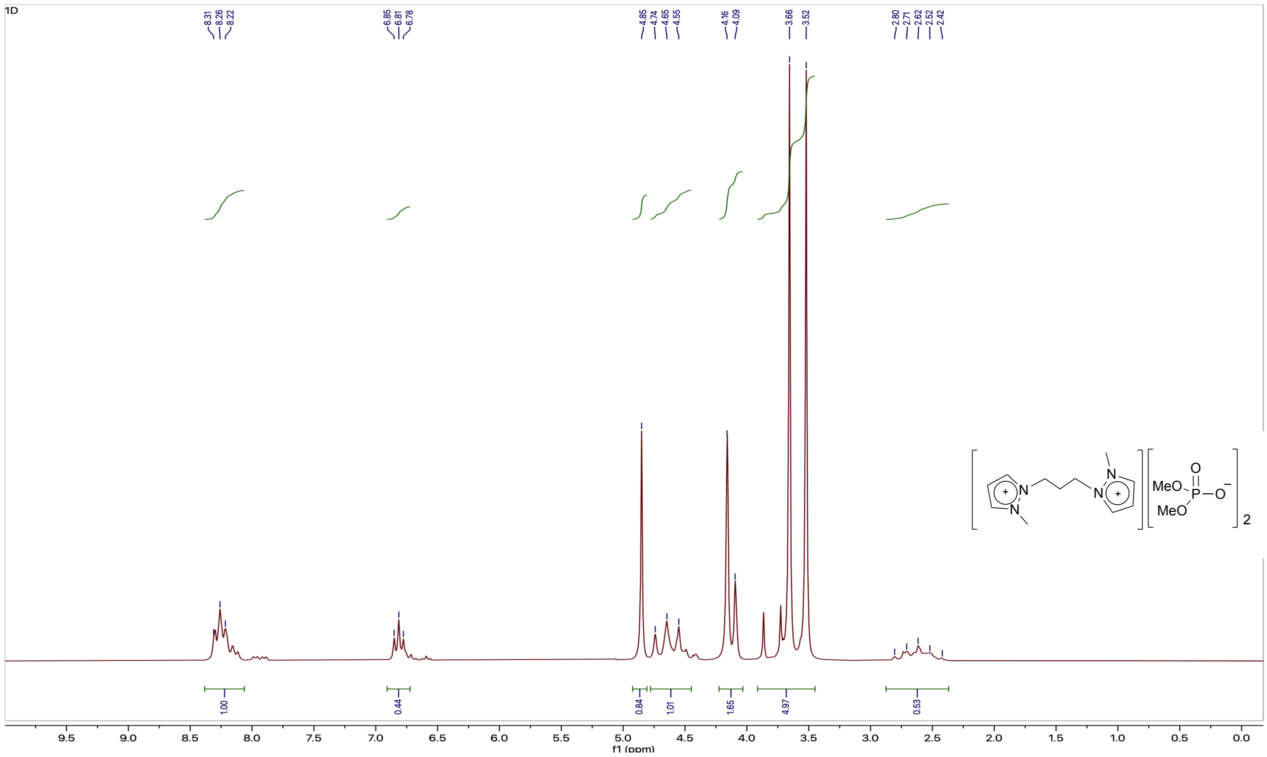

Figure S11-37. ^{1}H NMR of [Bis(Pyra-1)C3][DMPO$_4$]$_2$. (80 MHz, D_2O).

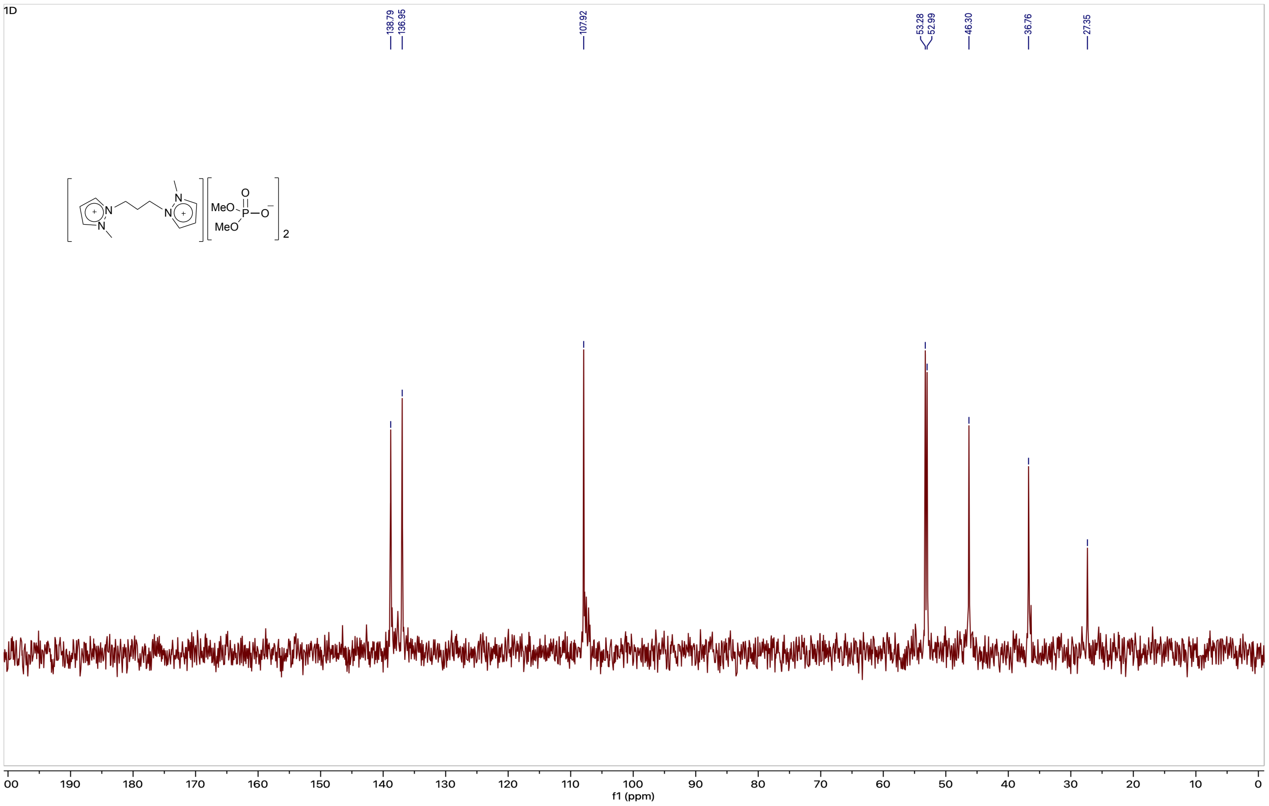

Figure S11-38. ^{13}C NMR of [Bis(Pyra-1)C3][DMPO$_4$]$_2$. (20 MHz, D_2O).

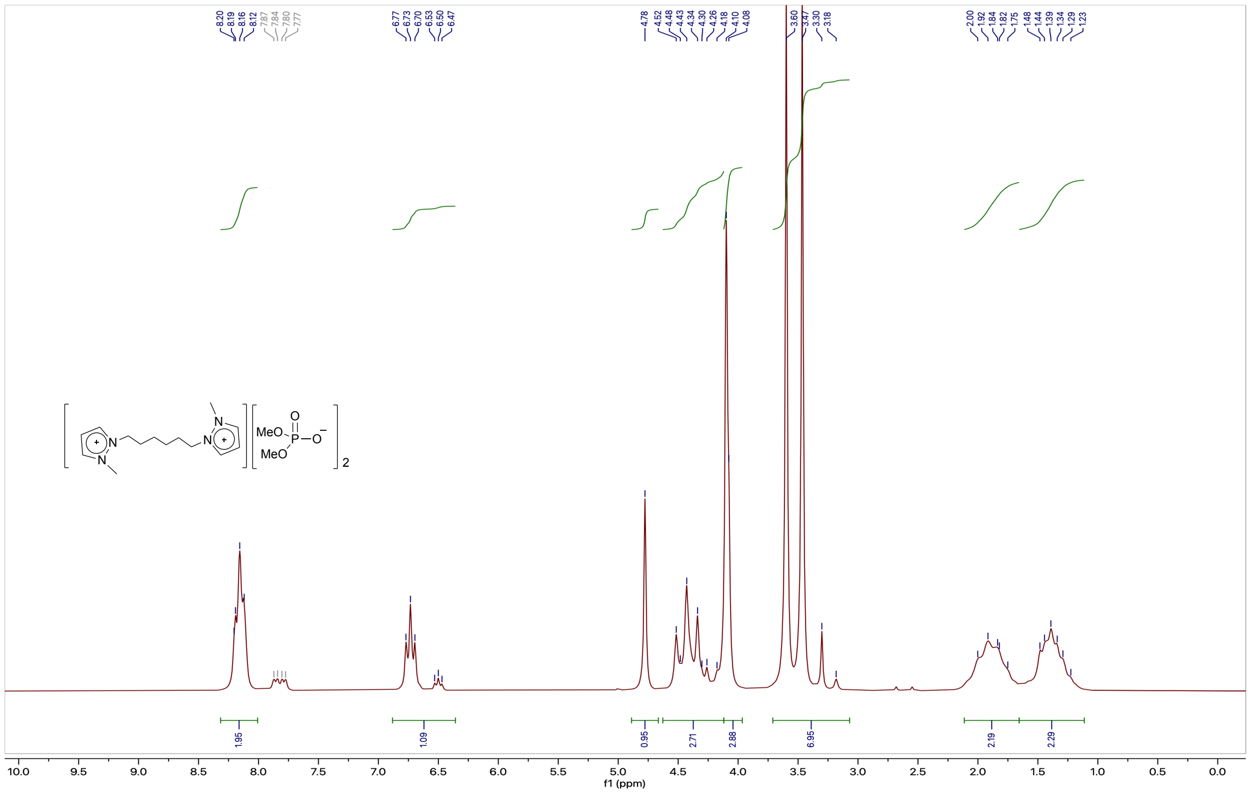

Figure S11-39. ^{1}H NMR of [Bis(Pyra-1)C6][DMPO$_4$]$_2$. (80 MHz, D_2O).

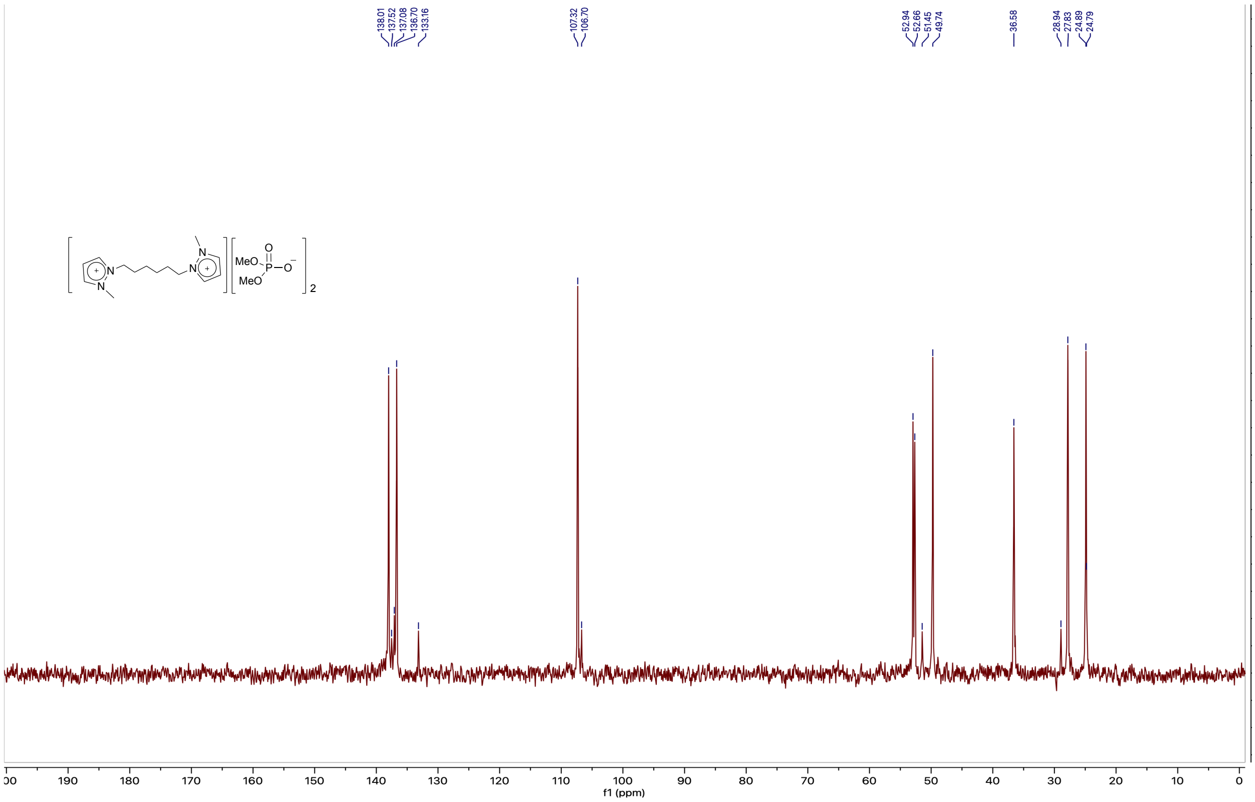

Figure S11-40. ^{13}C NMR of [Bis(Pyra-1)C6][DMPO$_4$]$_2$. (20 MHz, D_2O).

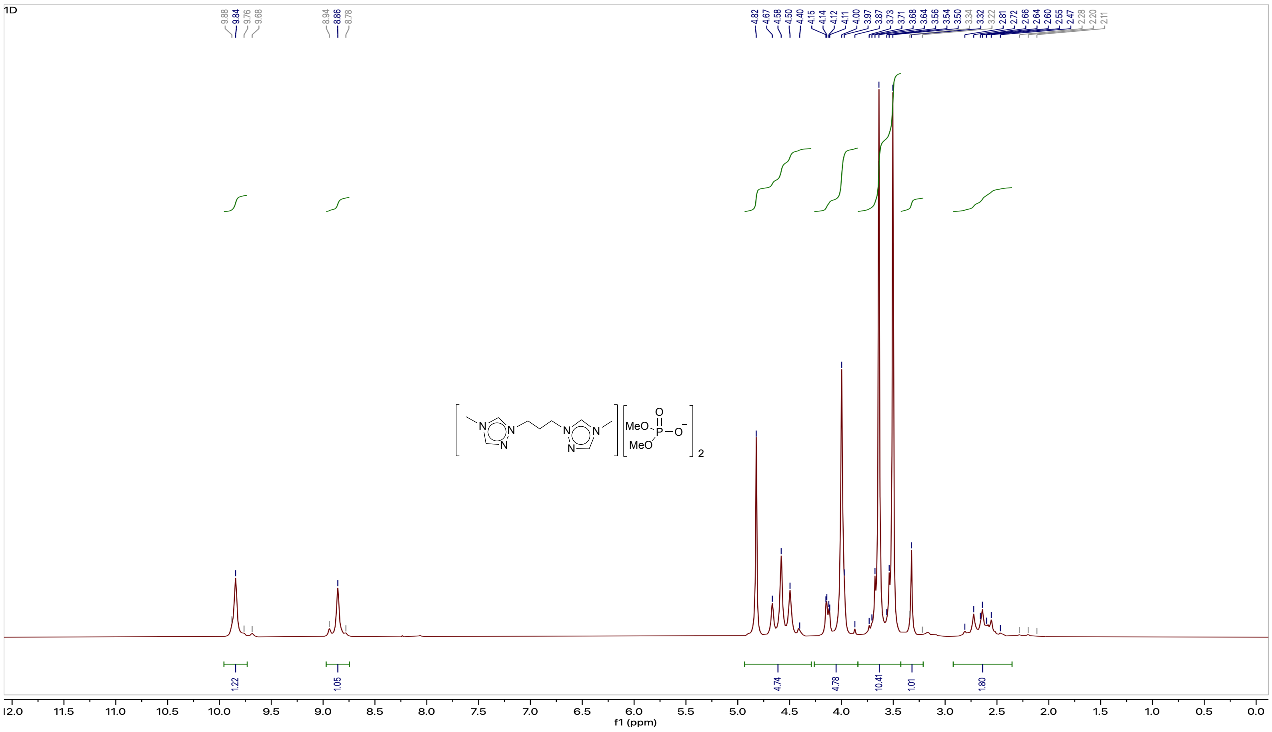

Figure S11-41. ^{1}H NMR of [Bis(124-Tz-1)C3][DMPO$_4$]$_2$. (80 MHz, D_2O).

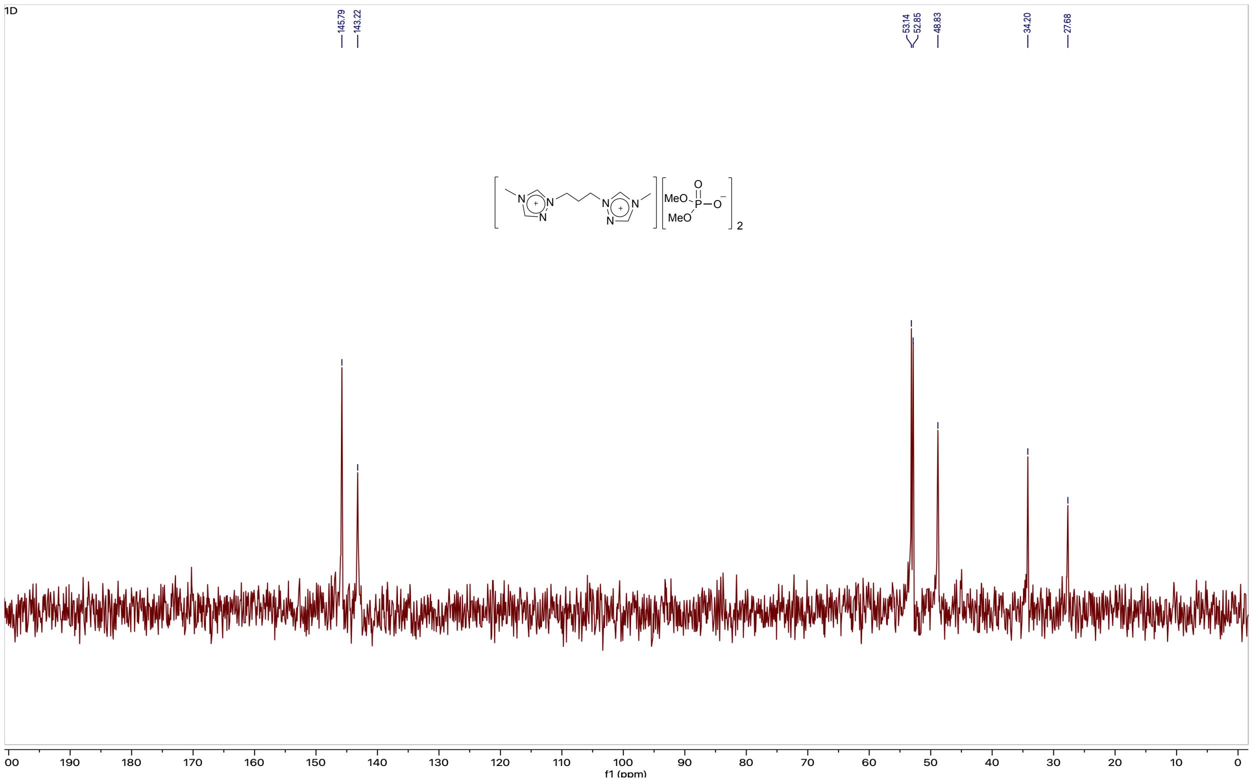

Figure S11-42. ^{13}C NMR of [Bis(124-Tz-1)C3][DMPO$_4$]$_2$. (20 MHz, D_2O).

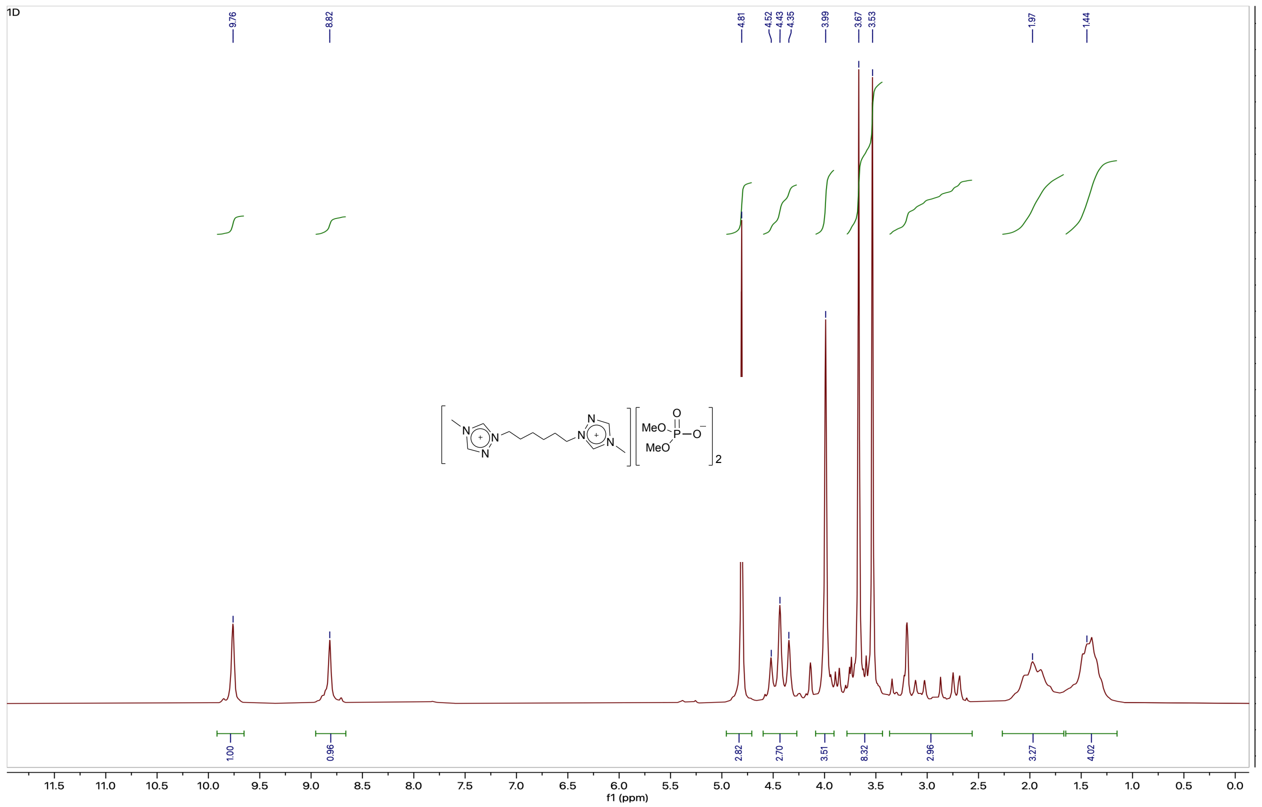

Figure S11-43. ^{1}H NMR of [Bis(124-Tz-1)C6][DMPO$_4$]$_2$. (80 MHz, D$_2$O).

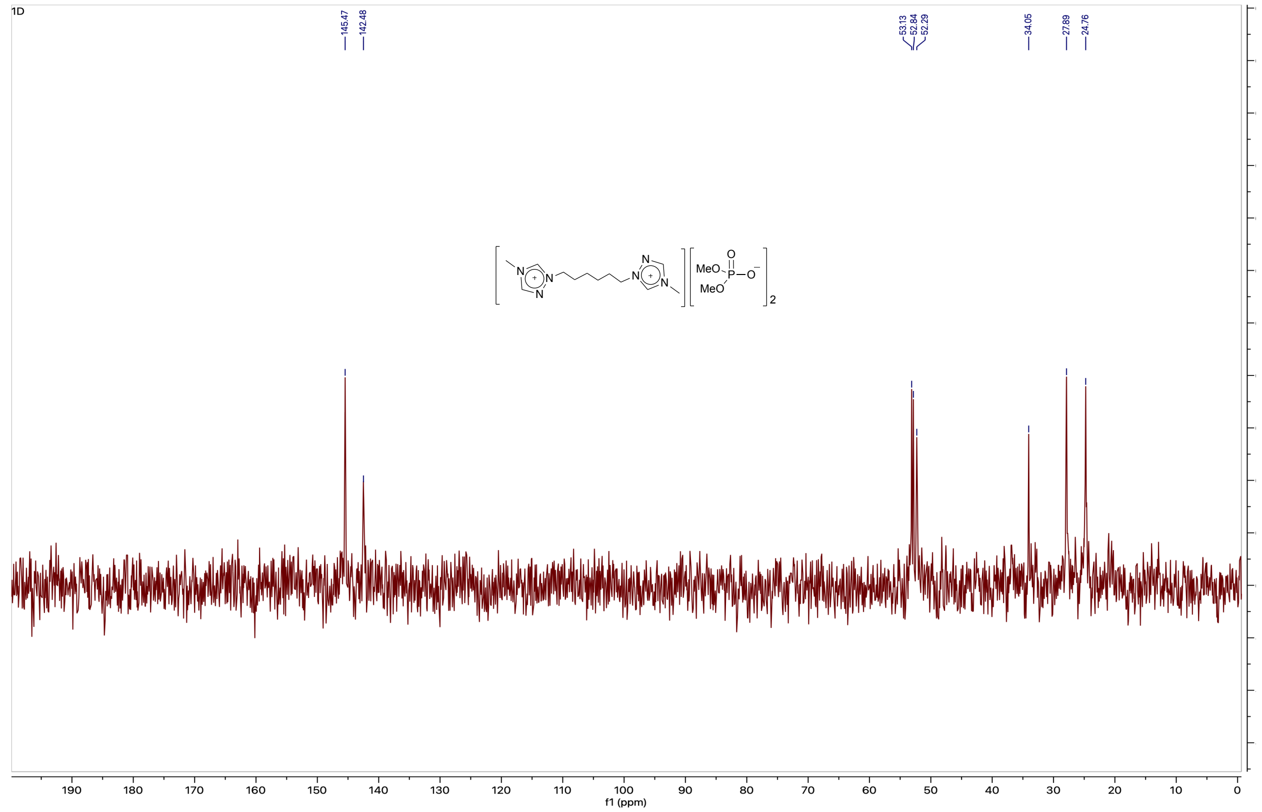

Figure S11-44. ^{13}C NMR of [Bis(124-Tz-1)C6][DMPO$_4$]$_2$. (20 MHz, D$_2$O).

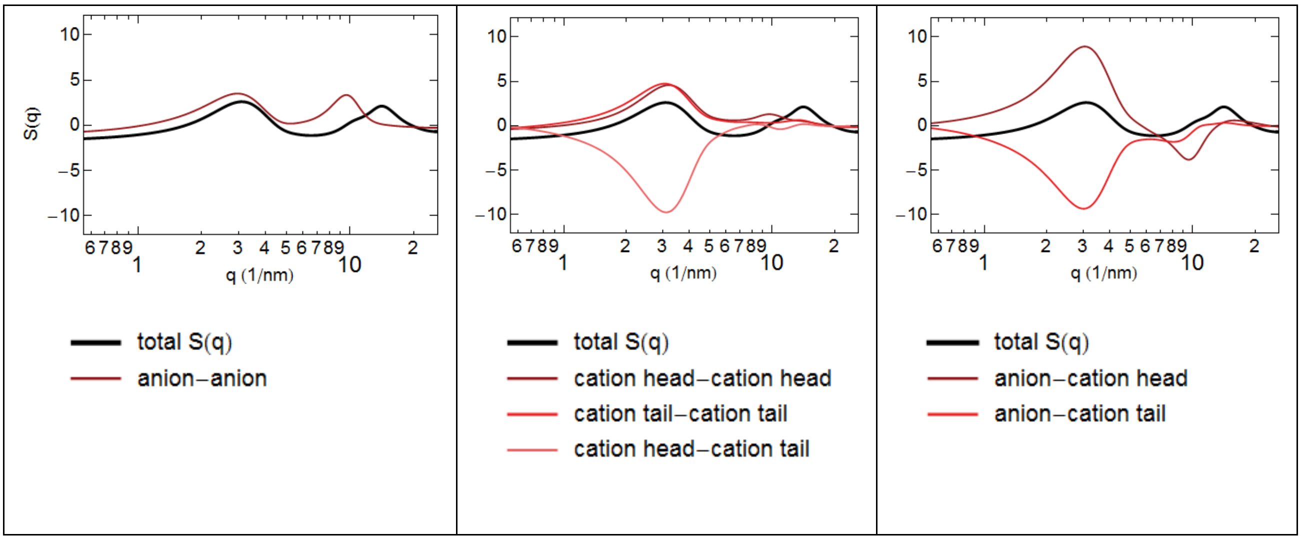

Figure S12. MD results for partial structure factors of pure TZ8 ([124-Tz-1,8][DMPO4]). The thick black curves show the total structure factor, *S*(*q*), calculated from the MD simulation. The cation head groups ("cation head") are polar; the cation tail groups ("cation tail") are nonpolar. The anions are polar.

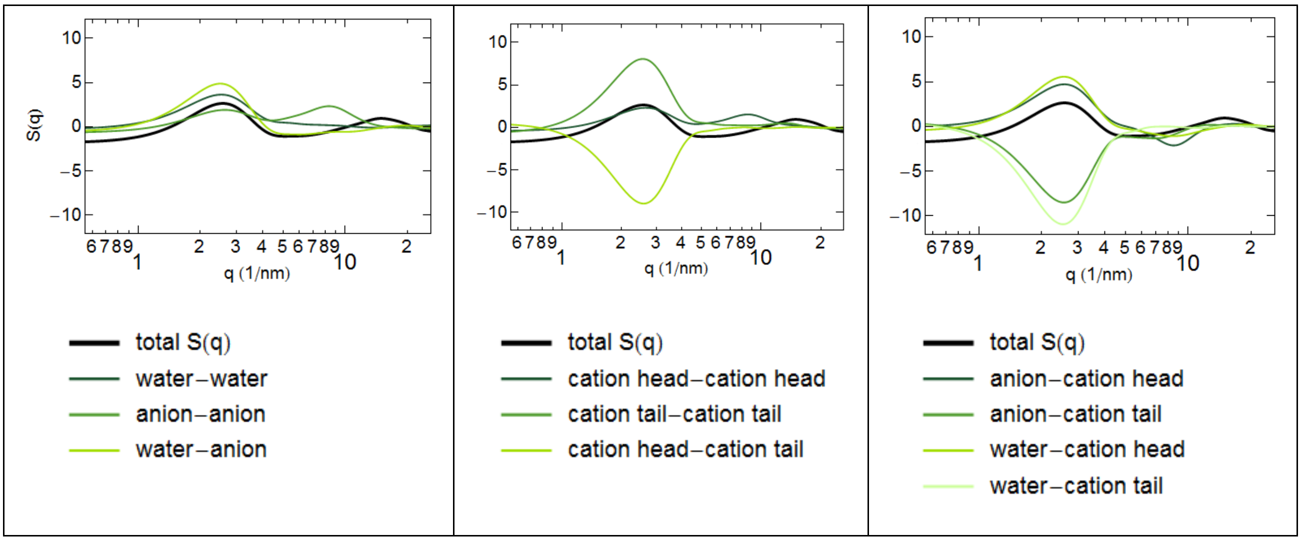

Figure S13. MD results for partial structure factors of 80% (w/w) aqueous solution of TZ8 ([124-Tz-1,8][DMPO4]). The thick black curves show the total structure factor, *S*(*q*), calculated from the MD simulation. The cation head groups ("cation head") are polar; the cation tail groups ("cation tail") are nonpolar. The anions are polar.

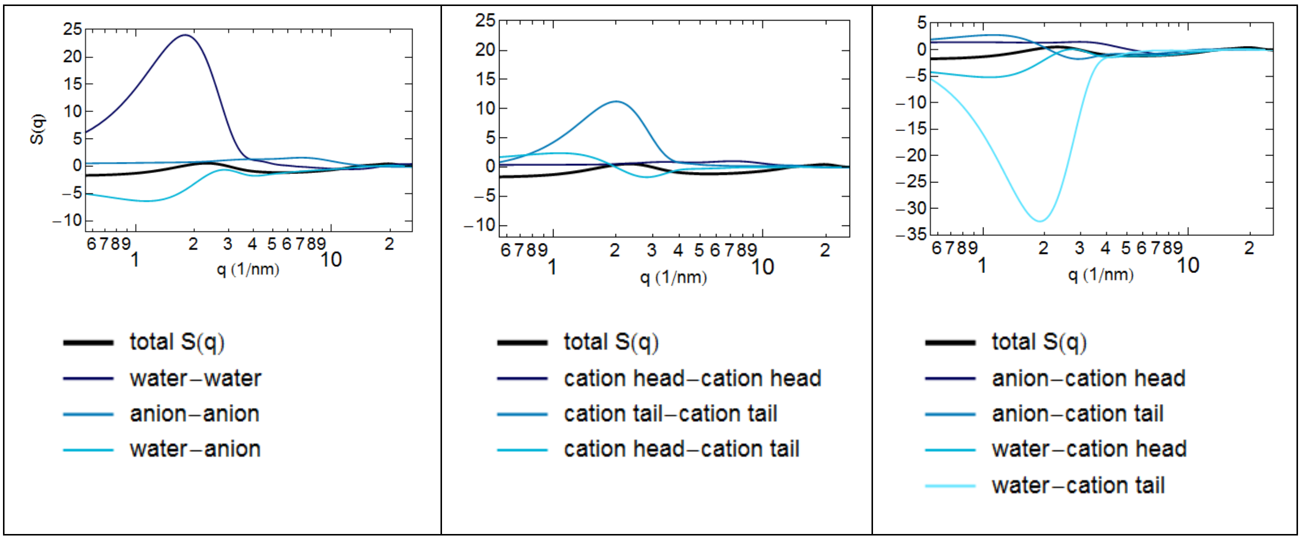

Figure S14. MD results for partial structure factors of 50% (w/w) aqueous solution of TZ8 ([124-Tz-1,8][$DMPO_4$]). The thick black curves show the total structure factor, $S(q)$, calculated from the MD simulation. The thin blue curves show the contributions of the indicated pairwise distributions to $S(q)$. The cation head groups ("cation head") are polar; the cation tail groups ("cation tail") are nonpolar. The anions are polar.

Table S2. Results of SMAXS analysis of Tz8 ([124-Tz-1,8][$DMPO_4$]) .

q value (1/nm)	TZ8_40 40%(w/w) χIL=0.036	TZ8_60 60% (w/w) χIL=0.077	TZ8_80 80%(w/w) χIL=0.18	TZ8_90 90%(w/w) χIL=0.34	TZ8_100 Pure IL χIL=1.0
	Peak Intensity				
**0.0115**	383.8606	620.7509	419.2253	549.7895	632.2481
**0.0346**	419.1509	557.7216	382.2181	543.1651	774.0193
**0.0577**	473.8975	523.1851	442.4965	618.7749	773.9971
**0.0807**	450.8084	472.9998	392.1527	614.5053	728.1312
**0.104**	439.8889	482.7261	405.1036	626.0804	717.8488
**0.127**	401.6253	456.5317	401.0068	563.2948	709.7298
**0.150**	409.0338	446.8997	400.8586	586.502	708.243
**0.173**	404.8009	477.3346	401.7754	559.2005	765.1605
**0.196**	424.5079	480.8216	400.757	560.6049	754.4954
**0.219**	451.7726	488.1902	412.6669	591.873	752.0058
**0.242**	419.554	480.2563	403.2946	608.5997	769.6205
**0.265**	431.7545	490.3865	430.9407	582.2798	774.8042
**0.288**	434.4102	494.8244	410.6832	575.1701	760.8276
**0.311**	439.3555	470.4137	427.0588	560.513	786.7188
**0.334**	450.9565	480.3908	430.9687	577.8882	781.5091
**0.357**	460.7033	485.2941	440.6892	603.153	814.8721
**0.381**	462.3533	486.181	448.9689	605.7868	811.5664
**0.404**	489.5849	517.1317	446.3944	630.9489	817.9053
**0.427**	513.8621	539.9719	461.6261	625.5384	815.4133
**0.450**	541.3167	564.0573	464.5514	653.6992	831.8233
**0.473**	580.7739	602.3688	508.4737	684.3255	898.143
**0.496**	1057.534	875.0996	490.5126	876.0732	1010.884
**0.519**	3576.105	5129.176	3157.222	5012.605	4361.297
**0.542**	14821.09	15609.83	12637.85	18095.44	22812.03
**0.565**	28719.1	27697.76	23179.5	32972.41	44527.99
**0.588**	32139.39	30570.44	25342.08	36795.67	50342.01
**0.611**	32149.34	30544.94	25446.34	37291.12	50866.62
**0.634**	32319.87	30382.08	25670.08	37552.64	51454.8
**0.657**	32547.31	30604.29	26015.37	37938.82	51892.34
**0.680**	32473.52	30486.95	26015.78	37895.42	52216.06
**0.703**	32709.61	30668.02	26448.07	38558.89	52821.95
**0.726**	32660.23	30949.69	26727.51	38753.87	53109.77
**0.750**	32740.8	31374.57	26933.06	39138.51	53630.6
**0.773**	32881.89	31584.87	27165.02	39513.63	54369.46
**0.796**	32856.31	31696.5	27464.34	39896.52	54729.93
**0.819**	33015.26	32084.64	27790.95	40196.94	55109.26

**0.842**	33081.96	32411.59	28127.46	40952.33	56129.44
**0.865**	33439.88	32857.43	28776.58	41673.21	56979.93
**0.888**	33645.17	33398.13	29068.67	42132.28	57658.08
**0.911**	33605.75	33591.73	29367.96	42713.4	58587.5
**0.934**	33796.91	33912.42	29854.88	43269.47	59411.65
**0.957**	33923.52	34450.7	30299.01	43918.92	60184.54
**0.98**	34131.99	34904.39	30654.08	44486.36	60951.98
**1.00**	34293.76	35416.47	31318.07	45262.72	61766.14
**1.03**	34436.47	36001.63	31879.89	45966.48	62622.25
**1.05**	34571.82	36580.51	32246.58	46660.3	63638.28
**1.07**	35012.52	37337.07	32877.12	47234.21	64674.64
**1.10**	35093.23	37742.56	33614.71	48105.77	65491.44
**1.12**	35571.01	38482.56	34060.09	49013.3	66484.88
**1.14**	36105.48	39333.52	34854.68	50104.75	67663.89
**1.16**	36338.58	40105.26	35469.69	50910.29	68717.96
**1.19**	36793.45	41144.73	36178.92	51796.31	69906.48
**1.21**	37490.11	42074.25	37095.53	52705.42	71103.38
**1.23**	38261.97	43083.75	38029.97	53867.35	72200.44
**1.26**	38773.95	43893.93	38519.98	54744.38	73333.03
**1.28**	39595.48	45168.48	39453.54	55756.29	74744.71
**1.30**	40421.25	46468.75	40274.16	56895.43	75971.61
**1.33**	41449.39	47897.13	41267.12	57935.9	77307.54
**1.35**	42738.53	49488.04	42462.86	59437.57	78946.3
**1.37**	44234.33	51310.96	43843.6	60928.22	80464.77
**1.4**	45895.34	53249.9	44957.75	62353.26	82051.29
**1.42**	47672.7	55383.34	46270.93	63865.86	83965.54
**1.44**	49903.25	57826.63	47769.82	65619.95	85651.37
**1.46**	52438.03	60277.12	49317.88	67330.65	87677.47
**1.49**	55211.88	63229.12	51031.49	69305.8	89658.7
**1.51**	58296.39	66433.1	52897.99	71224.22	91755.22
**1.53**	61806.16	69983.57	54816.1	73276.72	93763.64
**1.56**	65801.86	73972.49	56969.79	75404.17	96035.39
**1.58**	70223.08	78356.13	59201.16	77857.7	98195.16
**1.60**	74765.76	83197.9	61542.88	80307.97	100578.7
**1.63**	79562.96	88287.91	64004.18	82619.46	103011.6
**1.65**	85042.35	94332.54	67090.59	85668.97	105571.2
**1.67**	90487.55	101022.3	70042.83	88759.95	108476.8
**1.70**	96337.49	108356.8	73387.7	91796.92	111373.3
**1.72**	102124.7	116617.5	76971.05	95225.96	114154

**1.74**	107343.8	125351.7	80790.53	98891.69	117413.6
**1.76**	112328.3	134807.1	84831.31	102489.1	120577.7
**1.79**	117005.7	145242.9	89442.25	106525.2	123851.3
**1.81**	120954.2	156243.6	94227.22	110682	127364
**1.83**	124401.1	168233.2	99695.7	115340	131129.3
**1.86**	127174	180755.5	105415.4	120296	135213.3
**1.88**	129125	193692.3	111632	125492.7	139448.5
**1.90**	130503.3	207128.8	118624.7	131185	143774.8
**1.93**	130922.3	219730.8	125733.4	137034.6	147941.4
**1.95**	130977.1	231952	133502.8	143281.7	152604.7
**1.97**	130639	243575.8	141886.8	150058.9	157473.5
**1.99**	129486.4	253650.3	151094.3	157195	162512.9
**2.02**	128095.6	262061.5	160262.6	164486.1	167649.6
**2.04**	126691.4	268677.2	170606	172608	173214.4
**2.06**	125068.2	273286.5	181480.1	181168.9	178970.3
**2.09**	123266.5	275739.9	192480.6	190223.8	185022.3
**2.11**	121405.7	275367.4	203915.1	199584.5	191061.2
**2.13**	119570.7	273549.6	216207.7	209929.2	197462.1
**2.16**	117516.4	269826.4	228080.6	220279.9	204239.6
**2.18**	115816.9	264679.9	240153	231507.9	211474.6
**2.20**	114049.9	257773.3	251941	243133.6	218719.8
**2.23**	112414.6	250618.4	263152.9	255036.1	226502.2
**2.25**	110670.3	242123.8	273168.7	267047.2	234115.5
**2.27**	109271.9	233423.1	282618.4	279738.5	241958.2
**2.29**	107789.8	225428.7	290827.2	292568.1	250545.9
**2.32**	106647	216995.5	297666.2	306144.1	259389.5
**2.34**	105292.1	208523.3	302321	319064.3	268099.8
**2.36**	104012.5	200401.3	305049.6	331896.5	277128
**2.39**	102808.4	192638.5	306209.1	344689.7	286349.5
**2.41**	101964.2	185322.6	305413.5	356653.1	295926.6
**2.43**	100916.2	178434.6	302496.1	367798.1	305435.2
**2.46**	100078.4	171898.3	298411.7	378553.9	315339.8
**2.48**	99303.19	165902.5	292984.2	387934.4	325102.3
**2.50**	98451.31	160303.3	285997.1	396341.5	334677.3
**2.53**	97728.6	154795.1	278322.8	402903.5	344721
**2.55**	97111.02	150051.6	269958.8	408506	354561.6
**2.57**	96407.9	145371.2	260584.1	411932.5	363853.5
**2.59**	95947.51	141206.7	251571	413961.2	373367.7
**2.62**	95336.61	137153.5	242050.2	414221.3	382305.3

**2.64**	94798.24	133437.9	232370.3	413148.7	390700.3
**2.66**	94430.46	130150.1	223171.7	410161.3	399481.4
**2.69**	93943.67	126903.8	214103.9	405662.9	407194.5
**2.71**	93617.91	124079.2	205541.7	400282.7	414367.8
**2.73**	93175.57	121208.3	197004.5	393236.4	420840.2
**2.76**	92980.58	118610.3	189022.3	385408.8	426775.9
**2.78**	92588.46	116464.7	181486.6	377041.6	432555.7
**2.80**	92199.48	114002.9	173953.1	367113.9	435972.7
**2.83**	91838.96	111977.6	167022.5	357244.2	439362.8
**2.85**	91619.43	110102.4	160559.2	347426.8	442333.7
**2.87**	91362.01	108315.5	154427.3	337010.3	444197.2
**2.89**	91031.45	106582.3	148855.2	326477.9	445280.3
**2.92**	90701.08	104992.3	143506.2	316028.3	445241.4
**2.94**	90485.04	103573.1	138420.2	305787.8	444648.6
**2.96**	90176.71	102208	133645.7	295669.1	443238.4
**2.99**	89904.79	100915	129207.2	285806.8	440640.6
**3.01**	89567.74	99744.01	125093.5	275890.1	437491.6
**3.03**	89417.02	98592.84	121235.2	267016.2	433966.5
**3.06**	89019.75	97595.55	117489.9	257659.3	429419.5
**3.08**	88746.13	96745.16	114112.7	249108.3	424814.5
**3.10**	88431.5	95801.76	110869.9	240660.3	419205.3
**3.12**	88097.67	94848.14	107862.7	232772.9	412991.7
**3.15**	87665.26	94005.29	104938.9	225062.7	406356.4
**3.17**	87390.86	93273.63	102309.2	217958.1	400186
**3.19**	86999.36	92475.66	99716.75	211005.3	392915.6
**3.22**	86576.93	91918.19	97377.56	204288.4	385777
**3.24**	86266.34	91351.02	95055.61	198056.1	378516.7
**3.26**	85689.46	90607.56	92835.71	192067	370914.2
**3.29**	85413.11	90168.04	90914.12	186541.3	363328.3
**3.31**	84813.79	89548.27	88990.29	181034.6	355711.6
**3.33**	84290.25	88958.65	87176.08	175820.5	347823.7
**3.36**	83850.11	88442.38	85485.68	171015.4	340444.5
**3.38**	83358.88	87988.93	83877.15	166392.3	332717.1
**3.40**	82867.53	87630.63	82511.53	162227.7	325555.8
**3.42**	82298.76	87107.89	81005.64	157890.5	318181.2
**3.45**	81727.96	86631.93	79593.59	153814.9	310889.7
**3.47**	81251.73	86277.28	78312.6	150101.3	303925.8
**3.49**	80794.44	85819.01	77082.95	146475.7	296924
**3.52**	80147.05	85312.19	75937.82	142984.9	290174.2

**3.54**	79470.35	84919.47	74848.97	139794.9	283549
**3.56**	78928.78	84573.84	73888.75	136807.3	277167.7
**3.59**	78395.97	84135.39	72879.39	133739.8	270852.4
**3.61**	77756.92	83743.39	71841.88	130930.3	264461.2
**3.63**	77118.98	83311.65	70866.61	128237.7	258432.5
**3.66**	76523.63	82891.66	70072.16	125619.6	253170.6
**3.68**	75926.64	82515.93	69269.98	123225.8	247505.1
**3.70**	75406.69	82014.36	68525.18	120767.3	242032.7
**3.72**	74802.14	81699.51	67783.52	118632.1	236876.8
**3.75**	74231.71	81350.94	67028.74	116586.4	231849.7
**3.77**	73559.03	80847.03	66370.92	114483.1	226810.8
**3.79**	73014.29	80481.71	65745.29	112566.3	222243.3
**3.82**	72441.13	79967.04	65165.27	110763.9	217751.2
**3.84**	71995.43	79570.98	64576.21	109077.2	213301.6
**3.86**	71308.94	79213.73	64031.09	107252.7	208926.5
**3.89**	70908.24	78740.85	63549.84	105600.5	204804.1
**3.91**	70276.73	78153.47	62904.65	103873.1	200679.8
**3.93**	69709.9	77661.51	62366.14	102359.6	196834.7
**3.96**	69196.57	77236.35	61895.74	101011.6	193081.3
**3.98**	68776.58	76792.46	61491.73	99678.75	189615.9
**4**	68343.6	76461.82	61127.24	98523.47	186410.1
**4.02**	67939.94	76060.12	60702.07	97174.97	183259.8
**4.05**	67670.89	75636.74	60427.44	95988.96	179999.1
**4.07**	67191.6	75146.85	60030.12	94898.49	176913.6
**4.09**	66738.26	74704.5	59666.52	93871.11	173944.8
**4.12**	66504.85	74327.3	59304.9	92817.96	171179.6
**4.14**	66126.49	73908.9	58999.72	91771.98	168252.6
**4.16**	65647.85	73440.79	58686.63	90805.73	165614.2
**4.19**	65412.46	72999.08	58328.53	89770.74	163070
**4.21**	65083.03	72487.07	58017.72	88938.78	160534.7
**4.23**	64781.46	72083.18	57664.09	88007.03	158066.1
**4.26**	64430.13	71741.58	57420.17	87245.48	155806.2
**4.28**	64229.83	71330.07	57167.83	86470.51	153658.9
**4.3**	63914.18	70863.09	56871.37	85518.72	151302.5
**4.32**	63668.61	70481.31	56561.61	84877.46	149359.4
**4.35**	63393.52	69999.72	56260.37	84090.74	147251.1
**4.37**	63135.05	69617.71	56021.38	83407.17	145177.2
**4.39**	62939.76	69210.3	55721.36	82638.48	143046.5
**4.42**	62484.45	68713.53	55374.33	81905.08	140962.5

**4.44**	62314.15	68364.69	55109.63	81263.7	139079.5
**4.46**	62143.65	68050.1	54850.49	80537.22	137325.8
**4.49**	61867.52	67608.77	54582.94	79853.19	135491.6
**4.51**	61725.16	67315.6	54299.92	79278.68	133774.9
**4.53**	61591.27	66957.57	54087.16	78786.59	132238.7
**4.55**	61322.32	66513.65	53807.03	78129.31	130551.4
**4.58**	61178.22	66226.54	53462.45	77621.87	129104.4
**4.6**	61005.74	65908.52	53253.73	77027.61	127598.2
**4.62**	60899.85	65711.46	53035.51	76557.21	126186.2
**4.65**	60766.26	65457.37	52799.85	76069.36	124890.9
**4.67**	60685.11	65212.95	52594.89	75655.75	123413.4
**4.69**	60499.02	64950.78	52304.48	75158.83	122114.6
**4.72**	60348.56	64670.13	52095.4	74728.98	120723.5
**4.74**	60258.28	64467.61	51836.74	74193.31	119615.5
**4.76**	60112.32	64182.24	51623.92	73726.34	118300.4
**4.79**	60012.53	64119.26	51442.52	73426.5	117182
**4.81**	59915.12	63831.28	51146.08	72926.24	116023.9
**4.83**	59902.06	63698.76	50904.97	72506.29	114877.1
**4.85**	59795.47	63525.33	50743.2	72229.43	113805.9
**4.88**	59831.11	63474.48	50626.5	71967.91	112892.5
**4.9**	59725.39	63321.99	50353.09	71571.45	111947.1
**4.92**	59713.99	63275.79	50185.49	71221.88	111030.1
**4.95**	59631.81	63146.5	50009.28	70948.66	110102.6
**4.97**	59615.49	62998.06	49803.48	70537.76	109201
**4.99**	59758.52	62903.65	49668.19	70316.68	108372.7
**5.02**	59662.74	62770.6	49470.28	69908.22	107416.1
**5.04**	59540.3	62664.74	49315.93	69582.22	106574.8
**5.06**	59557.74	62675.14	49164.48	69315.58	105783
**5.09**	59573.61	62538.08	49001.31	69038.23	104977.6
**5.11**	59538.65	62522.89	48873.52	68803.68	104235.8
**5.13**	59527.55	62472.94	48768.29	68558.93	103509
**5.15**	59498.52	62384.64	48551.56	68257.96	102733.6
**5.18**	59513.3	62406.96	48445.74	68010.47	102095.8
**5.2**	59562.36	62324.42	48321.51	67766.4	101346.7
**5.22**	59601.7	62326.06	48222.34	67526.62	100739.9
**5.25**	59563.65	62379.5	48136.35	67309.73	100094.5
**5.27**	59637.58	62389.94	48071.78	67197	99535.47
**5.29**	59591.71	62381.39	47917.39	66871.97	98903.57
**5.32**	59677.48	62338.85	47872.66	66681.77	98326.95

**5.34**	59644.83	62367.51	47743.57	66457.38	97610.51
**5.36**	59697.12	62387.22	47620.92	66204.48	97072.18
**5.39**	59746.89	62398.57	47577.88	66049.55	96661.17
**5.41**	59813.7	62455.87	47470.14	65882.9	96087.13
**5.43**	59745.9	62484.81	47381.02	65666.37	95524.77
**5.45**	59768.34	62431.45	47319.79	65454.8	95031.36
**5.48**	59799.47	62547.11	47270.85	65338.45	94523.99
**5.5**	59847.83	62569.13	47222.86	65191.98	94022.22
**5.52**	59840.19	62546.74	47189.61	64920.41	93571.47
**5.55**	59958.33	62621.51	47123.31	64834.76	93153.35
**5.57**	59975.61	62726.8	47166.42	64708.15	92781.73
**5.59**	60046.49	62712.37	47176.88	64626.59	92368.57
**5.62**	60066.02	62723.24	47091.42	64443.98	91862.97
**5.64**	60093.95	62800.65	47068.99	64312.86	91529.39
**5.66**	60185.1	62872.83	47085.85	64153.47	91063.73
**5.68**	60269.72	62984.99	47127.51	64125.92	90887.44
**5.71**	60321.29	63038.83	47070.25	63998.8	90405.33
**5.73**	60376.28	63152.33	47087.78	63918.54	90030.86
**5.75**	60401.73	63194.79	47064.46	63771.28	89674.64
**5.78**	60483.25	63313.92	47105.76	63767.19	89370.32
**5.8**	60582.6	63430.86	47110.33	63674.6	89106.86
**5.82**	60577.22	63479.09	47158.43	63675.15	88838.44
**5.85**	60615.28	63549.06	47170.82	63502.04	88460.49
**5.87**	60624.83	63626.63	47203.2	63422.27	88134.24
**5.89**	60795.59	63754.46	47178.05	63390.26	87895.41
**5.92**	60851.25	63845.68	47201.15	63391.98	87598.16
**5.94**	60869.62	63813.78	47230.36	63290.23	87367.64
**5.96**	60883.11	63946.77	47273.51	63289.23	87085.35
**5.98**	60977.87	63949.29	47313.53	63211.58	86797.49
**6.01**	60972.31	64060.42	47319.91	63141.5	86484.64
**6.03**	61099.89	64235.18	47411.29	63203.1	86352.29
**6.05**	61201.23	64366.42	47537	63279.63	86201.09
**6.08**	61230.17	64426.76	47570.63	63247.9	85955.04
**6.1**	61367.73	64601.67	47685.82	63272.27	85777.73
**6.12**	61453.99	64782.05	47768.26	63274.16	85637.45
**6.15**	61499.79	64837.11	47805.78	63271.18	85452.74
**6.17**	61562.23	64934.27	47893.56	63246.02	85338.91
**6.19**	61701.13	65117.09	47963.61	63330.16	85148.27
**6.22**	61803.72	65205.61	48064.29	63328.94	84982.05

**6.24**	61825.18	65366.86	48100.14	63385.6	84865.03
**6.26**	61905.15	65429.77	48178.34	63448.02	84805.66
**6.28**	61923.88	65549.76	48257.96	63494.49	84692.76
**6.31**	62049.04	65653.19	48392.4	63515.16	84532.75
**6.33**	62120.67	65793.35	48486.29	63588.67	84470.69
**6.35**	62207.14	66008.21	48582.83	63677.72	84330.26
**6.38**	62297.05	66113.67	48686.04	63775.81	84272.12
**6.4**	62421.02	66222.95	48824.98	63780.57	84199.01
**6.42**	62482	66305.9	48927.06	63899.44	84197.78
**6.45**	62475.2	66366.34	48926.3	63812.17	83959.91
**6.47**	62593.49	66509.57	48986.52	63903.86	83905.07
**6.49**	62633.61	66604.34	49085.32	64026.67	83890.75
**6.52**	62732.8	66795.17	49235.44	64125.56	83834.34
**6.54**	62857.09	66976.66	49410.84	64294.86	83856.29
**6.56**	62946.58	67158.78	49561.66	64364.24	83875.25
**6.58**	63013.76	67262.43	49612.52	64423.45	83868.83
**6.61**	63101.9	67348.71	49758.89	64558.05	83843.8
**6.63**	63172.05	67497.14	49876.1	64734.27	83821.93
**6.65**	63308.6	67658.26	50069.29	64867.88	83830.7
**6.68**	63434.07	67829.93	50224.09	65019.98	83951.4
**6.7**	63507.82	68018.34	50341.3	65208.21	83935.08
**6.72**	63601.45	68223.39	50499.43	65385.79	84047.78
**6.75**	63739.52	68320.42	50627.33	65534.49	84123.76
**6.77**	63828.47	68467.27	50733.27	65632.1	84090.85
**6.79**	63927.31	68587.7	50890.19	65733.56	84107.85
**6.82**	63969.66	68784.19	51030.23	65897.65	84191.17
**6.84**	64144.13	68991.23	51207.77	66132.65	84303.52
**6.86**	64204.55	69102.91	51325.38	66274.07	84330.74
**6.88**	64304.11	69328.85	51507.6	66510.73	84499.54
**6.91**	64380.22	69464.26	51708.09	66695.31	84604.95
**6.93**	64539.51	69605.66	51852.57	66860.38	84697.08
**6.95**	64676.8	69783.41	52010.92	67046.52	84836.31
**6.98**	64691.56	70033.1	52167.41	67237.01	84916.33
**7**	64805.05	70129.66	52344.88	67431.65	85031.21
**7.02**	64923.37	70257.7	52491.9	67640.53	85158.98
**7.05**	65044.13	70482.43	52716.11	67812.69	85259.39
**7.07**	65206.35	70684.5	52881.08	68069.26	85479.94
**7.09**	65366.58	70804.22	53014.85	68271.24	85621.05
**7.11**	65375.71	70988.63	53189.58	68466.91	85850.4

**7.14**	65517.7	71144.08	53341.68	68723.7	85963.58
**7.16**	65618.96	71307.78	53501.61	68950.66	86138.78
**7.18**	65682.73	71522.45	53742.79	69138.27	86372.75
**7.21**	65874.81	71601.7	53923.52	69425.16	86532.86
**7.23**	65952.48	71801.62	54118.21	69642.62	86699.8
**7.25**	66042.74	71966.34	54274.68	69840.2	86867.36
**7.28**	66220.16	72194.03	54480.92	70106.68	87087.59
**7.3**	66269.76	72304.75	54682.91	70361.81	87303.11
**7.32**	66398.12	72504.49	54869.33	70631.05	87506.16
**7.35**	66476.57	72617.9	55021.76	70882.22	87679.2
**7.37**	66568.2	72858.26	55169.61	71174.74	87949.52
**7.39**	66707.36	73074.27	55376.6	71359.44	88246.94
**7.41**	66893.81	73213.82	55576.12	71642.29	88486.38
**7.44**	66913.13	73400.25	55768.67	71937.53	88683.81
**7.46**	67078.19	73560.28	55960.74	72200.43	88900.65
**7.48**	67197.3	73716.32	56175.24	72503.14	89193.08
**7.51**	67265	73928.23	56369.96	72807.75	89484.93
**7.53**	67409.03	74105.25	56549.82	73045.66	89839.81
**7.55**	67541.19	74289.96	56806.95	73346.93	90080.74
**7.58**	67645.26	74429.65	57003.08	73654.76	90366.15
**7.6**	67757.72	74663.27	57211.11	73878.59	90567.12
**7.62**	67963.67	74887.11	57486.02	74232.19	90962.98
**7.65**	68069.11	75049.2	57637.98	74592.17	91255
**7.67**	68196.78	75275.04	57885.08	74939.37	91600.95
**7.69**	68341.32	75480.08	58137.91	75318.51	91959.42
**7.71**	68479.59	75652.96	58334.85	75568.53	92406.2
**7.74**	68621.82	75902.81	58510.7	75903.8	92715.45
**7.76**	68773.63	76089.8	58783.61	76314.37	93091.78
**7.78**	68846.66	76249.31	59016.65	76600.61	93387.22
**7.81**	68993.7	76470.54	59271.58	76922.67	93742.41
**7.83**	69066.39	76666.28	59501.45	77340.82	94171.96
**7.85**	69240.14	76879.17	59689.49	77643.89	94597.93
**7.88**	69394.27	77091.05	59949.86	77946.95	94908.88
**7.9**	69564.18	77265.32	60176.23	78371	95265.95
**7.92**	69641.21	77421.61	60380.43	78697.93	95660.88
**7.95**	69708.2	77628.69	60660.12	79002.48	96070.6
**7.97**	69902.96	77951.03	60911.57	79453.63	96601.16
**7.99**	70100.43	78138.79	61173.01	79845.6	97008.34
**8.01**	70217.68	78364.27	61360.41	80187.5	97483.26

**8.04**	70343.28	78514.37	61600.45	80612.08	97936.17
**8.06**	70457.17	78719.7	61888.46	80940.42	98339.64
**8.08**	70647.97	78941	62128.21	81375.56	98885.18
**8.11**	70811.03	79144.62	62422.95	81788.63	99262.9
**8.13**	70953.52	79360.54	62569.68	82097.27	99753.09
**8.15**	71021.1	79557.39	62861.1	82491.55	100195.7
**8.18**	71230.51	79812.4	63123.49	82960.33	100719.9
**8.2**	71354.36	80101.22	63390.38	83316.49	101203.7
**8.22**	71548.32	80264.63	63671.4	83740.74	101685.5
**8.24**	71668.37	80442.24	63940.84	84213.35	102121
**8.27**	71852.53	80672.47	64206.65	84573.36	102733.1
**8.29**	72020.45	80905.64	64440.03	85039.62	103262
**8.31**	72129.66	81165.62	64675.77	85412.72	103765.2
**8.34**	72363.85	81379	65013.77	85907.48	104328.4
**8.36**	72440.23	81600.89	65286.92	86296.94	104869.5
**8.38**	72601.37	81821.99	65498.53	86702.1	105414.5
**8.41**	72770.93	82022.86	65783.77	87174.25	105908.2
**8.43**	72945.08	82244.75	66026.97	87604.82	106422.4
**8.45**	73102.09	82468.38	66268.97	88065.33	107015.1
**8.48**	73224.19	82677.66	66566.25	88482.97	107555.7
**8.5**	73333.37	82904.07	66840.07	88930.97	108112.6
**8.52**	73502.16	83136.33	67046.76	89334.6	108785.6
**8.54**	73670.4	83323.13	67319.38	89768.01	109396.7
**8.57**	73849.75	83570.11	67575.49	90273.15	109992
**8.59**	74031.29	83875.7	67905.81	90748.28	110609.7
**8.61**	74187.15	84053.14	68173.55	91185.04	111194.5
**8.64**	74373.34	84326.32	68481.88	91668.87	111810.1
**8.66**	74553.48	84583.46	68713.95	92166.11	112415
**8.68**	74683.71	84751.95	69005.8	92584.64	113044.2
**8.71**	74847	84984.68	69275.32	93058.71	113703.2
**8.73**	75041.56	85282.78	69571.59	93556.67	114306
**8.75**	75223.43	85424.36	69817.68	93982.08	114928.7
**8.78**	75351.42	85702	70142.08	94470.12	115600.2
**8.8**	75466.01	85884.75	70313.62	94925.26	116203.3
**8.82**	75634.92	86119.65	70631.69	95372.88	116839.4
**8.84**	75774.32	86425.15	70929.61	95880.19	117511
**8.87**	75926.78	86589.03	71178.62	96350.52	118099.9
**8.89**	76147.33	86811.15	71470.94	96756.71	118795.5
**8.91**	76313.4	87022.13	71726.13	97232.44	119402.3

**8.94**	76483.01	87270.32	72001.44	97719.17	120095.7
**8.96**	76657.56	87556.15	72342.6	98242.42	120838.9
**8.98**	76835.74	87771.32	72613.76	98716.23	121553.2
**9.01**	76990.82	88000.79	72818.74	99204.92	122169
**9.03**	77147.94	88158.58	73017.33	99636.29	122854.6
**9.05**	77235.16	88346.94	73318.02	99969.28	123472.9
**9.08**	77445.21	88586.86	73657	100523.2	124258.9
**9.1**	77627.9	88798.83	73868.99	100987.9	124855.5
**9.12**	77794.03	89049.04	74158.23	101487.4	125506
**9.14**	77982.78	89364.04	74475.53	102107.7	126338
**9.17**	78138.43	89615.09	74789.8	102574.8	127115.2
**9.19**	78343.56	89855.76	75029.75	103032.4	127761.5
**9.21**	78579.41	90191.41	75417.6	103566.1	128545.9
**9.24**	78725.49	90319.72	75696.99	104062.4	129267
**9.26**	78920.8	90681.46	75955.51	104602.8	130079.5
**9.28**	79138.02	90964.75	76263.42	105102.4	130795
**9.31**	79327.52	91234.2	76555.85	105709.9	131565.5
**9.33**	79597.84	91469.75	76896.8	106270.4	132319.1
**9.35**	79745.54	91714.85	77162.41	106708.8	133135.4
**9.37**	79949.7	91982.2	77487.09	107216.6	133930.6
**9.4**	80073.5	92272.91	77729.61	107691	134691
**9.42**	80311.56	92496.19	78005.47	108235.3	135459.6
**9.44**	80485.81	92673.11	78303.14	108749.7	136160
**9.47**	80679.11	92966.86	78610.88	109251	136939
**9.49**	80927.65	93250.16	78923.42	109747.6	137804.7
**9.51**	81156.18	93514.65	79219.76	110319.1	138694.3
**9.54**	81306.46	93760.81	79487.01	110729.3	139216.9
**9.56**	81381.66	93959.77	79675	111203.1	139847.3
**9.58**	81627.88	94148.87	79951.57	111691	140627.4
**9.61**	81841.1	94409.48	80235.65	112151.2	141380.6
**9.63**	82032.93	94697.6	80566.4	112709.8	142250.3
**9.65**	82190.55	94954.66	80868.33	113168.9	142996.7
**9.67**	82438.23	95150.85	81194.44	113737.1	143785.5
**9.7**	82665.62	95546.24	81455.81	114246.7	144621.5
**9.72**	82859.39	95762.32	81704.43	114763.8	145355
**9.74**	83037.44	95981.53	81984.81	115216.9	146063.5
**9.77**	83278.69	96217.19	82341.77	115712.5	146822.3
**9.79**	83423.3	96486.93	82568.66	116179.7	147634.4
**9.81**	83671.16	96889.66	82837.03	116774.4	148513.3

**9.84**	83830.85	97070.73	83179.63	117207.4	149197.5
**9.86**	84072.04	97359.26	83478.54	117757.2	150049.7
**9.88**	84258.65	97586.89	83730.43	118333.9	150772.4
**9.91**	84480.18	97863.62	84039.82	118799.9	151635.4
**9.93**	84643.21	98133.52	84315.62	119253.3	152442.1
**9.95**	84910.02	98363.96	84630.75	119775.1	153170.1
**9.97**	85108.17	98666.97	84829.54	120226.2	153918.6
**10**	85265.52	98999.16	85199.65	120719.6	154798.4
**10**	85490.21	99187.98	85468.48	121257.1	155527.4
**10**	85730.36	99466.12	85768.22	121766	156279.3
**10.1**	85968.22	99787.5	86116.93	122275.8	157147.6
**10.1**	86190.83	100091.2	86354.18	122769.2	157909.5
**10.1**	86364.53	100329.6	86722.16	123248.7	158714.4
**10.1**	86598.74	100596.6	86963.9	123741.4	159508.8
**10.2**	86827.8	100868.5	87225.42	124315.7	160311.8
**10.2**	87078.46	101143.8	87520.2	124817.9	161118.9
**10.2**	87287.17	101433	87833.52	125302.2	161841.5
**10.2**	87526.58	101796.6	88105.29	125844	162601.1
**10.3**	87735.21	102050.3	88407.38	126299.9	163450.5
**10.3**	88006.22	102334.5	88702.4	126758.4	164253.7
**10.3**	88219.11	102605.6	89031.67	127331.7	164975.6
**10.3**	88411.63	102859	89335.02	127806.9	165772.1
**10.3**	88669.99	103163.3	89624.53	128350.3	166550.1
**10.4**	88915.28	103495.1	89956.18	128829.4	167358.1
**10.4**	89069.91	103759.4	90180.76	129296.9	168054.2
**10.4**	89356.99	104031.9	90442.26	129780.7	168877.7
**10.4**	89610.78	104329.5	90726.06	130218.4	169692
**10.5**	89835.09	104621.2	91044.7	130703.9	170390.6
**10.5**	90057.93	104963.3	91362.3	131180.7	171129.8
**10.5**	90300.73	105207.8	91658	131643.6	171955
**10.5**	90585.86	105476.4	91934.92	132184	172712.7
**10.6**	90744.39	105778.8	92235.96	132675.7	173388.7
**10.6**	91042.79	106175.4	92536.06	133169.5	174278.5
**10.6**	91264.71	106438.4	92800.23	133671.5	175094.2
**10.6**	91469.52	106652.5	93084.13	134109.3	175703.6
**10.6**	91689.66	106943.9	93372.48	134565.4	176380.3
**10.7**	91936.46	107251.2	93688.42	135064.9	177201.2
**10.7**	92191.12	107542.7	93939.27	135527.9	177993
**10.7**	92447.95	107895.5	94235.88	136005.5	178690.3

**10.7**	92653.88	108142.4	94548.65	136535.2	179409
**10.8**	92904.48	108487.3	94857.71	136947.1	180175.1
**10.8**	93208.99	108781.5	95125.32	137497.3	181056.3
**10.8**	93438.47	109105.7	95486.94	137949	181734.2
**10.8**	93648.38	109330.1	95722.35	138343.4	182436.9
**10.9**	93898.4	109680.6	95966.19	138811.5	183060
**10.9**	94174	110034.5	96311.49	139366.2	183853
**10.9**	94485.49	110365.9	96571.05	139830.6	184647.3
**10.9**	94713.07	110646.4	96896.95	140278.3	185425.3
**10.9**	94944.34	111017.2	97221.4	140804.2	186096.6
**11**	95199.67	111319.8	97584.39	141262.8	186845.7
**11**	95513.07	111659.7	97848	141846.7	187625.3
**11**	95782.5	111968	98161.11	142274.5	188395.7
**11**	96019.17	112205.2	98424.2	142666.7	189119.9
**11.1**	96202.71	112466.1	98639.04	143040.1	189727.5
**11.1**	96477.07	112753.3	98950.04	143464.4	190315.7
**11.1**	96742.28	113106.9	99244.01	143923.1	191091.2
**11.1**	97007.43	113370.1	99515.14	144395.1	191785.4
**11.2**	97303.21	113767	99865.58	144860.7	192552.9
**11.2**	97558.43	114045.4	100165.4	145297	193193
**11.2**	97885.87	114366	100423.1	145790.3	193909.6
**11.2**	98098.12	114746.5	100734.6	146291.6	194636.7
**11.2**	98403.06	115025.6	101022.2	146717.9	195410.7
**11.3**	98693.26	115398.1	101354.8	147248.2	196099.8
**11.3**	98979.23	115718.8	101723.2	147707.7	196847.4
**11.3**	99217.31	116051.9	102022	148118.9	197565.8
**11.3**	99520.4	116382.9	102293.9	148588.9	198247.5
**11.4**	99772.25	116712.1	102550.9	149056	198842.4
**11.4**	100040.1	117052.5	102870.9	149451.4	199570.6
**11.4**	100284.2	117355.1	103143	149937	200178.8
**11.4**	100666.8	117784.5	103479.7	150438	200942.5
**11.5**	100925.9	118087.2	103751.1	150797.5	201652.5
**11.5**	101206.2	118370.9	104032	151294.4	202421.8
**11.5**	101477.4	118757.1	104327	151660.4	203001
**11.5**	101798.8	119112.5	104658.8	152150.3	203760.5
**11.5**	101979.6	119397.7	104888.5	152507.2	204292.6
**11.6**	102276.1	119623.7	105133.3	152944	204916.5
**11.6**	102514.3	119957.8	105450.5	153392.2	205616.9
**11.6**	102762.6	120305.1	105707.3	153755.8	206168.8

**11.6**	103137.2	120705	106045.1	154286	207057.9
**11.7**	103417.4	120992	106349.9	154769.8	207650.1
**11.7**	103666.1	121312.8	106629.1	155118.4	208299.4
**11.7**	104053.5	121741.4	106947.9	155594.6	209055.2
**11.7**	104377.4	122061.7	107229.4	156080.8	209657.2
**11.8**	104646.5	122392.3	107549.3	156449.2	210307.3
**11.8**	104862.3	122689.9	107765.3	156779.1	210882.4
**11.8**	105205.7	123045.6	108056.2	157274.6	211582.1
**11.8**	105521	123395.2	108402.5	157728.5	212189.4
**11.8**	105800.5	123726.4	108657.7	158117.5	212864.1
**11.9**	106151.9	124088	108977.6	158525.7	213523.6
**11.9**	106398.3	124413.2	109264	158996.3	214232.4
**11.9**	106655.9	124671.8	109504.9	159317.1	214844.7
**11.9**	106963.1	125086.5	109827.9	159809.4	215536.1
**12**	107280.3	125396.8	110172.8	160231.5	216131.7
**12**	107567.7	125776.9	110481	160629.3	216757
**12**	107886.4	126162	110738.9	161168.7	217413.4
**12**	108193.6	126527.5	111033.3	161556.1	218132.9
**12.1**	108506.1	126868.9	111357	161938	218694.2
**12.1**	108821.2	127158.7	111583.5	162299	219369.6
**12.1**	109142.9	127523.9	111858.7	162721.2	219965.8
**12.1**	109505.2	127853.5	112220.6	163233.2	220722.2
**12.1**	109767.2	128210.6	112472.9	163570.4	221273.2
**12.2**	110101.8	128579.7	112704.8	164019.3	221938.3
**12.2**	110404.9	128902.1	113034.3	164402.4	222515.2
**12.2**	110692.4	129198.6	113302.6	164812.4	223161.8
**12.2**	110980.9	129636	113670.1	165215.7	223816.4
**12.3**	111353	129910.2	113898.4	165632.1	224381.7
**12.3**	111591.2	130198.9	114155.9	165912.8	224973.2
**12.3**	111904	130518.1	114362.6	166323.2	225536
**12.3**	112251.8	130889.5	114659.7	166710.7	226179.8
**12.4**	112599.5	131289.3	114964	167145.1	226887.7
**12.4**	112829.8	131620.3	115219.8	167521.4	227402.1
**12.4**	113232.2	131947.5	115525.6	167949.4	228020.3
**12.4**	113537.8	132239.5	115784.5	168252.6	228614.4
**12.4**	113747.9	132547.3	115963.3	168572.8	229049.6
**12.5**	114029.6	132887.7	116279.6	168996.1	229752.1
**12.5**	114423.5	133278.7	116548.3	169414.1	230344.7
**12.5**	114706.3	133560.8	116796.7	169685.2	230790

**12.5**	115067.9	133895.9	117009.6	170057.2	231378.3
**12.6**	115339.6	134267.6	117306.7	170368.9	232035.3
**12.6**	115689.9	134530.7	117611.6	170774.1	232639.2
**12.6**	115984.7	134891.9	117786.2	171236.5	233094.4
**12.6**	116286.1	135212.1	118027.9	171455.6	233651.8
**12.6**	116622.2	135545.2	118334.8	171837.1	234178.4
**12.7**	116903.6	135790.3	118485.4	172089	234674.1
**12.7**	117177.7	136158.3	118739.6	172453	235187.3
**12.7**	117425.2	136403.7	118951.1	172785.4	235620.1
**12.7**	117780.3	136729.2	119168.3	173102.3	236138
**12.8**	118093.8	137014.2	119384	173296.7	236650.7
**12.8**	118386.2	137368.1	119668.3	173577.7	237174.2
**12.8**	118673.6	137611.1	119861.1	173848.8	237766.8
**12.8**	118992.9	137897.2	120040.8	174263.7	238141.9
**12.9**	119274	138178.3	120281.6	174552.9	238703.4
**12.9**	119602.5	138507	120537.4	174855.4	239124.3
**12.9**	119786.3	138743.1	120687.2	175053.7	239468.5
**12.9**	120128.2	139105.1	120916.5	175404.7	240041.7
**12.9**	120527	139395.2	121081.7	175737.9	240486
**13**	120751.9	139630.9	121243.5	175967.2	240890.9
**13**	121048.8	139976.4	121481.2	176108.9	241348.4
**13**	121417.1	140238.7	121680.7	176430.7	241734.1
**13**	121624.7	140406.5	121806.2	176604.8	242202.4
**13.1**	121846.6	140650.3	121985.8	176792.3	242541.4
**13.1**	122149.2	140998.3	122192.7	177057	242948.1
**13.1**	122512.2	141247.2	122293.1	177327.9	243305.5
**13.1**	122727.1	141501.5	122539	177566.5	243772.7
**13.2**	122993.4	141737.7	122700.5	177783.1	244106.7
**13.2**	123310.8	142089.4	122901.6	177974.8	244471.7
**13.2**	123563.8	142254.9	122996.6	178180.5	244816.2
**13.2**	123898.6	142480.7	123134.1	178375	245129.5
**13.2**	124192.1	142752	123252.3	178519.5	245489
**13.3**	124461.2	143022.2	123414.2	178744.5	245776.8
**13.3**	124790	143239.4	123575.8	178958.6	246033.6
**13.3**	125018.7	143436	123682.4	179049.8	246341.6
**13.3**	125281.9	143661.9	123818	179280.6	246703.3
**13.4**	125562.3	143841	123919.8	179350.9	246885.9
**13.4**	125835.4	144085.8	124027.1	179430.6	247151.7
**13.4**	126081.2	144315.7	124221.2	179640.8	247482.1

**13.4**	126289.9	144483.8	124252.6	179658.5	247598.4
**13.5**	126819.3	144902.3	124428.5	179958.9	248023.5
**13.5**	127092.3	145070.7	124512.6	180089.1	248261.1
**13.5**	127348.4	145262.7	124619.3	180120.3	248386.7
**13.5**	127626.4	145419.7	124676.2	180184.2	248556.7
**13.6**	127812.1	145551.4	124707.6	180222.4	248633.2
**13.6**	128077.9	145756.5	124801.5	180225.3	248745.2
**13.6**	128304.7	145929.6	124861.8	180333	248970.1
**13.6**	128563	146109.5	124908.8	180363.4	249115.2
**13.7**	128759.5	146291.9	124919.4	180452.8	249301.8
**13.7**	129011.8	146390.6	125001.5	180464.5	249253.7
**13.7**	129207	146468.2	125038.4	180449.4	249319
**13.7**	129447	146631.9	125049.8	180440.9	249334.4
**13.8**	129643.7	146806.5	124999.5	180431.7	249354.3
**13.8**	129935.4	146980.2	125107.8	180469.2	249498.7
**13.8**	130142.9	147083.9	125151.5	180452.4	249548.2
**13.8**	130424.1	147214.9	125141.8	180435.5	249611
**13.8**	130570.1	147287.9	125155.2	180449.3	249565.3
**13.9**	130799.7	147440.8	125147.9	180358.7	249559
**13.9**	131024	147593	125134.7	180344.4	249547.7
**13.9**	131181.3	147687.8	125169.9	180300.6	249473.2
**13.9**	131445.5	147720.6	125127.4	180192.8	249364.9
**14**	131638	147883	125132.3	180197.6	249364.7
**14**	131835.5	147993.7	125106	180079	249248.2
**14**	132059	147965.9	125021.2	179995.2	249117.8
**14**	132225.5	148133.1	125058.5	179917.2	249078.5
**14.1**	132423.7	148183.6	125043	179853.5	248916.3
**14.1**	132592.3	148251.4	124928.7	179736.6	248745
**14.1**	132773.7	148307.8	124827.6	179562.1	248535.4
**14.1**	132906.4	148360.2	124729.4	179356.7	248268.8
**14.1**	133147.3	148462.8	124669.7	179177.7	248159.5
**14.2**	133339.6	148519.4	124639	179035.6	247983.8
**14.2**	133547.6	148579.4	124686.4	178983.2	247856
**14.2**	133747.3	148687.3	124595.7	178825.6	247695.2
**14.2**	133958.9	148748.7	124530.9	178702.7	247506.9
**14.3**	134166.8	148752.3	124435.8	178521.9	247211.7
**14.3**	134358	148909.6	124439.1	178414.4	247070.3
**14.3**	134606.8	148924.3	124291.6	178251.6	246826.7
**14.3**	134733.4	148957.7	124211.4	178079	246491.2

**14.4**	134901.4	148948	124151	177885.9	246210.9
**14.4**	135108.6	148972.9	124018.2	177632.5	245840.9
**14.4**	135266.1	149030.8	123927.3	177512.6	245609.4
**14.4**	135436.2	149059.1	123847.1	177357.9	245320.3
**14.4**	135524.4	149096.6	123704.9	177059.8	244975.2
**14.5**	135736.6	149060.4	123581.7	176832.9	244649.5
**14.5**	135953.3	149014.2	123451.7	176585.4	244288.3
**14.5**	136057.2	149015.3	123302.3	176305.9	243910.3
**14.5**	136310	149106.3	123187.7	176148.5	243587.9
**14.6**	136318	148969.1	122978.2	175728.7	242905.4
**14.6**	136394.1	148853.2	122739.9	175357.3	242411.1
**14.6**	136516.9	148778.5	122575.2	175071.9	241996.7
**14.6**	136604.9	148686.4	122423.4	174728.3	241482.7
**14.7**	136742.9	148708.8	122275.9	174397	240964.2
**14.7**	136856.8	148659.7	122112.9	174154.8	240593.2
**14.7**	137058.3	148626.2	122009.2	173898.3	240103.8
**14.7**	137160.2	148587.3	121847.5	173560	239582.4
**14.7**	137366	148538.3	121672.5	173277	239163.9
**14.8**	137482.9	148511.3	121517	173003.9	238683.8
**14.8**	137616.9	148488.9	121378.5	172763.2	238167.4
**14.8**	137770.9	148483.7	121173.8	172437.2	237715.6
**14.8**	137975.9	148473.6	121046.4	172146.5	237297.8
**14.9**	138078.3	148433.7	120910.5	171830.2	236663.4
**14.9**	138164.9	148341.4	120683.9	171391.4	236106.3
**14.9**	138265.3	148213.4	120436.5	171066	235504.4
**14.9**	138389.8	148150.9	120216.1	170679.1	234831.2
**15**	138404.2	148003.9	119984	170242.9	234250.5
**15**	138551.5	147932.7	119836.6	169970.5	233771.6
**15**	138714.4	147829.8	119693.7	169600.8	233093.7
**15**	138812.3	147860.8	119509.1	169332.4	232526.7
**15**	138961.9	147726.9	119305.3	168918.3	231981.7
**15.1**	139034.3	147696.1	119058.1	168470.1	231333.5
**15.1**	139203.6	147633	118885.1	168165.7	230737.8
**15.1**	139237.3	147500	118624.5	167804.5	230037
**15.1**	139392.4	147515.9	118448.9	167437.3	229488.5
**15.2**	139509.2	147318.4	118172.1	167051.2	228868.3
**15.2**	139618.6	147253.7	117983.9	166679.7	228288.8
**15.2**	139761.9	147220.8	117872	166342	227638.5
**15.2**	139955.5	147228.9	117641.2	165998	227077

**15.3**	140038.5	147054.8	117427.9	165591.8	226458.8
**15.3**	140108.1	146928.9	117193.4	165189.1	225679.9
**15.3**	140269.9	146817.7	117023.6	164792.9	225148.4
**15.3**	140351.7	146766.8	116820.2	164407.8	224470.6
**15.3**	140477	146613.2	116556.8	164078.3	223787.7
**15.4**	140557.7	146569.4	116338.1	163672.6	223047.3
**15.4**	140748.3	146469.9	116105.4	163248	222429.7
**15.4**	140835.7	146411.2	115918.4	162785.9	221830.8
**15.4**	140898.9	146255.5	115682.5	162412.6	221115.9
**15.5**	140981	146139.2	115447.7	162022.2	220424
**15.5**	141157.1	146064	115282.5	161685.2	219754.7
**15.5**	141204.1	145955.3	115016.7	161239.4	219047.5
**15.5**	141286.2	145799.7	114763.9	160838.1	218388.7
**15.6**	141456	145791.8	114635.6	160501.3	217710.2
**15.6**	141561.1	145645.9	114326.2	160039.3	216928.1
**15.6**	141664.9	145514.9	114099.2	159676.4	216286.5
**15.6**	141822.6	145445.8	113915.5	159295.4	215517.2
**15.6**	141936.3	145373.8	113660.5	158908	214895.5
**15.7**	142067.3	145284.1	113510.4	158492	214338.7
**15.7**	142135.3	145112.2	113256.6	158090.6	213571.3
**15.7**	142254.2	144955.7	112985.8	157631.2	212811.1
**15.7**	142380.2	144905.9	112793.2	157247	212080.3
**15.8**	142550.6	144775.5	112578.6	156874.1	211440.2
**15.8**	142623.8	144696.6	112381.6	156439.5	210790.6
**15.8**	142692.4	144613.6	112155.6	156071.9	210023.4
**15.8**	142874.3	144496	111978	155652.9	209361.2
**15.9**	142959.2	144418.5	111726.5	155271.8	208635.9
**15.9**	143145.8	144358.6	111493.9	154850.9	207979.1
**15.9**	143205.5	144265.1	111295.6	154462.1	207234.9
**15.9**	143346.6	144155.3	111064.3	154051.4	206559.7
**15.9**	143429.6	144023.5	110839.4	153613.4	205861.2
**16**	143611.3	143939.4	110692	153271.6	205200.4
**16**	143694.6	143779.3	110454.6	152828.9	204414.1
**16**	143903.1	143739.5	110202	152422.9	203732.6
**16**	143972.3	143623.9	109990.5	152031.3	203043
**16.1**	144030.6	143488.4	109714.1	151652.3	202316.5
**16.1**	144109	143291.4	109455.7	151197.4	201469.4
**16.1**	144287.1	143266.1	109230.5	150800.6	200801.9
**16.1**	144465.1	143235.5	109089.2	150494.1	200322.5

**16.2**	144632.3	143171	108944.4	150130.5	199548.3
**16.2**	144706	143068.4	108709.2	149745	198919.4
**16.2**	144966.1	142991.4	108533.7	149415	198291.4
**16.2**	145129.2	142942.8	108340	149053.3	197559.5
**16.2**	145256.6	142803.3	108088.3	148591.8	196956
**16.3**	145361.9	142708.3	107906.9	148226.8	196189.5
**16.3**	145423.5	142515.1	107694.3	147784.9	195350.6
**16.3**	145580	142508	107510	147476.3	194690.8
**16.3**	145764.7	142426.7	107238.7	147020.7	194073.5
**16.4**	145971.3	142444.8	107116.4	146769.5	193429.1
**16.4**	146112.5	142361.3	106927.2	146353.7	192776.6
**16.4**	146284.1	142335.6	106698.8	146021.4	192035.2
**16.4**	146487.8	142268.5	106533.3	145702.7	191327.3
**16.5**	146650.2	142204	106356.7	145410.6	190723
**16.5**	146854.8	142162.7	106152	145025.6	190108.1
**16.5**	146976.4	142068.8	105996.5	144589.3	189345.5
**16.5**	147077.5	141961.7	105826.9	144177.8	188670.4
**16.5**	147230	141872.2	105553.4	143881.9	187954.9
**16.6**	147443.2	141839.8	105395.4	143510.3	187254.4
**16.6**	147655	141815.2	105268.2	143196.9	186661.7
**16.6**	147868.8	141735	105025.3	142846.9	186041.5
**16.6**	148022.2	141703.4	104892.7	142548.3	185479.9
**16.7**	148213.5	141670.8	104763.7	142177	184787.2
**16.7**	148436.9	141625.5	104588.7	141880.4	184205.7
**16.7**	148656.3	141655.3	104456.1	141599.1	183612.1
**16.7**	148755.3	141556.5	104159.1	141156.1	182901
**16.8**	149041.7	141571.7	103995.4	140935	182372.5
**16.8**	149322	141583.8	103934.8	140617.6	181848
**16.8**	149461.1	141475	103744.4	140198.4	181204.2
**16.8**	149635.7	141466	103559	139905	180526.6
**16.8**	149875.1	141425.8	103453.7	139609.7	180037.7
**16.9**	150099.8	141449.3	103252	139305.4	179391.7
**16.9**	150254.3	141394.4	103129	139041.1	178762.6
**16.9**	150440.4	141383.9	102923.3	138692.2	178188.7
**16.9**	150697	141388	102791	138430.1	177686.1
**17**	150859	141322.5	102619.1	138087.1	177007
**17**	151123.5	141314.4	102519.6	137753.3	176443.5
**17**	151380.4	141341.4	102314.8	137519	175941.8
**17**	151549.6	141270.9	102212.5	137154.7	175276.9

**17.1**	151738.6	141282.5	102068.7	136882.9	174720.5
**17.1**	152013.6	141329.2	101990.1	136552.9	174190.6
**17.1**	152254.8	141319.6	101826.3	136314.7	173613.7
**17.1**	152399.2	141289.2	101666.3	135941.2	172980.3
**17.1**	152633.8	141192.8	101552.7	135698.3	172463
**17.2**	152915.3	141250	101384.7	135441.6	171916.6
**17.2**	153172.7	141300.2	101271.1	135163.2	171381.1
**17.2**	153394.9	141226.9	101055.3	134828.2	170739.7
**17.2**	153492.7	141210.8	100935.8	134513.2	170165.4
**17.3**	153873.4	141301	100846.5	134323.5	169739.5
**17.3**	154103.3	141333.9	100726	134028.6	169256.2
**17.3**	154300.3	141322.2	100517	133724.2	168629.5
**17.3**	154561	141336.6	100452.7	133423.2	168085.5
**17.4**	154776.6	141320	100360	133163	167589.8
**17.4**	155007.1	141344.9	100200.2	132858.8	167041.9
**17.4**	155320	141345.4	100081.2	132616	166466.8
**17.4**	155596	141348.8	99989.35	132391	166008.6
**17.4**	155785	141315	99849.7	132119.5	165494.6
**17.5**	156002.5	141353.8	99720.64	131801.4	164987.6
**17.5**	156242.7	141433.4	99565.06	131592.9	164447.3
**17.5**	156526.2	141440.9	99527.31	131356.4	164025.1
**17.5**	156711.2	141369.4	99411.1	131026.7	163476.6
**17.6**	156971.7	141410.4	99239.68	130759.1	162989.8
**17.6**	157208.9	141518.9	99118.35	130506.9	162463.6
**17.6**	157500.7	141497.6	99041.27	130262.8	162007
**17.6**	157750.9	141526.2	98934.54	130038.1	161509.3
**17.7**	157916.6	141483.7	98728.8	129731.4	160927.5
**17.7**	158053.4	141477.6	98618.76	129423.7	160378.5
**17.7**	158392.6	141590.2	98565.24	129260.1	159964.9
**17.7**	158606.1	141628	98402.21	128967.7	159474.5
**17.7**	158847.8	141628.9	98326.35	128737.7	158971.2
**17.8**	159088.6	141653.8	98193.68	128510	158462
**17.8**	159286.7	141575.1	98044.9	128137	158008.9
**17.8**	159612.6	141729.1	97966.36	127965.6	157526.2
**17.8**	159775.6	141749.9	97891.41	127701.8	157083
**17.9**	159982.5	141664.6	97762.08	127456.2	156582.6
**17.9**	160308.1	141759.3	97696.83	127260.3	156177.4
**17.9**	160507.1	141742.7	97544.74	126963.1	155605
**17.9**	160825.1	141794.4	97470.47	126770.8	155239.7

**18**	160916.3	141819.4	97358.11	126532.9	154770.5
**18**	161181.9	141830.1	97226.66	126276.7	154241.4
**18**	161405.1	141835.8	97073.74	126076.1	153744.2
**18**	161723	141956.3	97036.07	125871.8	153354.9
**18**	161894.9	141919.8	96903.78	125621.3	152874.4
**18.1**	162118.2	141981.6	96818.75	125384.9	152455
**18.1**	162381.9	141992.5	96770.73	125181	152051.7
**18.1**	162607.1	142004.1	96607.84	124903.4	151580.1
**18.1**	162747.3	141975.1	96480.94	124673.2	151072.1
**18.2**	163020	142046.7	96356.02	124467	150659.9
**18.2**	163224	142035.5	96288.73	124183.5	150221.4
**18.2**	163454.3	142094.4	96213.1	123974.4	149834.9
**18.2**	163694	142127.3	96136.95	123804.7	149430.1
**18.3**	163835.2	142062.5	95975.27	123509.6	148896.7
**18.3**	164091.6	142135.9	95939.62	123284.5	148586.4
**18.3**	164291.9	142178.9	95804.9	123095.3	148088.9
**18.3**	164547.8	142268.2	95762.97	122905.3	147785.4
**18.3**	164740.8	142223.8	95600.7	122688.6	147268.2
**18.4**	164910.3	142223.4	95506.83	122479.1	146827
**18.4**	165148.9	142273.7	95404.36	122200.7	146429.5
**18.4**	165356.5	142251.1	95302.63	121977.7	146036.3
**18.4**	165496.7	142284.2	95208.36	121768.4	145628.9
**18.5**	165686.9	142324.4	95126.16	121615.7	145217.3
**18.5**	165878.4	142337.1	95047.49	121378.1	144752.4
**18.5**	166093	142339.3	94926.14	121195	144386.8
**18.5**	166198.4	142367.1	94800.87	120948.9	144020
**18.6**	166428.6	142348.4	94716.88	120769.1	143616.2
**18.6**	166602.4	142387	94655.11	120527.6	143215.8
**18.6**	166785.4	142368.8	94548.81	120369.6	142797.6
**18.6**	167029.6	142403.7	94470.96	120162.9	142436.2
**18.6**	167174.9	142424.4	94426.87	119928.2	142076.1
**18.7**	167284.8	142433.4	94257.53	119692.6	141634.5
**18.7**	167446	142419.6	94170.55	119410.3	141290.8
**18.7**	167643.9	142427.2	94057.51	119239.2	140850.9
**18.7**	167687.3	142301.8	93865.31	118998.2	140418.6
**18.8**	167924.8	142380.6	93837.09	118834.3	140104.9
**18.8**	168043.9	142325.1	93730.98	118581.5	139707.1
**18.8**	168157.1	142316.2	93635.51	118373.3	139314.9
**18.8**	168297.8	142322.6	93563.47	118223.9	138946.8

**18.9**	168444.7	142300.9	93439.28	117942.3	138564.3
**18.9**	168506.9	142253.1	93308.14	117731.8	138110.6
**18.9**	168516.4	142180.8	93148.68	117499.1	137661.4
**18.9**	168604.2	142161.5	93041.29	117282.9	137242.5
**18.9**	168699.2	142113.8	92920.42	116981.2	136823.1
**19**	168828.5	142010.7	92788.64	116713.8	136495.7
**19**	168775.8	141897.6	92605.51	116435.6	136136.7
**19**	168897.1	141838.1	92497.64	116220.3	135709.3
**19**	168935.3	141889.6	92447.96	116074.4	135454
**19.1**	169143.2	141859	92384.97	115959.8	135112.5
**19.1**	169289.9	141907.3	92312.57	115790.2	134793.2
**19.1**	169324.1	141890.7	92199.05	115532.3	134375.8
**19.1**	169394.9	141828.2	92050.72	115305.6	134056
**19.2**	169481.3	141794.7	91974.69	115145	133765.1
**19.2**	169621.5	141770	91853.31	114960.2	133448.9
**19.2**	169691.1	141679.7	91784.96	114721.7	133127.4
**19.2**	169713.7	141749.5	91680.57	114585.9	132809.6
**19.2**	169832.9	141600.6	91584.26	114336.9	132486.6
**19.3**	169843.9	141557.7	91427.75	114177.5	132136.1
**19.3**	169880	141452.1	91328.45	113944.6	131715.1
**19.3**	169910.5	141368	91209.9	113749.3	131349.4
**19.3**	169971	141386.2	91156.81	113525.3	131107.9
**19.4**	169982.8	141272.1	91019.77	113300.9	130709.8
**19.4**	170000.6	141199.9	90871.97	113132.8	130410.6
**19.4**	169964.5	141166.3	90753.43	112878.3	130046.6
**19.4**	169965.4	141061.6	90614.28	112625.6	129662.4
**19.5**	170031.3	140955.1	90558.49	112461.9	129384.1
**19.5**	170063.7	140887.6	90421.95	112272.2	129060.9
**19.5**	170043.2	140889.7	90356.26	112046.3	128738.2
**19.5**	170104.7	140827.2	90183.57	111923.6	128447.2
**19.5**	170107.3	140656	90062.4	111617.6	128081.9
**19.6**	169989.3	140527.1	89968.17	111459.3	127754.7
**19.6**	169998.6	140464.2	89819.77	111218.5	127447.4
**19.6**	169909.7	140333.7	89700.88	110930.2	127095
**19.6**	169873.8	140180.6	89538.6	110771.8	126752
**19.7**	169864.1	140143.3	89490.81	110624.2	126467.1
**19.7**	169821.2	140033.8	89388.09	110385.4	126058.9
**19.7**	169743.1	139969.6	89264.72	110189.5	125841.3
**19.7**	169751.6	139786.5	89113.94	109990.5	125581.7

**19.8**	169684.9	139689.6	88936.04	109814.3	125208.6
**19.8**	169627.3	139545.1	88848.32	109598	124854.6
**19.8**	169565.4	139528.9	88743.84	109369.6	124616.1
**19.8**	169521.6	139392.6	88622.35	109150.4	124349.6
**19.8**	169464.3	139232.2	88490.81	108993.2	123988.8
**19.9**	169309.6	139113.3	88316.08	108779.4	123638.9
**19.9**	169223.3	138921.4	88162.23	108491.1	123285.2
**19.9**	169106.2	138788.8	88015.1	108335.2	123024
**19.9**	168991.1	138658.9	87982.97	108116.8	122726.3
**20**	168887.7	138566.8	87794.26	107847.3	122377.4
**20**	168911	138452.3	87700.99	107698.4	122112.7
**20**	168782	138266	87550.49	107524	121831.1
**20**	168652.9	138149.3	87404.3	107280	121536.5
**20.1**	168534	137956	87284.77	107084.9	121210.2
**20.1**	168421.3	137789.3	87108.22	106865.1	120929.8
**20.1**	168242	137636.6	86924.09	106659.6	120597.8
**20.1**	168102.2	137581.9	86893.6	106474.5	120347.7
**20.1**	167997.4	137366.3	86707.7	106240.2	120014.5
**20.2**	167852.2	137196.4	86586.42	105959.4	119728.7
**20.2**	167709.3	136993.7	86433.11	105821.6	119466
**20.2**	167549.7	136876.2	86289.9	105579.2	119144.5
**20.2**	167385.8	136595.1	86182.61	105383	118855.6
**20.3**	167225.2	136463.9	86025.64	105176.5	118562
**20.3**	167034.5	136304.2	85878.74	104956.9	118267.1
**20.3**	166915	136141.8	85731.83	104758	118039.5
**20.3**	166752.6	136006.6	85608.31	104605.1	117713.8
**20.4**	166576.1	135802.8	85425.99	104336.4	117441.6
**20.4**	166311.4	135551.8	85276.64	104032.3	117091.9
**20.4**	166129	135315.1	85097.23	103879.9	116803.8
**20.4**	165990.1	135199.9	84981.54	103714.1	116536.7
**20.4**	165784.5	135027	84810.11	103489	116271.5
**20.5**	165588.8	134852.5	84680.97	103295.9	115933.3
**20.5**	165395.9	134617	84527.91	103067	115725.8
**20.5**	165189.4	134430.6	84360.71	102844	115435.9
**20.5**	164889.2	134159.4	84156.31	102597.8	115088.6
**20.6**	164703.7	133955	84077.75	102377.8	114830
**20.6**	164526.7	133691.5	83923	102244.7	114566
**20.6**	164303.4	133469.1	83692.23	102002.4	114226.6
**20.6**	164063.5	133251.7	83549.49	101740.8	113941.2

**20.7**	163812.4	133092.3	83424.51	101525	113691.1
**20.7**	163560.9	132865.8	83263.98	101286.7	113426.1
**20.7**	163307.6	132625.8	83135	101123.6	113222.4
**20.7**	163043.1	132449.6	82936.69	100839.2	112872.4
**20.7**	162799.9	132182.3	82800.44	100709.6	112616.9
**20.8**	162514.8	131862.9	82607.04	100412.5	112303.3
**20.8**	162341.7	131701.5	82410.23	100195.9	112039.4
**20.8**	162064.9	131438.9	82312.29	99984.58	111772.7
**20.8**	161714.7	131132.7	82080	99733.09	111409.9
**20.9**	161418	130910.9	81923.81	99530.67	111113
**20.9**	161278	130680.7	81799.85	99336.36	110852.8
**20.9**	160943.4	130454.4	81631.06	99188.81	110681.5
**20.9**	160750.9	130273.1	81477.49	98967.03	110377.6
**21**	160513.8	130045.4	81331.65	98730.91	110108.5
**21**	160190.1	129821.2	81125.02	98484.01	109901.3
**21**	159923.8	129559.7	80963.31	98286.38	109560
**21**	159571.7	129332.2	80793.03	98098.9	109272.6
**21**	159435.8	129054.4	80631.88	97921.61	109066.5
**21.1**	159115.5	128845.2	80473.06	97725.37	108782.3
**21.1**	158830.2	128578.4	80314.08	97447.37	108599.8
**21.1**	158572.4	128289.8	80143.62	97232.12	108264.3
**21.1**	158261.9	128082.7	79966.12	97079.41	108074.2
**21.2**	157944.9	127923.8	79825.26	96838.35	107755.4
**21.2**	157771.4	127673.5	79638.27	96662.87	107570.7
**21.2**	157413.2	127379.7	79541.98	96417.53	107267.1
**21.2**	157052.1	127071.7	79341.03	96157.3	106983.6
**21.3**	156811.3	126893.8	79156.69	96013.75	106789.6
**21.3**	156556.6	126640.8	79011.48	95854.51	106566.3
**21.3**	156196.5	126277.7	78794.54	95477.94	106198.3
**21.3**	155913.4	126034.8	78676.89	95311.41	106062.9
**21.3**	155618.5	125763	78491.61	95159.88	105770.3
**21.4**	155263.2	125471.1	78273.15	94941.44	105530.4
**21.4**	154954.6	125264.5	78101.8	94682.29	105292.9
**21.4**	154571.5	124952.7	77959.83	94466.04	105012.2
**21.4**	154404.8	124712.7	77834.82	94319.25	104894.8
**21.5**	154052.7	124446.8	77591.92	94108.36	104621.9
**21.5**	153676.2	124141.3	77340.63	93779.63	104236.5
**21.5**	153440.6	123913.5	77229.56	93611.95	104029.8
**21.5**	153063.2	123572.2	77088.54	93368.84	103777.2

**21.6**	152703.6	123360.6	76903.8	93191.01	103572.4
**21.6**	152392.6	123043.6	76690.62	92973.94	103318.8
**21.6**	152181.6	122851	76572.57	92825.65	103066.1
**21.6**	151711.9	122529.2	76350.38	92525.12	102767.9
**21.6**	151539.1	122315.7	76241.97	92355.9	102601.8
**21.7**	151115.4	121976.5	76056.22	92161.55	102404
**21.7**	150873.6	121808.5	75929.29	91941.58	102170.2
**21.7**	150437.5	121481.8	75729.9	91694.11	101831
**21.7**	150227.1	121245.6	75548.44	91493.13	101701.7
**21.8**	149983	120959.1	75390.96	91287.62	101441.3
**21.8**	149585.9	120733.2	75219.16	91159.75	101240
**21.8**	149263.2	120412.8	75020.96	90932.05	101030.1
**21.8**	149019.2	120189.7	74892.25	90711.61	100791.6
**21.9**	148737.3	119924.3	74673.18	90505.33	100539.5
**21.9**	148319.4	119672.8	74504.73	90319.31	100358.7
**21.9**	147932.6	119336.9	74369.43	90111.18	100076.4
**21.9**	147715.2	119116.9	74145.48	89913.05	99807.4
**21.9**	147287.1	118816.7	74004.07	89689.64	99559.9
**22**	147046.5	118585.5	73921.32	89543.8	99453.49
**22**	146566	118261	73607.54	89134.34	99134.64
**22**	146357.9	118014.4	73496.33	89069.58	98974.48
**22**	146025	117718.6	73330.2	88832.4	98754.84
**22.1**	145761.9	117449.5	73194.13	88703.37	98556.89
**22.1**	145203.7	117050	72853.37	88371.61	98188.31
**22.1**	144902.8	116762.4	72711.1	88167.23	97876.56
**22.1**	144523.5	116541.9	72622.14	87940.29	97737.17
**22.2**	144222.5	116225.9	72423	87759.92	97498.61
**22.2**	143861.1	115930.6	72227.95	87513.6	97317.78
**22.2**	143590.1	115657.4	72043.63	87337.14	97118.33
**22.2**	143305.8	115411.6	71941.22	87136.47	96889.79
**22.2**	142950	115122.7	71734.56	86872.89	96671.47
**22.3**	142502	114767.1	71588.1	86634.06	96366.31
**22.3**	142187.8	114635.1	71587.22	86504.03	96141.22
**22.3**	141883.8	114308.3	71323.29	86363.86	95963.91
**22.3**	141429.5	113946.9	70985	86052.07	95682.81
**22.4**	141296.7	113799	70834.38	85920.94	95550.5
**22.4**	139896.4	112563.7	70023.21	84924.25	94691.37
**22.4**	125473.3	100562.5	62118.03	75518.67	85183.62
**22.4**	110625.3	88958.97	55221.14	67101.52	74596.55

**22.5**	104198.5	83855.77	52101.48	63301.03	70061.69
**22.5**	93167.83	74944.81	46445.44	56430.58	62503.73
**22.5**	76364.05	61381.67	37933.56	46097.93	51167.69
**22.5**	62443.56	50132.42	31121.73	37786.09	41983.46
**22.5**	54171.71	43661.79	27175.22	32940.37	36732.08
**22.6**	53974.35	43450.6	27066.09	32840.76	36563.86
**22.6**	49699.82	39899.97	24753.4	30080.85	33806.45
**22.6**	42592.66	34185.47	21071.33	25713.85	28991.75
**22.6**	35010.34	28159.36	17416.96	21276.05	23519.14
**22.7**	33037.18	26620.42	16516.3	20092.04	22126.86
**22.7**	17262.98	13686.19	8286.045	10131.69	11413.4
**22.7**	3384.296	2645.633	1565.637	1946.507	2249.283
**22.7**	548.2232	446.5273	282.8078	375.3344	445.417
**22.8**	417.3751	351.14	243.2997	296.92	364.4324
**22.8**	399.0036	335.9976	233.9699	294.5429	362.0058
**22.8**	394.862	335.3602	228.5577	290.6452	351.3931
**22.8**	385.3201	330.8034	222.8922	283.5718	343.0532
**22.8**	375.3704	320.0983	216.7108	278.8795	341.483
**22.9**	366.471	316.0652	217.1128	277.3887	327.48
**22.9**	362.8123	318.4592	212.3508	269.3415	324.8318
**22.9**	360.6324	312.2986	209.1592	268.9804	324.9986
**22.9**	359.4939	311.0719	211.4008	267.8499	322.4238
**23**	357.297	304.8508	205.672	264.6034	322.8793
**23**	354.724	305.5221	204.1052	264.0992	316.0813
**23**	352.126	301.1314	205.1754	263.9324	315.0593
**23**	343.2906	299.6724	199.5213	259.4959	311.0794
**23.1**	349.6546	301.7007	200.8929	262.9115	315.2345
**23.1**	348.2204	301.2234	200.4212	256.9835	310.7679
**23.1**	346.0296	297.4615	196.8213	251.4522	308.9362
**23.1**	341.0285	297.2866	195.7069	251.4047	310.8788
**23.1**	339.9852	291.8119	195.3061	250.1347	308.3628
**23.2**	332.8122	289.8497	192.7231	250.2963	300.2762
**23.2**	333.0483	289.3131	192.2989	250.8278	295.5736
**23.2**	337.6078	290.5592	195.4739	251.0964	299.4725
**23.2**	329.7947	284.9158	193.4338	245.5152	296.6405
**23.3**	324.6663	279.0197	189.2775	243.0793	294.5861
**23.3**	325.1904	279.1866	190.0013	241.3033	296.6648
**23.3**	319.9612	274.4338	188.5063	242.3259	289.982
**23.3**	319.9931	280.089	191.4565	241.1328	293.23

**23.4**	316.1411	275.9537	183.3674	236.3192	285.6496
**23.4**	316.6163	272.1824	184.9101	237.7672	284.6324
**23.4**	310.7731	269.851	179.2315	236.157	283.854
**23.4**	306.617	270.0938	178.161	235.1226	282.0596
**23.4**	305.2306	267.4732	175.6495	231.1059	278.6408
**23.5**	305.889	263.9637	178.476	237.77	277.2198
**23.5**	302.9436	263.7196	173.6252	227.4856	276.9183
**23.5**	298.4172	261.6032	176.4845	224.3132	267.1325
**23.5**	291.472	253.0195	176.6834	227.8459	271.0476
**23.6**	295.5286	256.1824	177.1944	227.0304	267.2936
**23.6**	285.2304	249.565	169.5367	221.7447	268.3195
**23.6**	278.6359	250.7526	174.1463	218.0556	260.9259
**23.6**	284.9314	243.2857	175.1338	213.7215	265.1042
**23.7**	283.8566	246.0482	173.517	212.1077	259.9461
**23.7**	276.7883	241.0386	165.5169	210.9037	258.1949
**23.7**	275.1661	240.7224	161.9994	209.8027	255.7147
**23.7**	270.4593	234.4363	159.4273	210.4509	253.0001
**23.7**	263.9899	238.7434	163.3062	209.2726	251.9022
**23.8**	266.8987	235.1421	161.7256	207.9942	244.532
**23.8**	265.0863	228.269	154.1093	207.0358	246.5314
**23.8**	259.5306	226.962	155.5427	204.1198	245.1963
**23.8**	258.9367	228.7385	152.5155	203.1045	244.7478
**23.9**	258.3184	226.5325	151.4753	202.2718	239.7765
**23.9**	251.4668	224.4944	151.0246	199.3018	236.637
**23.9**	252.3777	217.8883	149.6389	192.3939	234.0568
**23.9**	250.5461	209.3979	147.7937	190.4153	232.1433
**24**	247.4359	216.2348	145.1135	194.568	232.0227
**24**	243.9782	218.0629	142.8434	187.4452	226.1069
**24**	245.1146	212.9239	142.903	187.581	228.0501
**24**	236.4395	211.0962	144.8573	186.9324	224.78
**24**	241.6722	213.0317	143.418	186.5373	226.9253
**24.1**	238.2357	214.2869	145.3905	183.9351	227.388
**24.1**	233.283	209.8023	138.1085	179.9566	221.9392
**24.1**	230.2062	203.187	137.3554	180.069	217.1725
**24.1**	224.2722	197.2503	138.693	176.5651	218.4599
**24.2**	227.9416	198.562	132.2738	175.6232	214.5204
**24.2**	223.6725	198.474	134.3384	176.4767	218.7196
**24.2**	221.1638	194.5188	134.6401	173.7206	214.286
**24.2**	224.9918	192.6156	133.8301	170.2495	211.2713

**24.3**	222.4452	187.9303	130.8892	169.2427	209.5507
**24.3**	217.8171	190.2719	132.1365	171.6959	214.7722
**24.3**	218.6398	187.3401	130.0472	170.8499	211.8232
**24.3**	211.2925	185.4976	126.3995	168.0575	209.9267
**24.3**	209.4062	186.7072	131.0807	173.5108	199.3846
**24.4**	212.9588	187.1969	127.334	161.3071	200.3268
**24.4**	209.9179	184.4739	124.6134	163.8869	198.544
**24.4**	208.5109	185.5169	128.0865	168.6609	202.1309
**24.4**	209.4777	181.2817	123.5534	158.3887	199.3366
**24.5**	214.2036	176.6079	123.8988	168.1283	205.0497
**24.5**	209.577	177.5303	127.0708	161.5523	202.7173
**24.5**	204.6184	179.0327	120.4574	160.0618	200.3496
**24.5**	201.1215	179.9541	122.5568	158.8835	199.8124
**24.6**	205.5797	178.3359	122.1327	156.783	199.2852
**24.6**	196.016	179.887	121.7776	158.3474	194.2978
**24.6**	201.9993	177.5981	120.9334	159.7792	202.2814
**24.6**	197.0387	174.0113	117.5776	162.3088	198.7058
**24.6**	194.3368	176.1841	117.8802	152.3802	193.0678
**24.7**	198.1026	174.6636	122.9203	152.7688	194.541
**24.7**	202.068	178.7371	121.2652	153.5669	196.2061
**24.7**	190.3181	171.1941	116.0418	157.5612	191.2897
**24.7**	193.0136	167.4637	116.2776	153.3381	185.9196
**24.8**	190.9047	168.025	115.6968	154.2614	187.9752
**24.8**	177.2546	160.834	110.5008	147.6503	179.1529
**24.8**	175.1516	162.2452	112.7963	153.5757	181.6202
**24.8**	175.4108	155.3098	112.6707	146.3814	179.3447
**24.9**	171.6831	160.2337	109.603	145.027	175.2218
**24.9**	170.3556	150.3976	107.4702	142.8313	163.5434
**24.9**	172.5298	142.6948	106.1417	139.1757	172.4014
**24.9**	162.5857	147.665	106.4611	141.4804	168.1463
**24.9**	163.1801	148.6399	105.6656	137.4666	162.6033
**25**	165.8146	145.8691	105.8907	141.702	165.1129
**25**	164.2707	145.1958	105.1807	140.5545	166.2335
**25**	157.4828	145.1468	102.6721	137.2406	167.2802
**25**	158.0854	145.4103	101.4348	132.259	163.31
**25.1**	156.6291	143.7655	103.7907	132.8774	163.9033
**25.1**	159.7153	144.3634	100.7515	133.3249	161.8646
**25.1**	154.6408	144.3329	99.52797	135.5519	160.1717
**25.1**	152.6657	132.186	95.75758	128.3343	157.032

**25.1**	155.6812	137.9944	94.47506	130.8996	159.8672
**25.2**	150.8944	141.4205	95.57477	127.6705	155.1458
**25.2**	150.8976	134.3375	100.4132	127.8181	151.51
**25.2**	151.7061	132.3867	95.71138	127.3808	151.9391
**25.2**	150.5165	134.0868	94.69148	128.9327	152.1045
**25.3**	151.2742	132.8228	93.90067	126.985	147.3581
**25.3**	147.8007	132.3665	90.64201	129.4926	151.5156
**25.3**	144.48	128.5831	90.9093	122.0253	150.3743
**25.3**	143.1582	131.087	93.46932	124.3072	151.7125
**25.4**	137.2504	129.4104	88.98663	125.4488	148.7893
**25.4**	137.632	128.386	92.36454	117.6706	144.1752
**25.4**	142.9865	127.372	87.49225	116.6606	139.9831
**25.4**	143.4236	130.6145	87.44068	120.8176	145.4812
**25.4**	142.3428	123.4899	90.78931	119.5159	138.8286
**25.5**	143.5064	122.825	92.05762	117.9388	143.7266
**25.5**	136.3896	121.2418	85.43839	116.2008	140.5941
**25.5**	136.9627	123.5126	90.08272	111.999	138.9553
**25.5**	141.7456	123.9025	87.11009	112.8635	143.5978
**25.6**	131.1286	120.8906	84.15997	110.2564	140.6532
**25.6**	137.6415	124.3889	86.34868	115.3984	136.6964
**25.6**	132.6371	128.857	85.25286	110.3847	133.5663
**25.6**	133.2627	123.6812	89.21112	110.0156	140.8884
**25.7**	131.2578	121.8682	89.43765	111.3293	140.4141
**25.7**	131.3558	119.4513	82.23855	112.3728	142.0203
**25.7**	125.1475	118.1045	86.68962	109.224	130.4114
**25.7**	127.4753	120.694	82.32445	108.4685	137.4371
**25.7**	125.0266	110.9912	81.22672	111.6867	134.5129
**25.8**	122.3557	109.0124	77.63353	113.8979	136.4392
**25.8**	125.6431	107.217	80.01044	103.4639	141.7489
**25.8**	122.0437	109.262	74.65735	106.1536	132.7536
**25.8**	119.818	110.3616	76.45203	105.8691	128.0731
**25.9**	118.0575	109.5051	73.32335	98.83727	126.7023
**25.9**	123.2206	109.9419	75.85713	108.1263	126.4696
**25.9**	117.5698	102.1566	78.40003	108.9322	130.7196
**25.9**	113.5968	102.2989	78.66652	102.685	122.3776
**26**	118.5168	109.945	74.33172	111.353	124.7943
**26**	119.8979	112.4847	76.69669	108.0312	132.3166
**26**	115.6533	112.3315	76.84946	99.89422	126.9852
**26**	108.912	109.679	76.8398	98.28751	123.2516

**26**	108.0619	105.6723	77.21253	109.393	122.5443
**26.1**	116.9075	99.51632	73.00436	100.8231	125.8973
**26.1**	118.8097	99.15827	69.96069	100.5505	113.6601
**26.1**	116.357	103.1597	80.31053	102.5397	118.1412
**26.1**	112.7916	103.5802	81.31047	100.4301	127.6275
**26.2**	107.4639	100.4713	77.37115	99.91959	123.3245
**26.2**	105.3724	107.3617	74.86137	99.48151	117.2915
**26.2**	102.013	106.286	78.14184	98.76301	122.5864
**26.2**	102.6981	100.3596	70.47619	88.7164	119.0562
**26.3**	106.4975	108.1661	70.2171	94.73779	118.0526
**26.3**	109.2122	106.649	79.44095	97.36419	123.6572
**26.3**	102.0455	95.08362	99.69862	96.97294	123.4807
**26.3**	102.6754	100.8239	112.0559	101.1292	125.3177
**26.3**	104.6383	88.99803	100.6459	92.35796	124.5512
**26.4**	103.251	90.08402	83.13844	89.63131	122.809
**26.4**	97.65094	96.26353	69.99214	88.74798	121.4671
**26.4**	98.61817	91.16242	69.07158	86.44097	117.3576
**26.4**	96.24524	81.47808	64.09045	92.05	121.2739
**26.5**	96.09486	85.1216	63.18918	87.64935	126.1743
**26.5**	103.3045	92.37273	63.13724	88.67046	128.4387
**26.5**	102.1167	88.19787	65.37939	84.93783	125.9254
**26.5**	93.56615	87.78232	61.21726	85.02286	113.4639
**26.6**	96.08531	90.13736	60.31786	91.04896	111.0116
**26.6**	97.54652	81.08288	59.82814	91.94002	104.9392
**26.6**	101.9708	82.25913	68.15308	87.53986	117.0647
**26.6**	92.28688	83.74048	66.64742	92.64346	117.7076
**26.6**	95.80923	90.48706	69.99577	89.24681	110.2727
**26.7**	104.9704	86.50848	65.05833	86.74787	101.4322
**26.7**	100.6992	90.57884	61.82845	84.61325	98.46624
**26.7**	97.12653	90.15472	63.36471	83.77238	98.86006
**26.7**	95.76956	83.06056	73.23937	76.90877	89.81036
**26.8**	93.67253	82.25705	62.21395	76.50094	88.76146
**26.8**	93.03788	66.96841	58.60463	66.74886	88.04854
**26.8**	87.23242	67.34854	61.5538	76.57147	88.11665
**26.8**	84.15161	79.29197	60.41546	87.4596	97.7155
**26.9**	86.70127	82.0315	60.41746	83.92615	109.1886
**26.9**	94.98924	72.43439	59.60665	78.32365	106.7612
**26.9**	79.16548	79.83749	55.11269	72.44592	92.40148
**26.9**	82.32517	74.82459	53.28852	75.66653	90.58481

**26.9**	75.52787	73.62521	52.3222	83.96915	97.56604
**27**	75.85093	83.17919	67.63325	81.93271	110.1424
**27**	79.89397	79.97615	68.72713	83.39226	117.8791
**27**	73.28885	75.4938	65.79376	78.88648	110.6599
**27**	81.87799	77.38412	66.11461	60.43562	89.29983
**27.1**	70.55956	78.99122	66.61032	60.2404	92.20736
**27.1**	77.45956	71.60908	54.43974	73.13255	98.3542
**27.1**	84.99359	82.71271	62.90662	86.27543	85.83607
**27.1**	67.36751	92.65536	58.84354	92.75821	87.50443
**27.2**	67.72883	85.68486	59.27829	84.07101	113.375
**27.2**	76.78788	75.97427	49.57674	83.43138	113.1641
**27.2**	106.2541	81.24843	54.01048	66.51835	107.0763
**27.2**	78.207	74.76974	48.97556	82.69447	103.0002
**27.2**	81.40983	68.51359	50.27277	95.58547	95.24046
**27.3**	110.9913	70.55664	49.79944	100.9303	76.76316
**27.3**	85.76019	96.34098	55.9453	93.52068	61.57439
**27.3**	76.61185	90.4017	48.13323	73.03877	96.81282
**27.3**	55.61152	84.79827	43.29306	70.32408	85.55804
**27.4**	70.30951	104.8742	46.34646	74.28973	65.99437

Peak I: highlighted in yellow; Peal I x $\sqrt{3}$: highlighted in green; Peak II: highlighted in blue; Peak III: highlighted in pink.
